# Reactions of Frustrated Lewis Pairs with Chloro‐Diazirines: Cleavage of N=N Double Bonds

**DOI:** 10.1002/anie.202209241

**Published:** 2022-08-03

**Authors:** Dipendu Mandal, Ting Chen, Zheng‐Wang Qu, Stefan Grimme, Douglas W. Stephan

**Affiliations:** ^1^ Institute of Drug Discovery Technology Ningbo University Ningbo 315211, Zhejiang China; ^2^ Department of Chemistry University of Toronto 80 St. George St Toronto ON M5S3H6 Canada; ^3^ Mulliken Center for Theoretical Chemistry, Clausius Institut für Physikalische und Theoretische Chemie Rheinische Friedrich-Wilhelms-Universität Bonn Beringstrasse 4 53115 Bonn Germany

**Keywords:** Bond Cleavage, Density Functional Theory, Diazirine, Frustrated Lewis Pairs, Mechanistic Study

## Abstract

The reactions of FLPs with diazomethanes leads to the rapid loss of N_2_. In contrast, in this work, we reported reactions of phosphine/borane FLPs with chlorodiazirines which led to the reduction of the N=N double bond, affording linked phosphinimide/amidoborate zwitterions of the general form R_3_PNC(Ar)NR′BX(C_6_F_5_)_2_. A detailed DFT mechanistic study showed that these reactions proceed via FLP addition to the N=N bond, followed by subsequent group transfer reactions to nitrogen and capture of the halide anion.

## Graphical abstract

While the reactions of FLPs with diazomethanes are known to lead to rapid loss of N_2_, reactions of phosphine/borane FLPs with chlorodiazirines prompt N=N double bond cleavage, affording linked phosphinimide/amidoborate zwitterions of the general form R_3_PNC(Ar)NR′BX(C_6_F_5_)_2_. A detailed DFT mechanistic study shows that these reactions proceed via FLP addition to the N=N bond.

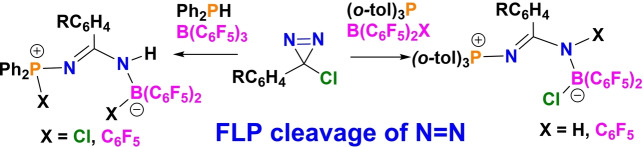

Since the discovery in 2006 of ability of frustrated Lewis pairs (FLPs) to activate dihydrogen^[1]^ and subsequently other small molecules,^[2]^ there has been speculation regarding the use of FLPs in N_2_ reduction. Nonetheless, this notion received little attention as main group‐N_2_ interactions were all but unknown. Nonetheless, in 2017, exploiting the ability of transition metal species to capture N_2_, Szymczak,^[3]^ Simonneau,^[4]^ and subsequently Sivasankar^[5]^ used complexes of the form (R_2_PCH_2_CH_2_PR_2_)_2_MN_2_ (M=Fe, Mo, Cr, W) in combination with B(C_6_F_5_)_3_ to activate the metal‐bound N_2_ fragment for borylation and silylation. More recently, Liddle and co‐workers^[6]^ targeted the reduction of N_2_ to NH_3_. This was achieved using a Ti_2_Mg_2_‐nitride species, [N(CH_2_CH_2_N(SiMe_3_))_3_MgNTi]_2_, in the presence of the FLP *t‐*Bu_3_P/B(C_6_F_5_)_3_ and H_2_. Similarly, Liddle et al^[7]^ have also shown that the uranyl‐nitride and boranes react with H_2_ ultimately liberating NH_3_.

In probing related main group chemistry, in 2012, we explored reactions of boranes with diazomethanes, demonstrating carbene insertion into B−C bonds (Scheme 1).^[8]^ However, in 2017, we isolated the unstable Ph_2_CN_2_B(C_6_F_5_)_3_ ^[9]^ which was subsequently stabilized by single electron reduction, leading to radical‐based intra‐ or intermolecular C−H bond activation.^[10]^ In parallel studies, we and others also showed that diazoesters^[11]^ and diazonium cations^[12]^ reacted with phosphine donors, affording adducts of the form EtOC(=O)CHNN(PR_3_), [ArN(PPh_3_)N(PPh_3_)]^+^ and [ArN_2_(PR_3_)]^+^, respectively. Subsequently, related chemistry of boranes and diazoesters has been exploited in organic synthesis by Melen and co‐workers.^[13]^ In addition, Melen and co‐workers have reviewed borane mediated carbene transfer reactions using diazomethanes,^[14]^ while more recently carbocations derived from protonation of diazomethane α‐carbon atoms were shown to be key intermediate in such reactions.^[15]^

**Scheme 1:**
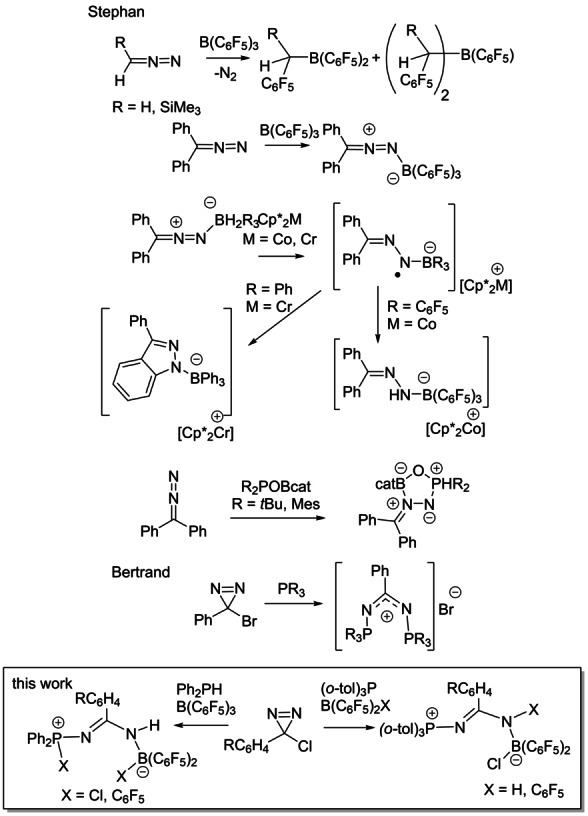
Addition reactions of diazomethanes with main group species.

In 2018, the seminal report by Braunschweig et al.^[16]^ demonstrated N_2_ reduction by a B^I^ species. To us, this further supported the notion that similar reactivity might also be achieved with a B^III^ species and a two‐electron donor (i.e., a FLP). Probing this related reactions of the intramolecular FLPs R_2_POBcat with diazomethanes, we found stable, isolable diazo‐heterocyclic species Ph_2_CN_2_(R_2_POBcat) and EtO_2_CCHN_2_(R_2_POBcat)_2_ in which both P and B centers are added to the N−N fragment.^[17]^

A closely related class of compounds to diazomethanes is diazirines. While reactivity with transition metal species has been explored,^[18]^ main group chemistry of diazirines has drawn lesser attention. In 1996, Bertrand et al. showed that phosphines react with bromodiazirines afforded cationic N,N′‐bis(phosphine) adducts via a proposed nitrene intermediate.^[19]^ In 2016, Wu et al^[20]^ exploited an oxidant to develop a metal‐free avenue to the cross‐coupling of boronic acids and diazirines. Again, these reactions proceed with loss of N_2_. In 2020, Lopchuk et al^[21]^ described the use of diazirines in the decarboxylative amination of redox‐active esters. Despite these reports, diazirines are described as “challenging to activate” by Arnold et al.^[22]^ in a recent report in which an engineered enzyme variant was shown to effect selective carbene transfer from diazirines. In this report, we describe the facile reactions of FLPs with the diazirines of the form ArC(Cl)N_2_. In contrast to all previous reactions of FLPs with diazo species as well as the previously mentioned biocatalytic protocol, these reactions result in the cleavage of the N=N bond (Scheme 1). The mechanism of these reactions is illuminated via DFT calculations and the implication of these findings for the potential application in N_2_ cleavage is considered.

The FLP derived from (*o*‐tol)_3_P and B(C_6_F_5_)_3_ was dissolved in CH_2_Cl_2_ and a solution of the diazirine (BrC_6_H_4_)CClN_2_ in CH_2_Cl_2_ was added. The mixture was allowed to stir at room temperature for 24 h. Following removal of the solvent, the product **1** was isolated in 96 % yield. Compound **1** exhibited ^31^P and ^11^B NMR chemical shifts at 26.3 and −1.2 ppm, consistent with P^V^ and four‐coordinate boron centers, respectively. The ^19^F NMR spectrum showed resonances attributable to two sets of C_6_F_5_ groups in a 2 : 1 ratio. The more intense signals observed at −137.2, −159.8 and −165.5 ppm were attributable to C_6_F_5_ rings on a four‐coordinate boron center. The signals at −143.7, −155.2 and −162.6 ppm suggested the migration of one C_6_F_5_ ring from B to nitrogen center, further inferring the coordination of chloride anion to boron. Recrystallization of **1** from CH_2_Cl_2_/pentane afforded X‐ray quality crystals. A crystallographic study^[23]^ of **1** confirmed the formulation as (*o*‐tol)_3_PNC(C_6_H_4_Br)N(C_6_F_5_)B(C_6_F_5_)_2_Cl (Scheme 2, Figure 1). The molecules in the asymmetric unit affirmed the cleavage of the N=N bond and the formation of P−N and B−N bonds which average 1.619(2) Å and 1.572(3) Å, respectively. The corresponding N−C bonds to the central carbon atom were found to average 1.356(3) Å and 1.307(3) Å with the latter being the N−C towards phosphorus. The newly formed N−C_C6F5_ and B−Cl bonds average 1.428(3) Å band 1.920(3) Å, respectively. These metric parameters are consistent with a C=N double bond adjacent to the phosphorus atom with a trigonal planar geometry at the central carbon of the diazirine.

**Scheme 2:**
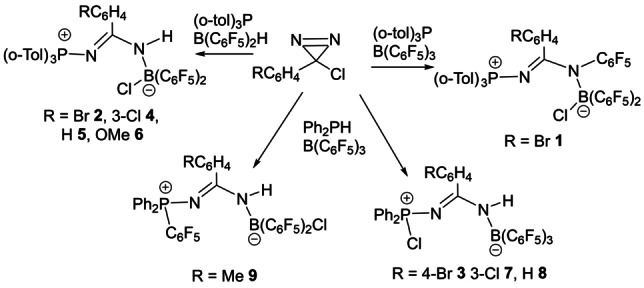
Synthesis of **1**–**9**.

**Figure 1:**
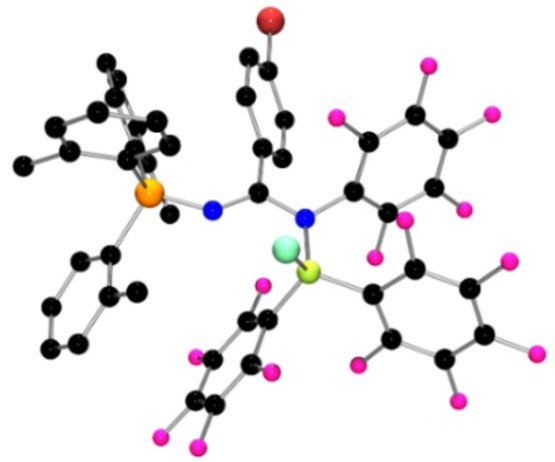
POV‐ray depiction of the molecular structure of **1**. Hydrogen atoms are omitted for clarity. C: black, F: hot pink, P: orange, N: blue, Cl: aquamarine, Br: red‐brown, B: yellow‐green.

The corresponding reaction of the Lewis pair (*o*‐tol)_3_P and dimeric borane [BH(C_6_F_5_)_2_]_2_ was performed in a similar fashion. As the classic Lewis acid‐base adduct of this pair proved insoluble, the solution was warmed to 40 °C for 24 h following the addition of the diazirine (BrC_6_H_4_)CClN_2_. Removal of the solvent afforded the product **2** in 92 % yield. This species exhibited ^31^P and ^11^B NMR chemical shifts at 23.3 and −3.6 ppm, respectively, while the ^19^F NMR spectrum showed resonances at −133.3, −160.4 and −165.6. These data suggested the migration of hydrogen atom from boron to nitrogen. This was confirmed via a crystallographic study^[23]^ revealing the formulation of **2** as (*o*‐tol)_3_PNC(C_6_H_4_Br)NH)B(C_6_F_5_)_2_Cl (Scheme 2, Figure 2). The metric parameters of **2** were similar to those in **1**, with the P−N and B−N bond lengths of 1.614(2) Å and 1.546(3) Å, respectively. Interestingly, the C−N bonds to the central carbon are 1.321(3) Å and 1.323(3) Å, suggesting a delocalized π‐system over the CN_2_ fragment.

**Figure 2:**
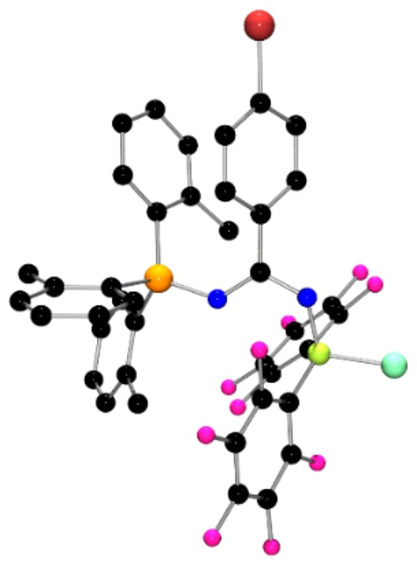
POV‐ray depiction of the molecular structure of **2**. Hydrogen atoms are omitted for clarity. C: black, F: hot pink, P: orange, N: blue, Cl: aquamarine, Br: red‐brown, B: yellow‐green.

As the third example, the reaction of (BrC_6_H_4_)CClN_2_ with the Lewis pair of Ph_2_PH and B(C_6_F_5_)_3_ was also performed at 40 °C for 24 h following addition of the diazirine (BrC_6_H_4_)CClN_2_. Removal of the solvent afforded the product **3** in 84 % yield. This species exhibited ^31^P and ^11^B NMR chemical shifts at 28.7 and −10.5 ppm, respectively, while the ^19^F NMR spectrum showed resonances at −134.4, −160.4 and −165.4 ppm. These data are again consistent with four‐coordinated P and B centers and the formulation of **3** as Ph_2_(Cl)PNC(C_6_H_4_Br)NHB(C_6_F_5_)_3_ (Scheme 2, Figure 3). This was also confirmed crystallographically.^[23]^ In this case, proton migration from P to N is apparent with quaternization at phosphorus resulting from the addition of chloride anion. The resulting P−N and P−Cl bond lengths are 1.560(3) Å and 2.0254(12) Å, respectively, while the N−B bond length is 1.563(4) Å. The shorter P−N bond and longer B−N bond in comparison to **1** are consistent with the substitutions on P, N and B respectively. The C−N bonds to the central carbon are bb1.315(4) Å and 1.323(4) Å, similar to those seen in **2**.

**Figure 3:**
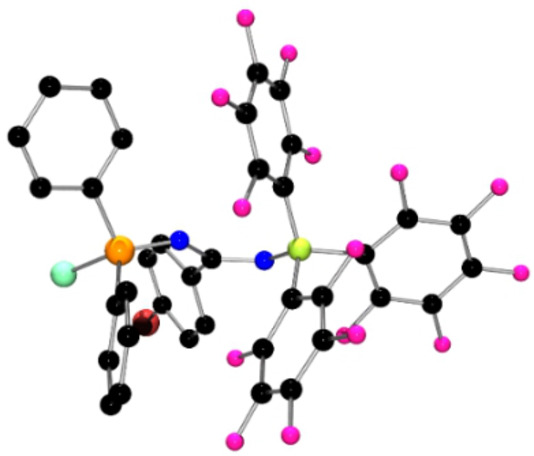
POV‐ray depiction of the molecular structure of **3**. Hydrogen atoms are omitted for clarity. C: black, F: hot pink, P: orange, N: blue, Cl: aquamarine, Br: red‐brown, B: yellow‐green.

The impact of perturbations to the diazirine was probed to some extent. For example, the reactions of (RC_6_H_4_)CClN_2_ with either (*o*‐tol)_3_P and BH(C_6_F_5_)_2_ or Ph_2_PH and B(C_6_F_5_)_3_ gave the products (*o*‐tol)_3_PNC(C_6_H_4_R)NHB(C_6_F_5_)_2_Cl (R=3‐Cl **4**, H **5**, OMe **6**) and Ph_2_(Cl)PNC(C_6_H_4_R)NHB(C_6_F_5_)_3_ (R=3‐Cl **7**, H **8**), respectively (Scheme 2). Interestingly, for R=Me, the reaction with Ph_2_PH and B(C_6_F_5_)_3_ afforded the product Ph_2_(C_6_F_5_)PNC(C_6_H_4_Me)NHB(C_6_F_5_)_2_Cl **9** as confirmed crystallographically^[23]^ (Scheme 2, Figure 4). The differing formulations of **3** and **9** each derived from the reaction of a diazirine and Ph_2_PH and B(C_6_F_5_)_3_ appear to arise from the electronic nature of the diazirine substituent. It should be noted that both **3** and **9** did not isomerize via Cl/C_6_F_5_ exchange reactions upon heating. However, reaction of compound **2** with the silane reagents Et_3_SiH, Me_3_SiCN, and Me_3_SiSCN gave the products (o‐tol)_3_PNC(C_6_H_4_Br)NH)B(C_6_F_5_)_2_X (X=H **10**, CN **11**, SCN **12**), respectively (Scheme 3), as characterized crystallographically (See Supporting Information).

**Figure 4:**
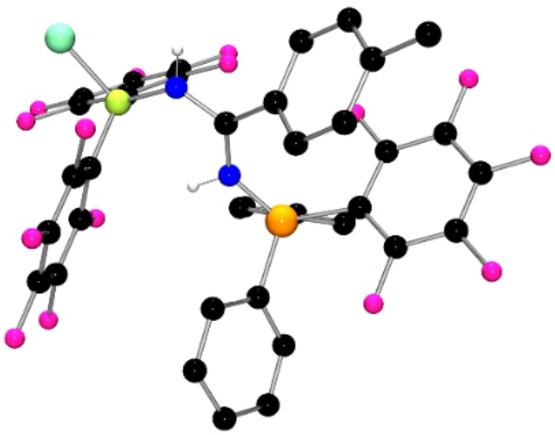
POV‐ray depiction of the molecular structure of **9**. Hydrogen atoms are omitted for clarity. C: black, F: hot pink, P: orange, N: blue, Cl: aquamarine, Br: red‐brown, B: yellow‐green.

**Scheme 3:**
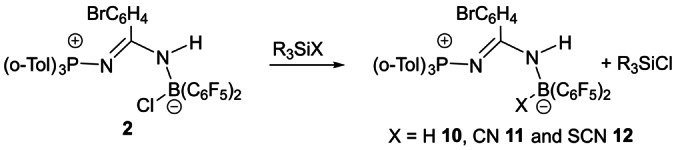
Synthesis of **10**–**12**.

To probe the mechanism affording the observed products, a DFT computational study was undertaken at the PW6B95‐D3/def2‐QZVP+COSMO‐RS//TPSS‐D3/def2‐TZVP+COSMO level in CH_2_Cl_2_ solution (Figure 5, see Supporting Information).^[24]^ The final Gibbs free energies (in kcal mol^−1^, at 298 K and 1 M concentration) are used in our discussion. The coordination of the borane B(C_6_F_5_)_3_ to the diazirine (BrC_6_H_4_)CClN_2_ prompted the further addition of the phosphine (*o*‐tol)_3_P, resulting in the formation of adduct **B**, (*o*‐tol)_3_PNC(BrC_6_H_4_)(Cl)NB(C_6_F_5_)_3_ in an exothermic process over a low barrier of 10.7 kcal mol^−1^ (via **TS1**). This reaction amounts to the FLP‐addition of the phosphine and borane to the N=N double bond and is directly analogous to the original FLP reactions described for combinations of phosphines, boranes and olefins.^[25]^ In addition, such FLP additions to diethylazo‐dicarboxylate has been previously described by the groups of Bourissou^[26]^ and Shaver.^[27]^ It is also noteworthy that this stands in contrast to reaction of the diazirine with phosphine alone that proceeds via an electrophilic nitrene intermediate as shown by Bertrand and co‐workers.^[19]^ However, in the present case, the intermediate **B** undergoes a further reaction in which additional borane also abstracts the chloride from the diazirine carbon over a sizable barrier of 23.7 kcal mol^−1^ (via **TS2**) to generate the transient cation **C^+^** [(*o*‐tol)_3_PNC(BrC_6_H_4_)NB(C_6_F_5_)_3_]^+^. This is followed by a rapid 1,2‐migration of a C_6_F_5_ fragment from boron to nitrogen (via **TS3^+^** to form **D^+^**) and slower chloride transfer from the borate anion ClB(C_6_F_5_)_3_^−^ to the boron center of **D^+^** over a moderate barrier of 19.8 kcal mol^−1^ (via **TS4**) affording the product **1**. The borane‐catalyzed reaction is thus rate‐limited by the chloride abstraction step over a barrier of 23.7 kcal mol^−1^.

**Figure 5:**
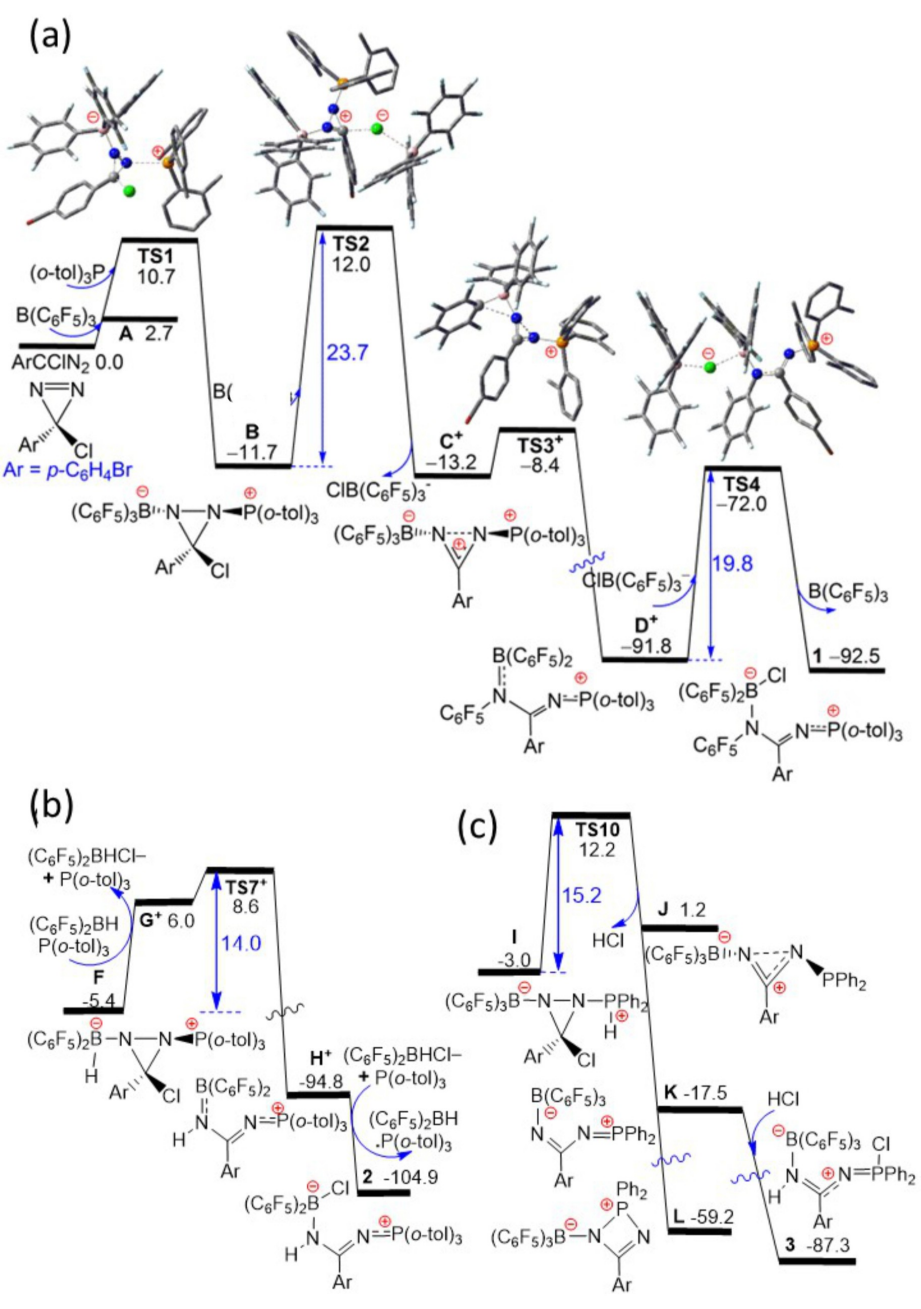
DFT computed Gibbs free energy paths (in kcal mol^−1^, at 298 K and 1 M concentration in CH_2_Cl_2_ solution) for the formation of a) **1**, b) **2** and c) **3**.

Computation probing the related pathways to **2** and **3** reveals that both reactions are initiated via the analogous addition of phosphine and borane to the N=N double bond (see Supporting Information Figures S1 and S2), but now mediated by the stable Lewis pair adducts of (C_6_F_5_)_2_BH⋅P(*o*‐tol)_3_ and (C_6_F_5_)_3_B⋅PHPh_2_ to form the respective adduct **F** and **I**. In the case of the reaction of (C_6_F_5_)_2_BH, (*o*‐tol)_3_P and the diazirine (BrC_6_H_4_)CClN_2_, chloride abstraction from **F** with (C_6_F_5_)_2_BH⋅P(*o*‐tol)_3_ followed by rapid 1,2‐H‐migration from boron to nitrogen within the resultant cation **G^+^** analogous to **D^+^** (via **TS7^+^** to form **H^+^**, Figure 5) and facile chloride transfer from the borate anion [(C_6_F_5_)_2_BHCl]^−^ to the boron center of **H^+^** eventually affords the product **2** in a highly exergonic step. In contrast, in the case of the reaction of B(C_6_F_5_)_3_, PPh_2_H and the diazirine (BrC_6_H_4_)CClN_2_, following the FLP addition to the N=N double bond, loss of HCl from the P/C fragments of adduct **I** encounters a low barrier of 15.2 kcal mol^−1^ (via **TS10**) to generate a transient Lewis acidic P center in intermediate **K**. Recapture of HCl by intermediate **K** affords **3** in a highly exergonic step. It is also noteworthy that this intermediate could also account for the migration of C_6_F_5_ from boron to phosphorus as seen in **9**. Presumably the nature of the substituents on the diazirine influences both the Lewis acidity of the phosphorus cation and the nucleophilicity of the borate fragment.

In conclusion, this work reported the FLP addition to chlorodiazirines. In contrast to diazomethanes where N_2_ is liberated, these reactions lead to the reduction of the diazirine N=N double bond as an activated form of N_2_, affording linked phosphinimide/amidoborate zwitterions of the form R_3_PNC(Ar)NR′BX(C_6_F_5_)_2_. This finding may promote further efforts to develop metal‐free avenues to dinitrogen chemistry. We are continuing to study related systems that could further this prospect.

Supplementary data including synthetic and spectral data, DFT‐computed energies and optimized Cartesian coordinates are deposited. Crystallographic data are deposited in CCDC.

## Conflict of interest

The authors declare no conflict of interest.

## Supporting information

As a service to our authors and readers, this journal provides supporting information supplied by the authors. Such materials are peer reviewed and may be re‐organized for online delivery, but are not copy‐edited or typeset. Technical support issues arising from supporting information (other than missing files) should be addressed to the authors.

Supporting Information

Supporting Information

Supporting Information

Supporting Information

Supporting Information

## Data Availability

The data that support the findings of this study are available in the Supporting Information of this article.
