# Supplementary material for: Reactions of Frustrated Lewis Pairs with Chloro‐Diazirines: Cleavage of N=N Double Bonds
Source: Angew Chem Int Ed Engl. 2022 Aug 3;61(37):e202209241. doi: 10.1002/anie.202209241 (PMC9543150; doi:10.1002/anie.202209241)
Supplement: Supplementary file 5 — Supporting Information [file ANIE-61-0-s004.pdf]

Angewandte Chemie
Eine Zeitschrift der Gesellschaft Deutscher Chemiker

Supporting Information

Reactions of Frustrated Lewis Pairs with Chloro-Diazirines: Cleavage of N=N Double Bonds

D. Mandal, T. Chen, Z.-W. Qu, S. Grimme, D. W. Stephan**

<u>Electronic Supporting Information</u>

Table of Contents

General information for synthesis 4
Synthetic procedures and characterization data 5
Synthesis of 1 5
Synthesis of 2 6
Synthesis of 3 6
Synthesis of 4 7
Synthesis of 5 8
Synthesis of 6 8
Synthesis of 7 9
Synthesis of 8 10
Synthesis of 9 10
Synthesis of 10 11
Synthesis of 11 12
Synthesis of 12 13
NMR spectra of all the compounds 14
Compound 1 14
Figure S1. ^{1}H NMR (500 MHz) spectrum of the compound 1 in CD_2Cl_2 (*= CD_2Cl_2). 14
Figure S2. ^{31}P NMR (203 MHz) spectrum of the compound 1 in CD_2Cl_2. 14
Figure S3. ^{19}F NMR (471 MHz) spectrum of the compound 1 in CD_2Cl_2. 15
Figure S4. ^{11}B NMR (161 MHz) spectrum of the compound 1 in CD_2Cl_2. 15
Figure S5. ^{13}C NMR (126 MHz) spectrum of the compound 1 in CD_2Cl_2 (*= CD_2Cl_2). 16
Compound 2 17
Figure S6. ^{1}H NMR (500 MHz) spectrum of the compound 2 in CD_2Cl_2 (*= CD_2Cl_2). 17
Figure S7. ^{31}P NMR (203 MHz) spectrum of the compound 2 in CD_2Cl_2. 17
Figure S8. ^{19}F NMR (471 MHz) spectrum of the compound 2 in CD_2Cl_2. 18

Figure S9. ^{11}B NMR (161 MHz) spectrum of the compound 2 in CD_2Cl_2. 18
Figure S10. ^{13}C NMR (126 MHz) spectrum of the compound 2 in CD_2Cl_2 (*= CD_2Cl_2, %= $CDCl_3$). 19
Compound 3 20
Figure S11. ^{1}H NMR (500 MHz) spectrum of the compound 3 in CD_2Cl_2 (*= CD_2Cl_2). 20
Figure S12. ^{31}P NMR (203 MHz) spectrum of the compound 3 in CD_2Cl_2. 20
Figure S13. ^{19}F NMR (471 MHz) spectrum of the compound 3 in CD_2Cl_2. 21
Figure S14. ^{11}B NMR (161 MHz) spectrum of the compound 3 in CD_2Cl_2. 21
Figure S15. ^{13}C NMR (126 MHz) spectrum of the compound 3 in CD_2Cl_2 (*= CD_2Cl_2). 22
Compound 4 23
Figure S16. ^{1}H NMR (500 MHz) spectrum of the compound 4 in $CH_2Cl_2/CDCl_3$ (5:1) (*= CH_2Cl_2). 23
Figure S17. ^{31}P NMR (203 MHz) spectrum of the compound 4 in $CH_2Cl_2/CDCl_3$ (5:1). 23
Figure S18. ^{19}F NMR (471 MHz) spectrum of the compound 4 in $CH_2Cl_2/CDCl_3$ (5:1). 24
Figure S19. ^{11}B NMR (161 MHz) spectrum of the compound 4 in $CH_2Cl_2/CDCl_3$ (5:1). 24
Figure S20. ^{13}C NMR (126 MHz) spectrum of the compound 4 in CD_2Cl_2 (*= CH_2Cl_2). 25
Compound 5 26
Figure S21. ^{1}H NMR (500 MHz) spectrum of the compound 5 in $CH_2Cl_2/CDCl_3$ (5:1) (*= CH_2Cl_2). 26
Figure S22. ^{31}P NMR (203 MHz) spectrum of the compound 5 in $CH_2Cl_2:CDCl_3$ (5:1). 26
Figure S23. ^{19}F NMR (471 MHz) spectrum of the compound 5 in $CH_2Cl_2:CDCl_3$ (5:1). 27
Figure S24. ^{11}B NMR (161 MHz) spectrum of the compound 5 in $CH_2Cl_2:CDCl_3$ (5:1). 27
Figure S25. ^{13}C NMR (126 MHz) spectrum of the compound 5 in $CH_2Cl_2:CDCl_3$ (5:1) (*= CH_2Cl_2). 28
Compound 6 29
Figure S26. ^{1}H NMR (500 MHz) spectrum of the compound 6 in CD_2Cl_2 (*= CD_2Cl_2). 29
Figure S27. ^{31}P NMR (203 MHz) spectrum of the compound 6 in CD_2Cl_2. 29
Figure S28. ^{19}F NMR (471 MHz) spectrum of the compound 6 in CD_2Cl_2. 30
Figure S29. ^{11}B NMR (161 MHz) spectrum of the compound 6 in CD_2Cl_2. 30
Figure S30. ^{13}C NMR (126 MHz) spectrum of the compound 6 in CD_2Cl_2 (*= CD_2Cl_2). 31
Compound 7 32
Figure S31. ^{1}H NMR (500 MHz) spectrum of the compound 7 in CD_2Cl_2 (*= CD_2Cl_2). 32
Figure S32. ^{31}P NMR (203 MHz) spectrum of the compound 7 in CD_2Cl_2. 32
Figure S33. ^{19}F NMR (471 MHz) spectrum of the compound 7 in CD_2Cl_2. 33
Figure S34. ^{11}B NMR (161 MHz) spectrum of the compound 7 in CD_2Cl_2. 33
Figure S35. ^{13}C NMR (126 MHz) spectrum of the compound 7 in CD_2Cl_2 (*= CD_2Cl_2). 34
Compound 8 35
Figure S36. ^{1}H NMR (500 MHz) spectrum of the compound 8 in CD_2Cl_2 (*= CD_2Cl_2). 35
Figure S37. ^{31}P NMR (203 MHz) spectrum of the compound 8 in CD_2Cl_2. 35
Figure S38. ^{19}F NMR (471 MHz) spectrum of the compound 8 in CD_2Cl_2. 36
Figure S39. ^{11}B NMR (161 MHz) spectrum of the compound 8 in CD_2Cl_2. 36
Figure S40. ^{13}C NMR (126 MHz) spectrum of the compound 8 in CD_2Cl_2 (*= CD_2Cl_2). 37
Compound 9 38
Figure S41. ^{1}H NMR (500 MHz) spectrum of the compound 9 in CD_2Cl_2 (*= CD_2Cl_2). 38
Figure S42. ^{31}P NMR (203 MHz) spectrum of the compound 9 in CD_2Cl_2. 38
Figure S43. ^{19}F NMR (471 MHz) spectrum of the compound 9 in CD_2Cl_2. 39

Figure S44. ^{11}B NMR (161 MHz) spectrum of the compound 9 in CD_2Cl_2. 39
Figure S45. ^{13}C NMR (126 MHz) spectrum of the compound 9 in CD_2Cl_2 (*= CD_2Cl_2). 40
Compound 10 41
Figure S46. ^{1}H NMR (500 MHz) spectrum of the compound 10 in CD_2Cl_2 (*= CD_2Cl_2). 41
Figure S47. ^{31}P NMR (203 MHz) spectrum of the compound 10 in CD_2Cl_2. 41
Figure S48. ^{19}F NMR (471 MHz) spectrum of the compound 10 in CD_2Cl_2. 42
Compound 11 43
Figure S49. ^{1}H NMR (500 MHz) spectrum of the compound 11 in CD_2Cl_2 (*= CD_2Cl_2). 43
Figure S50. ^{31}P NMR (203 MHz) spectrum of the compound 11 in CD_2Cl_2. 43
Figure S51. ^{19}F NMR (471 MHz) spectrum of the compound 11 in CD_2Cl_2. 44
Figure S52. ^{11}B NMR (161 MHz) spectrum of the compound 11 in CD_2Cl_2. 44
Figure S53. ^{13}C NMR (126 MHz) spectrum of the compound 11 in CD_2Cl_2 (*= CD_2Cl_2). 45
Compound 12 46
Figure S54. ^{1}H NMR (500 MHz) spectrum of the compound 12 in CD_2Cl_2 (*= CD_2Cl_2). 46
Figure S55. ^{31}P NMR (203 MHz) spectrum of the compound 12 in CD_2Cl_2. 46
Figure S56. ^{19}F NMR (471 MHz) spectrum of the compound 12 in CD_2Cl_2. 47
Figure S57. ^{11}B NMR (161 MHz) spectrum of the compound 12 in CD_2Cl_2. 47
Figure S58. ^{13}C NMR (126 MHz) spectrum of the compound 12 in CD_2Cl_2 (*= CD_2Cl_2). 48

Control reactions **49**

3-(4-Bromophenyl)-3-chloro-diazirine and $B(C_6F_5)_3$ 49
3-(4-Bromophenyl)-3-chloro-diazirine and $HB(C_6F_5)_2$ 49
3-(4-Bromophenyl)-3-chloro-diazirine and P(o-Tol)$_3$ 49
3-(4-Bromophenyl)-3-chloro-diazirine and HP(Ph)$_2$ 49

Figures for control reactions **50**

Figure S59. Image for control reactions after 24 h at RT. 50
Figure S60.^{19}F NMR (471 MHz) spectrum of the crude mixture of 3-(4-bromophenyl)-3-chloro-diazirine and $B(C_6F_5)_3$ after 24 h at RT. 51
Figure S61.^{19}F NMR (471 MHz) spectrum of the crudemixture of 3-(4-bromophenyl)-3-chloro-diazirine and $HB(C_6F_5)_2$ after 24 h at RT. 51
Figure S62.^{31}P NMR (203 MHz) spectrum of the crude mixture of 3-(4-bromophenyl)-3-chloro-diazirine and P(*o*-Tol)$_3$ after 24 h at RT. 52
Figure S63.^{31}P NMR (203 MHz) spectrum of the crude mixture of 3-(4-bromophenyl)-3-chloro-diazirine and HP(Ph)$_2$ after 24 h at RT. 52

Experimental references **53**

Computational Details **54**

Figure S64. DFT computed complete Gibbs free energy paths (in kcal/mol, at 298 K and 1 M concentration in CH_2Cl_2 solution) for the formation of 2. 56
Figure S65. DFT computed complete Gibbs free energy paths (in kcal/mol, at 298 K and 1 M concentration in CH_2Cl_2 solution) for the formation of 3. 57

Computational references **92**

Experimental

General information for synthesis

Experiments were carried under inert conditions using standard Schlenk techniques or a glove box as appropriate. Dichloromethane (DCM, CH_2Cl_2) and *n*-hexanes (C_6H_{14}) were dispensed from an MBRAUN Solvent Purification System, deoxygenated by bubbling Ar for 20 min, and stored over 3 Å molecular sieves prior to use whilst 1,2-dichloroethane (1,2-DCE, 1,2-$C_2H_4Cl_2$), was stirred over CaH_2 at room temperature under Ar overnight prior to distillation under reduced pressure and stored under 3 Å molecular sieves before use. Chloroform-d ($CDCl_3$) and dichloromethane-d_2 (CD_2Cl_2) solvents were used as received without any purification and those were stored over 4 Å molecular sieves prior to use. Vials and stir bar for reactions were oven-dried overnight before experiments. ^{1}H (500 MHz), ^{19}F (471 MHz), $^{19}F\{^{1}H\}$ (471 MHz), $^{31}P\{^{1}H\}$ (202 MHz), and $^{13}C\{^{1}H\}$ (126 MHz) NMR spectra were run at 298 K on Bruker 500 spectrometers. The chemical shifts (δ, ppm) for ^{1}H and $^{13}C\{^{1}H\}$ NMR spectra are given relative to solvent signals whereas an external reference standards used for $^{31}P\{^{1}H\}$ (85% H_3PO_4), ^{19}F ($CFCl_3$) and $^{19}F\{^{1}H\}$ ($CFCl_3$) NMR spectra. These NMR data are written as: chemical shift, multiplicity (s = singlet, d = doublet, t = triplet, q = quartet, m = multiplet, br = broad), coupling constants (Hz) and integration. The single-crystal *X*-ray data were collected on a Bruker D8 QUEST diffractometer using Cu (60W, Diamond, μKα = 12.894 mm^{-1}) micro-focus *X*-ray sources at 150 K. The structure was solved and refined using Full-matrix least-squares based on F^2 with a suite of programs SHELXS and SHELXL[1] compiled in OLEX2.[2] The reagents $B(C_6F_5)_3$[3] and diazirines[4] were prepared by following literature method or a slight variations thereof. All other reagents were purchased commercially and used as received.

Synthetic procedures and characterization data

Synthesis of 1

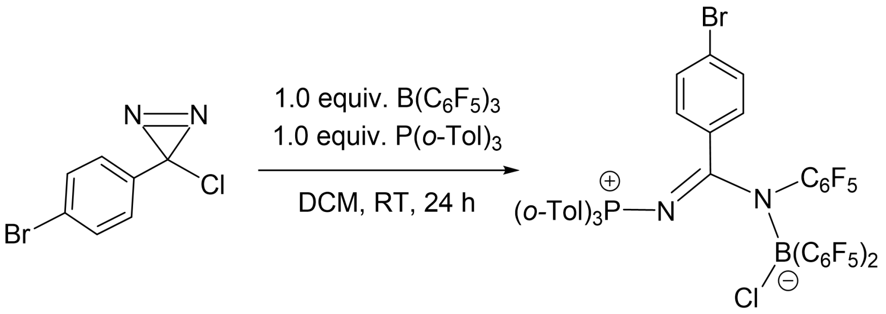

Into a 4 mL open top PTFE vial equipped with a stir bar, $B(C_6F_5)_3$ (51mg, 0.10 mmol, 1.0 equiv.) and P(*o*-Tol)$_3$ (30 mg, 0.1 mmol, 1.0 equiv.) were dissolved in DCM (0.5 mL). After addition of a solution of diazirine (23 mg, 0.1 mmol, 1.0 equiv.) in DCM (0.5 mL), the reaction mixture was allowed to stir at RT for 24 h. After removal of all volatiles, the residue was washed with *n*-hexane (3 x 1 mL). Further, the residue was dried in affording compound **1** (101 mg, 96%). *X*-ray quality crystals were grown with a mixture of solvent of DCM:*n*-hexane (1:5) and stored at -30 °C for two days. **1**: ^{1}H NMR (500 MHz, CD_2Cl_2): δ_H 7.57 (t, J = 7.1 Hz, 3 H, Ar-*H*), 7.54 – 7.32 (m, 6 H, Ar-*H*), 7.29 – 7.16 (m, 5 H, Ar-*H*), 7.07 (d, J = 7.9 Hz, 2 H, Ar-*H*), 1.89 (s, 9 H, -C*H*$_3$);^{31}P NMR (203 MHz, CD_2Cl_2): δ_P26.3 (s, 1 P, -*P*(*o*-Tol)$_3$);^{19}F NMR (471 MHz, CD_2Cl_2): δ_F -137.2 (m, 4 F, *o*-C_6F_5 of -BCl(C_6F_5)$_2$), -143.7 (m, 2 F, *o*-NC_6F_5), -155.2 (m, 1 F, *p*-NC_6F_5), -159.8 (m, 2 F, *p*-C_6F_5 of -BCl(C_6F_5)$_2$), -162.6 (m, 2 F, *m*-NC_6F_5), -165.5 (m, 4 F, *m*-C_6F_5 of -BCl(C_6F_5)$_2$); ^{11}B NMR (161 MHz, CD_2Cl_2): δ_B -1.2 (br s, 1 B, -*B*Cl(C_6F_5)$_2$); ^{13}C NMR (126 MHz, CD_2Cl_2): δ_C 169.1 (d, $^2J_{C\text{-}P}$ = 4.9 Hz, -P-N=*C*(Ar)-N-), 145.3 (br s, -C_6F_5), 143.7 (d, $^3J_{C\text{-}P}$ = 9.2 Hz, C_{Ar}), 143.3 (br s, -C_6F_5), 142.4 (br s, -C_6F_5), 139.5 (br s, -C_6F_5), 138.3 (br s, -C_6F_5), 137.5 (br s, -C_6F_5), 136.4 (br s, -C_6F_5), 135.0 (s, C_{Ar}), 134.8 (d, $^2J_{C\text{-}P}$ = 12.8 Hz, C_{Ar}), 134.3 (br s, -C_6F_5), 133.9 (d, $^3J_{C\text{-}P}$ = 12.0 Hz, C_{Ar}), 131.3 (s, C_{Ar}), 130.6 (s, C_{Ar}), 128.8 (d, $^4J_{C\text{-}P}$ = 4.5 Hz, C_{Ar}), 128.0 (s, C_{Ar}), 127.6 (d, $^2J_{C\text{-}P}$ = 14.1 Hz, C_{Ar}), 121.1 (d, $^1J_{C\text{-}P}$ = 98.2 Hz, C_{Ar}), 22.1 (s, *C*H$_3$ of P(*o*-Tol)$_3$).

Synthesis of 2

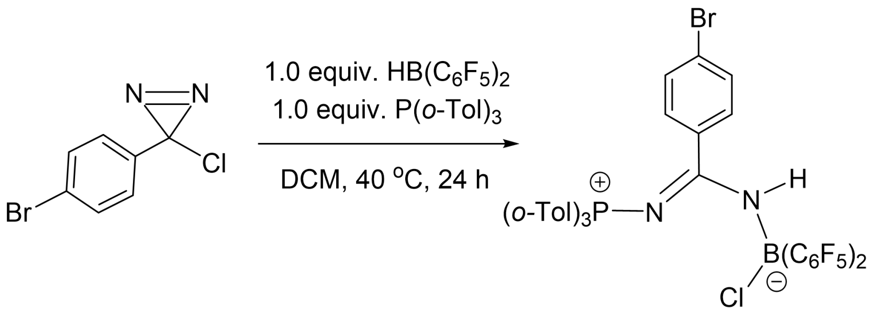

2 (81 mg, 92%) was prepared by following the protocol for **1** whilst the reaction vial was heated at 40 °C for 24 h. *X*-ray quality crystals were grown with a mixture of solvent of DCM:*n*-hexane (1:5) and stored at -30 °C for two days. **2**: ^{1}H NMR (500 MHz, CD_2Cl_2): δ_H10.11 (br s, 1 H, -N*H*), 8.20 –7.89 (m, 2 H, Ar-*H*), 7.66 – 7.46 (m, 3 H, Ar-*H*), 7.43 (d, *J* = 7.2 Hz, 2 H, Ar-*H*), 7.37 – 7.16 (m, 9 H, Ar-*H*), 1.89 (s, 9 H, -C*H*$_3$);^{31}P NMR (203 MHz, CD_2Cl_2): δ_P23.3 (s, 1 P, -*P*(*o*-Tol)$_3$);^{19}F NMR (471 MHz, CD_2Cl_2): δ_F -133.3 (m, 4 F, *o*-C_6F_5 of -BCl(C_6F_5)$_2$), -160.4 (m, 2 F, *p*-C_6F_5 of -BCl(C_6F_5)$_2$), -165.6 (m, 4 F, *m*-C_6F_5 of -BCl(C_6F_5)$_2$); ^{11}B NMR (161 MHz, CD_2Cl_2): δ_B -3.6 (br s, 1 B, -*B*Cl(C_6F_5)$_2$); ^{13}C NMR (126 MHz, CD_2Cl_2): δ_C 166.8 (d, $^2J_{C\text{-}P}$ = 9.4 Hz, -P-N=*C*(Ar)-N-), 145.7 (br s, -*C*$_6$F$_5$), 144.1 (d, $^3J_{C\text{-}P}$ = 8.4 Hz), 142.2 (br s, -*C*$_6$F$_5$), 138.6 (br s, -*C*$_6$F$_5$), 136.5 (br s, -*C*$_6$F$_5$), 135.5 (s, C_{Ar}), 134.5 (d, $^2J_{C\text{-}P}$ = 12.8 = 12.6 Hz, C_{Ar}), 134.4 (d, $^3J_{C\text{-}P}$ = 11.2 Hz, C_{Ar}), 132.7 (s, C_{Ar}), 132.1 ($^4J_{C\text{-}P}$ = 4.7 Hz, C_{Ar}), 131.5 (s, C_{Ar}), 129.2 (s, C_{Ar}), 128.3 (d, $^2J_{C\text{-}P}$ = 13.8 Hz, C_{Ar}), 121.9 (d, $^1J_{C\text{-}P}$ = 97.8 Hz, C_{Ar}), 22.7 (s, *C*H$_3$ of P(*o*-Tol)$_3$).

Synthesis of 3

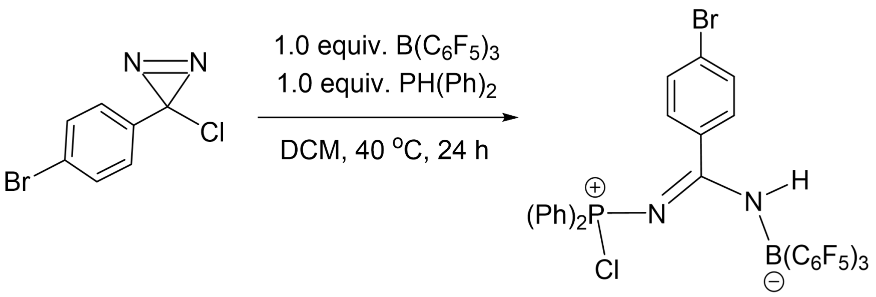

3 (78 mg, 84%)was prepared by following the protocol for **1** whilst the reaction vial was heated at 40 °C for 24 h. *X*-ray quality crystals were grown with a mixture of solvent of DCM:*n*-hexane (1:5) and stored at -30 °C for two days. **3**: ^{1}H NMR (500 MHz, CD_2Cl_2): δ_H

7.72 – 7.66 (m, 2 H, Ar-*H*), 7.54 (dt, *J* = 8.4, 2.1 Hz, 2 H, Ar-*H*), 7.50 – 7.43 (m, 4 H, Ar-*H*), 7.38 (br s, 1 H, -N*H*), 7.37 – 7.28 (m, 4 H, Ar-*H*), 7.23 (dt, *J* = 8.6, 2.2 Hz, 2 H, Ar-*H*);^{31}P NMR (203 MHz, CD_2Cl_2): δ_P28.7 (s, 1 P, -*P*(Ph)$_2$Cl);^{19}F NMR (471 MHz, CD_2Cl_2): δ_F -134.4 (m, 4 F, *o*-C_6F_5 of -B(C_6F_5)$_3$), -160.4 (m, 3 F, *p*-C_6F_5 of -B(C_6F_5)$_3$), -165.4 (m, 4 F, *m*-C_6F_5 of -B(C_6F_5)$_3$); ^{11}B NMR (161 MHz, CD_2Cl_2): δ_B-10.5 (br s, 1 B, -*B*(C_6F_5)$_3$); ^{13}C NMR (126 MHz, CD_2Cl_2): δ_C 169.8 (d, $^2J_{C\text{-}P}$ = 3.5 Hz, -P-N=*C*(Ar)-N-), 149.6 (br s, -*C*$_6$F$_5$), 147.7 (br s, -*C*$_6$F$_5$), 138.3 (br s, -*C*$_6$F$_5$), 136.4 (br s, -*C*$_6$F$_5$), 135.1 (d, $^4J_{C\text{-}P}$ = 3.6 Hz, C_{Ar}), 132.7 (s, C_{Ar}), 131.6 (d, $^3J_{C\text{-}P}$ = 11.8 Hz, C_{Ar}), 129.8 (d, $^2J_{C\text{-}P}$ = 15.4 Hz, C_{Ar}), 128.9 (s, C_{Ar}), 128.8 (s, C_{Ar}), 127.0 (d, $^1J_{C\text{-}P}$ = 181.4 Hz, C_{Ar}).

Synthesis of 4

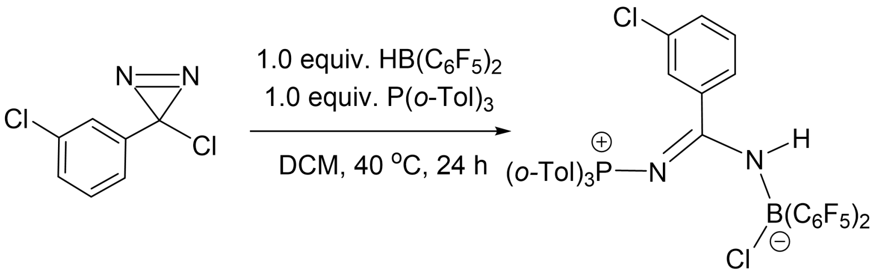

4 (70 mg, 79%) was prepared by following the protocol for **1** whilst the reaction vial was heated at 40 °C for 24 h. **4**: ^{1}H NMR (500 MHz, $CH_2Cl_2/CDCl_3$ (5:1)): δ_H 7.70 (tt, *J* = 7.6, 2.2 Hz, 1 H, Ar-*H*), 7.64 – 7.55 (m, 1 H, Ar-*H*), 7.53 – 7.39 (m, 5 H, Ar-*H*), 7.34 – 7.23 (m, 2 H, Ar-*H*), 7.20 (t, *J* = 6.6 Hz, 3 H, Ar-*H*), 7.16 (t, *J* = 8.2, 1.4 Hz, 3 H, Ar-*H*), 7.09 (t, *J* = 1.9 Hz, 1 H, Ar-*H*), 7.03 (ddd, *J* = 8.0, 2.0, 1.0 Hz, 1 H, Ar-*H*), 6.92 (t, *J* = 7.9 Hz, 1 H, Ar-*H*), 1.89 (s, 3 H, -C*H*$_3$); ^{31}P NMR (203 MHz, $CH_2Cl_2/CDCl_3$ (5:1)): δ_P 22.4 (s, 1 P, -*P*(*o*-Tol)$_3$);^{19}F NMR (471 MHz, $CH_2Cl_2/CDCl_3$ (5:1)): δ_F -133.3 (m, 4 F, *o*-C_6F_5 of -B(C_6F_5)$_3$), -160.4 (m, 3 F, *p*-C_6F_5 of -B(C_6F_5)$_3$), -165.6 (m, 4 F, *m*-C_6F_5 of -B(C_6F_5)$_3$); ^{11}B NMR (161 MHz, $CH_2Cl_2/CDCl_3$ (5:1)): δ_B -3.6 (br s, 1 B, -*B*(C_6F_5)$_2$Cl); ^{13}C NMR (126 MHz, CD_2Cl_2): δ_C 168.5 (d, $^2J_{C\text{-}P}$ = 4.4 Hz, -P-N=*C*(Ar)-N-), 149.6 (br s, -*C*$_6$F$_5$), 147.6 (br s, -*C*$_6$F$_5$), 143.5 (br s, -*C*$_6$F$_5$), 140.8 (br s, -*C*$_6$F$_5$), 138.2 (s), 135.4 (d, $^4J_{C\text{-}P}$ = 2.8 Hz, C_{Ar}), 134.4 (d, $^2J_{C\text{-}P}$ = 13.5 Hz, C_{Ar}), 133.8 (s, C_{Ar}), 133.3 (d, $^3J_{C\text{-}P}$ = 11.1 Hz, C_{Ar}), 134.2 (d, $^2J_{C\text{-}P}$ = 11.6 Hz, C_{Ar}), 131.1 (s, C_{Ar}), 130.4 (s, C_{Ar}), 128.1 (d,

$^{2}J_{C\text{-}P}$ = 13.6 Hz, C_{Ar}), 127.5 (s, C_{Ar}), 125.4 (s, C_{Ar}), 121.4 (d, $^{1}J_{C\text{-}P}$ = 102.2 Hz, C_{Ar}), 22.9 (s, CH_3 of P(*o*-Tol)$_3$).

Synthesis of 5

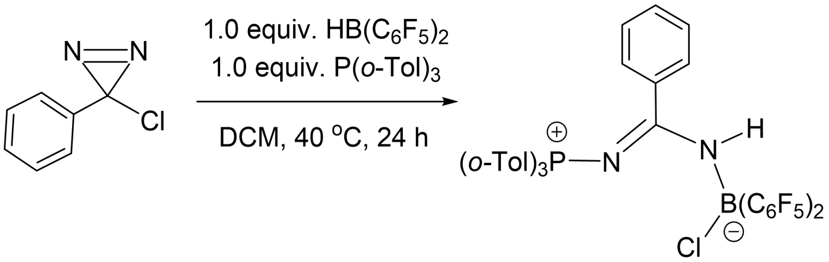

5 (63 mg, 78%) was prepared by following the protocol for **1** whilst the reaction vial was heated at 40 °C for 24 h. **5**: ^{1}H NMR (500 MHz, CH_2Cl_2/$CDCl_3$ (5:1)): δ_H 7.67 (tt, *J* = 7.5, 1.6 Hz, 1 H, Ar-*H*), 7.60 (tt, *J* = 7.6, 1.5 Hz, 1 H, Ar-*H*), 7.52 – 7.39 (m, 4 H, Ar-*H*), 7.38 – 7.32 (m, 2 H, Ar-*H*), 7.33 – 7.23 (m, 2 H, Ar-*H* and -N*H*), 7.21 (dt, *J* = 8.5, 1.1 Hz, 3 H, Ar-*H*), 7.18 – 7.12 (m, 2 H, Ar-*H*), 7.06 (tt, *J* = 7.5, 1.2 Hz, 1 H, Ar-*H*), 6.91 (tt, *J* = 7.6, 1.7 Hz, 2 H, Ar-*H*), 1.89 (s, -C*H*$_3$); ^{31}P NMR (203 MHz, CH_2Cl_2/$CDCl_3$ (5:1)): δ_P 21.4 (s, 1 P, -*P*(*o*-Tol)$_3$); ^{19}F NMR (471 MHz, CH_2Cl_2/$CDCl_3$ (5:1)): δ_F -133.0 (m, 4 F, *o*-C_6F_5 of -BCl(C_6F_5)$_2$), -160.0 (m, 2 F, *p*-C_6F_5 of -BCl(C_6F_5)$_2$), -165.1 (m, 4 F, *m*-C_6F_5 of -BCl(C_6F_5)$_2$); ^{11}B NMR (161 MHz, CH_2Cl_2/$CDCl_3$ (5:1)): δ_B -3.6 (br s, 1 B, -*B*Cl(C_6F_5)$_2$); ^{13}C NMR (126 MHz, CH_2Cl_2/$CDCl_3$ (5:1)): δ_C 169.3 (d, $^{2}J_{C\text{-}P}$ = 4.1 Hz, -P-N=*C*(Ar)-N-), 150.8 (br s, -C_6F_5), 148.8 (br s, -C_6F_5), 139.9 (br s, -C_6F_5), 137.7 (br s, -C_6F_5), 134.6 (d, $^{4}J_{C\text{-}P}$ = 3.4 Hz, C_{Ar}), 133.7 (d, $^{3}J_{C\text{-}P}$ = 13.6 Hz, C_{Ar}), 133.5 (d, $^{2}J_{C\text{-}P}$ = 11.1 Hz, C_{Ar}), 132.6 (d, $^{2}J_{C\text{-}P}$ = 11.1 Hz, C_{Ar}), 130.4 (s, C_{Ar}), 128.7 (s, C_{Ar}), 127.9 (s, C_{Ar}), 127.4 (d, $^{3}J_{C\text{-}P}$ = 12.7 Hz, C_{Ar}), 126.5 (s, C_{Ar}), 121.1 (d, $^{1}J_{C\text{-}P}$ = 97.2 Hz, C_{Ar}), 22.6 (s, CH_3 of P(*o*-Tol)$_3$).

Synthesis of 6

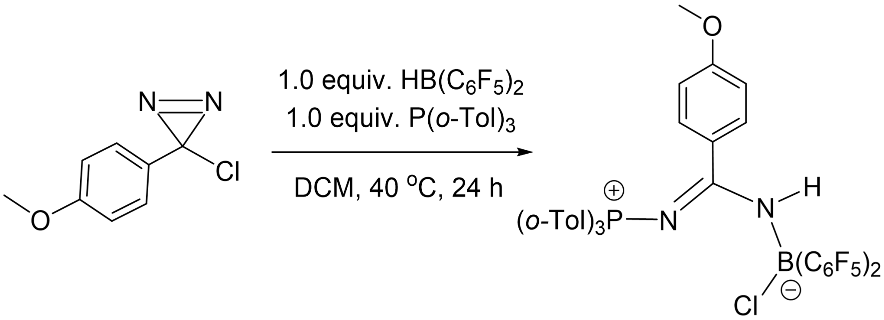

6 (64 mg, 77%) was prepared by following the protocol for **1** whilst the reaction vial was heated at 40 °C for 24 h. **6**: ^{1}H NMR (500 MHz, CD_2Cl_2): δ_H 9.37 (br s, 1 H, -N*H*), 8.25 (dt, *J* = 9.0, 2.7 Hz, 2 H, Ar-*H*), 7.67 (dt, *J* = 8.0, 1.8 Hz, 2 H, Ar-*H*), 7.52 – 7.46 (m, 6 H, Ar-*H*), 7.67 (td, *J* = 7.5, 2.2 Hz, 3 H, Ar-*H*), 6.94 (dt, *J* = 9.1, 1.9 Hz, 2 H, Ar-*H*), 3.85 (s, 3 H, -OC*H*$_3$), 2.25 (s, 9 H, -C*H$_3$*); ^{31}P NMR (203 MHz, CD_2Cl_2): δ_P 22.8 (s, 1 P, -*P*(*o*-Tol)$_3$); ^{19}F NMR (471 MHz, CD_2Cl_2): δ_F -133.0 (m, 4 F, *o*-C_6F_5 of -BCl(C_6F_5)$_2$), -160.7 (m, 2 F, *p*-C_6F_5 of -BCl(C_6F_5)$_2$), -165.7 (m, 4 F, *m*-C_6F_5 of -BCl(C_6F_5)$_2$); ^{11}B NMR (161 MHz, CD_2Cl_2): δ_B 3.6 (br s, 1 B, -*B*Cl(C_6F_5)$_2$); ^{13}C NMR (126 MHz, CD_2Cl_2): δ_C 166.7 (d, $^2J_{C\text{-}P}$ = 9.4 Hz, -P-N=*C*(Ar)-N-), 164.6 (s, C_{Ar}), 149.0 (br s, -C_6F_5), 146.9 (br s, -C_6F_5), 138.2 (br s, -C_6F_5), 136.2 (br s, -C_6F_5), 135.1 (d, $^4J_{C\text{-}P}$ = 2.7 Hz, C_{Ar}), 134.4 (d, $^2J_{C\text{-}P}$ = 13.6 Hz, C_{Ar}), 134.0 (d, $^3J_{C\text{-}P}$ = 11.0 Hz, C_{Ar}), 131.7 (s, C_{Ar}), 128.0 (d, $^3J_{C\text{-}P}$ = 13.0 Hz, C_{Ar}), 125.5 (d, $^2J_{C\text{-}P}$ = 20.1 Hz, C_{Ar}), 122.5 (s, $^1J_{C\text{-}P}$ = 96.0 Hz, C_{Ar}), 114.5 (s, C_{Ar}), 56.2 (s, -O*C*H$_3$), 22.4 (d, *J* = 4.8 Hz, *C*H$_3$ of P(*o*-Tol)$_3$).

Synthesis of 7

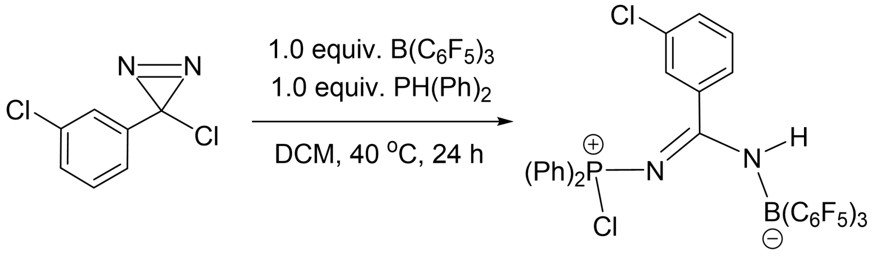

7 (76 mg, 74%) was prepared by following the protocol for **1** whilst the reaction vial was heated at 40 °C for 24 h. **7**: ^{1}H NMR (500 MHz, CH_2Cl_2): δ_H 7.87 – 7.80 (m, 1 H, Ar-*H*), 7.69 (tt, *J* = 8.4, 1.2 Hz, 2 H, Ar-*H*), 7.64 – 7.59 (m, 1 H, Ar-*H*), 7.57 – 7.52 (m, 1 H, Ar-*H*), 7.57 – 7.39 (m, 4 H, Ar-*H*and -N*H*), 7.36 (dt, *J* = 7.9, 1.1 Hz, 2 H, Ar-*H*), 7.36 (dt, *J* = 8.4, 1.2 Hz, 2 H, Ar-*H*), 7.29 – 7.21 (m, 2 H, Ar-*H*); ^{31}P NMR (203 MHz, CD_2Cl_2): δ_P29.6 (s, 1 P, -*P*(Ph)$_2$Cl); ^{19}F NMR (471 MHz, CD_2Cl_2): δ_F -134.4 (m, 4 F, *o*-C_6F_5 of -B(C_6F_5)$_3$), -160.3 (m, 3 F, *p*-C_6F_5 of -B(C_6F_5)$_3$), -165.3 (m, 4 F, *m*-C_6F_5 of -B(C_6F_5)$_3$); ^{11}B NMR (161 MHz, CD_2Cl_2): δ_B10.5 (br s, 1 B, -*B*(C_6F_5)$_3$); ^{13}C NMR (126 MHz, CD_2Cl_2): δ_C 169.3 (d, $^2J_{C\text{-}P}$ = 3.6 Hz, -P-N=*C*(Ar)-N-), 149.6 (br s, -C_6F_5), 147.7 (br s, -C_6F_5), 138.3 (br s, -C_6F_5), 136.4 (br s, -C_6F_5), 135.4 (s, C_{Ar}), 135.1 (d, $^4J_{C\text{-}P}$ = 3.6 Hz, C_{Ar}), 131.9 (s, C_{Ar}), 131.6 (d, $^3J_{C\text{-}P}$ = 12.7 Hz, C_{Ar}),

131.1 (s, C_{Ar}), 130.0 (s, C_{Ar}), 129.8 (d, $^{2}J_{C-P}$ = 15.4 Hz, C_{Ar}), 128.7 (s, C_{Ar}), 127.6 (s, C_{Ar}), 126.4 (d, $^{1}J_{C-P}$ = 233.4 Hz, C_{Ar}).

Synthesis of 8

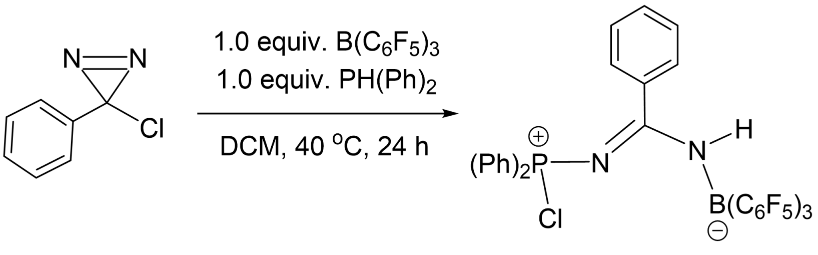

8 (69 mg, 81%) was prepared by following the protocol for **1** whilst the reaction vial was heated at 40 °C for 24 h. **7**:^{1}H NMR (500 MHz, CD_2Cl_2): δ_H 7.67 (tt, J = 7.5, 1.3 Hz, 2 H, Ar-H), 7.49 (tt, J = 7.3, 1.6 Hz, 1 H, Ar-H), 7.48 – 7.42 (m, 6 H, Ar-H), 7.42 – 7.30 (m, 6 H, Ar-$H$and 1 H of NH);^{31}P NMR (203 MHz, CD_2Cl_2): δ_P 28.2 (s, 1 P, -P(Ph)$_2$Cl);^{19}F NMR (471 MHz, CD_2Cl_2): δ_F -134.4 (m, 4 F, o-C_6F_5 of -B(C_6F_5)$_3$), -160.6 (m, 3 F, p-C_6F_5 of -B(C_6F_5)$_3$), -165.5 (m, 4 F, m-C_6F_5 of -B(C_6F_5)$_3$); ^{11}B NMR (161 MHz, CD_2Cl_2): δ_B−10.6 (br s, 1 B, -B(C_6F_5)$_3$); ^{13}C NMR (126 MHz, CD_2Cl_2): δ_C 170.8 (d, $^{2}J_{C-P}$ = 3.8 Hz, -P-N=C(Ar)-N-), 149.6 (br s, -C_6F_5), 147.7 (br s, -C_6F_5), 138.3 (br s, -C_6F_5), 136.5 (br s, -C_6F_5), 134.9 (d, $^{2}J_{C-P}$ = 3.6 Hz), 131.9 (s, C_{Ar}), 131.5 (d, $^{3}J_{C-P}$ = 12.9 Hz), 129.7 (d, $^{2}J_{C-P}$ = 15.7 Hz), 129.4 (s, C_{Ar}), 128.6 (d, $^{1}J_{C-P}$ = 133.5 Hz,), 127.3 (s, C_{Ar}). 126.4 (s, C_{Ar}).

Synthesis of 9

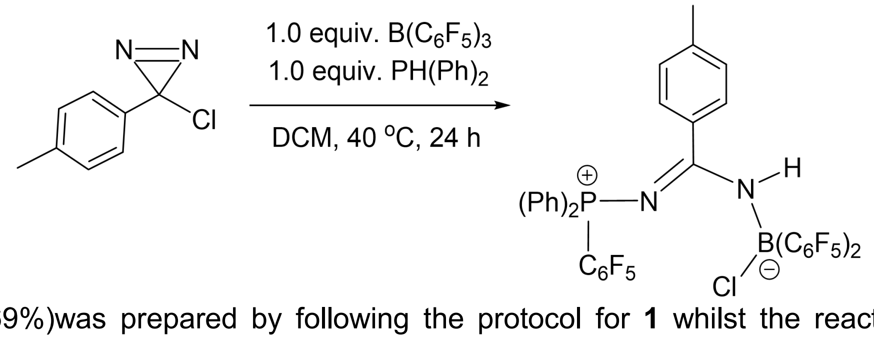

9 (60 mg, 69%)was prepared by following the protocol for **1** whilst the reaction vial was heated at 40 °C for 24 h. X-ray quality crystals were grown with a mixture of solvent of DCM:n-hexane (1:5) and stored at -30 °C for two days. **9**:^{1}H NMR (500 MHz, CD_2Cl_2): δ_H 8.78 (br s, 1 H, -NH), 8.04 (d, J = 8.4 Hz, 2 H, Ar-H), 7.94 (d, J = 6.9 Hz, 2 H, Ar-H), 7.91 (d,

J = 8.2 Hz, 2 H, Ar-*H*), 7.69 (td, J = 6.9, 1.8 Hz, 2 H, Ar-*H*), 7.69 (td, J = 7.7, 1.9 Hz, 2 H, Ar-*H*), 7.27 (d, J = 8.1 Hz, 2 H, Ar-*H*), 2.4 (s, 3 H, -C*H*$_3$); ^{31}P NMR (203 MHz, CD_2Cl_2): δ_P 4.2 (s, 1 P, -*P*(Ph)$_2$(C_6F_5));^{19}F NMR (471 MHz, CD_2Cl_2): δ_F -132.1 (m, 2 F, *o*-C_6F_5 *of* P(C_6F_5)Ph_2), -133.9 (m, 4 F, *o*-C_6F_5 of -BCl(C_6F_5)$_2$), -158.9 (m, 2 F, *p*-C_6F_5 of -BCl(C_6F_5)$_2$), -161.4 (m, 2 F, *p*-C_6F_5 *of* P(C_6F_5)Ph_2), -164.6 (m, 2 F, *m*-C_6F_5 of -BCl(C_6F_5)$_2$), -166.3 (m, 2 F, *p*-C_6F_5 *of* P(C_6F_5)Ph_2); ^{11}B NMR (161 MHz, CD_2Cl_2): δ_B -7.0 (br s, 1 B, -*B*(C_6F_5)$_2$Cl); ^{13}C NMR (126 MHz, CD_2Cl_2): δ_C 167.1 (d, $^2J_{C\text{-}P}$ = 5.4 Hz, -P-N=*C*(Ar)-N-), 149.6 (br s, -*C*$_6$F$_5$), 148.8 (br s, -*C*$_6$F$_5$), 147.5 (br s, -*C*$_6$F$_5$), 146.8 (br s, -*C*$_6$F$_5$), 145.2 (s, C_{Ar}), 140.1 (br s, -*C*$_6$F$_5$), 138.6 (br s, -*C*$_6$F$_5$), 138.1 (br s, -*C*$_6$F$_5$), 136.1 (br s, -*C*$_6$F$_5$), 135.1 (d, $^4J_{C\text{-}P}$ = 2.6 Hz, C_{Ar}), 132.7 (d, $^3J_{C\text{-}P}$ = 11.7 Hz, C_{Ar}), 130.2 (d, $^2J_{C\text{-}P}$ = 14.8 Hz, C_{Ar}), 129.9 (s, C_{Ar}), 129.3 (s, C_{Ar}), 126.8 (s, C_{Ar}), 125.1 (d, $^1J_{C\text{-}P}$ = 118.9 Hz, C_{Ar}), 21.9 (s, *C*H_3 of Ar).

Synthesis of 10

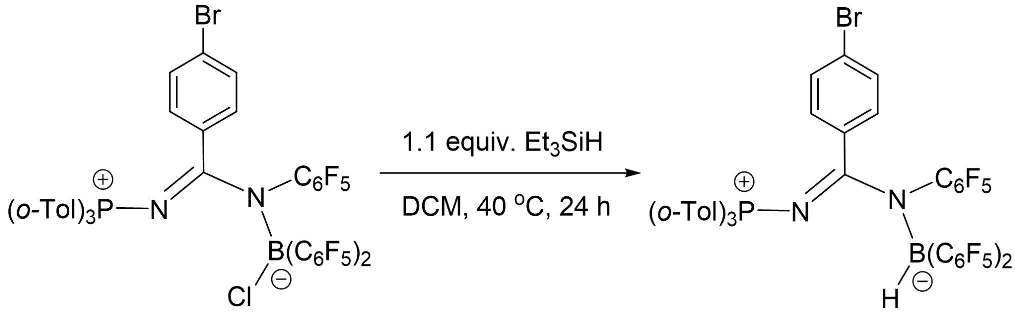

Into a 4 mL open top PTFE vial equipped with a stir bar, compound **1** (52.4 mg, 0.05 mmol, 1.0 equiv.) was dissolved in DCM (1.0 mL). After addition of Et_3SiH (9.6 µL, 0.06 mmol, 1.1 equiv.), the reaction mixture was allowed to stir at 40 °C for 24 h. After removal of all volatiles, the residue was washed with *n*-hexane (3 x 1 mL). Further, the residue was dried in affording compound **10** (44 mg, 87%). *X*-ray quality crystals were grown with a mixture of solvent of DCM:*n*-hexane (1:5) and stored at -30 °C for two days. Due to limited solubility partial characterization data included here. **10**: ^{1}H NMR (500 MHz, CD_2Cl_2): δ_H 7.52 (t, J = 7.3 Hz, 4 H, Ar-*H*), 7.35 - 7.28 (m, 4 H, Ar-*H*), 7.20 (dd, J = 1.9, 4 H, Ar-*H*), 6.99 (d, J = 8.3, 2 H, Ar-*H*), 6.77 (d, J = 8.7 Hz, 2 H, Ar-*H*), 1.9 (s, 9 H, -C*H*$_3$); ^{31}P NMR (203 MHz, CD_2Cl_2): δ_P 22.4 (s, 1 P, -*P*(*o*-Tol)$_3$); ^{19}F NMR (471 MHz, CD_2Cl_2): δ_F -132.5 (m, 4 F, *o*-C_6F_5 of -

BH(C_6F_5)$_2$), -144.5 (m, 2 F, *o*-NC_6F_5), -158.4 (m, 2 F, *p*-C_6F_5 of -BH(C_6F_5)$_2$), -160.9 (m, 1 F, *p*-NC_6F_5), -164.4 (m, 2 F, *m*-NC_6F_5), -166.3 (m, 4 F, *m*-C_6F_5 of -BH(C_6F_5)$_2$).

Synthesis of 11

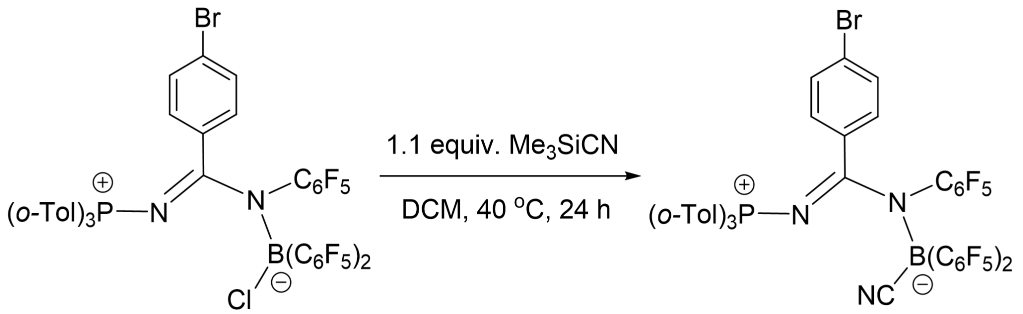

11 (43 mg, 83%) was prepared by following the protocol for **10** whilst Me_3SiCN (7.5 µL, 0.06 mmol, 1.1 equiv.) was added. *X*-ray quality crystals were grown with a mixture of solvent of DCM:*n*-hexane (1:5) and stored at -30 °C for two days. **11**: ^{1}H NMR (500 MHz, CD_2Cl_2): δ_H 8.72 (s, 1 H, Ar-*H*), 8.02 – 7.63 (m, 2 H, Ar-*H*), 7.63 – 7.26 (m, 4 H, Ar-*H*), 7.25 – 6.98 (m, 4 H, Ar-*H*), 6.97 – 6.49 (m, 5 H, Ar-*H*), 2.40 (s, 3 H, -C*H₃*), 2.09 (s, 3 H, -C*H₃*), 1.61 (s, 3 H, -C*H₃*); ^{31}P NMR (203 MHz, CD_2Cl_2): δ_P 24.7 (s, 1 P, -*P*(*o*-Tol)$_3$); ^{19}F NMR (471 MHz, CD_2Cl_2): δ_F -130.8 (m, 4 F, *o*-C_6F_5 of -B(CN)(C_6F_5)$_2$), -140.7 (m, 2 F, *o*-NC_6F_5), -154.3 (m, 2 F, *p*-C_6F_5 of -B(CN)(C_6F_5)$_2$), -155.7 (m, 1 F, *p*-NC_6F_5), -162.3 (m, 2 F, *m*-NC_6F_5), -164.1 (m, 4 F, *m*-C_6F_5 of -B(CN)(C_6F_5)$_2$); ^{11}B NMR (161 MHz, CD_2Cl_2): δ_B -13.9 (br s, 1 B, -*B*(CN)(C_6F_5)$_2$); ^{13}C NMR (126 MHz, CD_2Cl_2): δ_C 173.1 (d, $^2J_{C\text{-}P}$ = 5.6 Hz, -P-N=*C*(Ar)-N-), 149.8 (br s, -C_6F_5), 147.8 (br s, -C_6F_5), 145.1 (br s, -C_6F_5), 144.1 (br s, -C_6F_5), 143.7 (d, $^2J_{C\text{-}P}$ = 9.2 Hz), 143.2 (br s, -C_6F_5), 142.6 (br s, -C_6F_5), 141.8 (br s, -C_6F_5), 139.8 (br s, -C_6F_5), 138.4 (br m, C_{Ar}), 136.5 (br m, C_{Ar}), 135.0 (m, C_{Ar}), 134.9 (d, $^2J_{C\text{-}P}$ = 12.8 Hz), 134.2 (br m, C_{Ar}), 133.9 - 133.4 (m, C_{Ar}), 133.3 (br m, C_{Ar}), 132.8 (br m), 131.5 (s, C_{Ar}), 131.4 (br s, C_{Ar}), 130.0 (br m, C_{Ar}), 128.6 (br m, C_{Ar}), 128.3 (br m, C_{Ar}), 127.2 (br m, C_{Ar}), 126.1 (br m, C_{Ar}), 125.8 (s, C_{Ar}), 123.2 (d, $^1J_{C\text{-}P}$ = 80.1 Hz, C_{Ar}), 123.2 (d, $^1J_{C\text{-}P}$ = 96.1 Hz, C_{Ar}), 119.4 (d, $^1J_{C\text{-}P}$ = 88.8 Hz, C_{Ar}), 24.3 (br s, *C*H$_3$ of P(*o*-Tol)$_3$), 22.7 (br s, *C*H$_3$ of P(*o*-Tol)$_3$), 22.2 (br s, *C*H$_3$ of P(*o*-Tol)$_3$).

Synthesis of 12

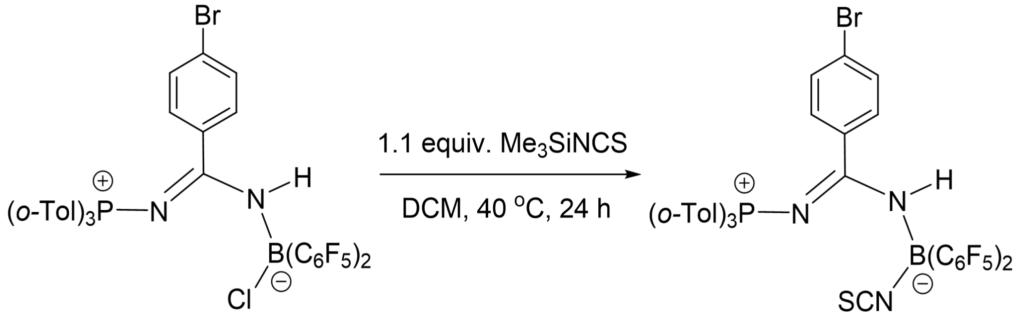

12 (36 mg, 80%) was prepared by following the protocol for **10** whilst compound **2** (44.1 mg, 0.05 mmol, 1.0 equiv.) and Me_3SiNCS (8.6 µL, 0.06 mmol, 1.1 equiv.) were added. *X*-ray quality crystals were grown with a mixture of solvent of DCM:*n*-hexane (1:5) and stored at -30 °C for two days. **12**: ^{1}H NMR (500 MHz, CD_2Cl_2): δ_H 7.56 – 7.45 (m, 3 H, Ar-*H*), 7.43 – 7.27 (m, 1 H, Ar-*H*), 7.27 – 7.10 (m, 3 H, Ar-*H*), 7.10 - 7.0 (m, 5 H, Ar-*H*), 2.0 (s, 9 H, -C*H*$_3$); ^{31}P NMR (203 MHz, CD_2Cl_2): δ_P 22.5 (s, 1 P, -*P*(*o*-Tol)$_3$); ^{19}F NMR (471 MHz, CD_2Cl_2): δ_F -135.0 (m, 4 F, *o*-C_6F_5 of -B(NCS)$(C_6F_5)_2$), -159.4 (m, 2 F, *p*-C_6F_5 of -B(NCS)$(C_6F_5)_2$), -164.7 (m, 4 F, *m*-C_6F_5 of -B(NCS)$(C_6F_5)_2$); ^{11}B NMR (161 MHz, CD_2Cl_2): δ_B -12.3 (br s, 1 B, -*B*(NCS)$(C_6F_5)_2$); ^{13}C NMR (126 MHz, CD_2Cl_2): δ_C 169.8 (d, $^2J_{C\text{-}P}$ = 4.2 Hz, -P-N=*C*(Ar)-N-), 149.4 (br s, -C_6F_5), 147.4 (br s, -C_6F_5), 143.5 (br s, -C_6F_5), 138.6 (br s, -C_6F_5), 136.5 (br s,C_{Ar}), 135.7 (br s, C_{Ar}), 135.2 (d, $^4J_{C\text{-}P}$ = 6.7 Hz, C_{Ar}), 134.4 (s, C_{Ar}), 134.0 (s, C_{Ar}), 131.7 (s, C_{Ar}), 128.8 (s, C_{Ar}), 127.2 (m, C_{Ar}), 125.7 (s, C_{Ar}), 121.3 (d, $^1J_{C\text{-}P}$ = 80.1 Hz, C_{Ar}), 22.4 (s, *C*H$_3$ of P(*o*-Tol)$_3$).

NMR spectra of all the compounds

Compound 1

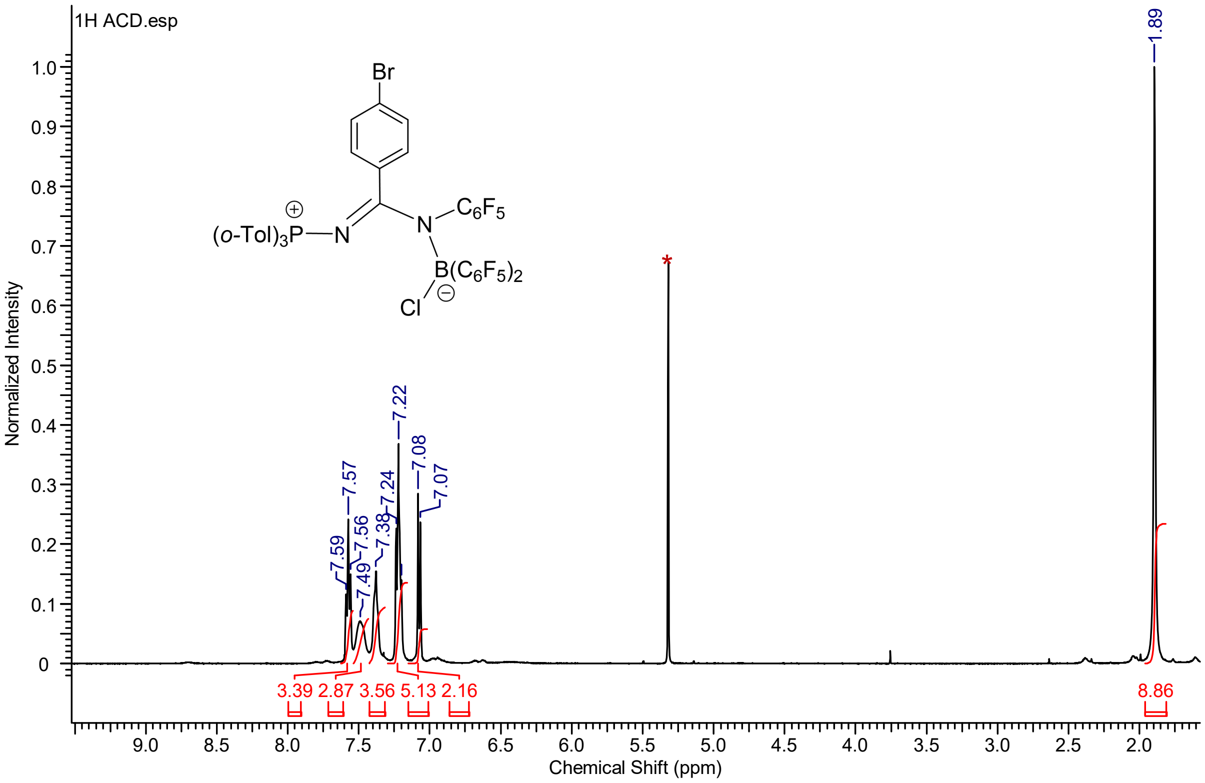

Figure S1. ^{1}H **NMR (500 MHz) spectrum of the compound 1 in CD_2Cl_2 (*= CD_2Cl_2).**

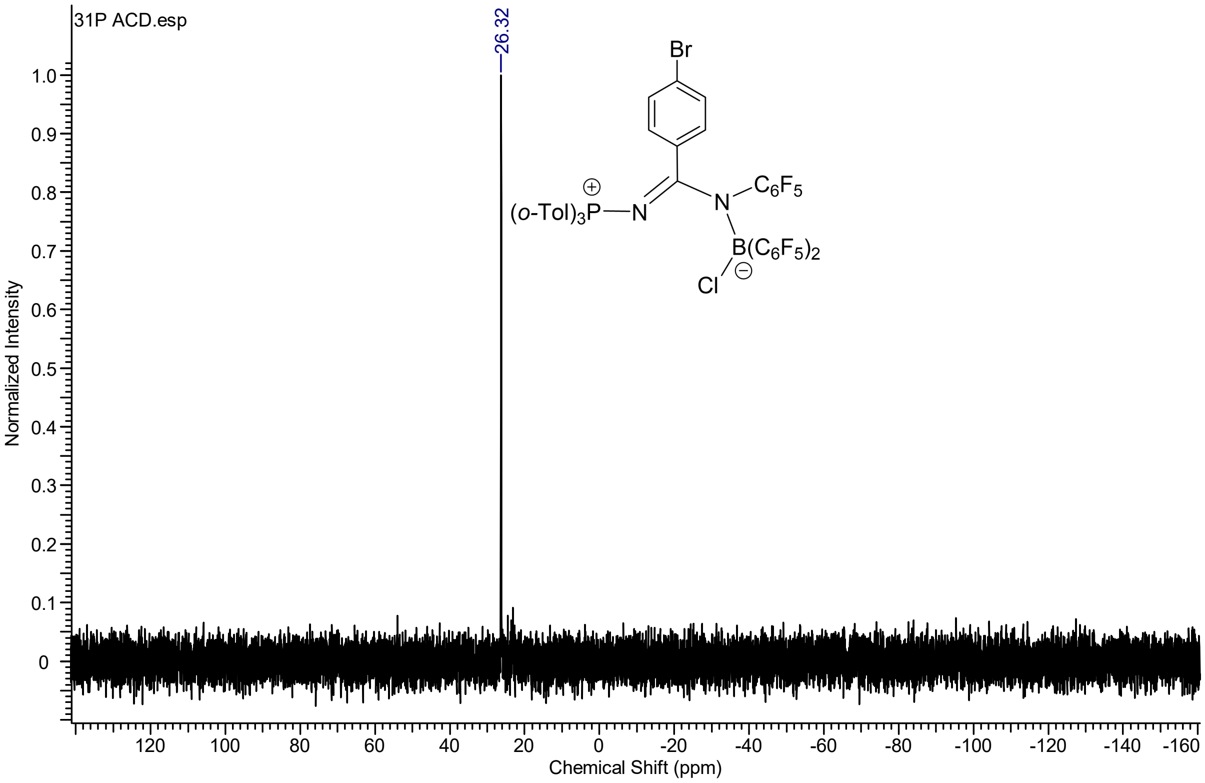

Figure S2. ^{31}P **NMR (203 MHz) spectrum of the compound 1 in CD_2Cl_2.**

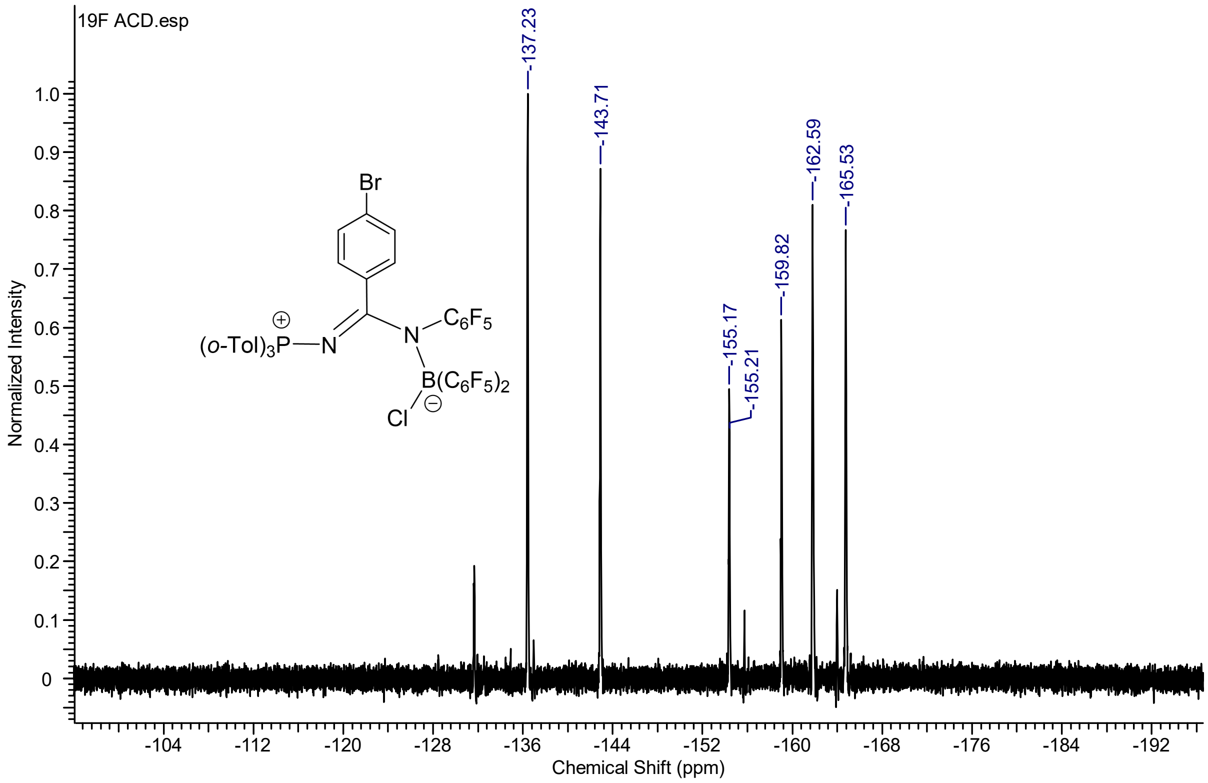

Figure S3. ^{19}**F NMR (471 MHz) spectrum of the compound 1 in** CD_2Cl_2.

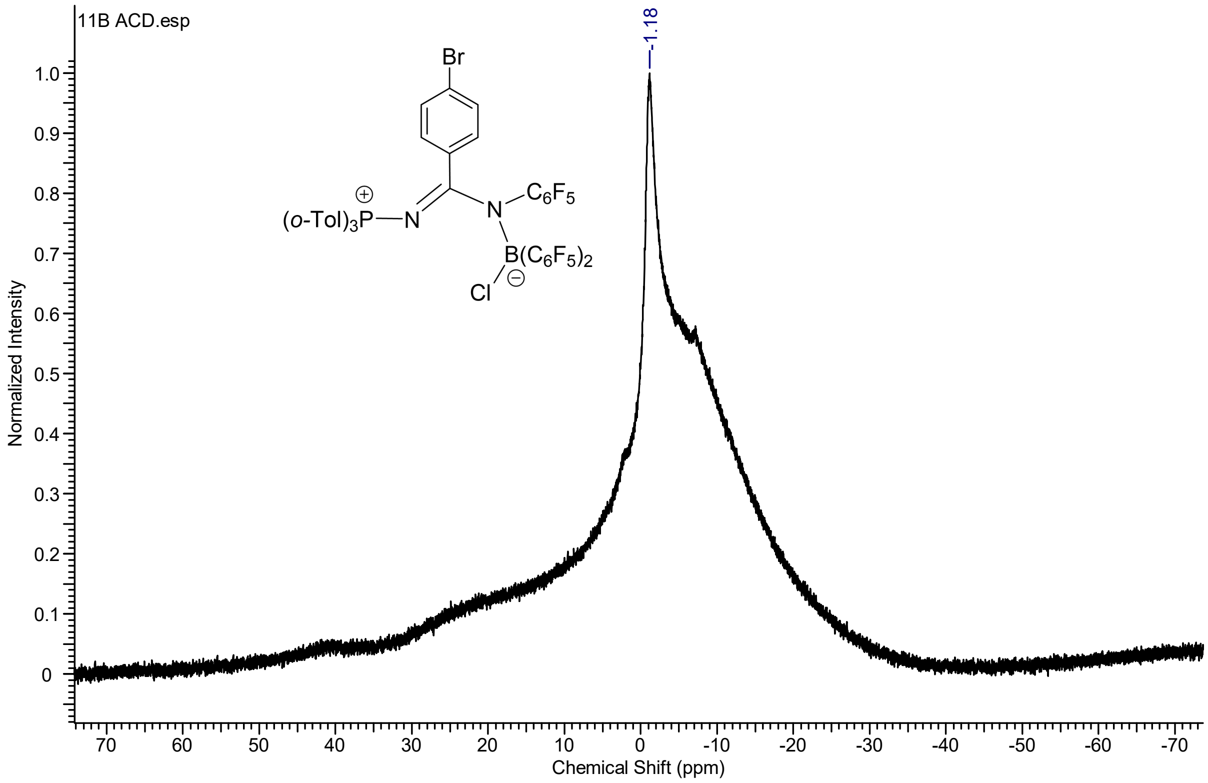

Figure S4. ^{11}**B NMR (161 MHz) spectrum of the compound 1 in** CD_2Cl_2.

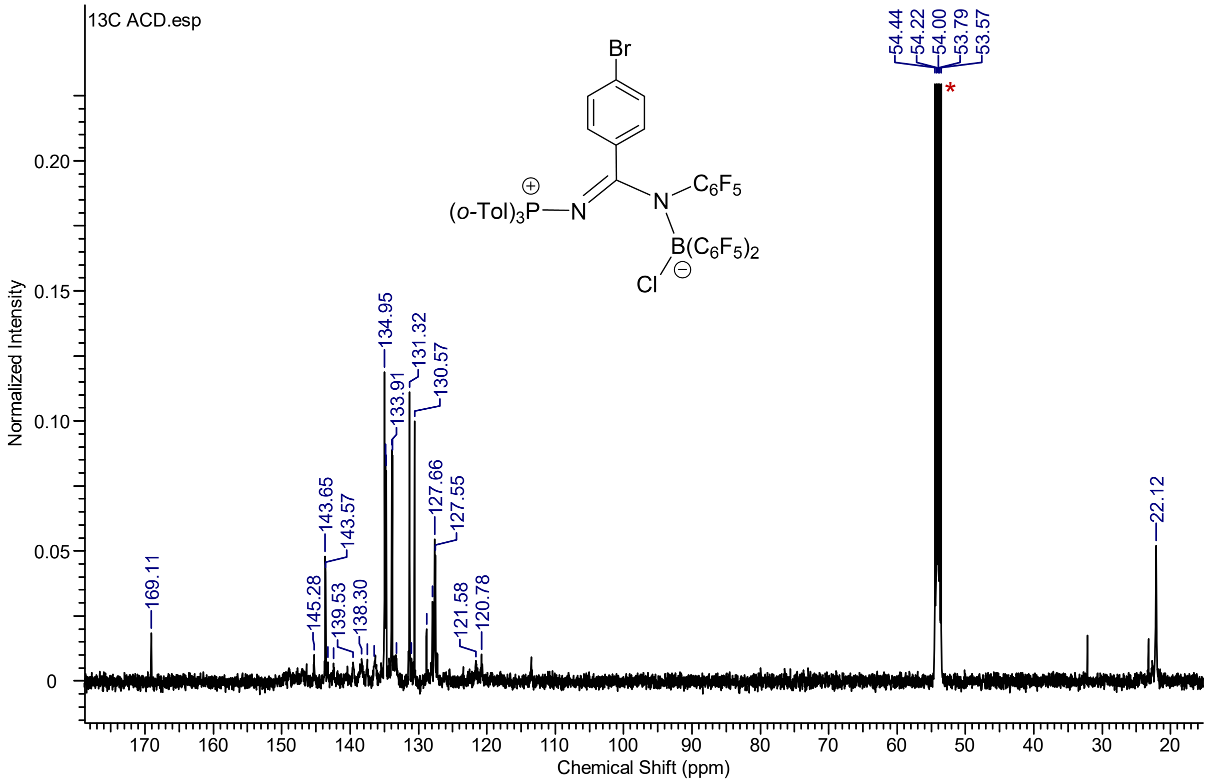

Figure S5. ^{13}C **NMR (126 MHz) spectrum of the compound 1 in** CD_2Cl_2 **(*=** CD_2Cl_2**).**

Compound 2

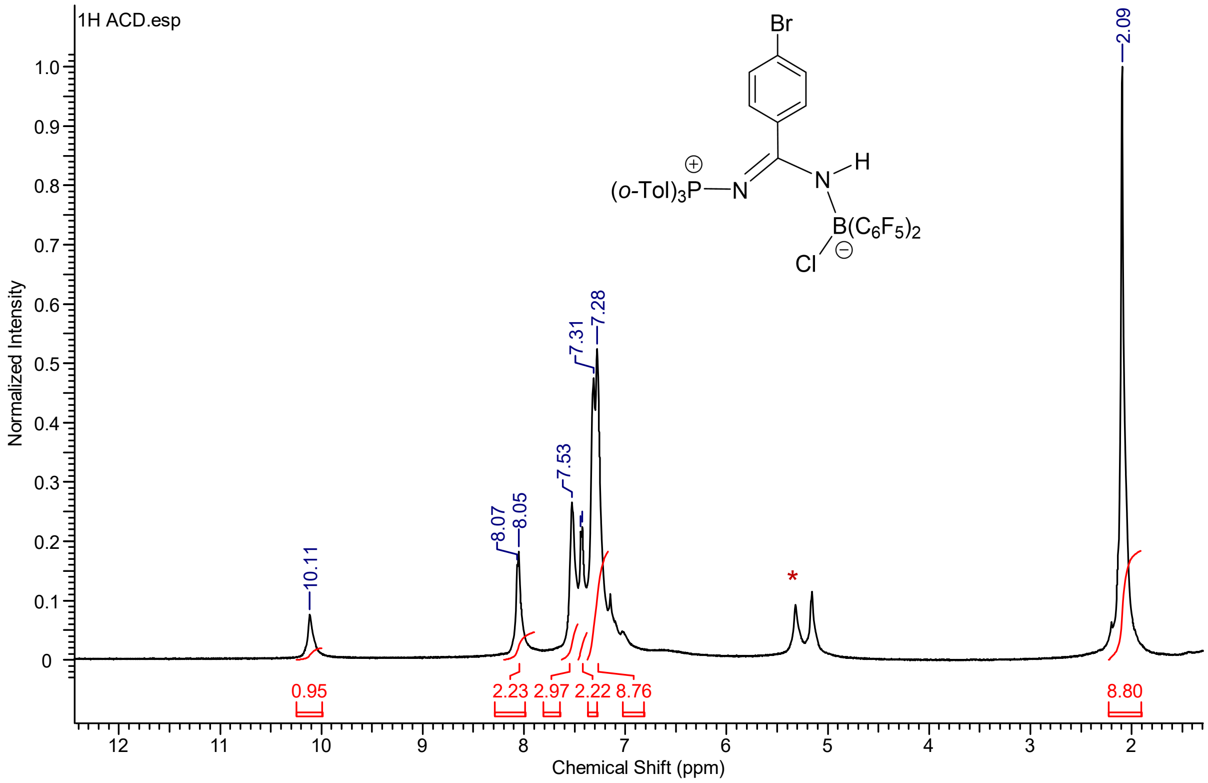

Figure S6. ^{1}H NMR (500 MHz) spectrum of the compound 2 in CD_2Cl_2 (*= CD_2Cl_2).

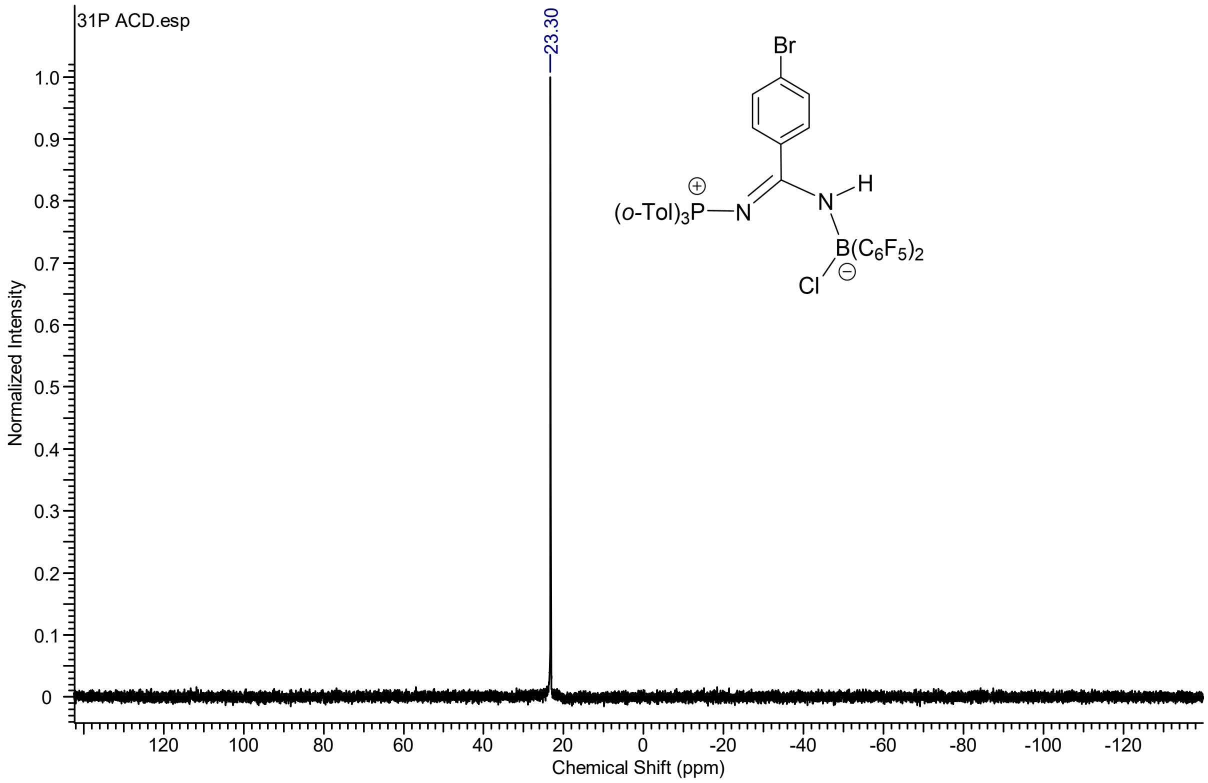

Figure S7. ^{31}P NMR (203 MHz) spectrum of the compound 2 in CD_2Cl_2.

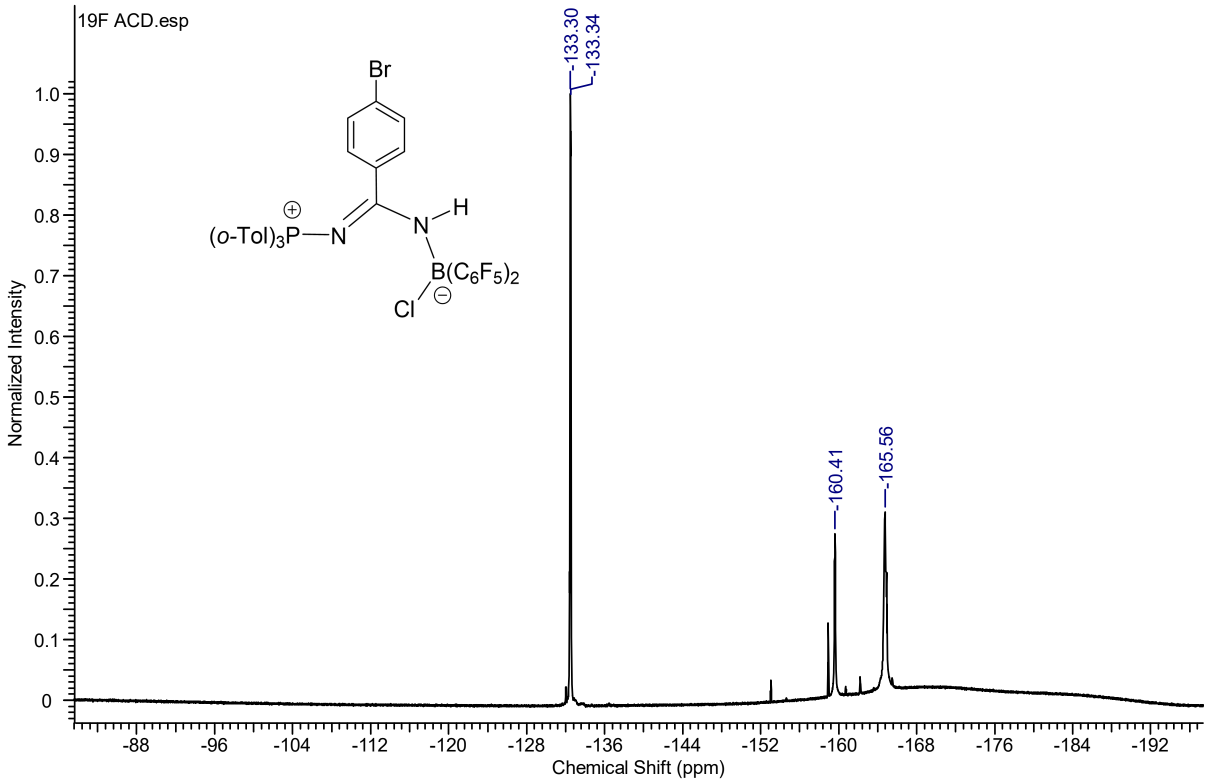

Figure S8. **^{19}F NMR (471 MHz) spectrum of the compound 2 in CD_2Cl_2.**

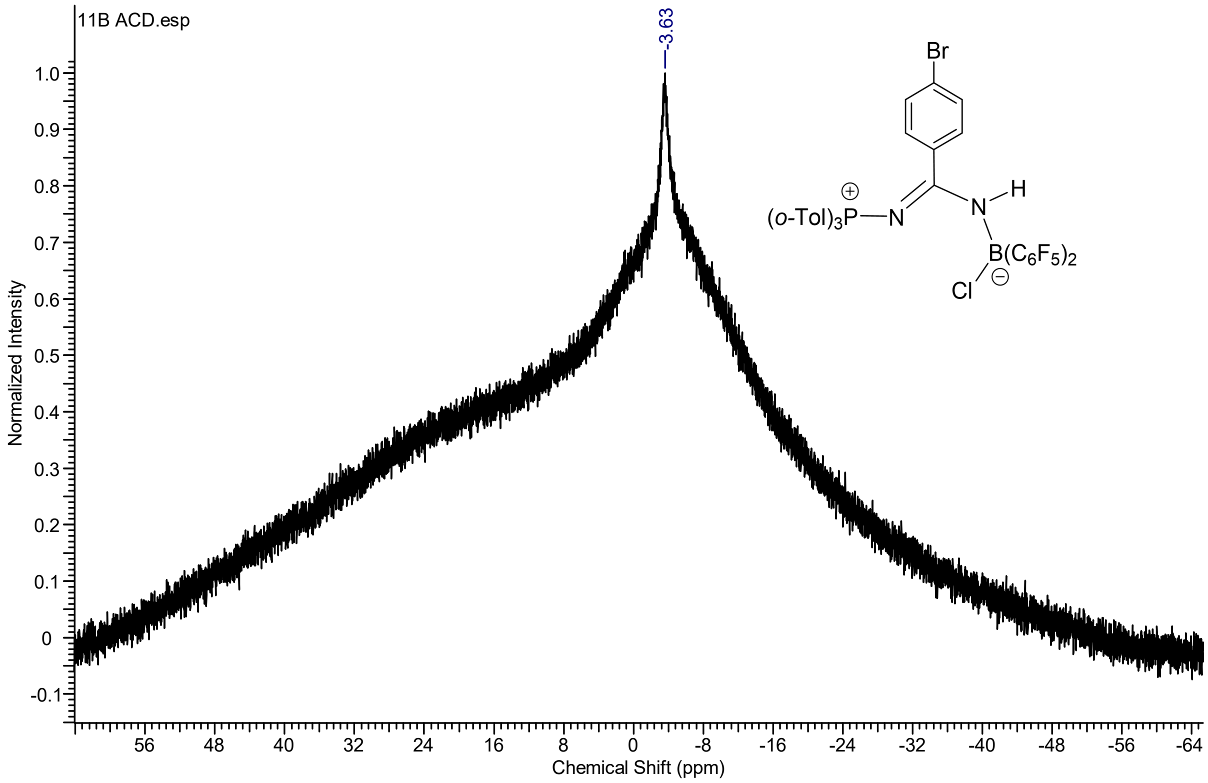

Figure S9. **^{11}B NMR (161 MHz) spectrum of the compound 2 in CD_2Cl_2.**

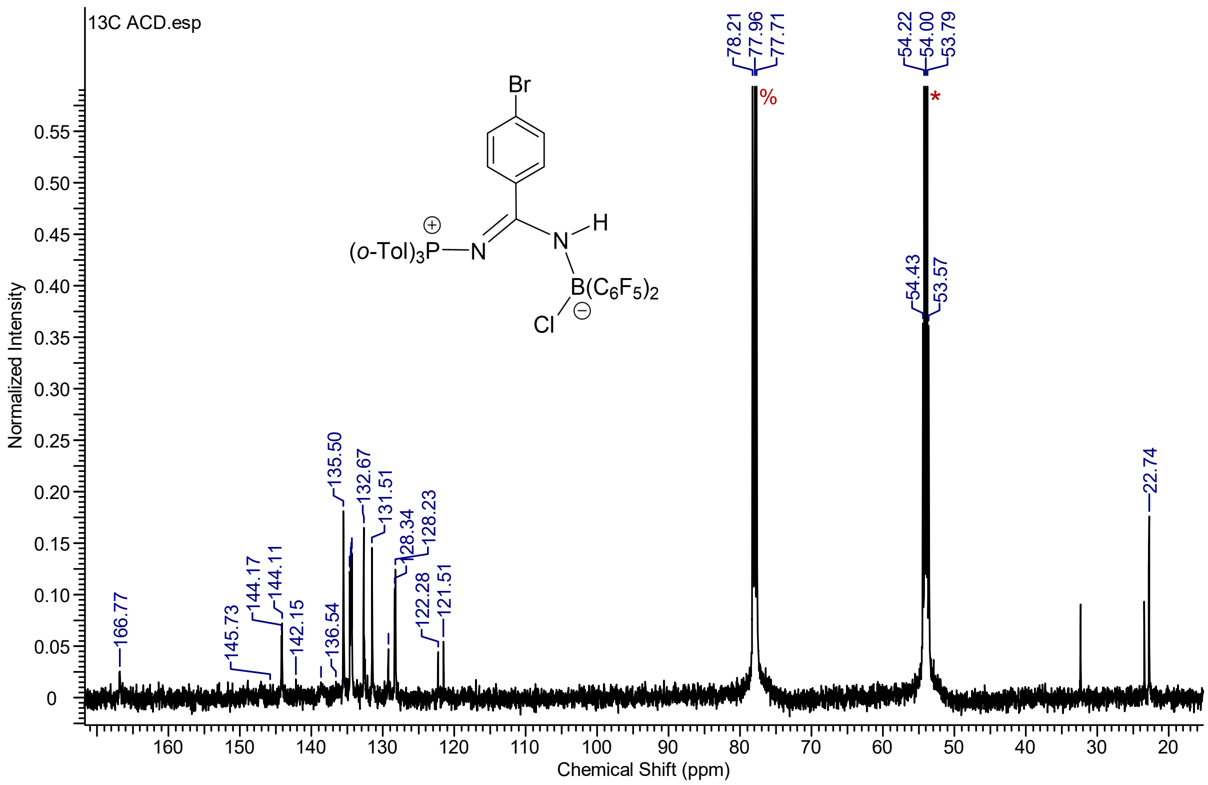

Figure S10. ^{13}C **NMR (126 MHz) spectrum of the compound 2 in** CD_2Cl_2 **(*=** CD_2Cl_2**, %=** $CDCl_3$**).**

Compound 3

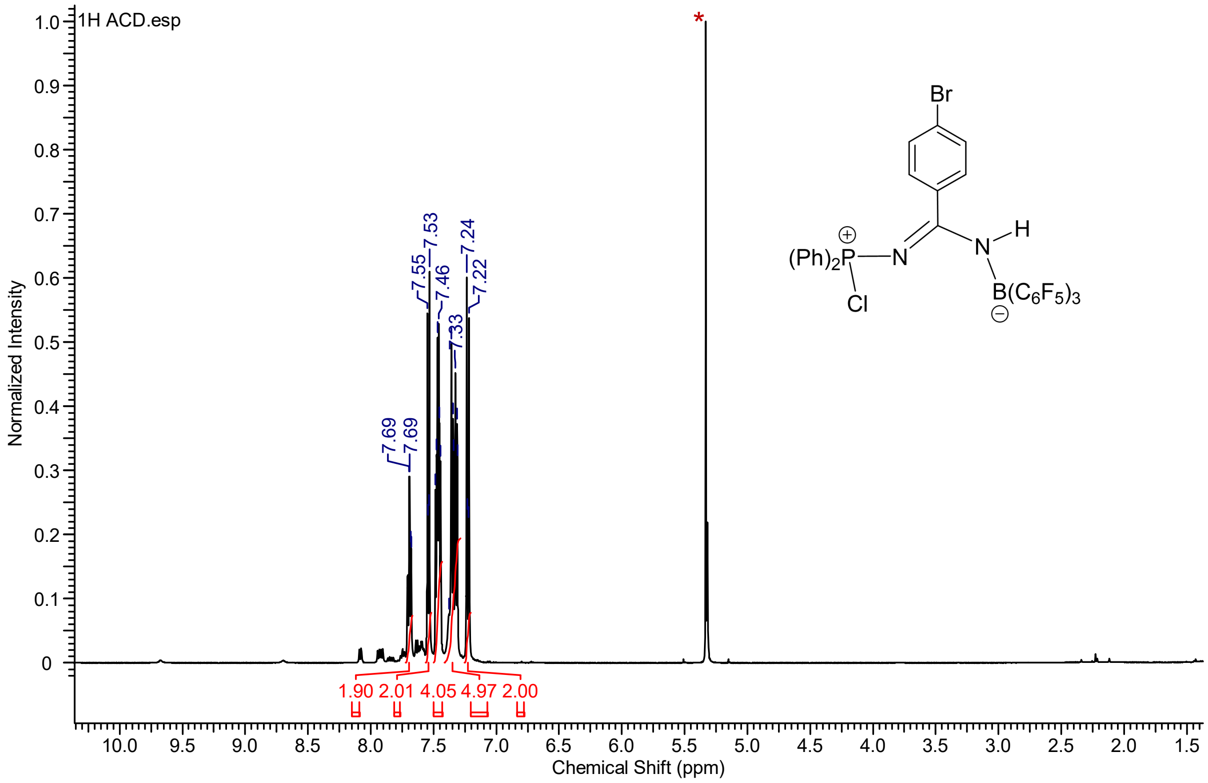

Figure S11. ^{1}H NMR (500 MHz) spectrum of the compound 3 in CD_2Cl_2 (*= CD_2Cl_2).

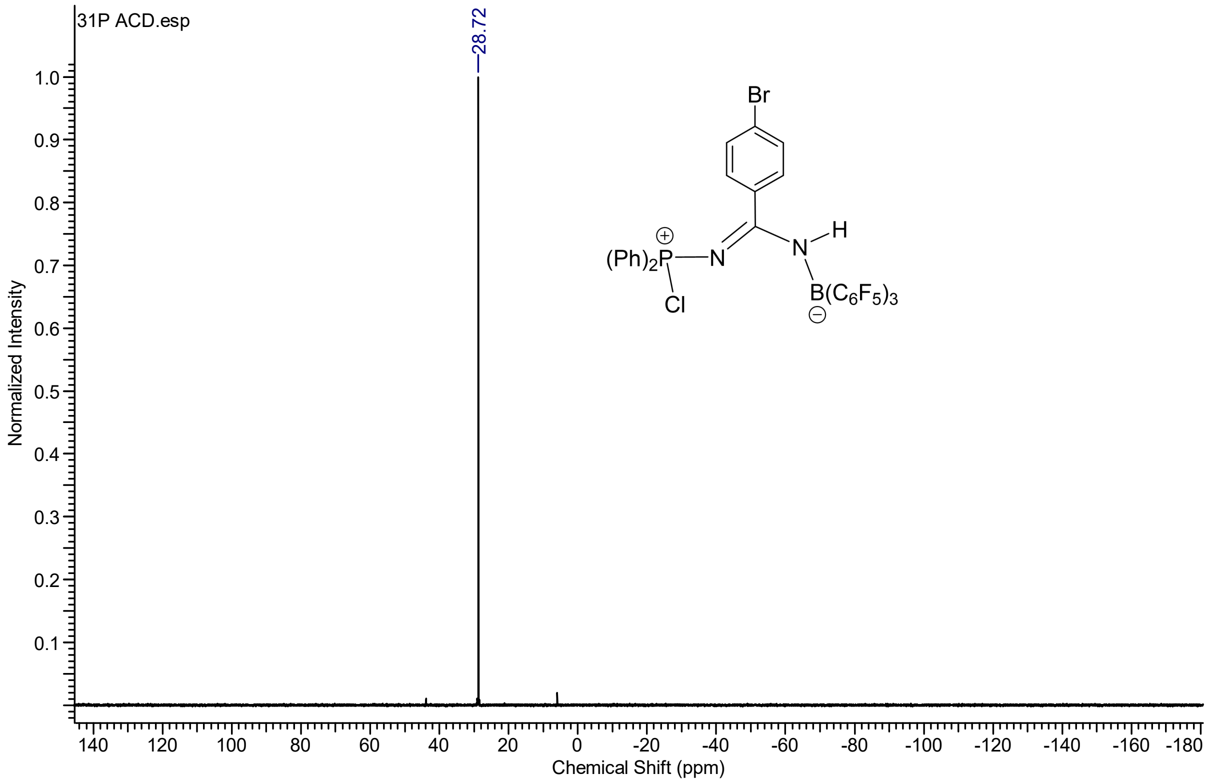

Figure S12. ^{31}P NMR (203 MHz) spectrum of the compound 3 in CD_2Cl_2.

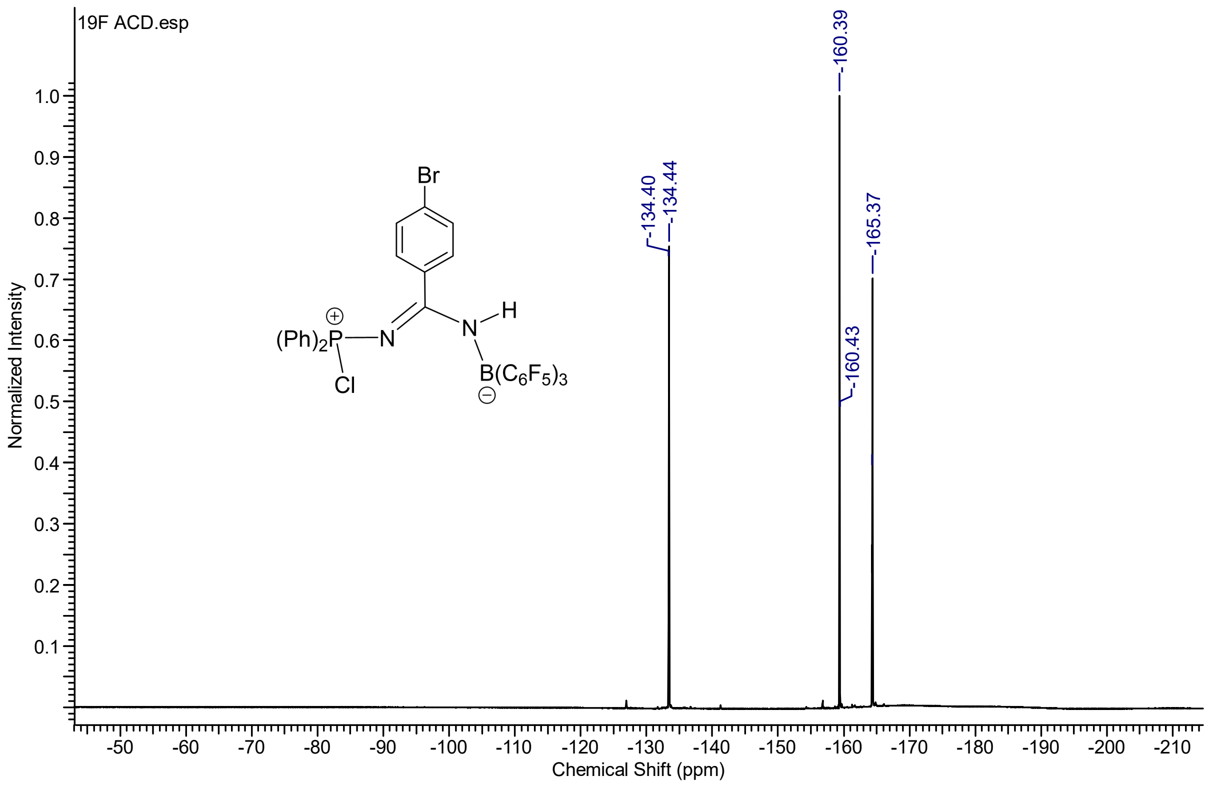

Figure S13. ^{19}**F NMR (471 MHz) spectrum of the compound 3 in** CD_2Cl_2**.**

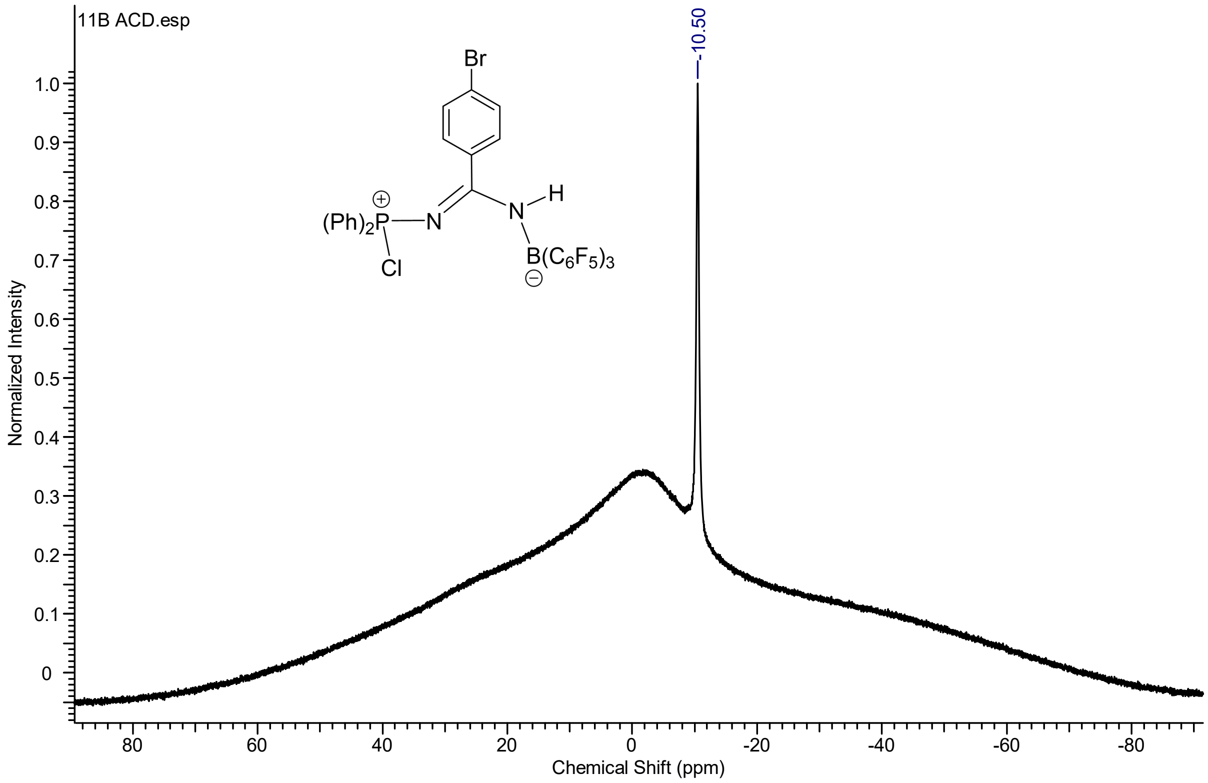

Figure S14. ^{11}**B NMR (161 MHz) spectrum of the compound 3 in** CD_2Cl_2**.**

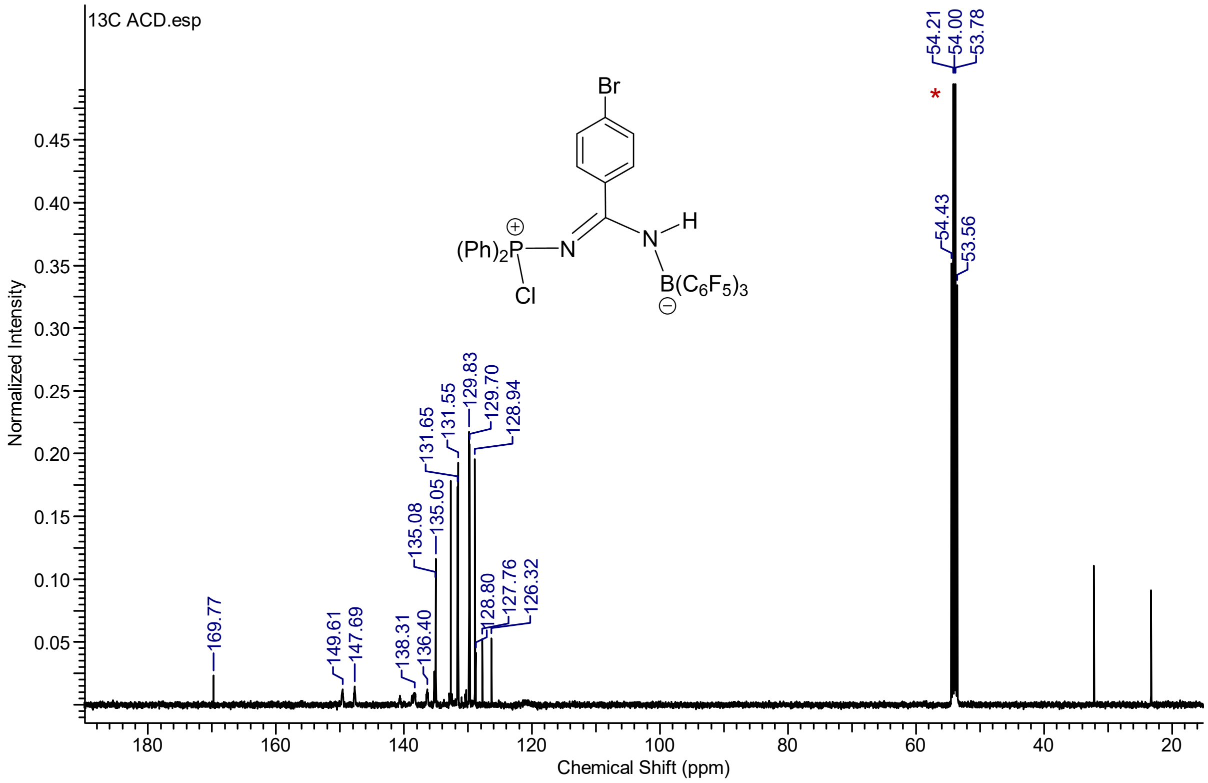

Figure S15. ^{13}C NMR (126 MHz) spectrum of the compound 3 in CD_2Cl_2 (*= CD_2Cl_2).

Compound 4

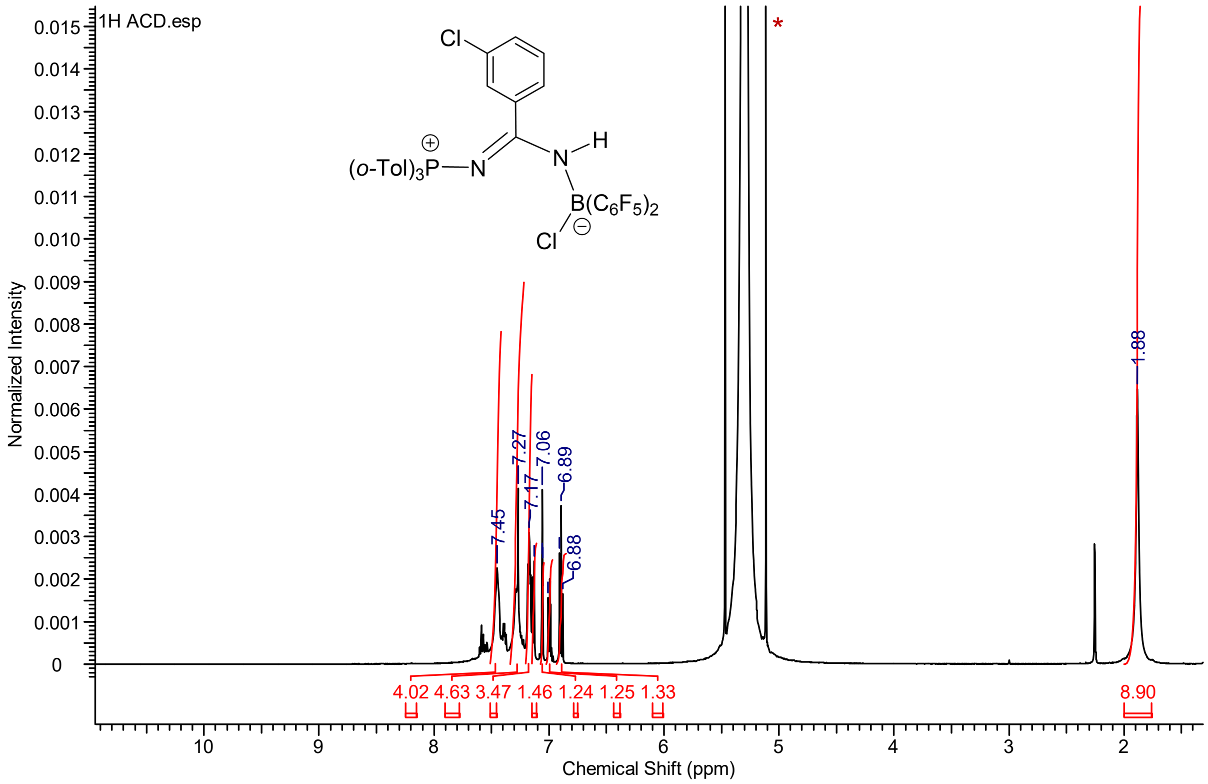

Figure S16. ^{1}H NMR (500 MHz) spectrum of the compound 4 in $CH_2Cl_2/CDCl_3$ (5:1) (*= CH_2Cl_2).

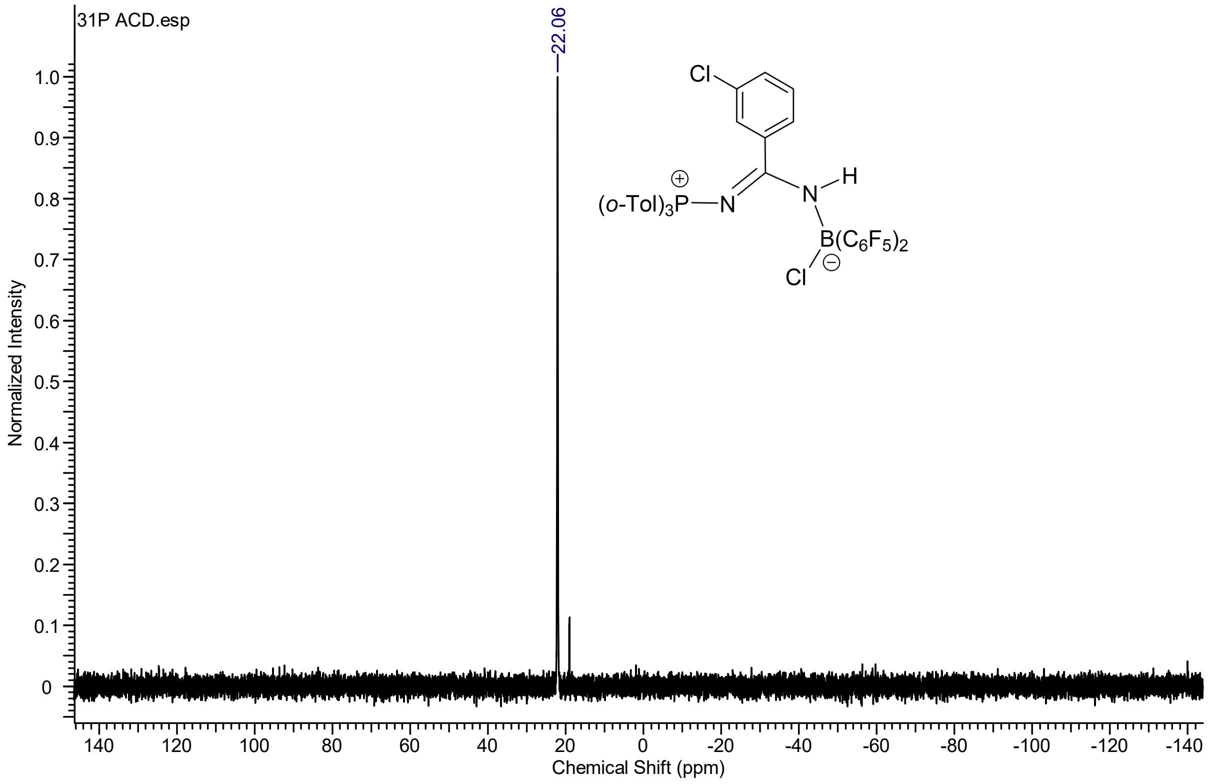

Figure S17. ^{31}P NMR (203 MHz) spectrum of the compound 4 in $CH_2Cl_2/CDCl_3$ (5:1).

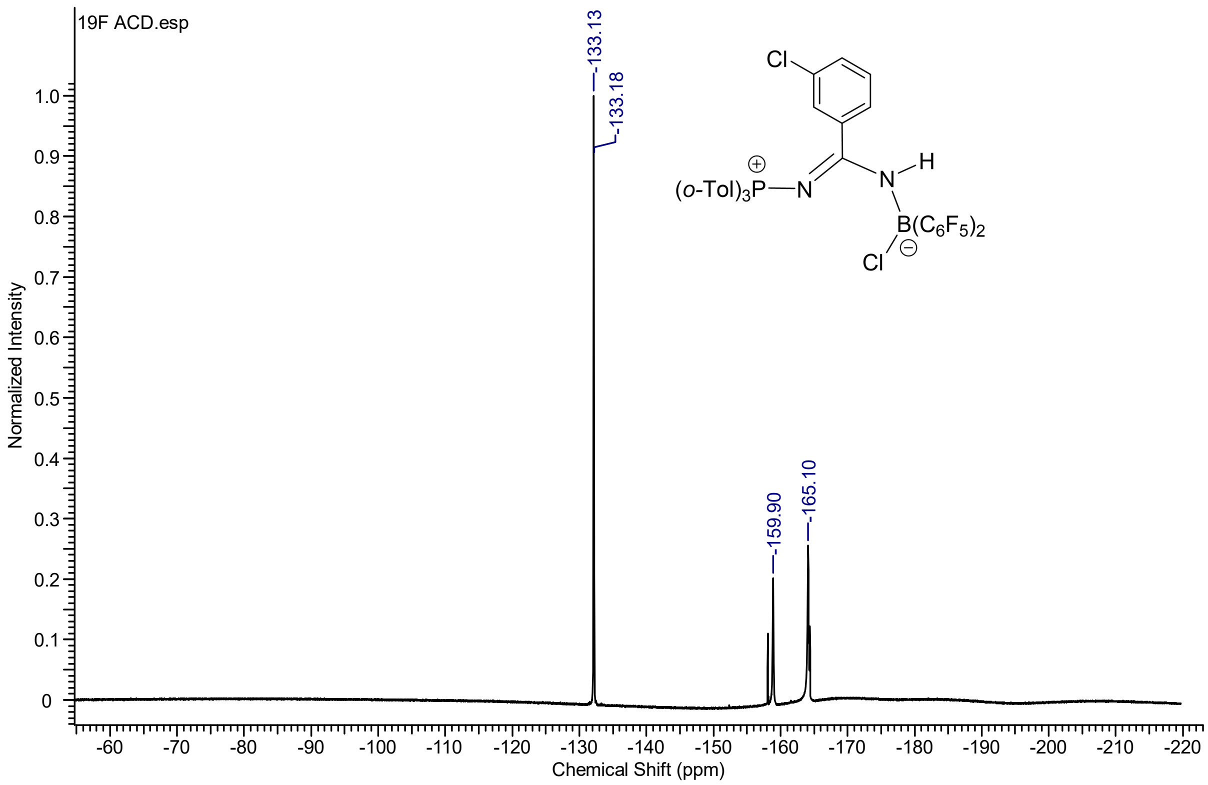

Figure S18. **^{19}F NMR (471 MHz) spectrum of the compound 4 in $CH_2Cl_2/CDCl_3$ (5:1).**

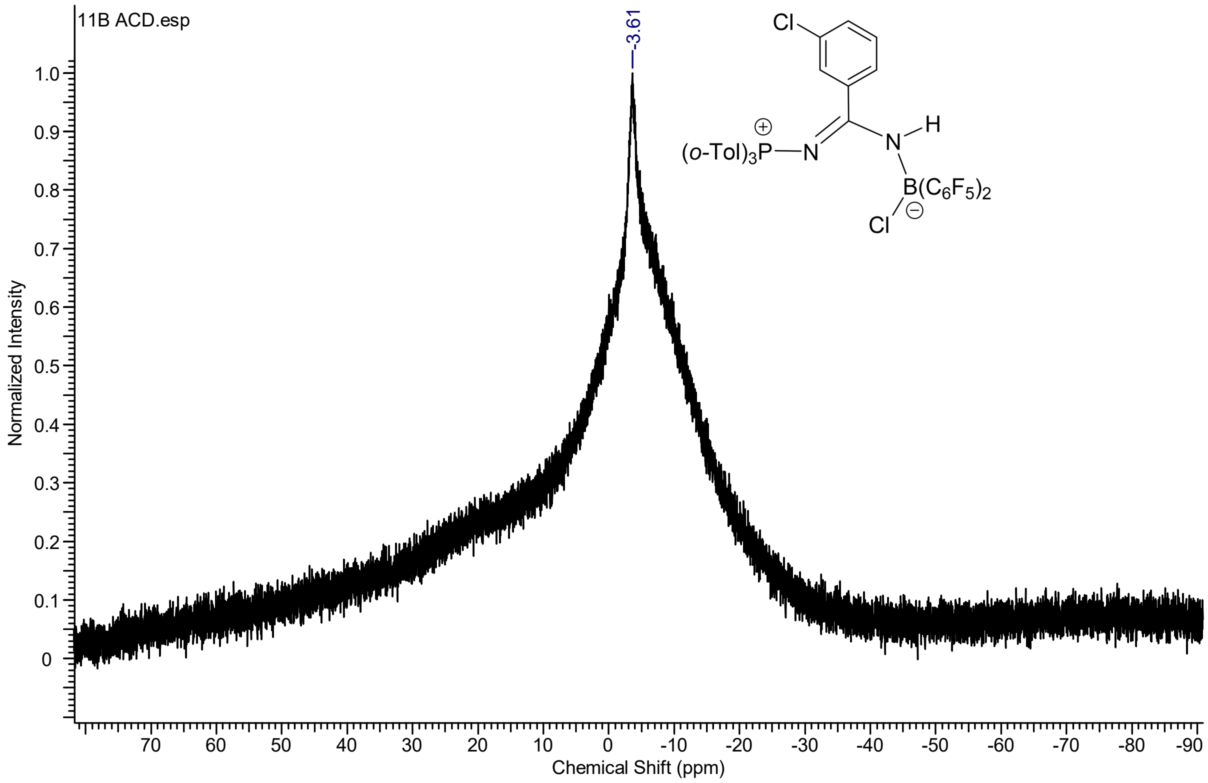

Figure S19. **^{11}B NMR (161 MHz) spectrum of the compound 4 in $CH_2Cl_2/CDCl_3$ (5:1).**

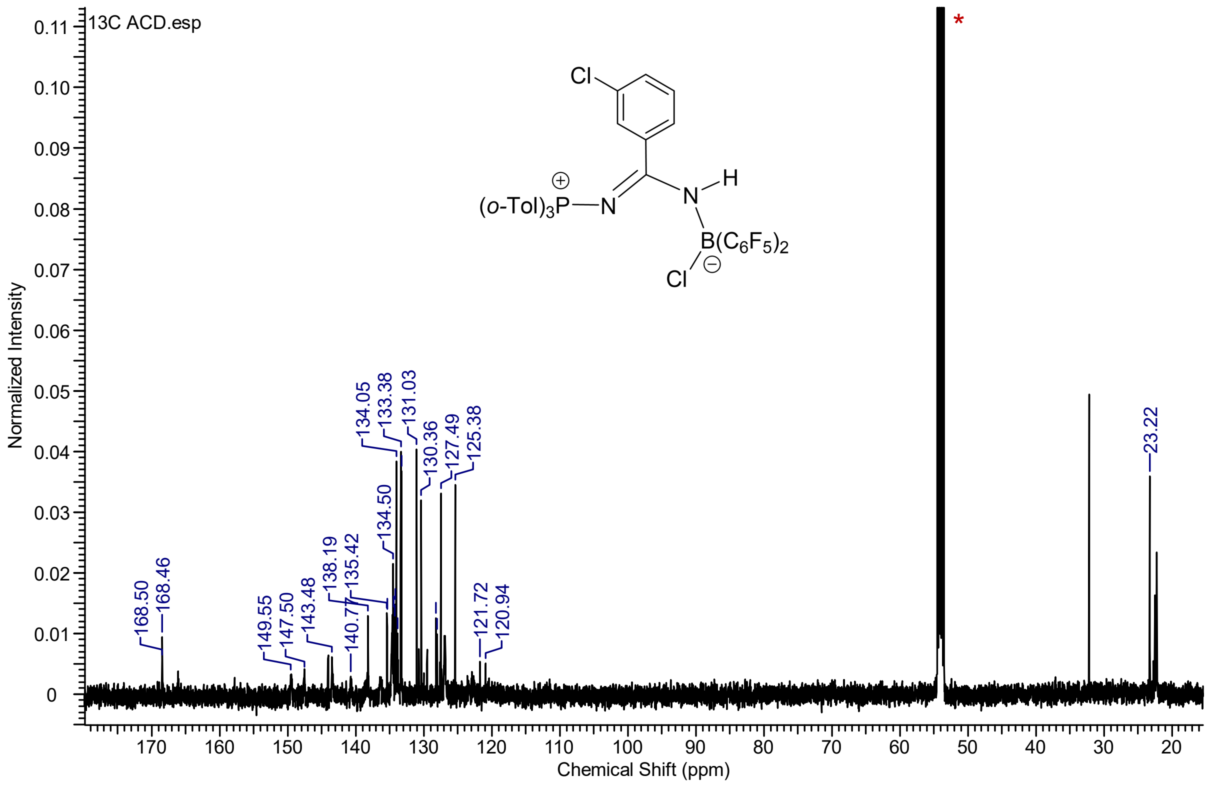

Figure S20. **^{13}C NMR (126 MHz) spectrum of the compound 4 in CD_2Cl_2 (*= CH_2Cl_2).**

Compound 5

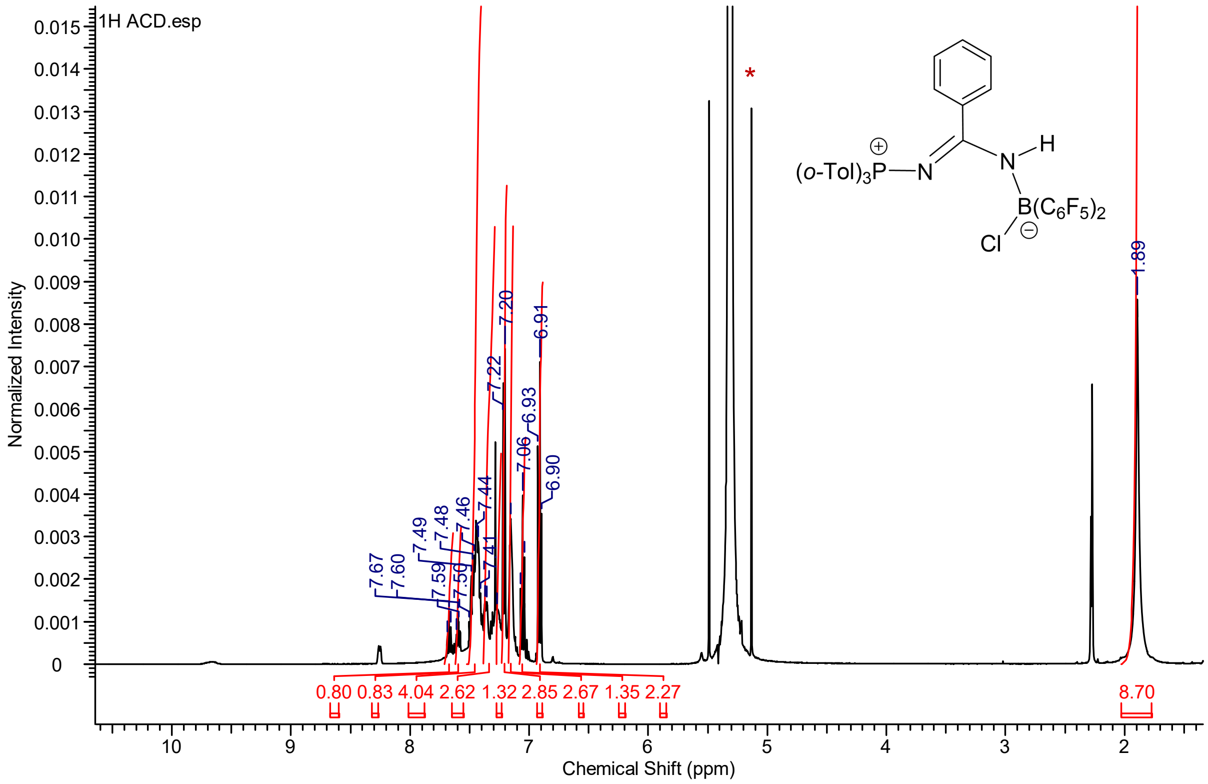

Figure S21. ^{1}**H NMR (500 MHz) spectrum of the compound 5 in** $CH_2Cl_2/CDCl_3$ **(5:1) (*=** CH_2Cl_2**).**

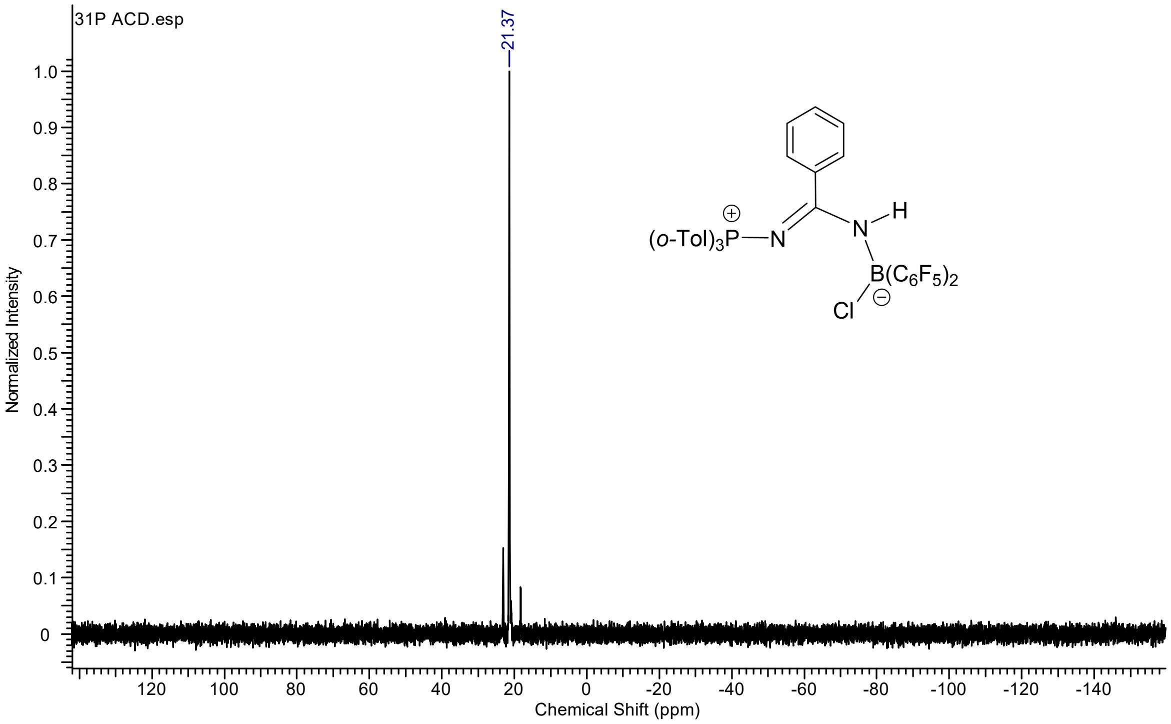

Figure S22. ^{31}**P NMR (203 MHz) spectrum of the compound 5 in** $CH_2Cl_2:CDCl_3$ **(5:1).**

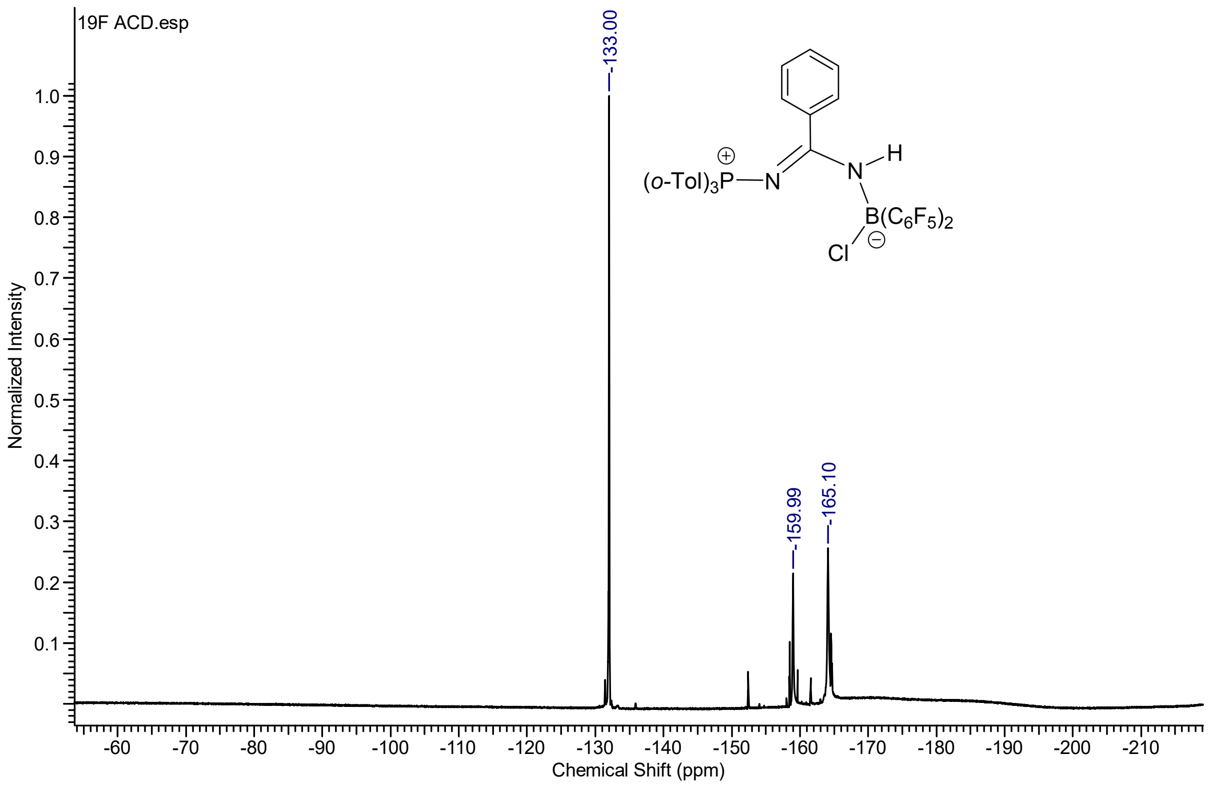

Figure S23. **^{19}F NMR (471 MHz) spectrum of the compound 5 in CH_2Cl_2:$CDCl_3$ (5:1).**

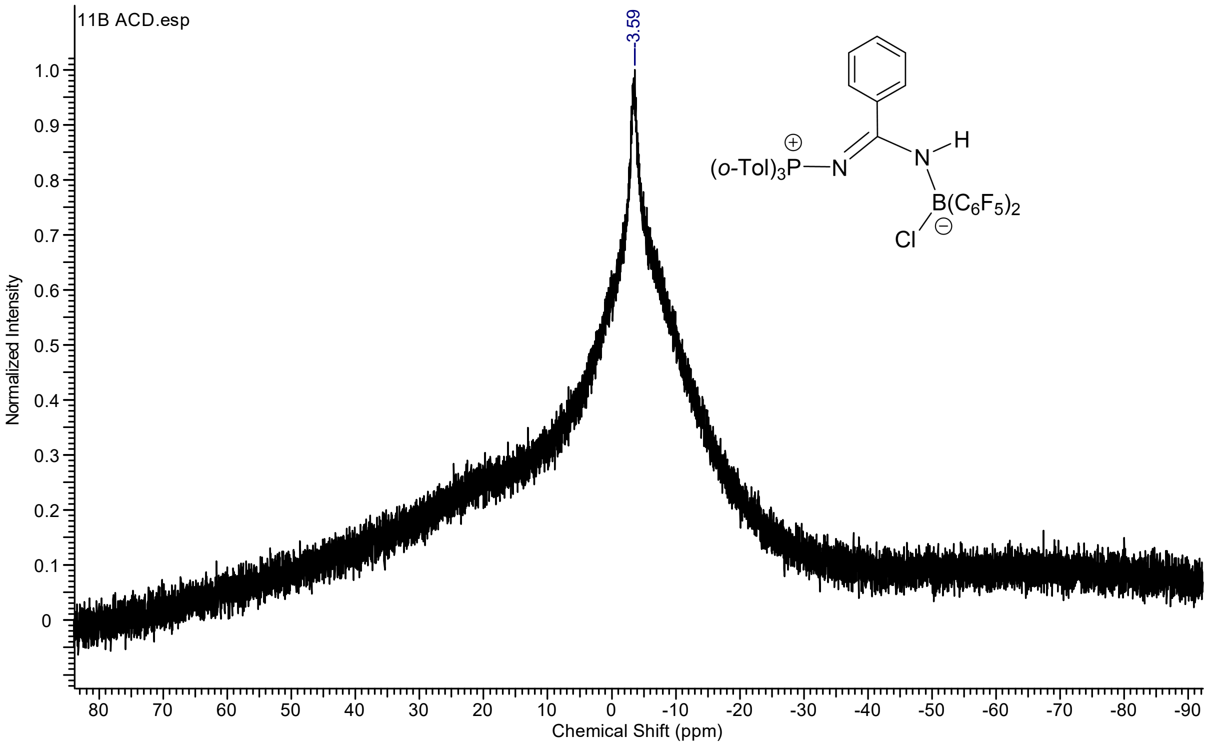

Figure S24. **^{11}B NMR (161 MHz) spectrum of the compound 5 in CH_2Cl_2:$CDCl_3$ (5:1).**

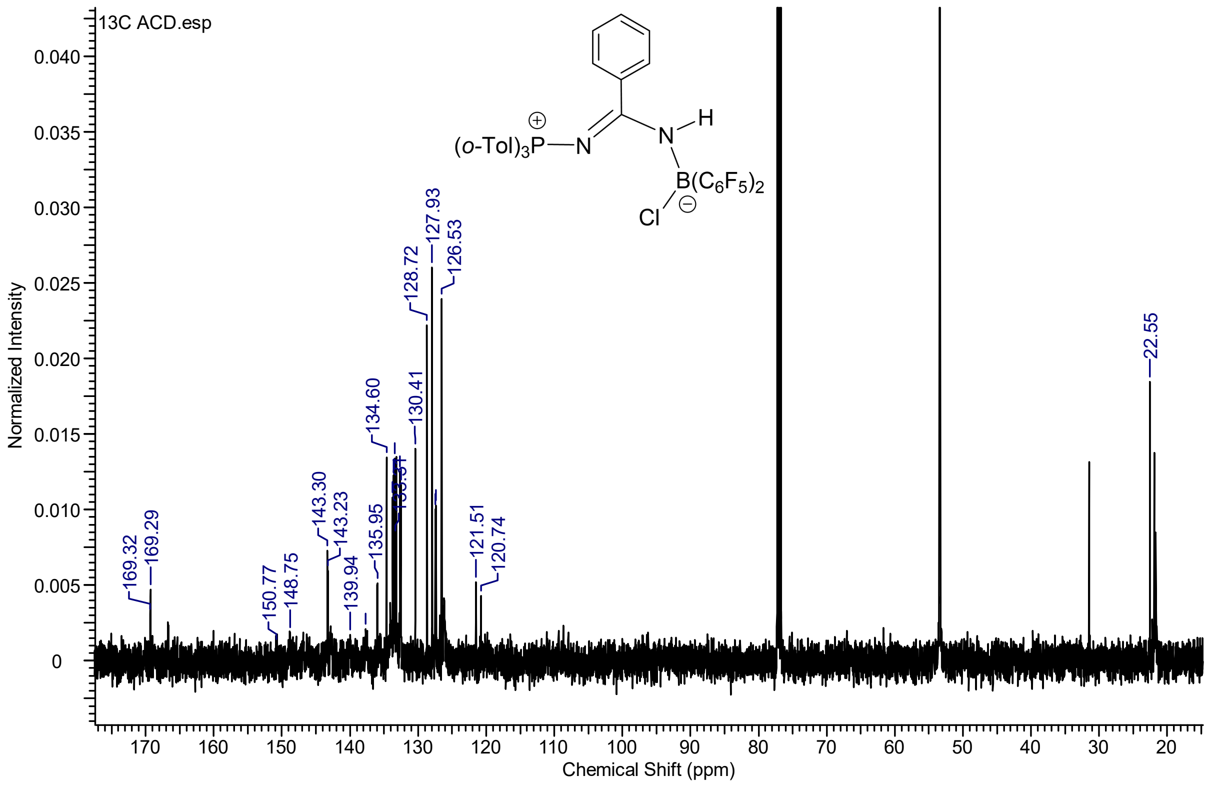

Figure S25. ^{13}C NMR (126 MHz) spectrum of the compound 5 in CH_2Cl_2:$CDCl_3$ (5:1) (*= CH_2Cl_2).

Compound 6

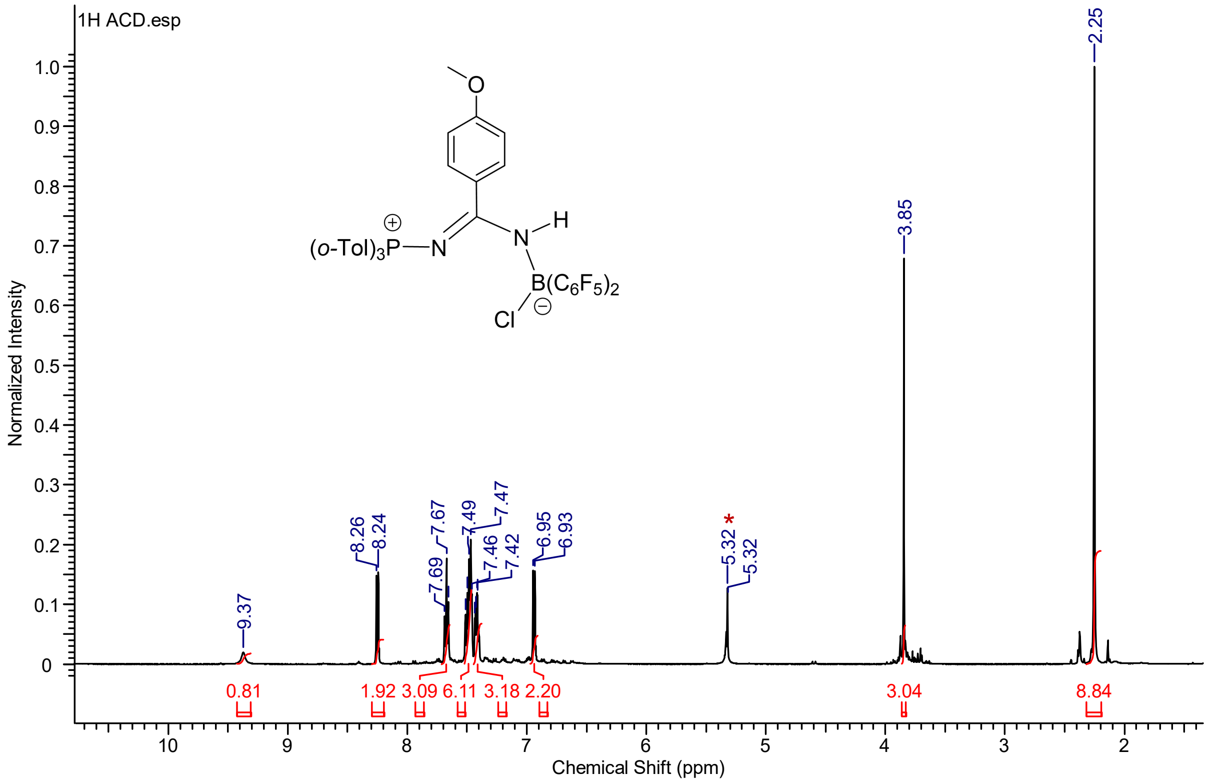

Figure S26. **^{1}H NMR (500 MHz) spectrum of the compound 6 in CD_2Cl_2 (*= CD_2Cl_2).**

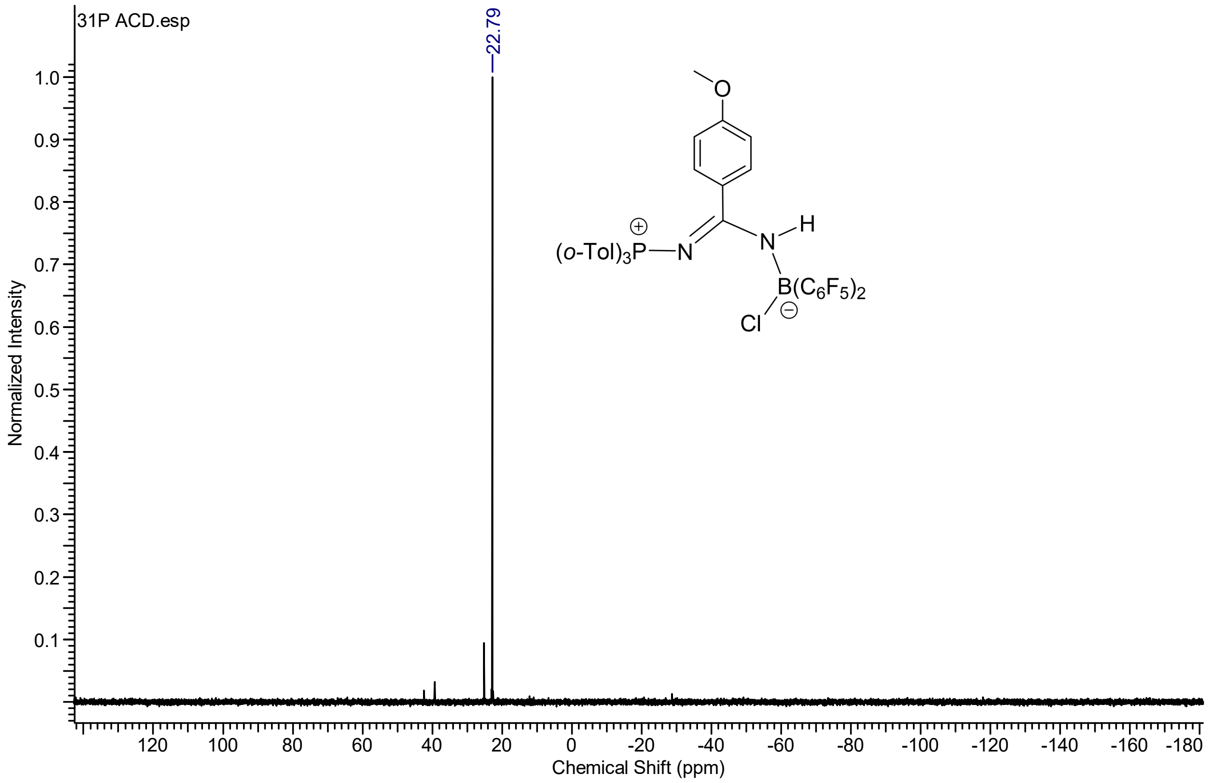

Figure S27. **^{31}P NMR (203 MHz) spectrum of the compound 6 in CD_2Cl_2.**

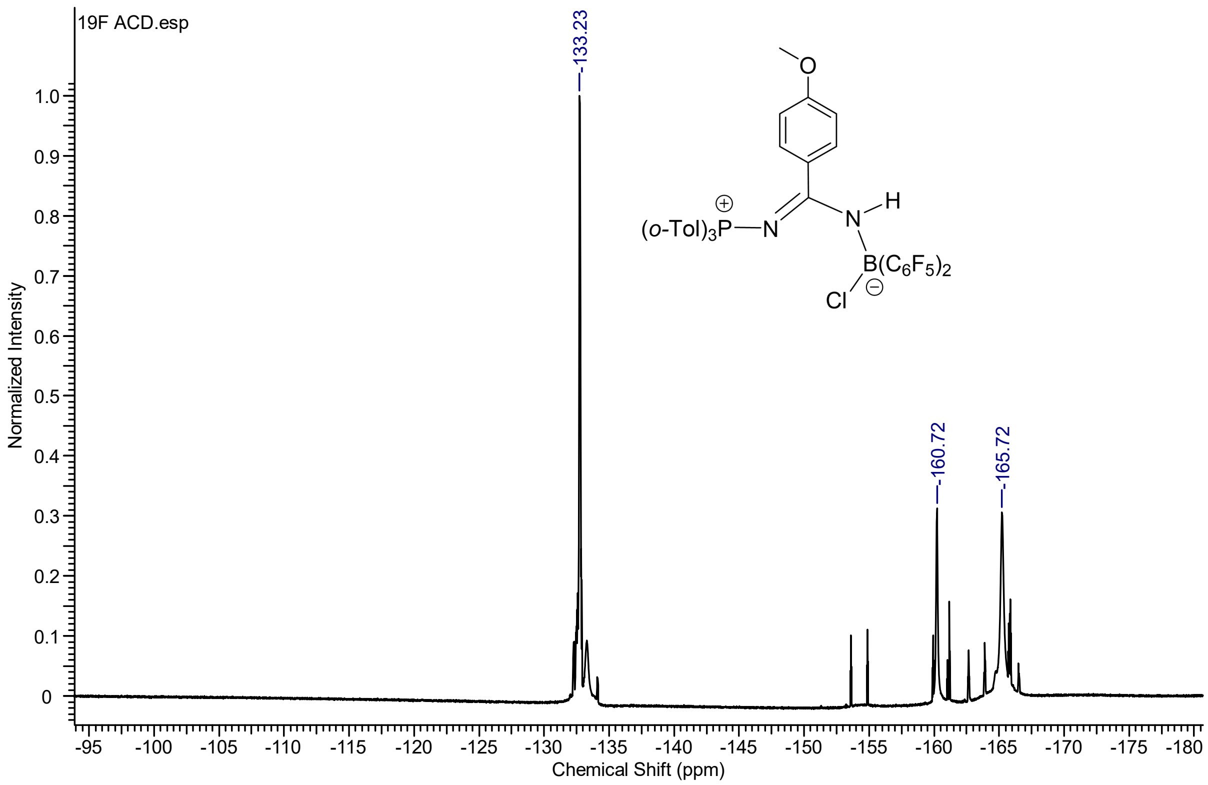

Figure S28. ^{19}**F NMR (471 MHz) spectrum of the compound 6 in** CD_2Cl_2**.**

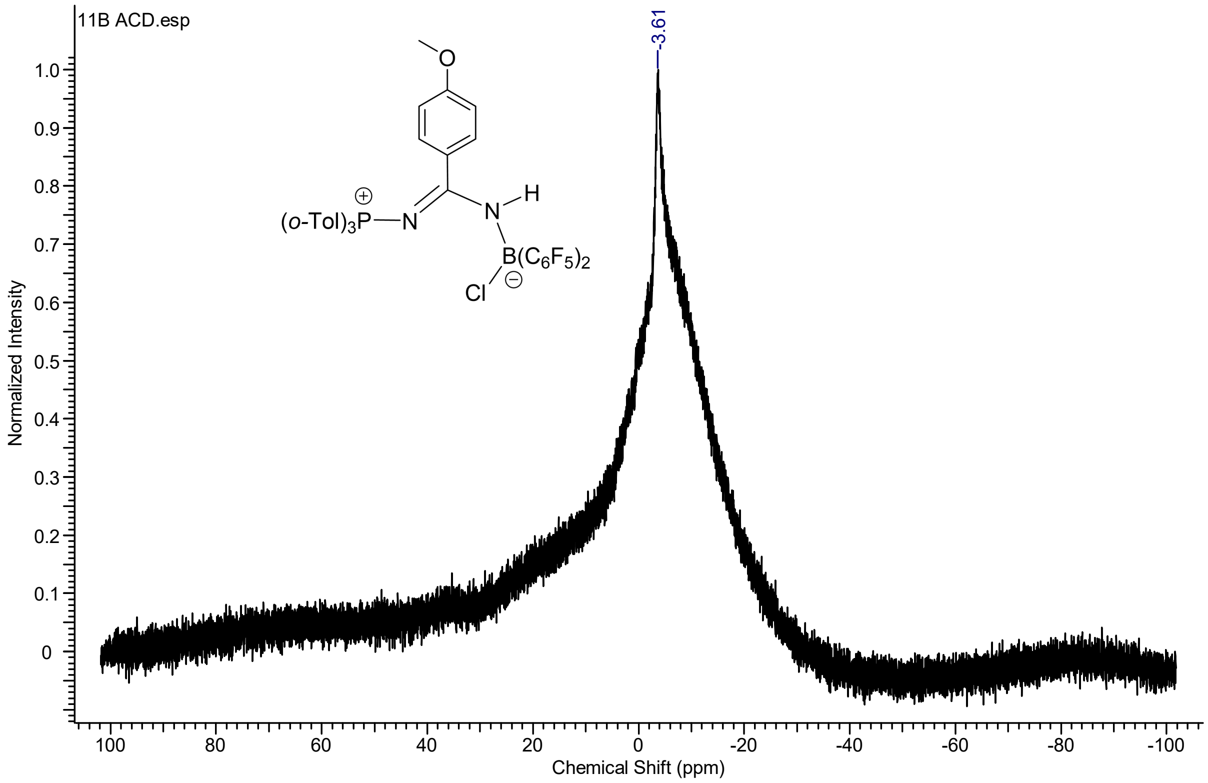

Figure S29. ^{11}**B NMR (161 MHz) spectrum of the compound 6 in** CD_2Cl_2**.**

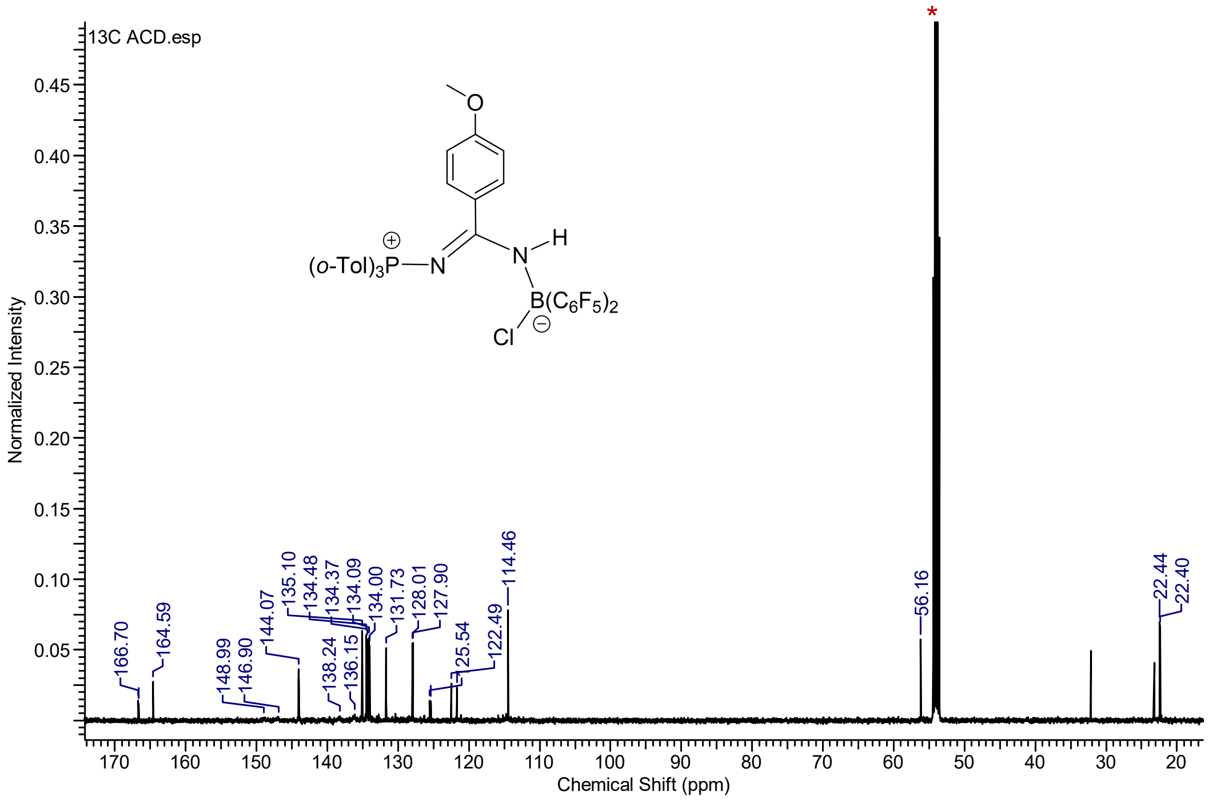

Figure S30. **^{13}C NMR (126 MHz) spectrum of the compound 6 in CD_2Cl_2 (*= CD_2Cl_2).**

Compound 7

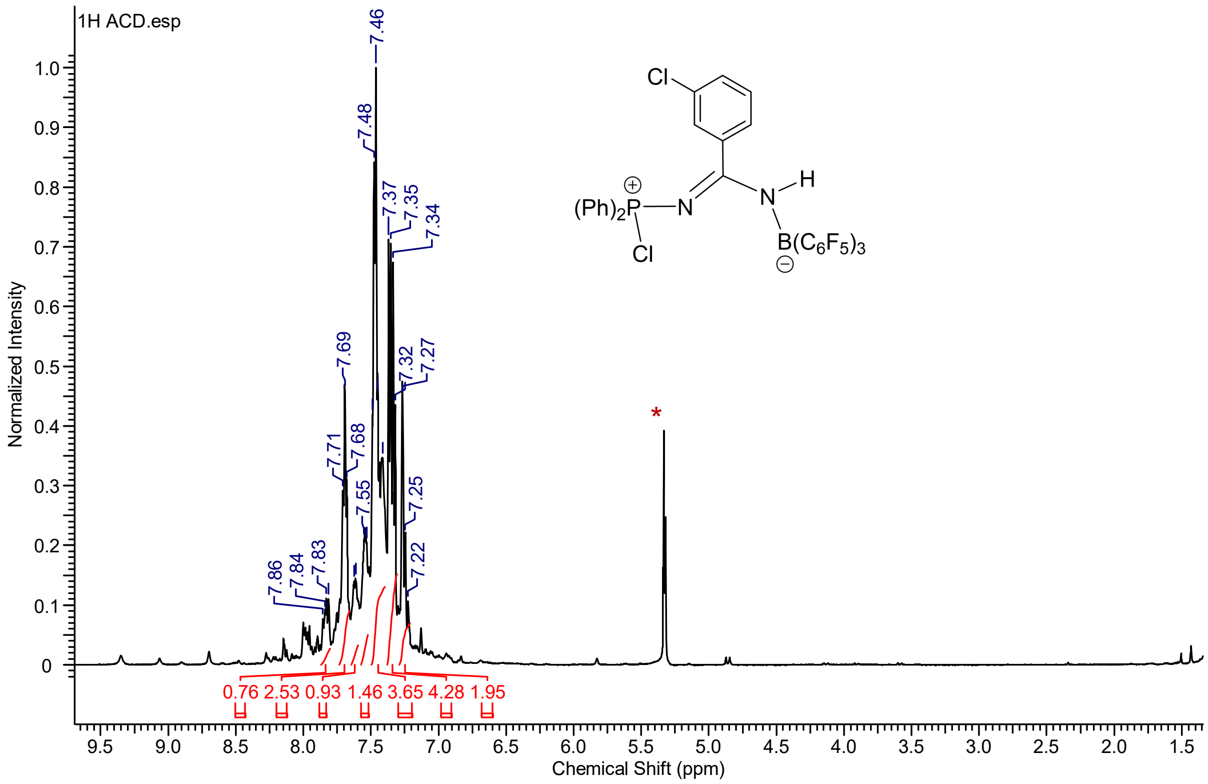

Figure S31. ^{1}H NMR (500 MHz) spectrum of the compound 7 in CD_2Cl_2 (*= CD_2Cl_2).

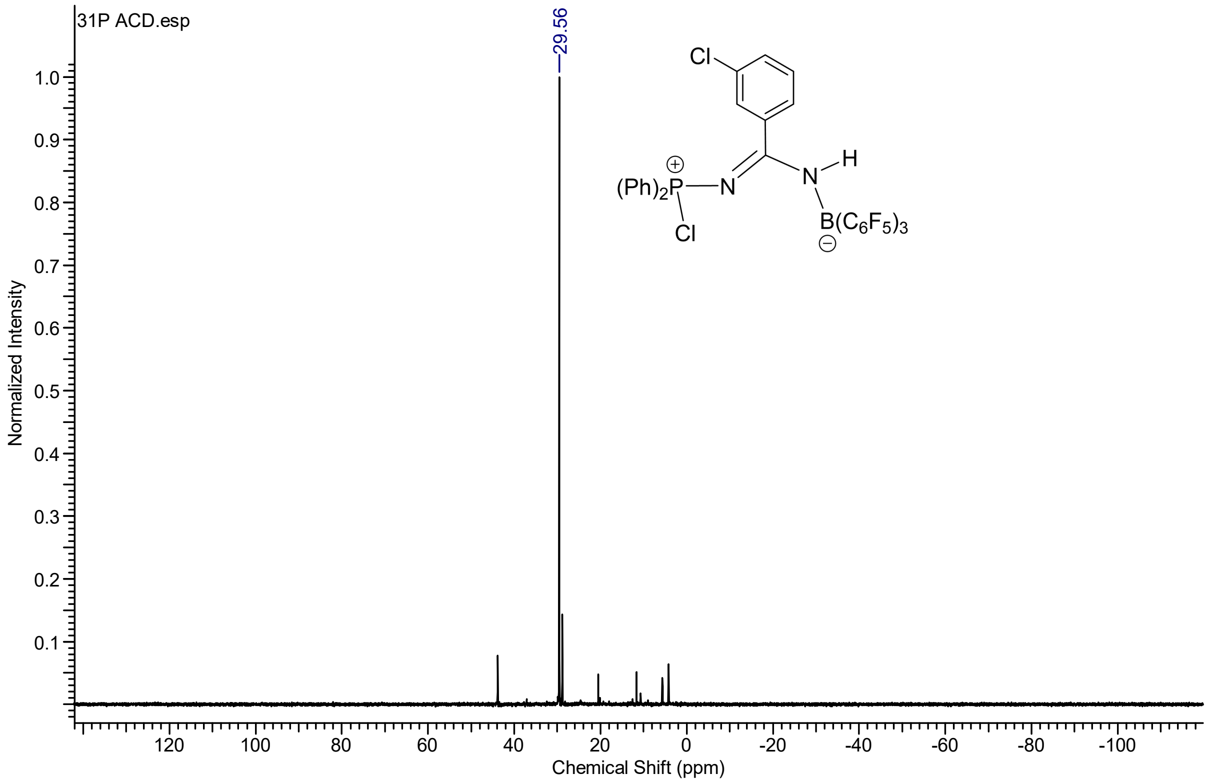

Figure S32. ^{31}P NMR (203 MHz) spectrum of the compound 7 in CD_2Cl_2.

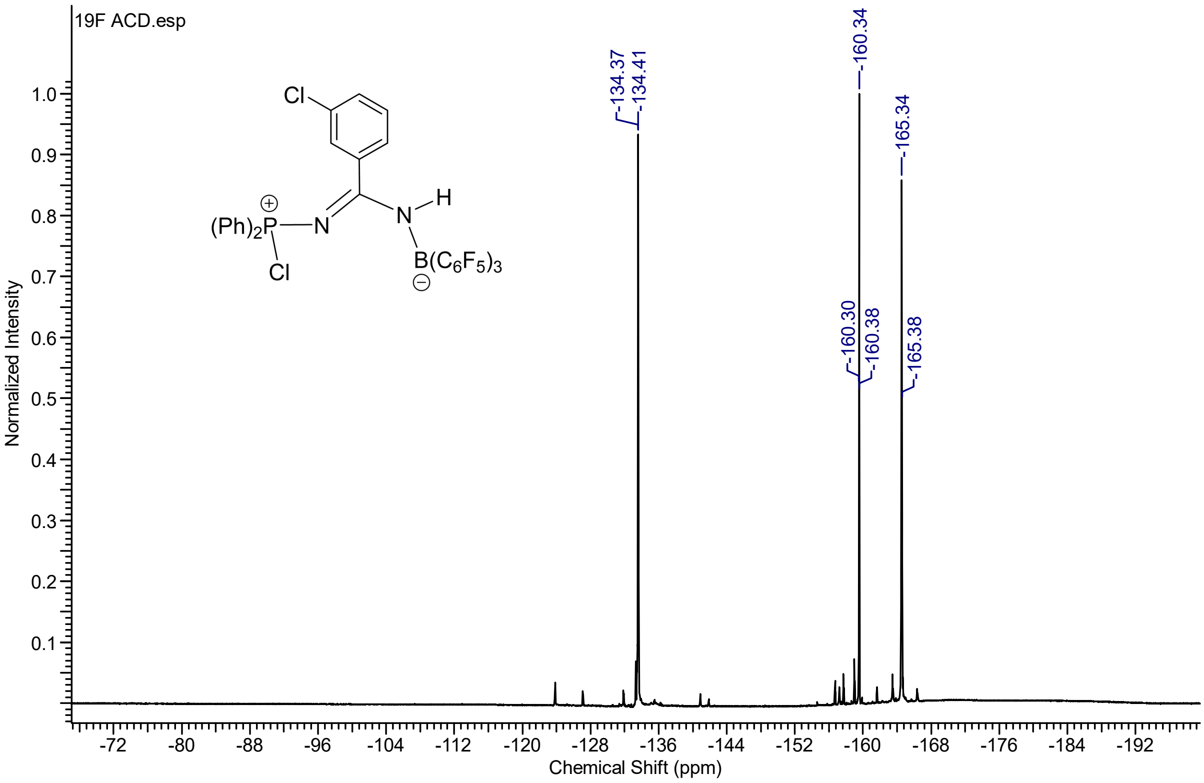

Figure S33. ^{19}**F NMR (471 MHz) spectrum of the compound 7 in** CD_2Cl_2**.**

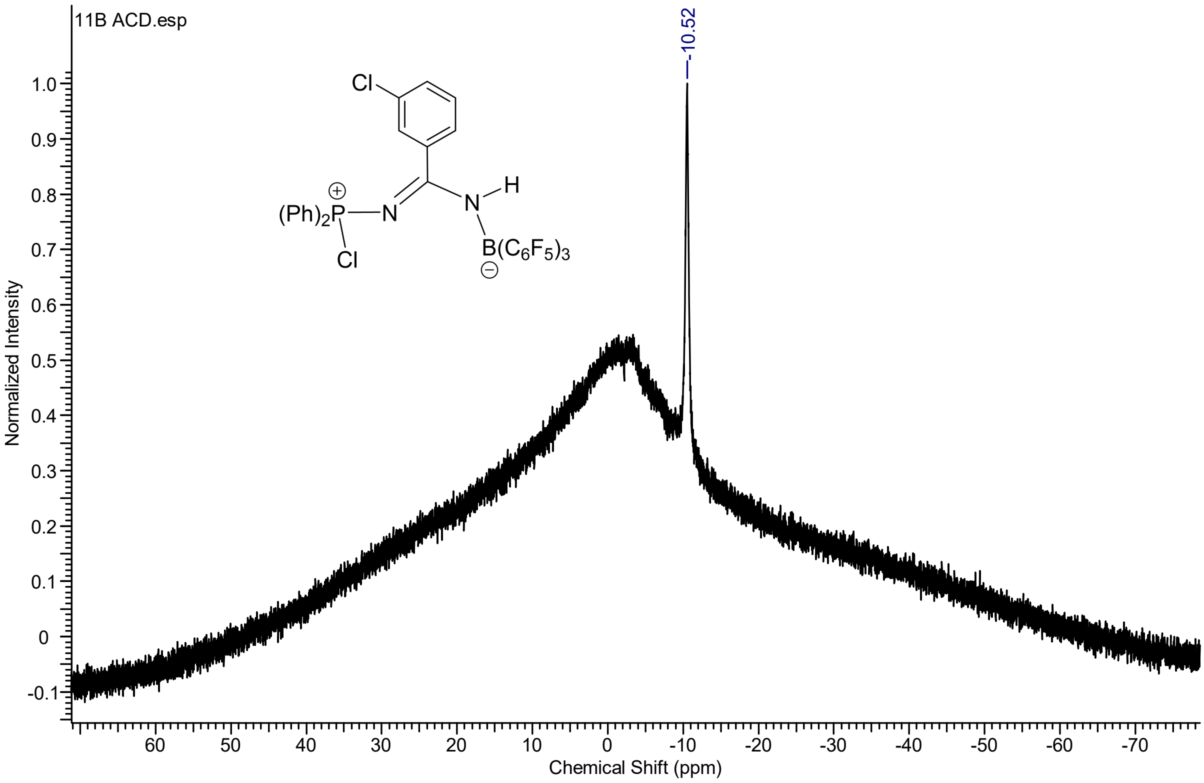

Figure S34. ^{11}**B NMR (161 MHz) spectrum of the compound 7 in** CD_2Cl_2**.**

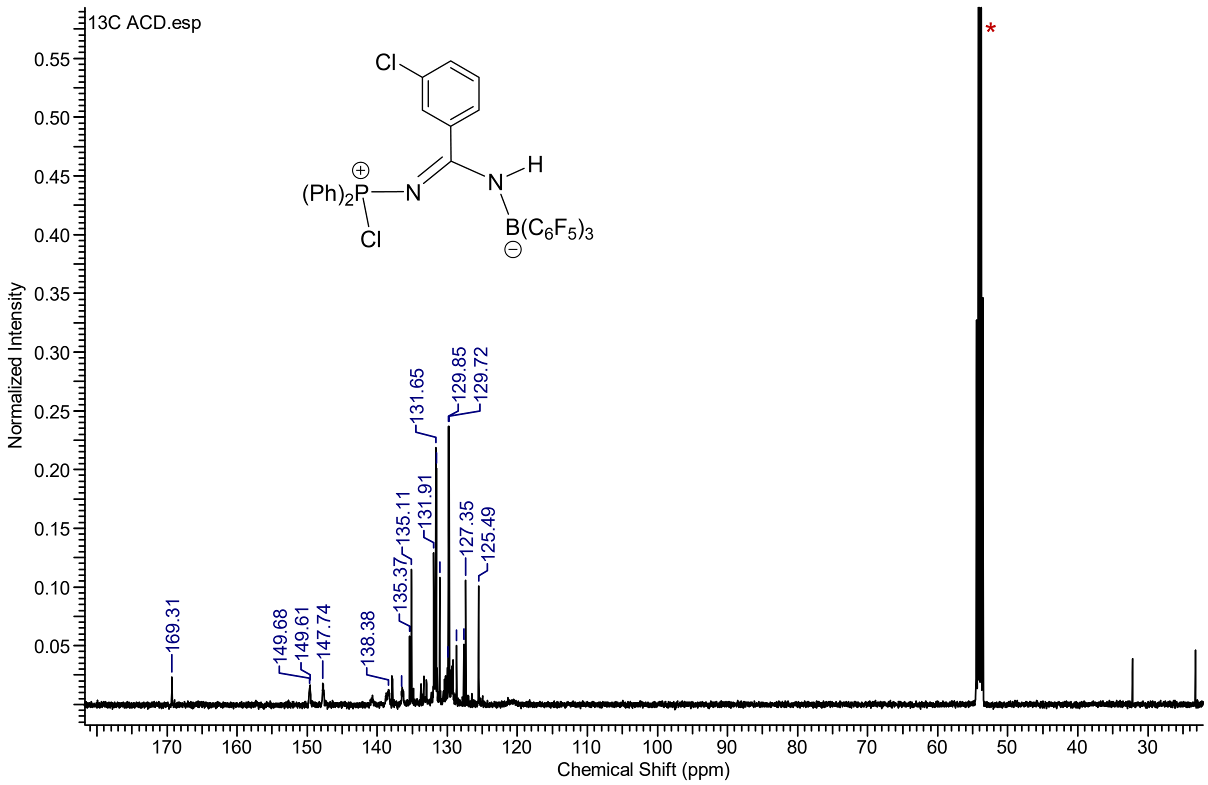

Figure S35. ^{13}C NMR (126 MHz) spectrum of the compound 7 in CD_2Cl_2 (*= CD_2Cl_2).

Compound 8

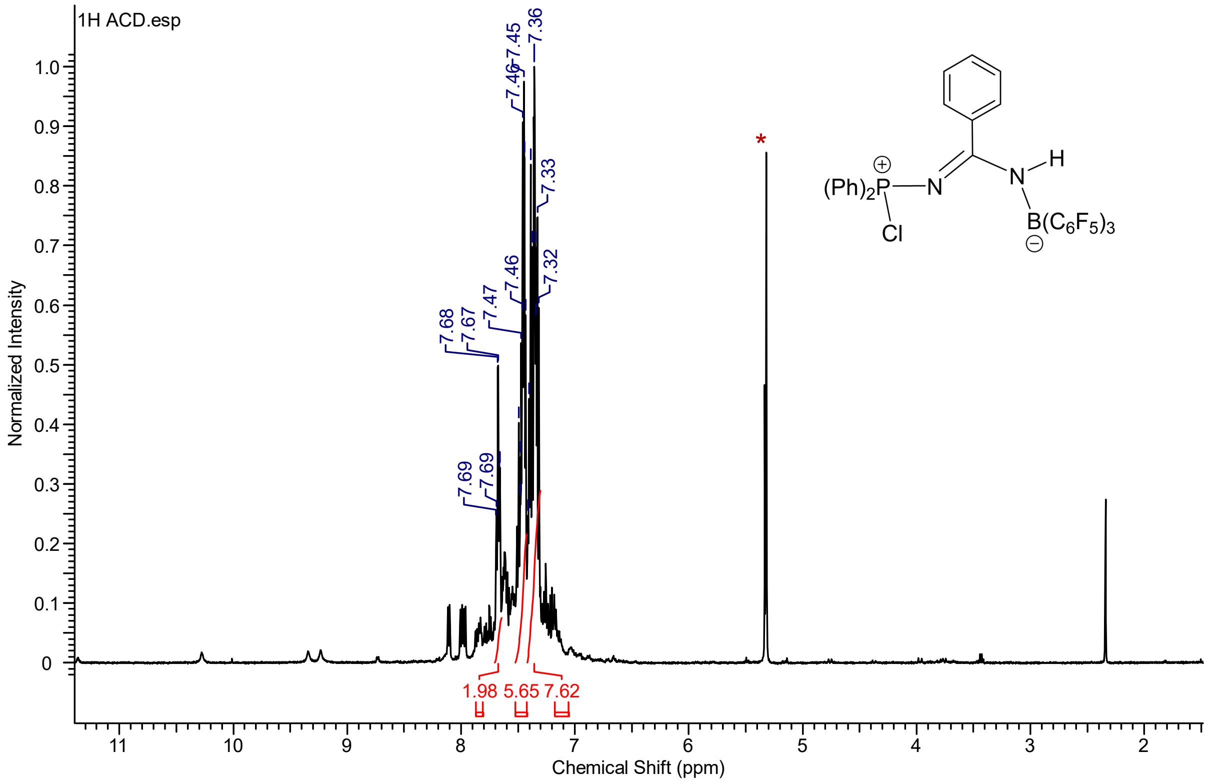

Figure S36. ^{1}H NMR (500 MHz) spectrum of the compound 8 in CD_2Cl_2 (*= CD_2Cl_2).

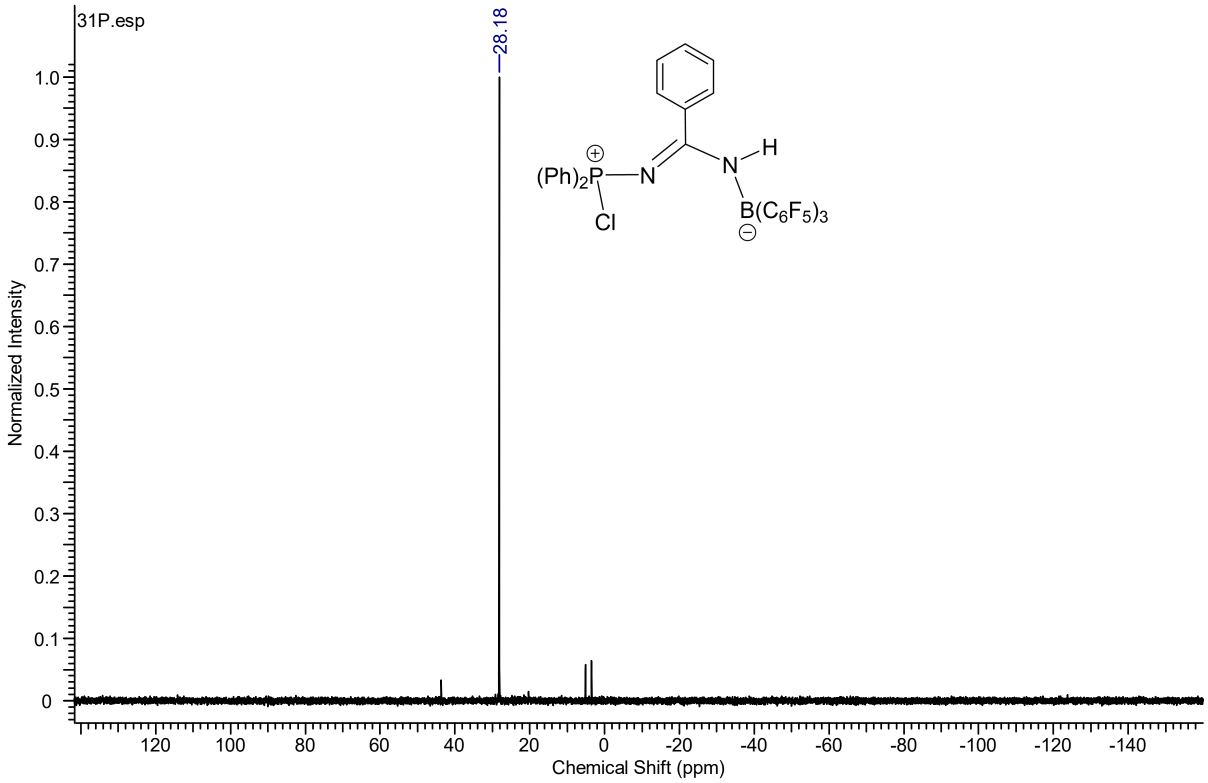

Figure S37. ^{31}P NMR (203 MHz) spectrum of the compound 8 in CD_2Cl_2.

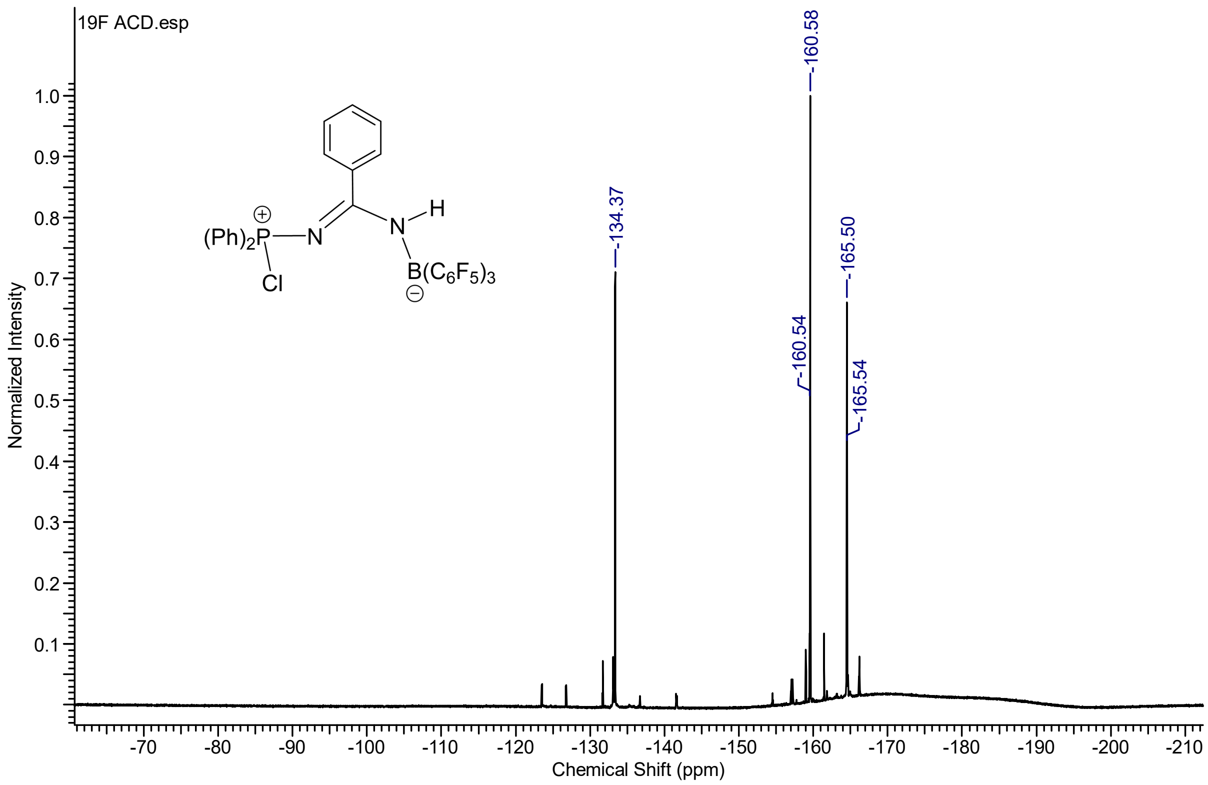

Figure S38. ^{19}**F NMR (471 MHz) spectrum of the compound 8 in** CD_2Cl_2.

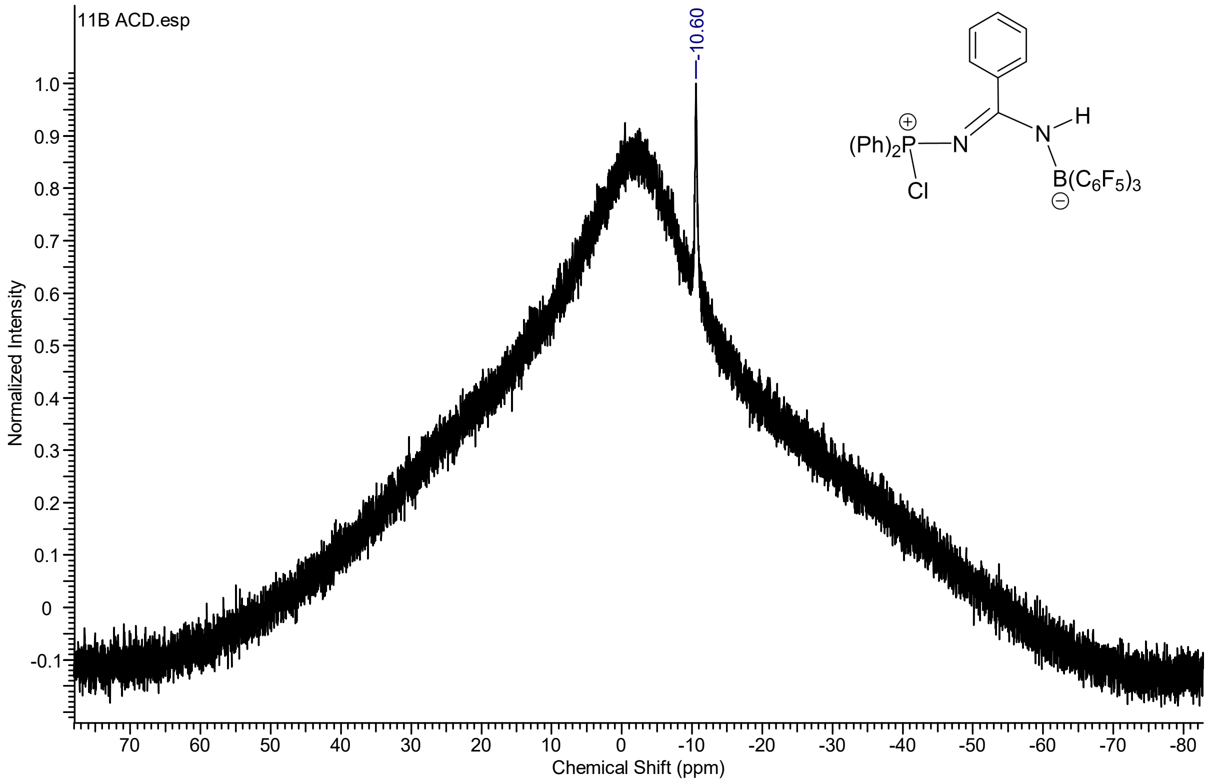

Figure S39. ^{11}**B NMR (161 MHz) spectrum of the compound 8 in** CD_2Cl_2.

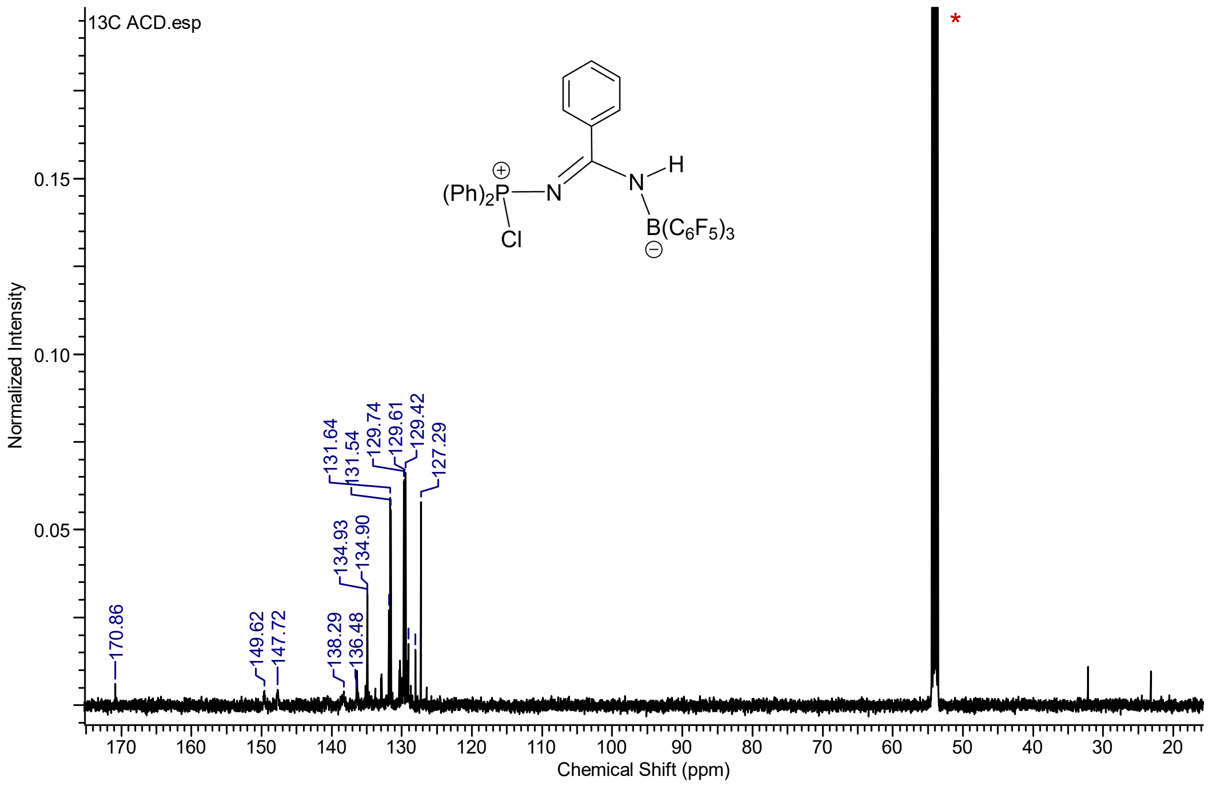

Figure S40. **^{13}C NMR (126 MHz) spectrum of the compound 8 in CD_2Cl_2 (*= CD_2Cl_2).**

Compound 9

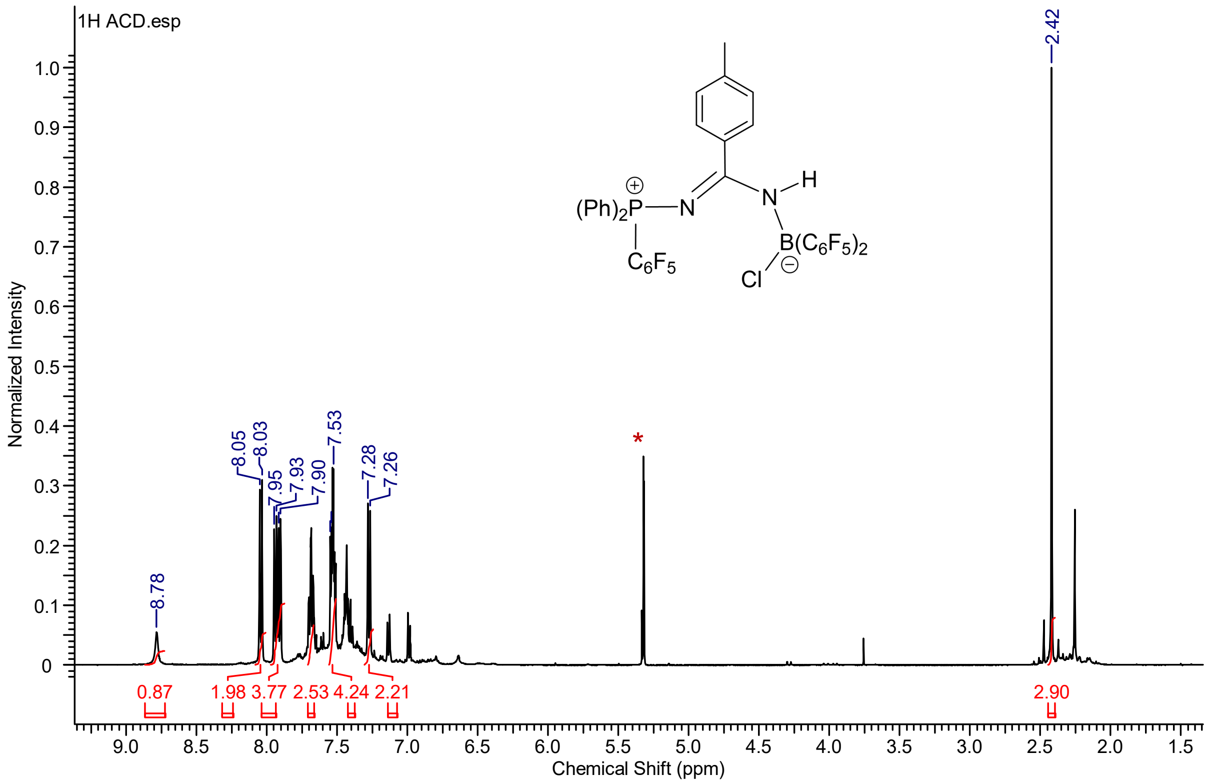

Figure S41. **^{1}H NMR (500 MHz) spectrum of the compound 9 in CD_2Cl_2 (*= CD_2Cl_2).**

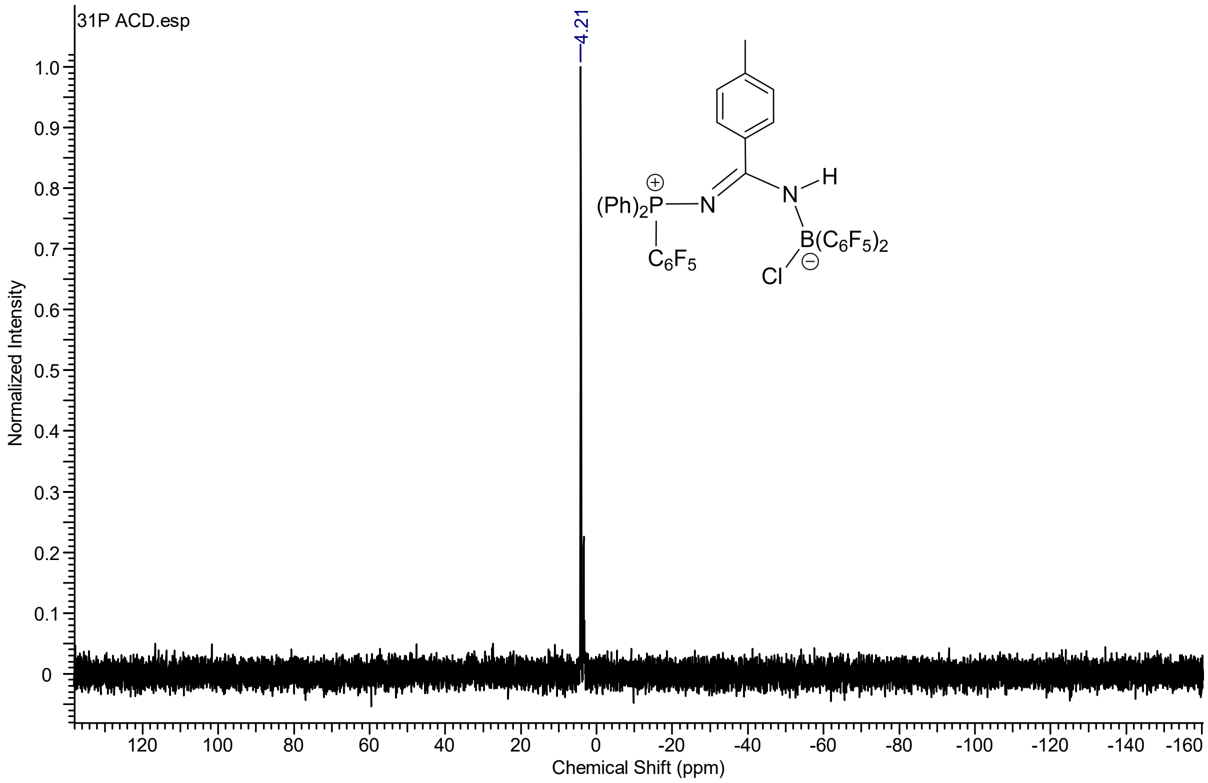

Figure S42. **^{31}P NMR (203 MHz) spectrum of the compound 9 in CD_2Cl_2.**

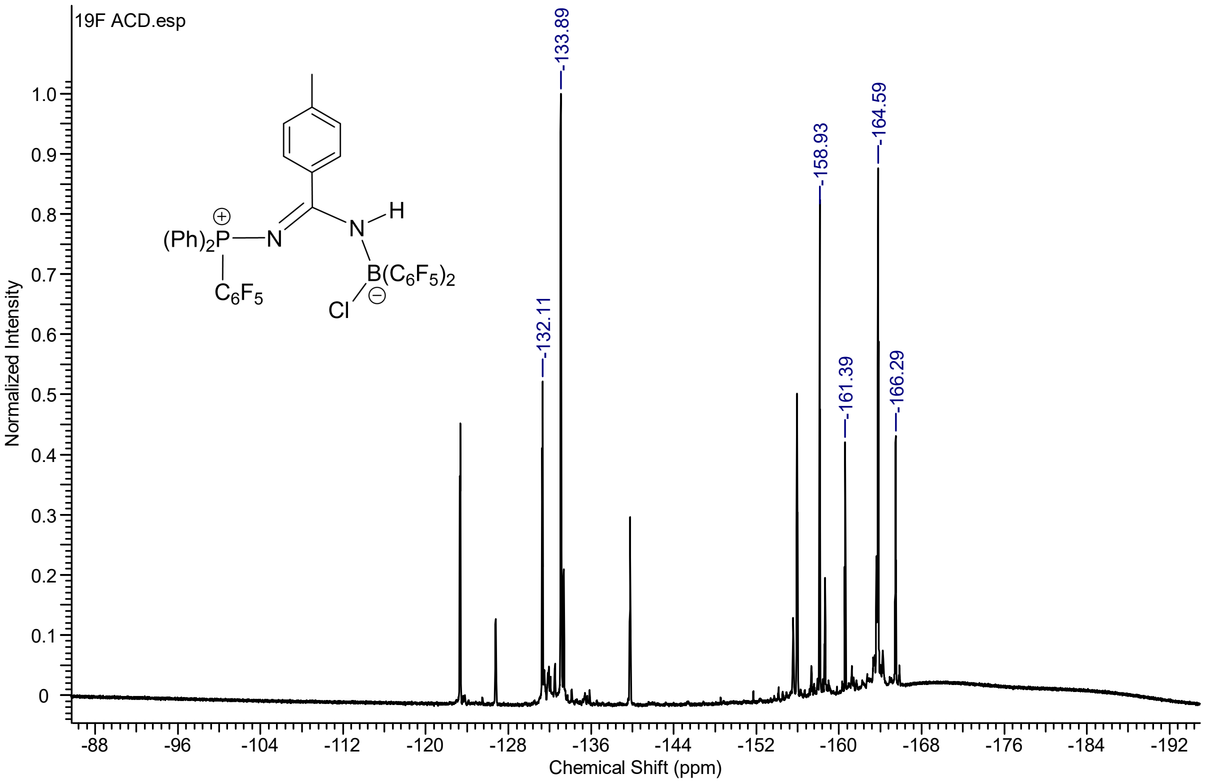

Figure S43. ^{19}**F NMR (471 MHz) spectrum of the compound 9 in** CD_2Cl_2**.**

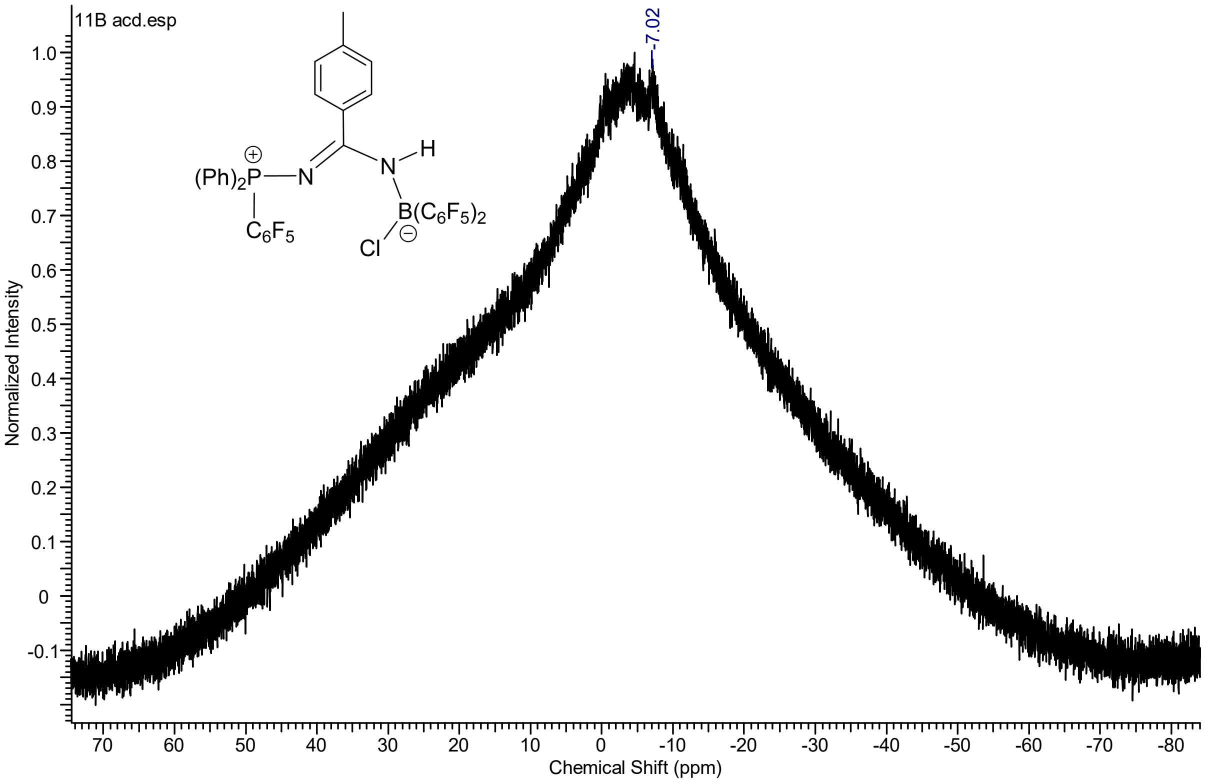

Figure S44. ^{11}**B NMR (161 MHz) spectrum of the compound 9 in** CD_2Cl_2**.**

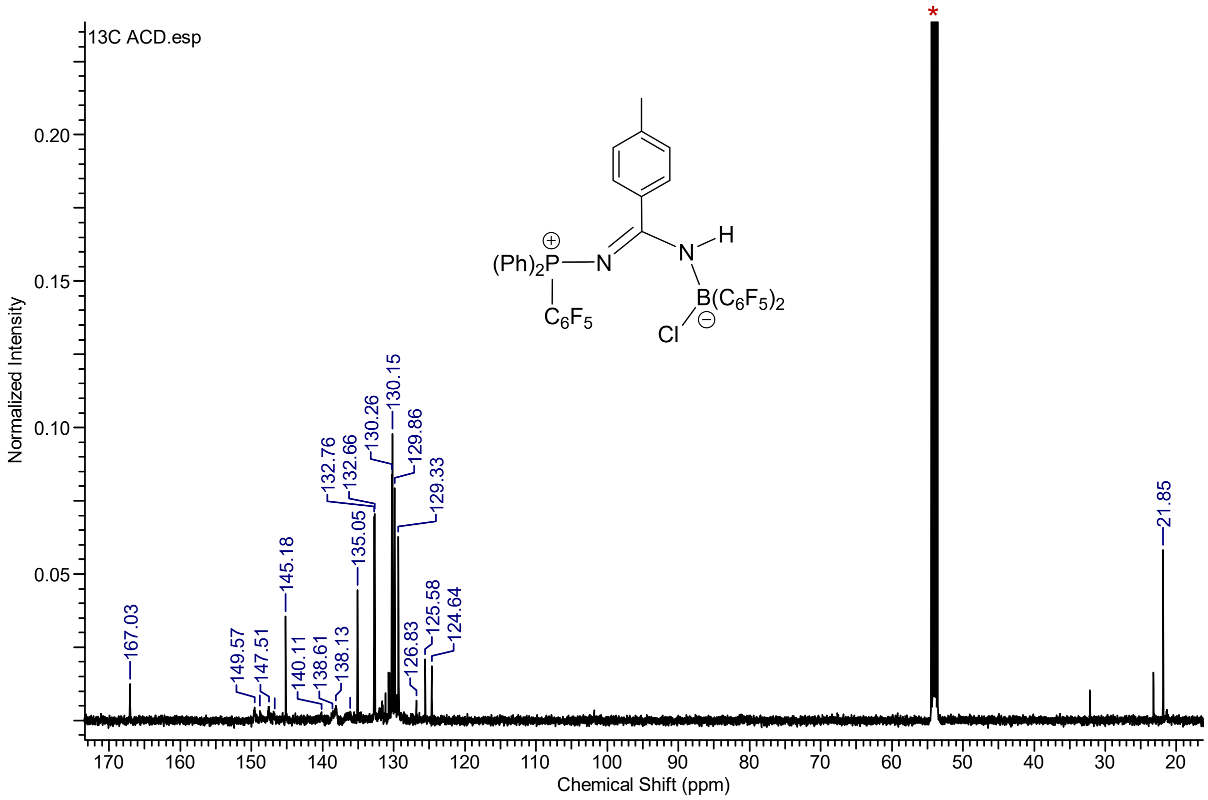

Figure S45. ^{13}**C NMR (126 MHz) spectrum of the compound 9 in** CD_2Cl_2 **(*=** CD_2Cl_2**).**

Compound 10

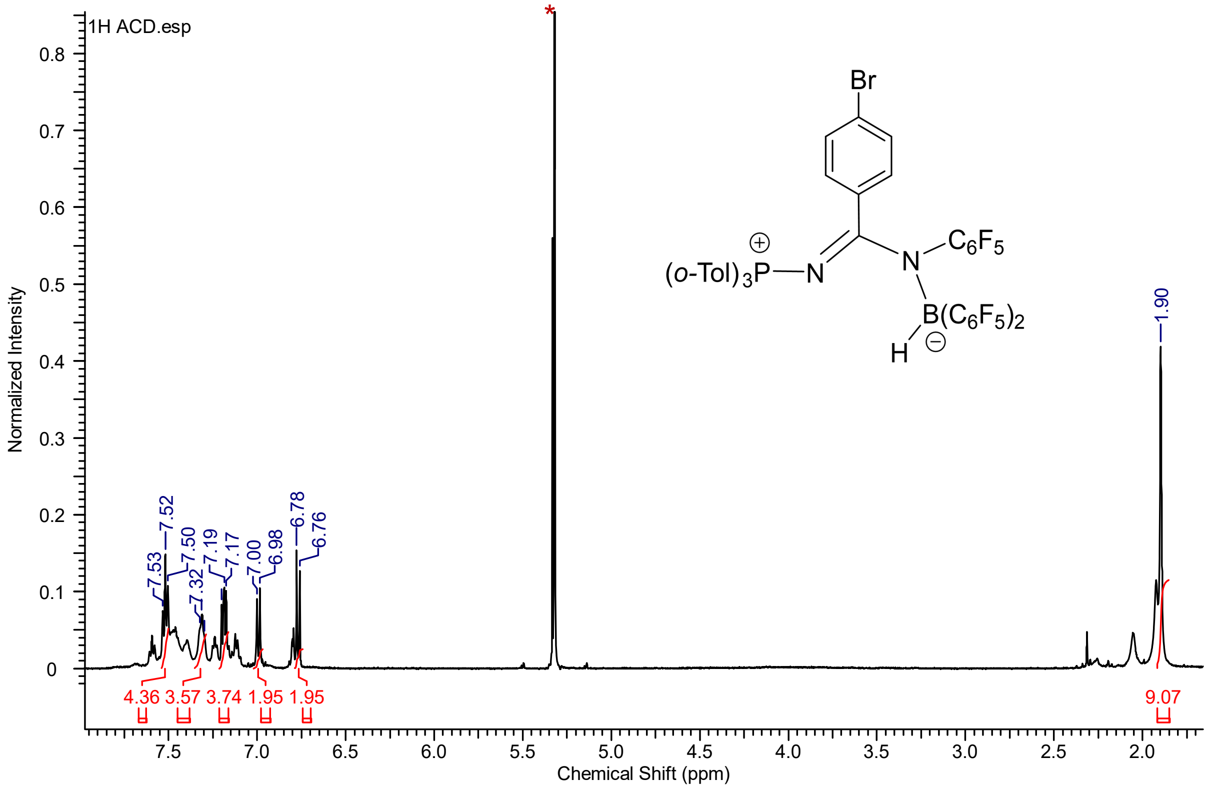

Figure S46. ^{1}**H NMR (500 MHz) spectrum of the compound 10 in CD_2Cl_2 (*= CD_2Cl_2).**

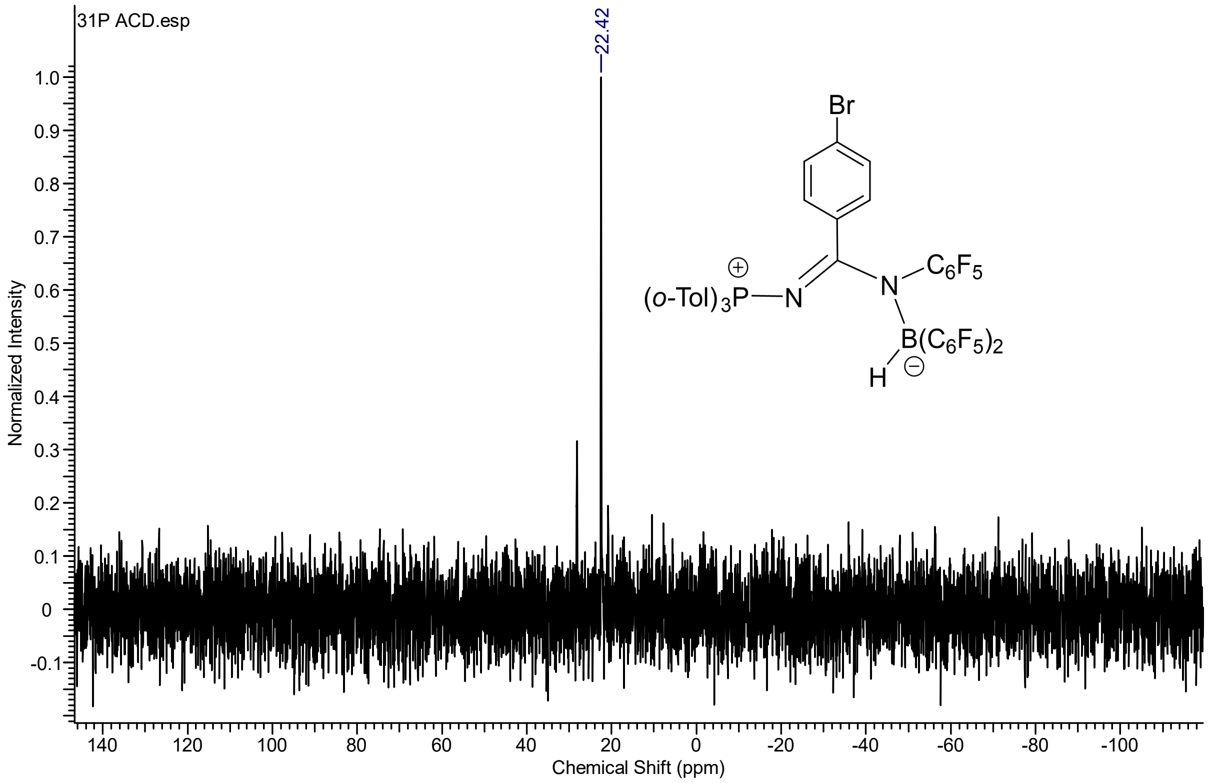

Figure S47. ^{31}**P NMR (203 MHz) spectrum of the compound 10 in CD_2Cl_2.**

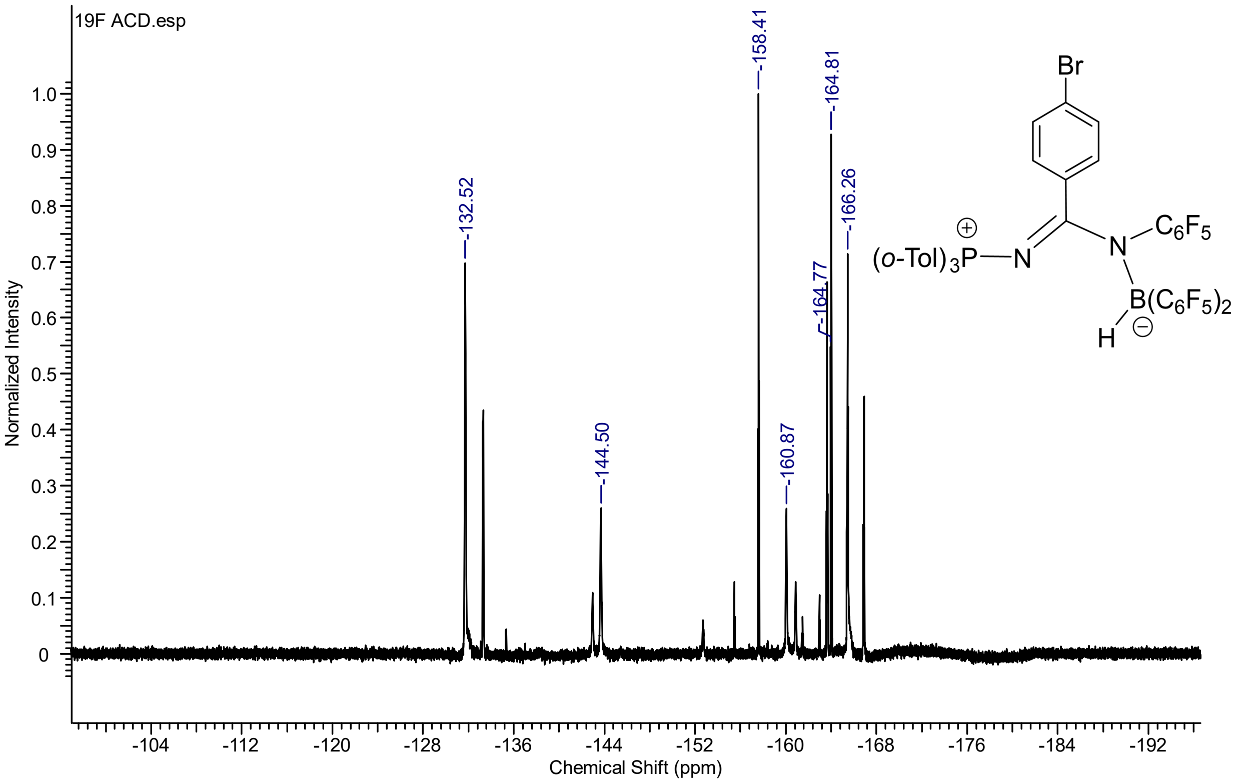

Figure S48. **^{19}F NMR (471 MHz) spectrum of the compound 10 in CD_2Cl_2.**

Compound 11

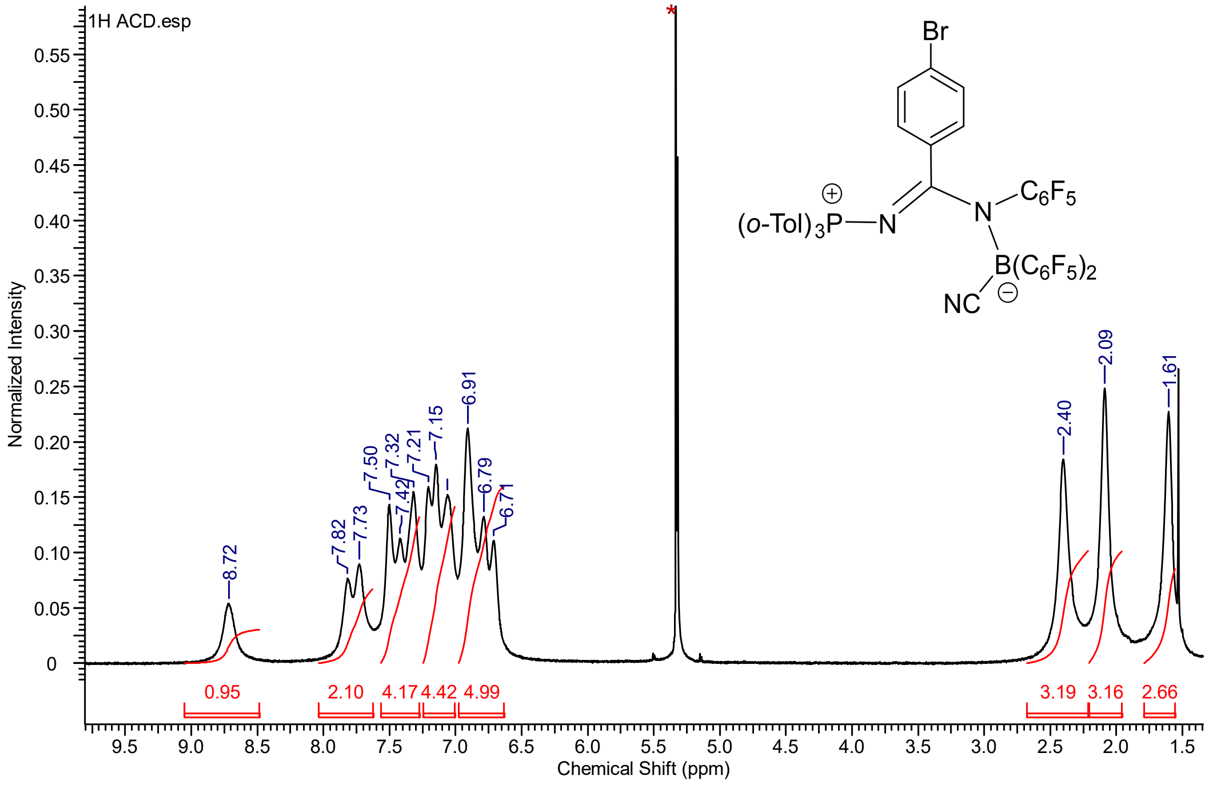

Figure S49. ^{1}**H NMR (500 MHz) spectrum of the compound 11 in** CD_2Cl_2 **(*=** CD_2Cl_2**).**

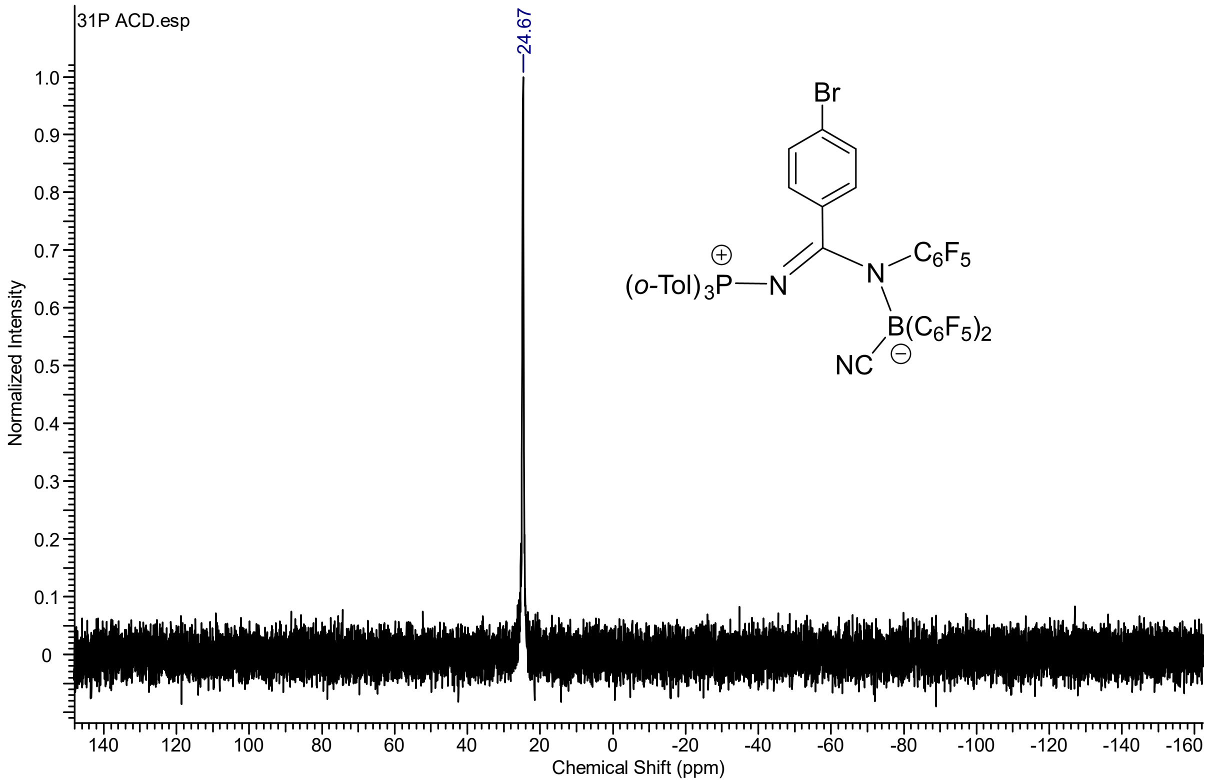

Figure S50. ^{31}**P NMR (203 MHz) spectrum of the compound 11 in** CD_2Cl_2**.**

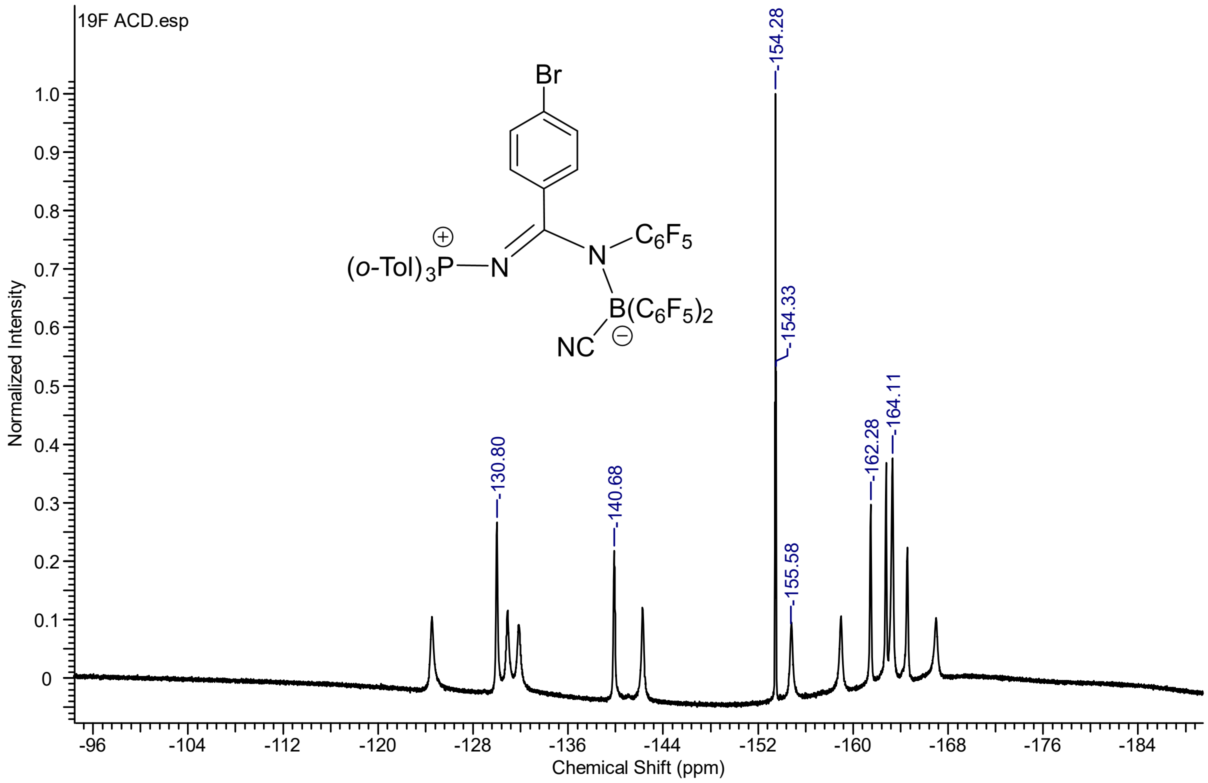

Figure S51. **^{19}F NMR (471 MHz) spectrum of the compound 11 in CD_2Cl_2.**

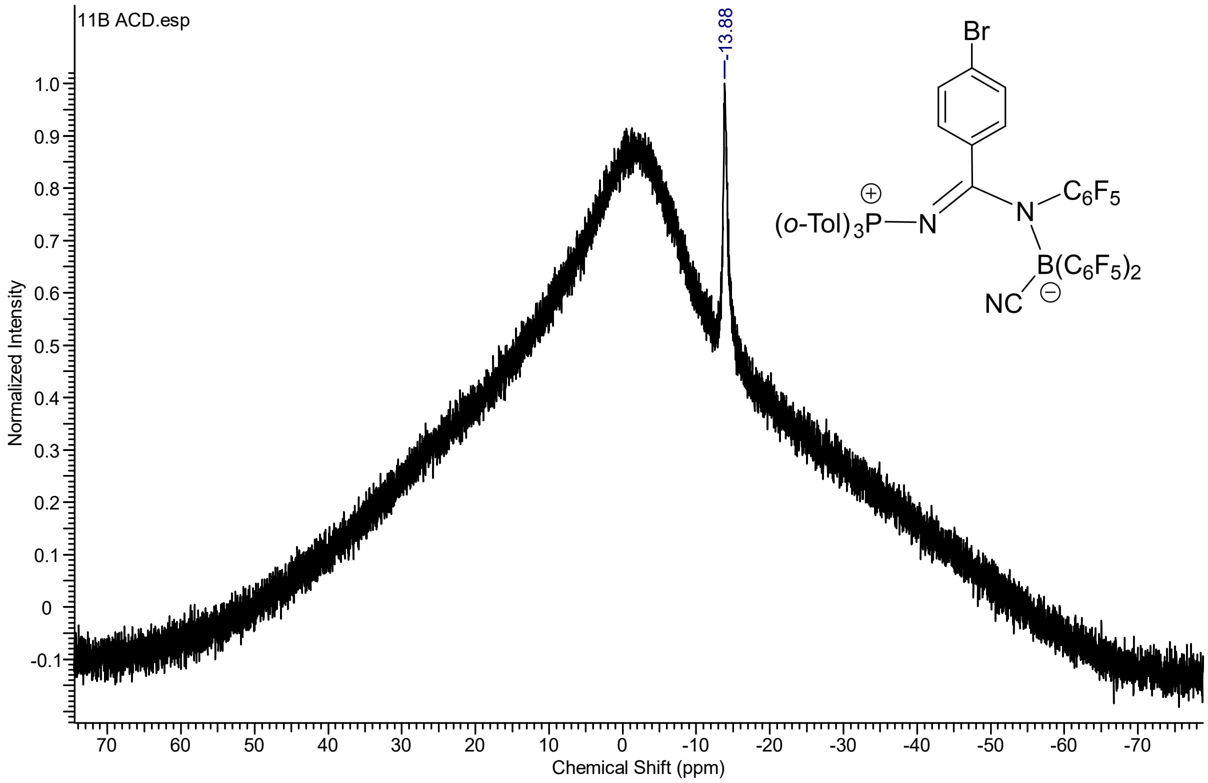

Figure S52. **^{11}B NMR (161 MHz) spectrum of the compound 11 in CD_2Cl_2.**

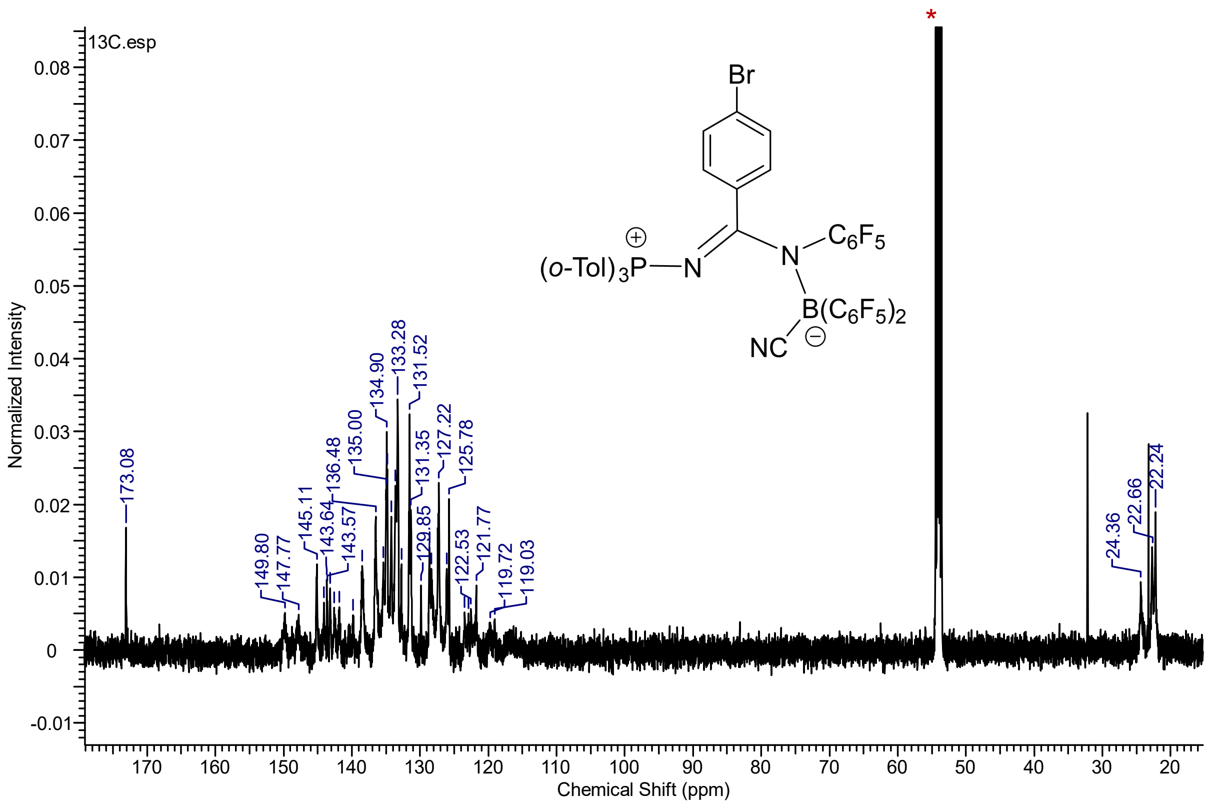

Figure S53. **^{13}C NMR (126 MHz) spectrum of the compound 11 in CD_2Cl_2 (*= CD_2Cl_2).**

Compound 12

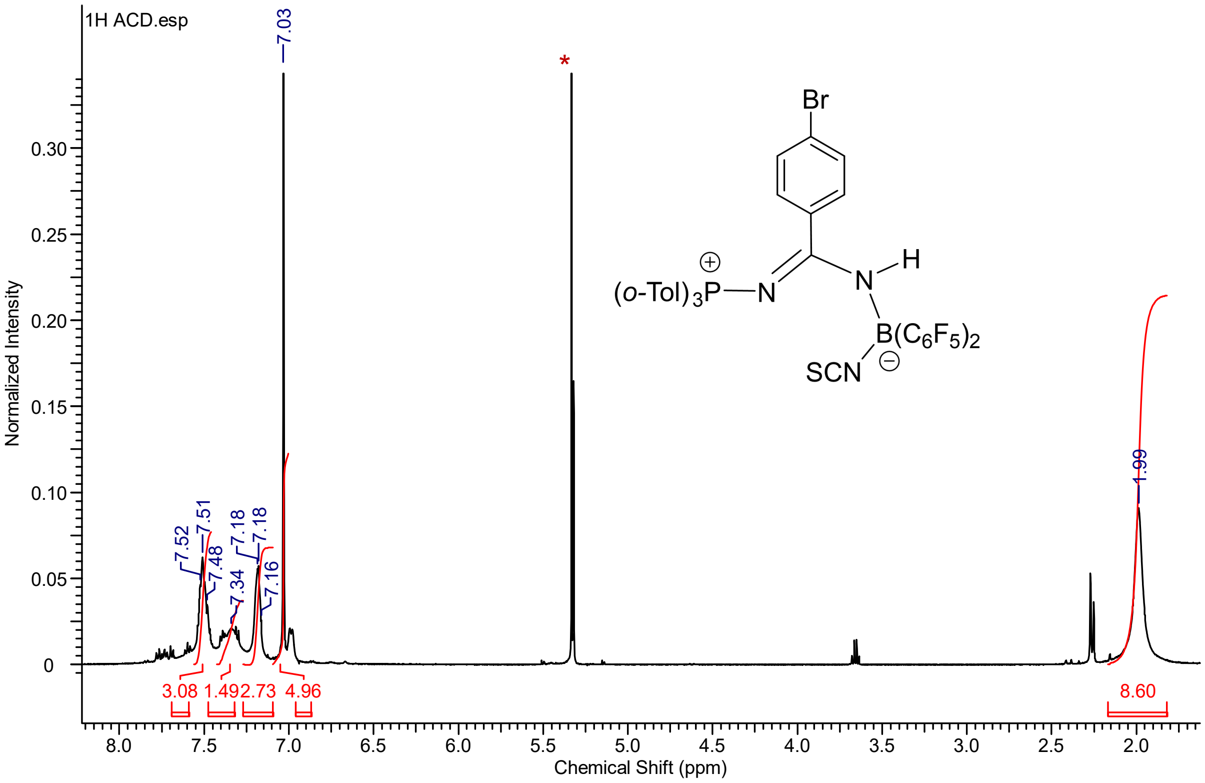

Figure S54. **^{1}H NMR (500 MHz) spectrum of the compound 12 in CD_2Cl_2 (*= CD_2Cl_2).**

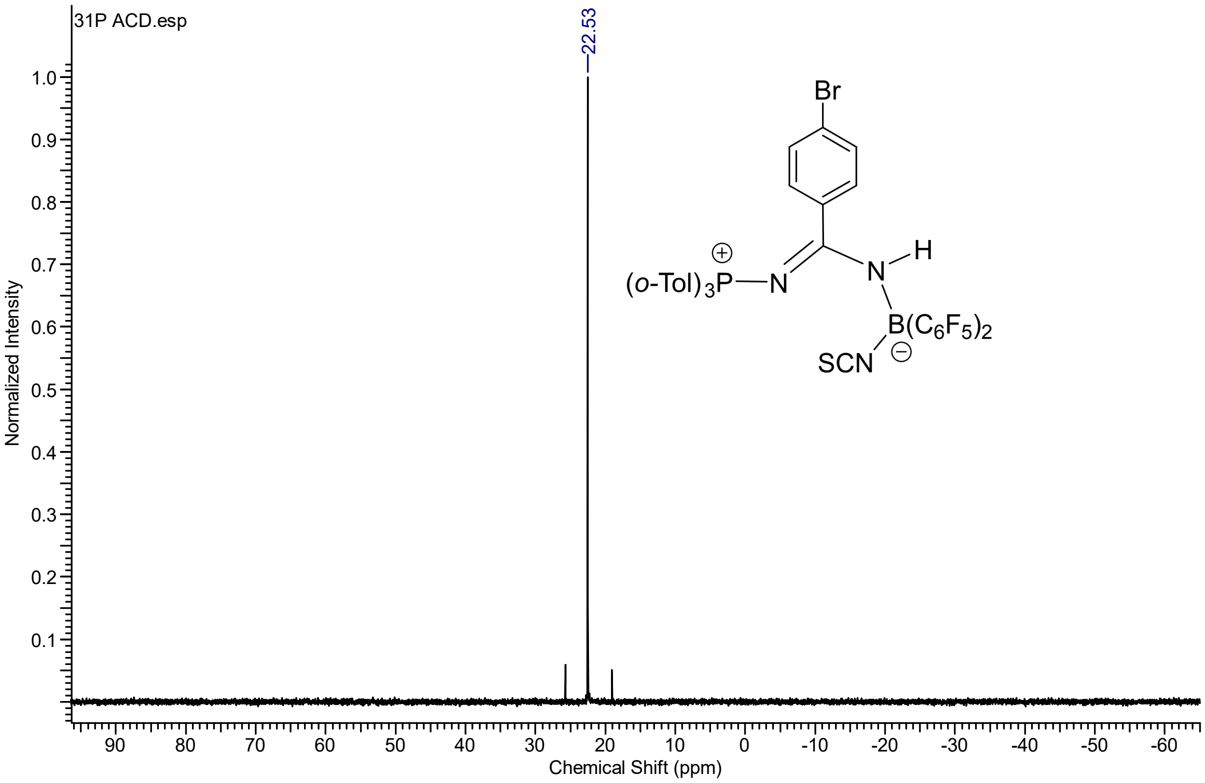

Figure S55. **^{31}P NMR (203 MHz) spectrum of the compound 12 in CD_2Cl_2.**

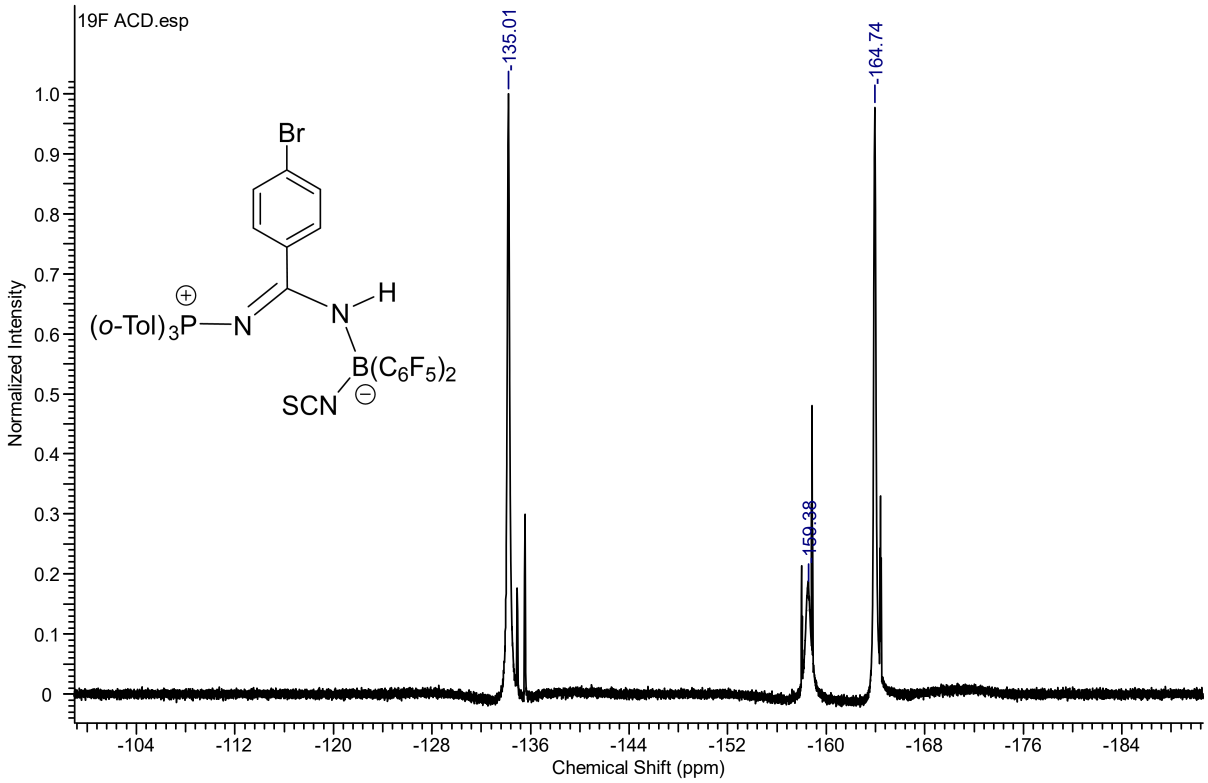

Figure S56. ^{19}F NMR (471 MHz) spectrum of the compound 12 in CD_2Cl_2.

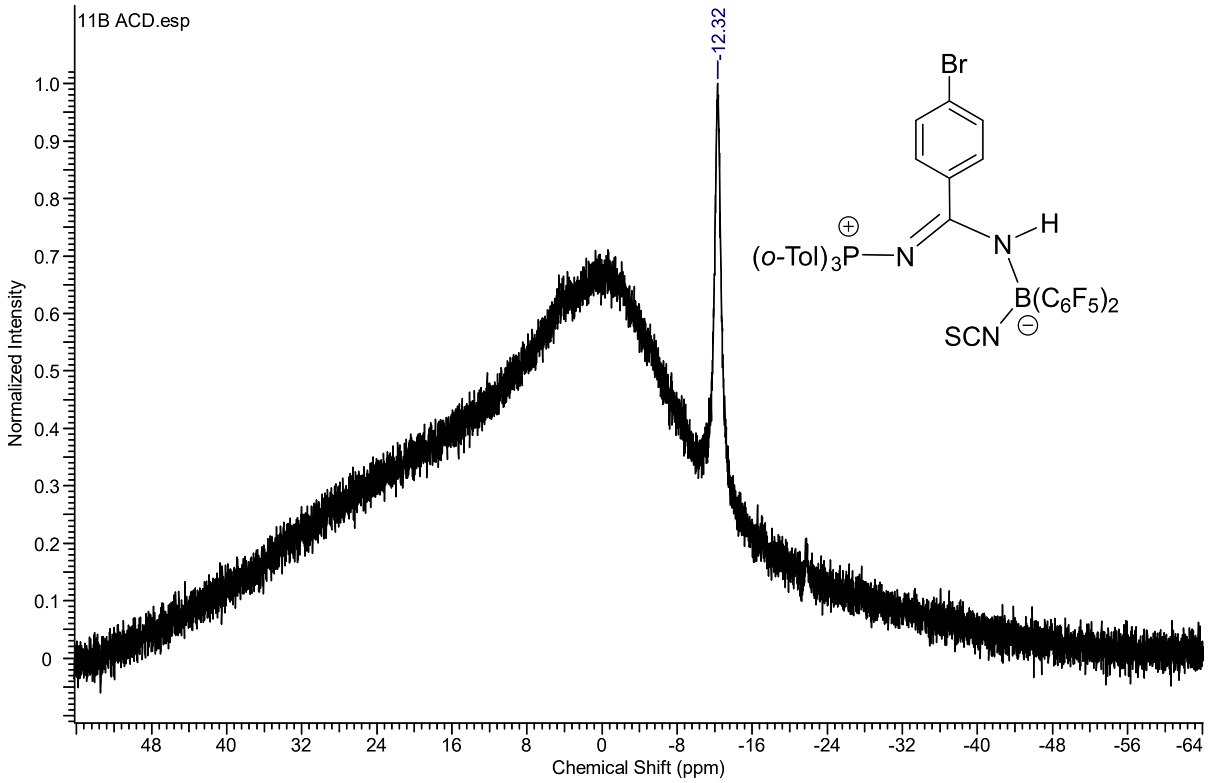

Figure S57. ^{11}B NMR (161 MHz) spectrum of the compound 12 in CD_2Cl_2.

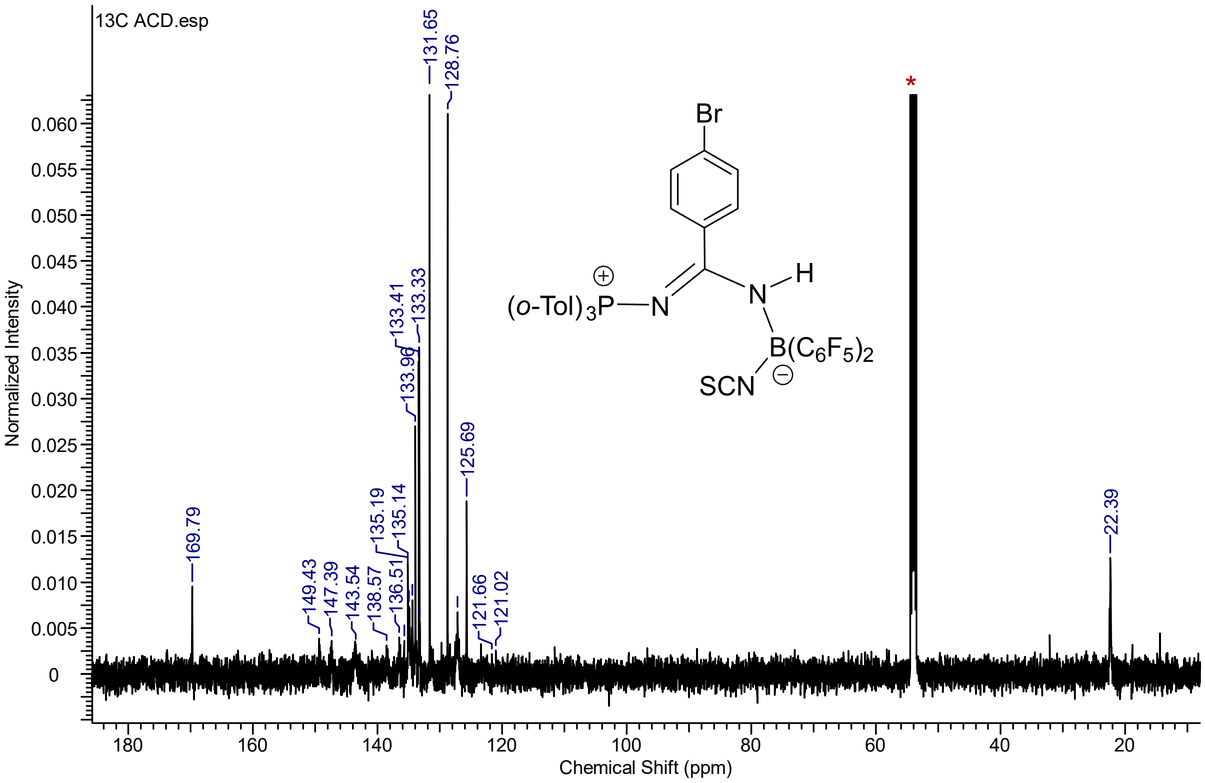

Figure S58. **^{13}C NMR (126 MHz) spectrum of the compound 12 in CD_2Cl_2 (*= CD_2Cl_2).**

Control reactions

3-(4-Bromophenyl)-3-chloro-diazirine and $B(C_6F_5)_3$

Into a J Young NMR tube, $B(C_6F_5)_3$ (51mg, 0.10 mmol, 1.0 equiv.) was taken in DCM (0.5 mL). After addition of a solution of 3-(4-bromophenyl)-3-chloro-diazirine (23mg, 0.1 mmol, 1.0 equiv.) in DCM (0.5 mL), the reaction mixture was allowed to left at RT for 24 h. An immediate bubble observed during addition. The mixture was analyzed by NMR spectroscopy.

3-(4-Bromophenyl)-3-chloro-diazirine and $HB(C_6F_5)_2$

Into a J Young NMR tube, $HB(C_6F_5)_2$ (35mg, 0.10 mmol, 1.0 equiv.) was taken in DCM (0.5 mL). After addition of a solution of 3-(4-bromophenyl)-3-chloro-diazirine (23mg, 0.1 mmol, 1.0 equiv.) in DCM (0.5 mL), the reaction mixture was allowed to left at RT for 24 h. An immediate strong bubble formed during addition. The mixture was analyzed by NMR spectroscopy.

3-(4-Bromophenyl)-3-chloro-diazirine and $P(o\text{-Tol})_3$

Into a J Young NMR tube, P(*o*-Tol)$_3$ (30 mg, 0.1 mmol, 1.0 equiv.) was taken in DCM (0.5 mL). After addition of a solution of 3-(4-bromophenyl)-3-chloro-diazirine (23mg, 0.1 mmol, 1.0 equiv.) in DCM (0.5 mL), the reaction mixture was allowed to left at RT for 24 h. The mixture was analyzed by NMR spectroscopy.

3-(4-Bromophenyl)-3-chloro-diazirine and $HP(Ph)_2$

Into a J Young NMR tube, $HP(Ph)_2$ (18mg, 0.1 mmol, 1.0 equiv.) was taken in DCM (0.5 mL). After addition of a solution of 3-(4-bromophenyl)-3-chloro-diazirine (23mg, 0.1 mmol, 1.0 equiv.) in DCM (0.5 mL), the reaction mixture was allowed to left at RT for 24 h. The mixture was analyzed by NMR spectroscopy.

Figures for control reactions

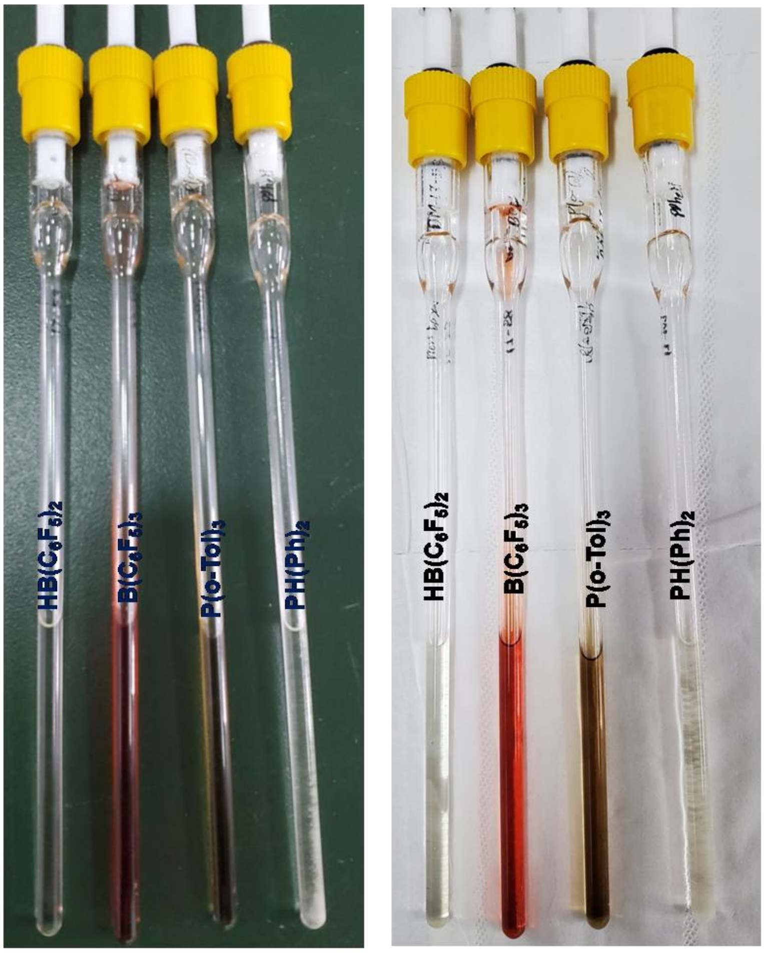

Different background for clarity

Figure S59. Image for control reactions after 24 h at RT.

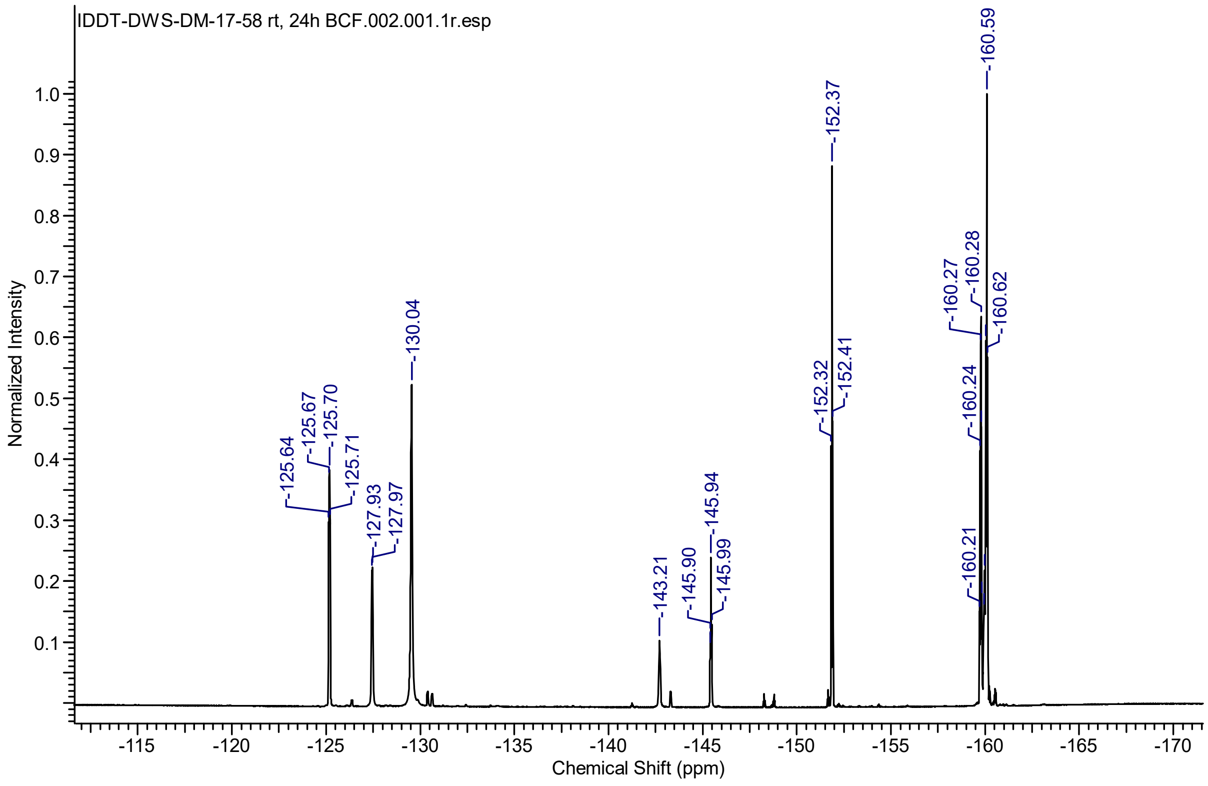

Figure S60.[19]**F NMR (471 MHz) spectrum of the crude mixture of 3-(4-bromophenyl)-3-chloro-diazirine and B(C_6F_5)$_3$ after 24 h at RT.**

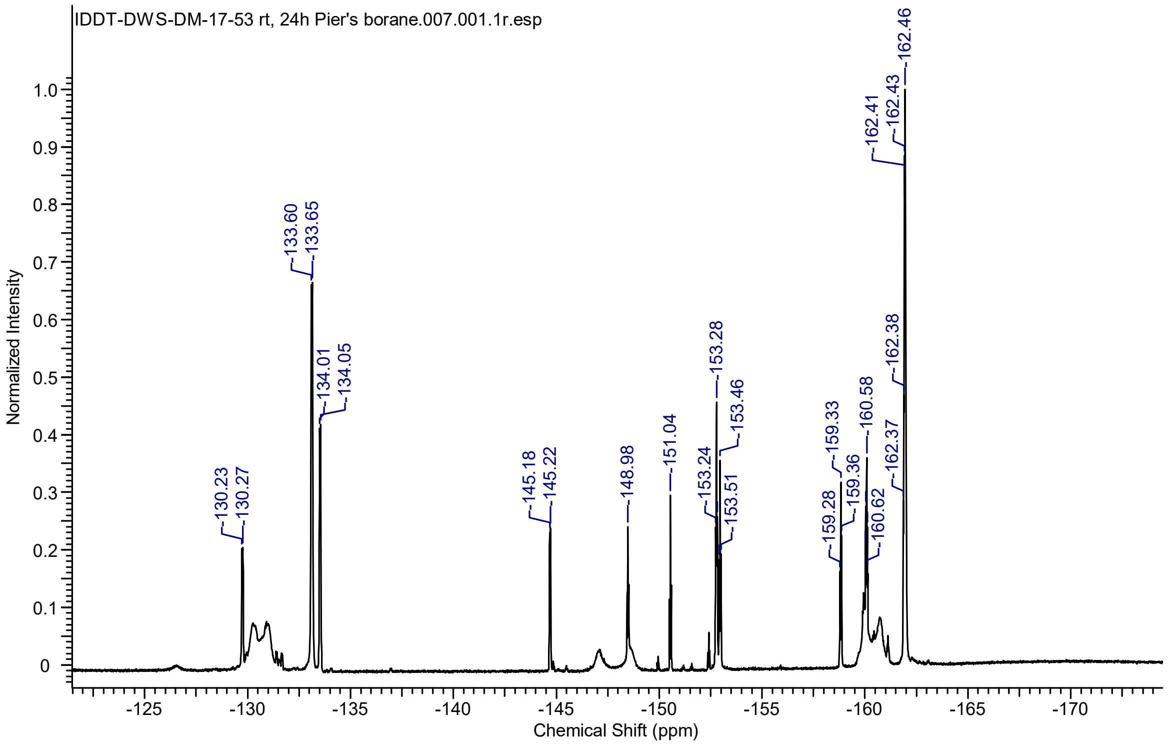

Figure S61.[19]**F NMR (471 MHz) spectrum of the crudemixture of 3-(4-bromophenyl)-3-chloro-diazirine and HB(C_6F_5)$_2$ after 24 h at RT.**

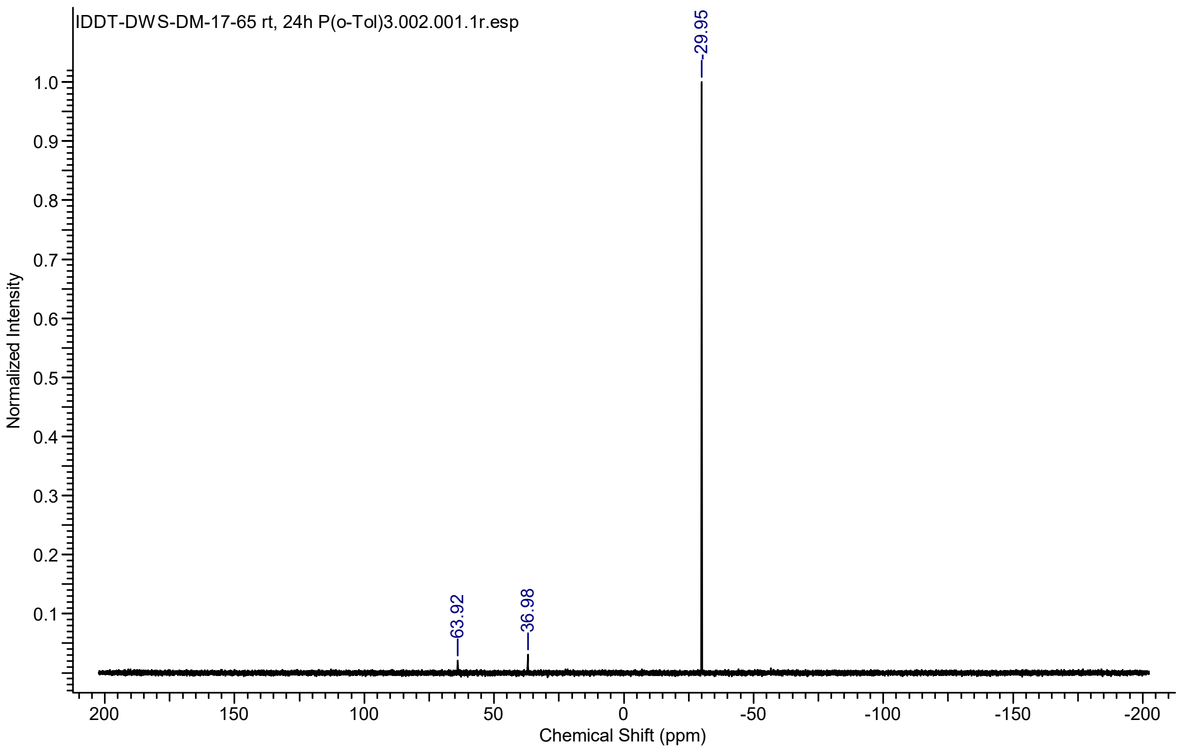

Figure S62.^{31}**P NMR (203 MHz) spectrum of the crude mixture of 3-(4-bromophenyl)-3-chloro-diazirine and P(*o*-Tol)**$_3$ **after 24 h at RT.**

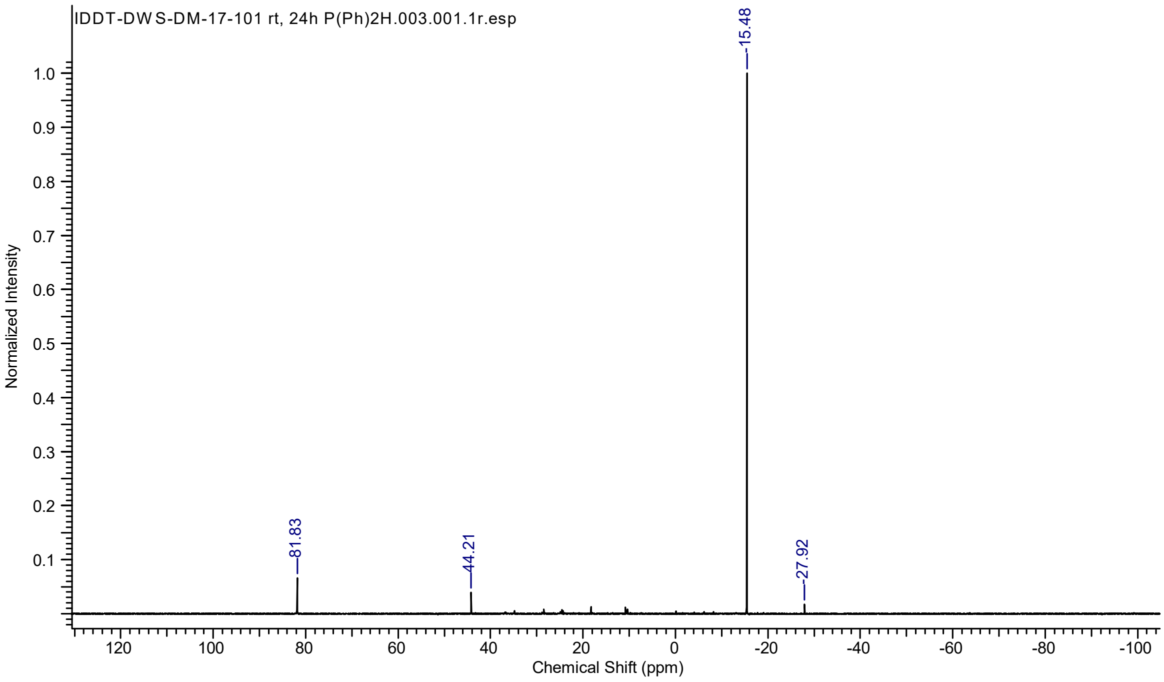

Figure S63.^{31}**P NMR (203 MHz) spectrum of the crude mixture of 3-(4-bromophenyl)-3-chloro-diazirine and HP(Ph)**$_2$ **after 24 h at RT.**

Experimental references

1. Sheldrick, G. M. *Acta Cryst*. *Sec*. *A* **2008**, 64, 112-122.

2. Dolomanov, O. V.; Bourhis, L. J.; Gildea, R. J.; Howard, J. A. K.; Puschmann, H. *J. Appl. Crystallogr*. **2009**, *42*, 339-341.

3. Gazis, T. A.; Dasgupta, A.; Hill, M. S.; Rawson, J. M.; Wirth, T.; Melen, R. L., *Dalton Trans*., **2019**, 48, 12391-12395.

4. Dherange, B. D.; Kelly, P. Q.; Liles, J. P.; Sigman, M. S.; Levin, M. D., *J. Am. Chem. Soc.* **2021**, 143, 11337−11344.

Computational Details

The quantum chemical DFT calculations have been performed with the TURBOMOLE 7.4 suite of programs[1] The initial structures generated according to their Lewis structures are checked with the CREST method using the xTB program for low-lying conformers as input.[2] The structures are fully optimized at the TPSS-D3/def2-TZVP + COSMO level of theory, which combines the TPSS meta-GGA density functional[3] with the BJ-damped DFT-D3 dispersion correction[4] and the def2-TZVP basis set,[5] using the Conductor-like Screening Model (COSMO) continuum solvation model[6] for CH_2Cl_2 solvent (dielectric constant ε = 8.93 and solvent diameter R_{solv} = 2.94 Å). The density-fitting RI-J approach[5a, 7] is used to accelerate the geometry optimization and numerical harmonic frequency calculations[8] in solution. The optimized structures are characterized by frequency analysis to identify the nature of located stationary points (no imaginary frequency for true minima and only one imaginary frequency for transition state) and to provide thermal corrections (at 298.15 K and 1 atm) according to the modified ideal gas – rigid rotor – harmonic oscillator model.[9] This choice of dispersion-corrected meta-GGA functional makes the efficient exploration of all potential reaction paths possible.

The final solvation free energies in CH_2Cl_2 solution are computed with the COSMO-RS solvation model[10] (parameter file: BP_TZVP_C30_1601.ctd) using the COSMOtherm program package[[11] on the above TPSS-D3 optimized structures, and corrected by +1.89 $kcal \cdot mol^{-1}$ to account for higher reference solute concentration of 1 $mol \cdot L^{-1}$ usually used in solution. To check the effects of the chosen DFT functional on the reaction energies and barriers, single-point calculations at the meta-GGA TPSS-D3[3] and hybrid-meta-GGA PW6B95-D3[12] levels are performed using a larger def2-QZVP basis set.[5b, 13] The final reaction Gibbs free energies (ΔG) are determined from the electronic single-point energies plus TPSS-D3 thermal corrections and COSMO-RS solvation free energies. In our discussion, higher-level PW6B95-D3 Gibbs free energies (in kcal/mol, at 298.15 K and 1 mol/L concentration) will be used in our discussion unless specified otherwise. The applied

DFT methods in combination with the large AO basis set provide usually accurate electronic energies leading to errors for chemical energies (including barriers) on the order of typically 1-2 kcal/mol. This has been tested thoroughly for the huge data base GMTKN55[14] which is the common standard in the field of DFT benchmarking. To help experimental NMR assignment, nuclear magnetic shielding constants are computed using the GIAO (Gauge Including Atomic Orbital) method[15] at the TPSS/def2-QZVP level; final ^{31}P, ^{11}B NMR chemical shifts are computed using the known experimental ^{31}P and ^{11}B-NMR signals at 26.4 and 3.0 ppm of compound **1** (*o*-Tol)$_3$PNC(C_6H_4Br)N(C_6F_5)B(C_6F_5)$_2$Cl in this work as reliable reference, while ^{13}C and ^{1}H NMR chemical shifts are computed relative to Si(CH_3)$_4$.

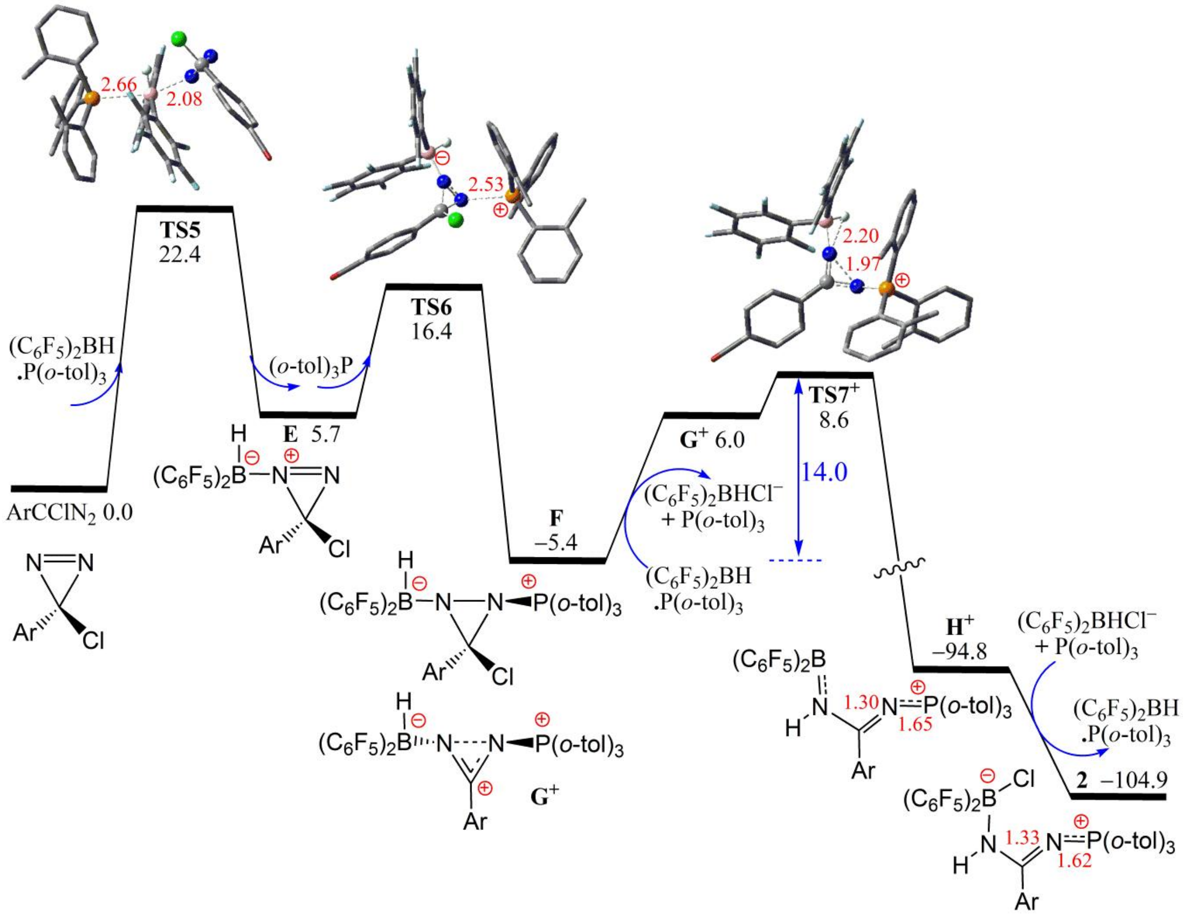

Figure S64. DFT computed complete Gibbs free energy paths (in kcal/mol, at 298 K and 1 M concentration in CH_2Cl_2 solution) for the formation of 2.

The diazirine $ArCClN_2$ (Ar = *p*-C_6H_4Br) may directly abstract a $(C_6F_5)_2BH$ unit from the stable Lewis adduct $(C_6F_5)_2BH$ • P(*o*-tol)$_3$, which is 5.7 kcal/mol endergonic over a sizable barrier of 22.4 kcal/mol (via **TS5**) to form the Lewis adduct **E** along with released base P(*o*-tol)$_3$; further P…N addition between E and P(*o*-tol)$_3$ is still 11.1 kcal/mol exergonic over a low barrier of 10.7 kcal/mol (via **TS6**), making the formation of **F** as the FLP adduct of $ArCClN_2$ 5.4 kcal/mol exergonic from the stable Lewis adduct $(C_6F_5)_2BH$•P(*o*-tol)$_3$.

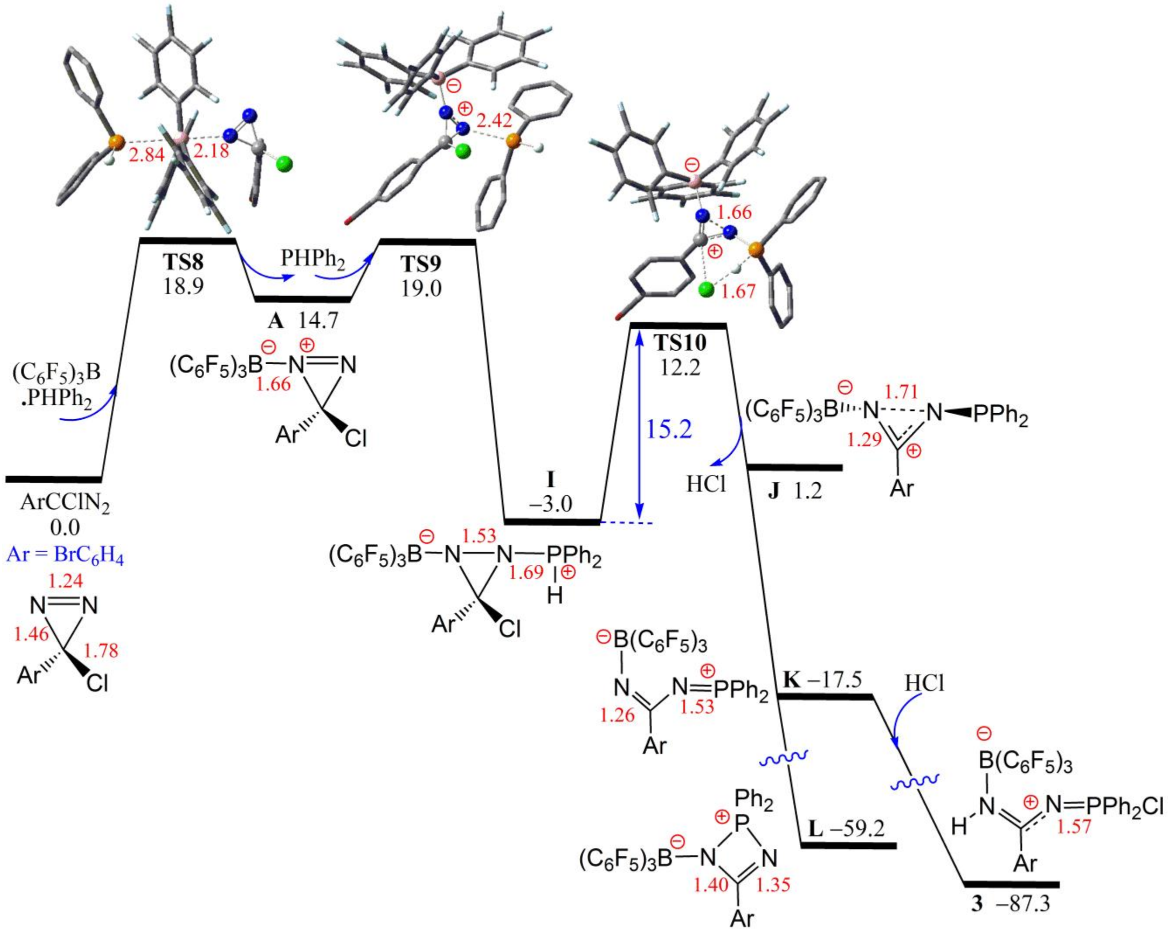

Figure S65. DFT computed complete Gibbs free energy paths (in kcal/mol, at 298 K and 1 M concentration in CH_2Cl_2 solution) for the formation of 3.

The diazirine $ArCClN_2$ (Ar = *p*-C_6H_4Br) may directly abstract a $B(C_6F_5)_3$ unit from the stable Lewis adduct $(C_6F_5)_3B$•$PHPh_2$, which is 14.7 kcal/mol endergonic over a moderate barrier of 18.9 kcal/mol (via **TS8**) to form the Lewis adduct **A** along with released base $HPPh_2$; further P…N addition between **A** and $HPPh_2$ is still 17.7 kcal/mol exergonic over a very low barrier of 4.3 kcal/mol (via **TS9**), making the formation of **I** as the FLP adduct of $ArCClN_2$ 3.0 kcal/mol exergonic from the stable Lewis adduct $(C_6F_5)_3B$•$PHPh_2$.

Table S1. TPSS-D3/def2-TZVP + COSMO computed imaginary frequency (ImF), zero-point energies (ZPE), gas-phase enthalpic (Hc) and Gibbs free-energy (Gc) corrections; the COSMO-RS computed solvation enthalpic (Hsol) and Gibbs free-energy (Gsol) corrections in CH_2Cl_2 solution; TPSS-D3/def2-QZVP and PW6B95-D3/def2-QZVP single-point energies (TPSS-D3 and PW6B95-D3); the total PW6B95-D3 free energies G_P; the relative electronic energies (ΔE_T and ΔE_P) and Gibbs energies (ΔG_T and ΔG_P) at the TPSS-D3 and PW6B95-D3 levels. (groups Ph = C_6H_5; To = *o*-$C_6H_5CH_3$; f = C_6F_5)

Reactions in CH_2Cl_2	Im cm^{-1}	ZPE kcal /mol	Hc kcal /mol	Gc kcal /mol	Hsol kcal /mol	Gsol kcal /mol	TPSS-D3 E_h	PW6B95-D3 E_h	G_P E_h	ΔE_T kcal /mol	ΔE_P kcal /mol	*ΔG_P kcal /mol*	ΔG_T kcal /mol
*Direct chloride Cl- abstraction from diazirine **Rc** AcCClN₂ (Ar = p-C₆H₄Br) with borane is highly endergonic and thus unlikely*													
$ArCN_2^+ + Cl^-$	0	58.58	65.68	27.81	-147.29	-124.68	-3412.94433	-3414.90368	-3415.05204	0.00	0.00	*0.00*	0.00
$ArCClN_2$ (or **Rc**)	0	59.19	65.70	36.52	-13.64	-9.58	-3413.19404	-3415.15563	-3415.10969	-156.70	-158.10	*-36.18*	-34.78
..though chloride Cl⁻ is bound to boranes Bf₃ and (HBf₂)₂ in solution (f = C₆F₅)													
$Bf_3 + Cl^-$	0	94.50	114.69	51.66	-104.39	-85.06	-2669.93293	-2672.52064	-2672.56785	0.00	0.00	*0.00*	0.00
Bf_3Cl^-	0	94.07	114.03	58.89	-46.00	-41.86	-2670.02433	-2672.61506	-2672.58491	-57.35	-59.25	*-10.71*	-8.81
$0.5(HBf_2)_2 + Cl^-$	0	70.40	84.88	40.40	-98.66	-80.68	-1942.29338	-1944.13924	-1944.19891	0.00	0.00	*0.00*	0.00
HBf_2Cl^-	0	69.63	83.53	39.65	-49.54	-44.25	-1942.36254	-1944.20937	-1944.21370	-43.40	-44.01	*-9.28*	-8.67
Frustrated Lewis pair of PTo₃ (To = o-C₆H₅CH₃) and Bf₃ (f = C₆F₅)													
$Bf_3 + PTo_3$	0	314.95	347.08	253.60	-35.27	-26.87	-3364.48029	-3367.88909	-3367.52176	0.00	0.00	*0.00*	0.00
$Bf_3.PTo_3$	0	316.62	348.94	270.95	-28.91	-23.64	-3364.49767	-3367.90427	-3367.50713	-10.91	-9.52	*9.18*	7.79
*Formation of **1** from Bf₃, PTo₃ and diazirine **Rc** AcCClN₂ (Ar = p-C₆H₄Br)*													
The metable N..B complexes is unstable at room temperature, but it can be stabilized by additional N..P addition													
Bf_3 + **Rc** + PTo_3	0	374.13	412.77	290.12	-48.91	-36.46	-6777.67433	-6783.04472	-6782.63146	0.00	0.00	*0.00*	0.00
**A** + PTo_3	0	374.84	413.71	305.79	-45.25	-34.76	-6777.69494	-6783.06508	-6782.62713	-12.94	-12.77	*2.71*	2.55
**TS1**	0	375.09	414.88	320.76	-39.30	-31.85	-6777.71482	-6783.07787	-6782.61445	-25.41	-20.80	*10.67*	6.07
**B**	0	376.55	415.37	324.44	-42.27	-34.67	-6777.74090	-6783.11485	-6782.65006	-41.77	-44.01	*-11.67*	-9.44
..followed by facile chloride Cl⁻ abstraction with another B(C₆F₅)₃													
**B** + Bf_3	0	471.21	528.82	385.42	-57.19	-46.53	-8987.35723	-8994.98248	-8994.43640	0.00	0.00	*0.00*	0.00
**B**.Bf_3	0	471.98	530.40	402.46	-52.88	-45.22	-8987.37671	-8995.00050	-8994.42820	-12.22	-11.31	*5.15*	4.23
**TS2**	206i	470.55	529.00	399.81	-47.23	-40.29	-8987.37076	-8994.99327	-8994.41733	-8.49	-6.77	*11.97*	10.25
$C^+ + ClBf_3^-$	0	470.80	528.69	384.23	-115.43	-102.02	-8987.27603	-8994.89463	-8994.43888	50.96	55.13	*-1.55*	-5.73
..rapid N-N cleavage and rate-limiting aryl-shift													
C^+	0	376.73	414.66	325.34	-69.43	-60.16	-6317.25170	-6322.27957	-6321.85397	0.00	0.00	*0.00*	0.00

**TS3**$^+$	130i	374.65	412.93	322.21	-67.01	-57.85	-6317.25122	-6322.27061	-6321.84631	0.30	5.62	*4.80*	-0.51
**D**$^+$	0	378.40	416.30	326.51	-63.87	-55.12	-6317.37508	-6322.41471	-6321.97921	-77.43	-84.81	*-78.59*	-71.21
..and exergonic chloride Cl^- transfer to the Bf_2-site from $ClBf_3^-$ anion													
**D**$^+$ + $ClBf_3^-$	0	472.47	530.33	385.40	-109.87	-96.98	-8987.39942	-8995.02977	-8994.56412	0.00	0.00	*0.00*	0.00
**D**$^+$.$ClBf_3^-$	0	473.31	531.72	402.69	-48.78	-41.24	-8987.49101	-8995.12194	-8994.54293	-57.47	-57.84	*13.29*	13.66
**TS4**	71i	472.12	530.48	401.53	-51.48	-44.04	-8987.47688	-8995.10527	-8994.53256	-48.61	-47.38	*19.80*	18.57
**1** + Bf_3	0	472.70	530.36	386.55	-56.50	-45.74	-8987.48639	-8995.11434	-8994.56521	-54.58	-53.07	*-0.68*	-2.19
*Formation of **2** from $(C_6F_5)_2BH$, (o-tol)$_3$P and the diazirine **Rc** $(BrC_6H_4)CClN_2$*													
Reaction 2 with stable diborane $(HBf_2)_2$ ($f = C_6F_5$)													
$(HBf_2)_2$	0	140.79	166.80	99.68	-17.05	-14.10	-2963.96555	-2966.98523	-2966.84584	0.00	0.00	*0.00*	0.00
**TS5a**	46i	138.61	164.57	96.91	-18.05	-14.83	-2963.92979	-2966.94669	-2966.81288	22.44	24.18	*20.68*	18.94
2*HBf_2	0	139.23	164.59	83.00	-22.71	-17.18	-2963.92429	-2966.94310	-2966.83218	25.89	26.44	*8.57*	8.02
Lewis adduct formation is very facile between PTo_3 and dimeric $(HBf_2)_2$													
PTo_3 + $(HBf_2)_2$	0	361.24	400.67	292.18	-38.07	-29.54	-4118.82351	-4123.00031	-4122.57574	0.00	0.00	*0.00*	0.00
**TS5b**	0	361.05	400.69	308.06	-32.95	-27.52	-4118.82968	-4123.00497	-4122.55489	-3.87	-2.92	*13.09*	12.14
HBf_2.PTo_3 + HBf_2	0	361.48	400.44	292.73	-38.77	-30.55	-4118.82170	-4122.99814	-4122.57431	1.13	1.36	*0.90*	0.67
..followed by even fast adduct formation between HBf_2 and PTo_3													
HBf_2 + PTo_3	0	290.06	316.16	234.01	-32.37	-24.04	-2636.82011	-2639.48663	-2639.14600	0.00	0.00	*0.00*	0.00
HBf_2.PTo_3	0	291.87	318.15	251.23	-27.41	-21.96	-2636.85956	-2639.52660	-2639.15822	-24.76	-25.08	*-7.67*	-7.35
..with the P..B complex of $(HBf_2)_2$ and PTo_3 is 3.4 kcal/mol stable than PTo_3													
PTo_3 + 0.5$(HBf_2)_2$	0	290.84	317.27	242.35	-29.54	-22.50	-2636.84074	-2639.50770	-2639.15283	0.00	0.00	*0.00*	0.00
HBf_2.PTo_3	0	291.87	318.15	251.23	-27.41	-21.96	-2636.85956	-2639.52660	-2639.15822	-11.81	-11.86	*-3.39*	-3.34
*The N..B complexes of **Rc** $ArCClN_2$ with diborane $(HBf_2)_2$ is 2.3 kcal/mol unstable*													
**Rc** + 0.5$(HBf_2)_2$	0	129.58	149.10	86.36	-22.17	-16.63	-4895.17681	-4898.64824	-4898.53261	0.00	0.00	*0.00*	0.00
**Rc**.HBf_2	0	129.96	149.33	94.41	-21.26	-16.51	-4895.18834	-4898.65606	-4898.52890	-7.23	-4.90	*2.33*	0.00
..which can be stabilized by PTo_3 addition													
**Rc** + HBf_2.PTo_3	0	351.05	383.84	287.75	-41.06	-31.55	-6050.05360	-6054.68222	-6054.26791	0.00	0.00	*0.00*	0.00
**TS5**	218i	349.35	383.21	300.20	-35.50	-28.09	-6050.04313	-6054.66883	-6054.23219	6.57	8.40	*22.41*	20.58
**E** + PTo_3	0	350.40	383.20	286.92	-42.27	-31.96	-6050.04630	-6054.67114	-6054.25881	4.58	6.96	*5.71*	3.34
**TS6**	73i	349.86	383.45	300.38	-39.00	-31.07	-6050.05818	-6054.67390	-6054.24171	-2.88	5.23	*16.44*	8.34
**F**	0	351.53	384.68	303.75	-41.57	-33.55	-6050.08384	-6054.71011	-6054.27652	-18.98	-17.50	*-5.40*	-6.87

*..followed by HBf_2 unit transfer from the Lewis adduct HBf_2PTo_3 to the adduct **F***

**F** + $HBf_2.PTo_3$	0	643.61	702.93	555.60	-69.05	-55.45	-8686.94789	-8694.24065	-8693.43758	0.00	0.00	*0.00*	0.00
**G0** + PTo_3	0	642.77	702.75	555.39	-68.36	-54.81	-8686.93125	-8694.21862	-8693.41488	10.44	13.82	*14.25*	10.87

..and chloride Cl- abstraction with HBf2 and further Cl- transfer to the boron site of H+

**G0**	0	422.33	468.88	362.88	-47.34	-39.37	-7532.07328	-7538.20354	-7537.68497	-4.19	-0.51	*8.85*	4.00
G^+ + f_2BHCl^-	0	421.31	467.29	344.97	-116.61	-101.90	-7531.95442	-7538.08292	-7537.68953	70.40	75.18	*5.98*	0.03
**TS7**$^+$	93i	419.25	465.62	341.47	-113.57	-99.08	-7531.95733	-7538.07763	-7537.68533	68.58	78.50	*8.62*	-2.48
**H**$^+$ + f_2BHCl^-	0	424.77	470.35	348.69	-113.69	-99.09	-7532.10740	-7538.25389	-7537.85010	-25.59	-32.10	*-94.78*	-89.43
**TS7a**	0	423.60	470.53	362.88	-45.64	-37.94	-7532.21929	-7538.36609	-7537.84525	-95.80	-102.51	*-91.73*	-86.19
**2a** + $0.5(HBf_2)_2$	0	424.96	471.21	356.44	-70.39	-53.86	-7532.18201	-7538.32532	-7537.83861	-72.41	-76.93	*-87.57*	-84.22
**2** + $0.5(HBf_2)_2$	0	424.71	470.82	356.51	-51.10	-41.11	-7532.21923	-7538.36797	-7537.86084	-95.77	-103.69	*-101.51*	-94.76

*Formation of **3** from $B(C_6F_5)_3$, PPh_2H and the diazirine **Rc** $BrC_6H_4)CClN_2$*

12.0 kcal/mol stable Lewis adduct of Bf_3 and $HPPh_2$

Bf_3 + $HPPh_2$	0	213.28	239.38	157.08	-30.40	-22.71	-3015.23097	-3018.25892	-3018.03877	0.00	0.00	*0.00*	0.00
$Bf_3.PHPh_2$	0	214.23	240.73	172.56	-25.81	-20.96	-3015.27411	-3018.30251	-3018.05791	-27.06	-27.35	*-12.01*	-11.72

*Facile FLP addition to the NNC ring of **Rc** $ArCClN_2$*

**Rc** + $Bf_3.PHPh_2$	0	273.42	306.43	209.08	-39.45	-30.54	-6428.46815	-6433.45814	-6433.16760	0.00	0.00	*0.00*	0.00
**TS8**	126i	272.26	305.97	222.93	-33.48	-26.70	-6428.46033	-6433.45323	-6433.13751	4.91	3.08	*18.89*	20.71
**A** + $HPPh_2$	0	273.17	306.01	209.27	-40.38	-30.59	-6428.44563	-6433.43491	-6433.14414	14.13	14.58	*14.72*	14.27
**TS9**	36i	272.25	305.77	222.92	-35.18	-28.31	-6428.46885	-6433.45046	-6433.13732	-0.44	4.82	*19.00*	13.74
**I**	0	274.06	307.17	226.18	-39.89	-32.59	-6428.49327	-6433.48383	-6433.17231	-15.76	-16.12	*-2.96*	-2.60
**TS10**	562i	270.36	304.32	220.62	-42.79	-33.64	-6428.46956	-6433.44910	-6433.14812	-0.88	5.67	*12.23*	5.67
**J** + HCl	0	270.69	305.14	212.14	-39.92	-30.23	-6428.47596	-6433.46164	-6433.16573	-4.90	-2.20	*1.18*	-1.53

..followed by HCl addition

**Rc** + $Bf_3.PHPh_2$	0	273.42	306.43	209.08	-39.45	-30.54	-6428.46815	-6433.45814	-6433.16760	0.00	0.00	*0.00*	0.00
**K** + HCl	0	271.95	306.14	213.79	-44.63	-34.36	-6428.50161	-6433.48753	-6433.19556	-21.00	-18.44	*-17.54*	-20.09
**L** + HCl	0	272.39	306.29	214.42	-40.18	-30.40	-6428.56377	-6433.56121	-6433.26194	-60.01	-64.68	*-59.20*	-54.53
**3**	0	277.14	310.35	228.83	-36.41	-29.25	-6428.63016	-6433.62782	-6433.30676	-101.66	-106.48	*-87.32*	-82.51

Table S2. TPSS-D3/def2-TZVP + COSMO optimized Cartesian coordinates (in Å) in CH_2Cl_2 solution. Each structure is labeled by a specific name (See also **Table S1**, and **Figures S1 and S2**), followed by the number of atoms, the total energy (in hartrees), and the detailed atomic coordinates (in double-column text list). (groups Ph = C_6H_5; To = *o*-$C_6H_5CH_3$; f = C_6F_5)

1 : product $To_3PNCArNfBf_2Cl$

92

Energy = -6777.628402589

Br	-2.7629237	5.9663355	-1.1402431
C	-1.9498753	4.2979248	-0.6965085
C	-1.0956074	3.6929819	-1.6172210
C	-2.2412770	3.7094991	0.5315220
C	-0.5305358	2.4623411	-1.2983019
H	-0.8841000	4.1656519	-2.5694280
C	-1.6830229	2.4698096	0.8277686
H	-2.9017669	4.1986613	1.2375129
C	-0.8328749	1.8352220	-0.0827080
H	0.1194997	1.9708865	-2.0144823
H	-1.9110061	1.9953948	1.7770743
C	-0.3059227	0.4690155	0.2018474
N	1.0417813	0.2969584	0.1952414
N	-1.0771739	-0.5662675	0.4139475
C	1.8454617	1.4699279	0.2412558
B	1.7324627	-1.1118114	0.4220866
P	-2.6800602	-0.8097445	0.5067951
C	1.8351957	2.3054302	1.3635289
C	2.7121962	1.8088314	-0.8018980
Cl	1.2816578	-1.5987184	2.2329133
C	1.1378663	-2.2596637	-0.5789506
C	3.3638088	-0.9599564	0.3231655
C	-3.7633701	0.4625111	-0.2120393
C	-3.0658346	-0.9306701	2.2703827
C	-3.0245317	-2.4044903	-0.2673368
F	1.0294156	2.0227377	2.4004539
C	2.6480955	3.4303376	1.4468675
F	2.7316431	1.0682684	-1.9233101
C	3.5491302	2.9171131	-0.7279352
C	0.4910213	-1.9996805	-1.7898114
C	1.2816447	-3.6218844	-0.3005677
C	4.1133476	-1.4242620	-0.7654540
C	4.1411980	-0.3547689	1.3225567
C	-3.6342795	0.8863541	-1.5546084
C	-4.6675642	1.1150254	0.6447675
C	-2.0847737	-0.4838359	3.1680656
C	-4.2816730	-1.4747244	2.7463913
C	-2.4488725	-3.5960592	0.2362251
C	-3.9158899	-2.4528255	-1.3496598
F	2.6169632	4.2122219	2.5388554
C	3.5165889	3.7325410	0.4010937
F	4.3773398	3.2111659	-1.7437577
F	0.3875169	-0.7306087	-2.2655100
C	-0.0537197	-2.9795535	-2.6125856
F	1.9856829	-4.0378901	0.7790214
C	0.7570760	-4.6372413	-1.0965925
F	3.5148705	-1.9880805	-1.8420028
C	5.5007035	-1.3283674	-0.8566717
F	3.5643078	0.1963376	2.4155071
C	5.5262508	-0.2327929	1.2681930
C	-2.6935147	0.2488900	-2.5439341
C	-4.4249201	1.9593083	-1.9838117
H	-4.7401005	0.8098050	1.6830556
C	-5.4495987	2.1692807	0.1891867
H	-1.1328926	-0.1305015	2.7870070
C	-2.3013013	-0.5432577	4.5413148
C	-4.4732743	-1.5174559	4.1324088
C	-5.3559731	-2.0309453	1.8450060
C	-2.7525745	-4.7912722	-0.4265712
C	-1.5880357	-3.6435315	1.4687753
H	-4.4014894	-1.5441781	-1.6866164
C	-4.2021408	-3.6553728	-1.9863768
F	4.3171755	4.8048027	0.4806010
F	-0.6919343	-2.6437894	-3.7540513
C	0.0717116	-4.3170099	-2.2605760
F	0.9083450	-5.9323224	-0.7450224
F	6.1505122	-1.8022503	-1.9383987
C	6.2181814	-0.7292550	0.1697946
F	6.2016445	0.3723192	2.2658239
H	-1.9917747	-0.4398481	-2.0744703
H	-2.1180167	1.0170072	-3.0669953
H	-3.2527748	-0.3112267	-3.3014338
H	-4.3202047	2.3023358	-3.0098957
C	-5.3228719	2.5965723	-1.1327449
H	-6.1395245	2.6627618	0.8661704
H	-1.5330627	-0.1976224	5.2259292
C	-3.5039746	-1.0579322	5.0234167
H	-5.4057947	-1.9240115	4.5151259
H	-5.5167726	-1.4150205	0.9559242
H	-6.3012797	-2.1016736	2.3887344
H	-5.0883451	-3.0357628	1.4987378
H	-2.3069603	-5.7124418	-0.0601023
C	-3.6043987	-4.8290285	-1.5285195
H	-0.8807776	-2.8128508	1.5182950
H	-1.0290673	-4.5820880	1.5015198
H	-2.2139975	-3.5989718	2.3687018
H	-4.8859081	-3.6733182	-2.8290654
F	-0.4500579	-5.2827330	-3.0375525
F	7.5550427	-0.6237250	0.0995061
H	-5.9144361	3.4307792	-1.4976128
H	-3.6874531	-1.1096333	6.0926572
H	-3.8099931	-5.7755401	-2.0195595

2a : contact ion pair $Bf_2NHCArNPTo_3^+Cl^-$

82

Energy = -6050.014508954

Br	-4.5514850	-3.4780428	-3.6931711

Cl	1.4900251	-1.1120538	-4.0497715
P	-1.8826586	0.7150295	1.3121242
F	4.8552083	-1.0040561	-0.1132747
F	2.2226975	1.9568511	-2.7468731
F	1.5036440	-2.2506294	1.0195456
F	3.2129967	2.1666125	1.2691132
F	3.4966948	1.8996494	3.9504325
F	1.7558950	-2.4989006	3.7051370
F	6.9847358	-0.4964288	-1.6938826
F	2.7756343	-0.4324301	5.1778965
F	4.3526172	2.4248835	-4.3338072
N	0.9730974	-0.1726472	-1.1999365
H	1.0706493	-0.4578750	-2.2221479
F	6.7407757	1.2102972	-3.8117263
N	-0.5721659	0.5351824	0.3522920
C	-0.3102241	-0.1547456	-0.7271495
C	2.3122383	-0.0189012	1.0564125
C	-1.3040134	-0.9100146	-1.5174807
C	2.8506962	0.9964678	1.8446349
C	3.4444852	0.4348991	-1.3837527
C	4.6960972	-0.1403301	-1.1426631
C	3.3698706	1.3024730	-2.4808235
C	-1.2556666	0.7473768	3.0038661
C	-0.3595897	1.7595266	3.4248673
C	1.9649446	-1.1921136	1.7263563
C	-2.5726061	-0.3754635	-1.7757797
H	-2.8087314	0.6377290	-1.4676840
C	-2.5753500	2.3399680	0.9318111
C	-3.5333752	-1.1200225	-2.4480077
H	-4.5130030	-0.7045001	-2.6512188
C	-3.2006507	-0.5212340	1.1567533
C	-2.1617206	2.9515034	-0.2617136
H	-1.4408256	2.4485704	-0.8980977
C	0.1170586	2.8769327	2.5323594
H	0.3151949	2.5321872	1.5158159
H	1.0274874	3.3222147	2.9406530
H	-0.6395952	3.6681767	2.4727074
C	-4.4916095	-0.0722101	0.8240535
H	-4.6893787	0.9912974	0.7420495
C	2.1075923	-1.3510168	3.0995694
C	3.0082362	0.8802013	3.2211066
C	-0.9863551	-2.1940742	-1.9840392
H	0.0044084	-2.6011336	-1.8193621
C	-2.9282605	-1.9055982	1.2259863
C	-3.4728229	2.9991893	1.8034271
C	-1.7176190	-0.2156981	3.9140276
H	-2.4401273	-0.9574634	3.5917437
C	0.0624344	1.7344965	4.7592872
H	0.7547931	2.5006705	5.0978432
C	2.6333573	-0.3031506	3.8516410
C	5.8039683	0.0963840	-1.9464866
C	-3.2133639	-2.4191275	-2.8404703
C	-1.2757931	-0.2192386	5.2324168
H	-1.6348039	-0.9758265	5.9224884
C	4.4594485	1.5758019	-3.2971345
C	-2.6424460	4.2062438	-0.6205485
H	-2.3170008	4.6676521	-1.5473605
C	-1.5739571	-2.4698040	1.5644819
H	-1.2148605	-3.1095397	0.7533678
H	-1.6299954	-3.0860689	2.4681233
H	-0.8288360	-1.6937792	1.7384148
C	-3.9416153	4.2602800	1.4157128
H	-4.6384278	4.7780197	2.0695632
C	-1.9488513	-2.9650153	-2.6272123
H	-1.7165632	-3.9703558	-2.9585711
C	-0.3771440	0.7603530	5.6536822
H	-0.0215242	0.7711458	6.6795050
C	5.6808502	0.9615078	-3.0313114
C	-5.5120300	-0.9768195	0.5610247
H	-6.5014078	-0.6151124	0.3010331
C	-3.9724241	-2.7951393	0.9456735
H	-3.7697370	-3.8622601	0.9824523
C	-3.5387031	4.8607769	0.2241090
H	-3.9225541	5.8411698	-0.0416505
B	2.1892060	0.1046104	-0.5082507
C	-3.9210186	2.4304886	3.1268562
H	-4.1034038	1.3534587	3.0813406
H	-4.8423714	2.9201235	3.4512522
H	-3.1597979	2.5988635	3.8973681
C	-5.2470742	-2.3461858	0.6143233
H	-6.0317047	-3.0632842	0.3930501

2 : product $To_3PNCArNHBf_2Cl$
82
Energy = -6050.036952058

Br	-6.6928191	-0.4535984	-2.0054578
Cl	2.0129775	-2.5403040	-3.3283317
P	-0.5847538	1.0678730	1.1705977
F	4.5467875	-1.5783093	-1.5621788
F	1.1086357	1.5057368	-2.7567914
F	0.1557290	-3.7604649	-1.2677784
F	3.3169015	-0.7736896	0.7079043
F	3.3108894	-2.2551452	2.9079212
F	0.1651396	-5.2108390	0.9651548
F	6.3668036	0.3697928	-1.8509984
F	1.7325079	-4.4851057	3.0966275
F	2.9279353	3.4201456	-3.0008921
N	0.1786012	-0.9696720	-2.0159821
H	-0.3009464	-1.3375135	-2.8305717
F	5.5856354	2.9014458	-2.5608412
N	0.0019838	0.3398688	-0.1499759
C	-0.5770166	-0.2740105	-1.1731471
C	1.7811709	-2.2077457	-0.4409994
C	-2.0426269	-0.2821549	-1.4436555
C	2.5364539	-1.8776428	0.6865063
C	2.7203159	-0.1286112	-2.0448233
C	4.0934675	-0.3438405	-1.8879293
C	2.3897632	1.1620038	-2.4603158
C	0.5680480	0.7681161	2.5307360
C	1.8886821	1.2731643	2.4785514

C	0.9928214	-3.3538398	-0.2782783
C	-2.8120252	0.8816104	-1.3476263
H	-2.3373522	1.8308326	-1.1228556
C	-0.6029488	2.8625534	0.9140734
C	-4.1895493	0.8433023	-1.5380064
H	-4.7813762	1.7477614	-1.4595025
C	-2.2459737	0.5378106	1.6718031
C	-0.3793486	3.3131895	-0.3951739
H	-0.2057084	2.5873279	-1.1813110
C	2.4343892	2.0468390	1.3054063
H	2.0906740	1.6315880	0.3562871
H	3.5266596	2.0254763	1.3204701
H	2.1164707	3.0949814	1.3514963
C	-3.2552340	1.5049572	1.8097936
H	-3.0170114	2.5531812	1.6630420
C	0.9662146	-4.1277718	0.8768473
C	2.5429444	-2.6240796	1.8639699
C	-2.6737560	-1.4934489	-1.7627744
H	-2.0873228	-2.4046877	-1.8382168
C	-2.5541231	-0.8336620	1.8146932
C	-0.7594752	3.7893263	1.9712780
C	0.1108919	0.0992600	3.6766055
H	-0.9144238	-0.2530662	3.7193181
C	2.7092369	1.0585338	3.5917914
H	3.7301277	1.4300715	3.5615098
C	1.7498725	-3.7593977	1.9638679
C	5.0592390	0.6426236	-2.0423060
C	-4.7953488	-0.3806524	-1.8128860
C	0.9487494	-0.1011122	4.7679945
H	0.5820897	-0.6260086	5.6444646
C	3.3224379	2.1857901	-2.6213623
C	-0.3340294	4.6728744	-0.6854086
H	-0.1548059	5.0042811	-1.7033625
C	-1.5424595	-1.9354161	1.6280166
H	-1.6858104	-2.4111867	0.6511639
H	-1.6751224	-2.7093991	2.3896592
H	-0.5119045	-1.5807970	1.6713160
C	-0.7212286	5.1518879	1.6493546
H	-0.8528687	5.8769692	2.4483969
C	-4.0534008	-1.5536756	-1.9375340
H	-4.5409688	-2.4971265	-2.1548903
C	2.2557940	0.3826108	4.7221500
H	2.9239413	0.2322398	5.5650454
C	4.6691980	1.9285962	-2.4042791
C	-4.5629386	1.1335771	2.0971989
H	-5.3327429	1.8924147	2.1952957
C	-3.8790754	-1.1813277	2.1056519
H	-4.1282280	-2.2333135	2.2189514
C	-0.5129831	5.5959607	0.3443253
H	-0.4835088	6.6614531	0.1360022
B	1.6701411	-1.3529694	-1.8386058
C	-0.9224208	3.3947008	3.4184189
H	-1.5413693	2.5033527	3.5459313
H	-1.3787710	4.2136114	3.9803668
H	0.0534193	3.1780458	3.8683167
C	-4.8750293	-0.2184591	2.2443824
H	-5.8940778	-0.5237195	2.4623684

3 : product $Ph_2(Cl)PNCArNHBf_3$

73

Energy = -6428.411187968

Br	6.7303758	3.2858746	-0.9136926
Cl	3.8905693	-2.0007093	0.6131379
P	1.8437707	-2.1064403	0.3223494
F	-0.9909704	1.4576821	-2.9340777
F	-1.2241049	-0.1345404	-5.0795651
F	-3.8255357	-0.3170011	0.6415904
F	-2.0183899	-2.2496896	-0.1173957
F	-3.8539105	1.2232179	-1.8411270
F	-0.8489986	3.2667083	1.2569319
F	0.4918228	0.7763329	2.3604538
F	-0.3222648	0.0882362	4.7852777
F	-1.8895207	-2.7807488	-4.7774597
F	-5.3281967	3.4333327	-1.8413284
F	-4.5733629	-1.1258464	3.0899904
F	-2.2891705	-3.7891353	-2.2599587
F	-2.8274123	-0.9327901	5.2006893
F	-2.3489146	5.4850575	1.2159471
F	-4.6157188	5.6018721	-0.3294583
N	0.1882621	1.2484092	-0.3268659
H	0.3762324	2.2336043	-0.4747972
N	1.1504331	-0.8294031	-0.2678219
C	-2.2441029	2.1305193	-0.3137160
C	1.2671206	0.4918756	-0.3253530
C	-1.5310954	-0.2921043	-1.4075526
C	2.5866227	1.1562972	-0.4572737
C	-1.3175935	0.1556440	-2.7143078
C	1.2332140	-2.5935419	1.9406985
C	-1.8503976	-1.6452338	-1.3194266
C	-1.4336982	-0.6418661	-3.8470056
C	-1.6164913	0.2159584	1.3419585
C	-0.7795912	0.3033386	2.4562963
C	-3.4143333	2.2452953	-1.0681235
C	1.6050691	-3.4351156	-0.8469601
C	-2.9011921	-0.2524439	1.6330175
C	3.5489018	0.6419428	-1.3342180
H	3.3294561	-0.2458983	-1.9178959
C	-1.9468065	3.2652258	0.4469405
C	-1.7810766	-1.9808564	-3.7003372
C	-4.2122787	3.3879140	-1.0857130
C	-1.9900292	-2.4842299	-2.4234065
C	-3.3236292	-0.6565068	2.8928867
C	0.0282790	-3.3124145	1.9947021
H	-0.4786572	-3.6043929	1.0826387
C	-1.1697557	-0.0560488	3.7457686
C	-0.5151667	-3.6514657	3.2308369
H	-1.4470347	-4.2060312	3.2723538
C	1.2714645	-3.1266030	-2.1726208
H	1.1261613	-2.0913815	-2.4634421
C	1.8864049	-2.2188918	3.1227460

H	2.8169508	-1.6634029	3.0830488
C	4.7778203	1.2762950	-1.4837829
H	5.5171062	0.8886817	-2.1752055
C	2.8680147	2.3123335	0.2831655
H	2.1364891	2.7012144	0.9855548
C	4.1018483	2.9459948	0.1584940
H	4.3288671	3.8290224	0.7446839
C	-2.4446652	-0.5574607	3.9663467
C	-2.7077332	4.4289709	0.4587426
C	5.0393468	2.4192229	-0.7286242
C	1.7927455	-4.7669069	-0.4494351
H	2.0577947	-5.0010350	0.5772671
C	0.1380032	-3.2832066	4.4083004
H	-0.2883133	-3.5508239	5.3703235
C	-3.8577710	4.4918027	-0.3197008
B	-1.3026333	0.7773430	-0.1748740
C	1.1162764	-4.1559413	-3.0975293
H	0.8528888	-3.9232856	-4.1245790
C	1.3364747	-2.5691191	4.3529224
H	1.8423701	-2.2791333	5.2680518
C	1.6297225	-5.7888729	-1.3807942
H	1.7648246	-6.8218283	-1.0762635
C	1.2912732	-5.4835842	-2.7019090
H	1.1635843	-6.2837845	-3.4245940

$ArCClN_2$: diazirine **Rc** $(BrC_6H_4)CClN_2$
15
Energy = -3413.112367427

Br	3.4178084	0.0003254	0.0549467
C	1.5083902	0.0006801	-0.0381068
C	0.8328643	-1.2180061	-0.0811362
C	0.8324264	1.2194501	-0.0635019
C	-0.5592631	-1.2106987	-0.1464527
H	1.3786140	-2.1546951	-0.0648673
C	-0.5598690	1.2124140	-0.1292330
H	1.3777102	2.1560817	-0.0334983
C	-1.2582702	0.0009924	-0.1715127
H	-1.0990521	-2.1523425	-0.1820738
H	-1.1000246	2.1542433	-0.1523443
C	-2.7404848	0.0013740	-0.2372835
N	-3.4671533	-0.5741981	-1.3667913
N	-3.4681030	0.6687728	-1.3140507
Cl	-3.5896757	-0.0671334	1.3227357

A : Bf_3 adduct of $(BrC_6H_4)CClN_2$ at one N
49
Energy = -5622.648965905

Br	-6.5959051	-0.2376095	0.8923180
C	-4.9306689	0.0916513	0.0273589
C	-4.1808801	-0.9892116	-0.4355431
C	-4.4928459	1.4067449	-0.1284467
C	-2.9571983	-0.7424899	-1.0488147
H	-4.5417098	-2.0048695	-0.3218289
C	-3.2709824	1.6429224	-0.7506921
H	-5.0929339	2.2349440	0.2298891
C	-2.4939776	0.5707285	-1.2012316
H	-2.3710766	-1.5795433	-1.4143824
H	-2.9220473	2.6618478	-0.8751098
C	-1.1655671	0.7933202	-1.8170797
N	0.0044264	0.1354195	-1.2701749
N	-0.3941835	-0.3744781	-2.3336997
Cl	-0.8596381	2.3039591	-2.6374406
B	1.1823304	-0.0873035	-0.1181400
C	0.2915262	-0.7714100	1.0647063
C	2.2575010	-1.0621530	-0.8319828
C	1.6997141	1.4296699	0.1682574
C	0.1627574	-0.2815283	2.3677991
C	-0.4512809	-1.9281729	0.8009052
C	2.7949080	-2.2068652	-0.2403043
C	2.7474390	-0.7793406	-2.1099656
C	0.8024727	2.4556394	0.4802878
C	3.0395286	1.8263973	0.1285859
F	0.8765135	0.7864911	2.7850739
C	-0.6696731	-0.8635193	3.3209477
F	-0.3512198	-2.5236930	-0.4146614
C	-1.3015669	-2.5342444	1.7162356
F	2.4445126	-2.5664475	1.0171331
C	3.7255168	-3.0281630	-0.8719684
F	2.3412249	0.3502216	-2.7463729
C	3.6696003	-1.5723382	-2.7806190
F	-0.5183937	2.1693458	0.6128039
C	1.1690843	3.7760735	0.6995252
F	4.0289848	0.9359494	-0.1091282
C	3.4549761	3.1382485	0.3516814
F	-0.7518822	-0.3510885	4.5603632
C	-1.4173884	-1.9902982	2.9915394
F	-2.0066119	-3.6296076	1.3841423
F	4.2084556	-4.1200324	-0.2517686
C	4.1621127	-2.7133381	-2.1540958
F	4.1001439	-1.2425909	-4.0116090
F	0.2464359	4.7102045	0.9899771
C	2.5150540	4.1234633	0.6303039
F	4.7603864	3.4570970	0.3027365
F	-2.2268874	-2.5537929	3.8971682
F	5.0573126	-3.4943494	-2.7758960
F	2.9004357	5.3891212	0.8428216

$Bf_3.PHPh_2$: adduct of $B(C_6F_5)_3$ and $HPPh_2$
58
Energy = -3015.137621050

B	0.1317664	0.4452220	-0.0341967
C	1.2177867	-0.0115174	1.0896523
C	0.5430086	1.8137204	-0.8301867
C	-1.4283336	0.4885913	0.4395036
C	1.1364983	-1.2716204	1.6883899
C	2.3241590	0.7388766	1.5001337
C	1.5308069	1.8674500	-1.8135218
C	-0.0415123	3.0538231	-0.5505356
C	-2.4238418	0.7133542	-0.5166340
C	-1.9091254	0.3227799	1.7394703

C	2.0635631	-1.7872619	2.5813671
C	3.2820535	0.2674622	2.3978806
C	1.8911279	3.0134832	-2.5120074
C	0.2842011	4.2304963	-1.2203865
C	-3.7845738	0.7265695	-0.2465218
C	-3.2658159	0.3261401	2.0601134
C	3.1582433	-1.0065629	2.9386940
C	1.2533395	4.2128812	-2.2166391
C	-4.2126501	0.5211075	1.0611927
F	0.0855186	-2.0777880	1.3828560
F	2.5301731	1.9980916	1.0459613
F	2.2407236	0.7424545	-2.1167502
F	-0.9573927	3.1835821	0.4401037
F	-2.0611590	0.9285791	-1.8105294
F	-1.0617799	0.1707134	2.7852222
F	1.9205339	-3.0234019	3.0939171
F	4.3246686	1.0425386	2.7524593
F	2.8532526	2.9757692	-3.4534083
F	-0.3241694	5.3893745	-0.9037573
F	-4.6858662	0.9240453	-1.2269399
F	-3.6684283	0.1538720	3.3340372
F	4.0751200	-1.4737518	3.8005448
F	1.5796568	5.3384374	-2.8714102
F	-5.5229842	0.5219936	1.3558790
P	0.1241818	-1.0986310	-1.4483958
C	-1.4502035	-1.9475230	-1.7184793
C	1.3941638	-2.3845585	-1.2438515
H	0.3405171	-0.5727424	-2.7291086
C	-2.2084013	-2.4848072	-0.6669735
C	-1.9263993	-2.0406886	-3.0353094
C	2.7202757	-2.0400728	-0.9371970
C	1.0386988	-3.7365811	-1.3524130
C	-3.4254897	-3.1065920	-0.9356839
H	-1.8589626	-2.4126587	0.3548790
H	-1.3486847	-1.6201714	-3.8532281
C	-3.1450952	-2.6642244	-3.2964696
H	3.0093569	-1.0010526	-0.8412072
C	3.6736053	-3.0380819	-0.7476052
C	1.9975929	-4.7295182	-1.1576086
H	0.0151622	-4.0133402	-1.5817247
H	-4.0084725	-3.5178432	-0.1173905
C	-3.8964798	-3.1954533	-2.2472155
H	-3.5073531	-2.7290534	-4.3178645
H	4.6970260	-2.7624159	-0.5117123
C	3.3144710	-4.3830458	-0.8528141
H	1.7116499	-5.7736244	-1.2413536
H	-4.8489903	-3.6757318	-2.4502490
H	4.0588309	-5.1580445	-0.6963105

Bf3.PTo3 : Lewis adduct of B(C_6F_5)$_3$ and PTo$_3$
77
Energy = -3364.340608951

B	0.0010143	0.0004260	0.8823728
C	1.3414150	-0.8271073	1.2837029
C	0.0483374	1.5753816	1.2824087
C	-1.3871049	-0.7463506	1.2801651
C	1.5780196	-2.1286558	0.8245196
C	2.3504240	-0.3497713	2.1316655
C	1.0568877	2.4302741	0.8210614
C	-0.8675485	2.2112595	2.1318847
C	-2.6308843	-0.2957385	0.8210086
C	-1.4820308	-1.8633324	2.1218690
C	2.7134614	-2.8759896	1.1003628
F	0.6461347	-2.7501946	0.0605941
C	3.5116105	-1.0595260	2.4331874
F	2.2458191	0.8481619	2.7559014
C	1.1373103	3.7873996	1.0958965
F	2.0602931	1.9330501	0.0564940
C	-0.8324168	3.5718680	2.4325605
F	-1.8506982	1.5217648	2.7591683
C	-3.8475593	-0.9041758	1.0913473
F	-2.6996374	0.8255478	0.0617325
C	-2.6791783	-2.5134861	2.4172545
F	-0.3943895	-2.3770884	2.7452008
C	3.7052285	-2.3297059	1.9057073
F	2.8667047	-4.1114783	0.5883729
F	4.4405750	-0.5291726	3.2521462
C	0.1699310	4.3739265	1.9024778
F	2.1299760	4.5372838	0.5817448
F	-1.7539624	4.1121900	3.2533704
C	-3.8740562	-2.0405274	1.8903278
F	-4.9919497	-0.4142726	0.5792662
F	-2.6883249	-3.5883533	3.2294270
F	4.8204186	-3.0232981	2.1832849
F	0.2138419	5.6866753	2.1791369
F	-5.0340703	-2.6586412	2.1623033
P	0.0003391	0.0004197	-1.4814924
C	-1.5489393	-0.7453755	-2.1164208
C	1.4197491	-0.9692149	-2.1175995
C	0.1294896	1.7140168	-2.1194749
C	-1.8951358	-1.9565776	-1.4904379
C	-2.3858435	-0.2366095	-3.1411984
C	2.6419662	-0.6666919	-1.4904528
C	1.3962986	-1.9457329	-3.1448740
C	-0.7425170	2.6223543	-1.4924426
C	0.9860401	2.1807659	-3.1480683
C	-3.0469061	-2.6590527	-1.8283677
H	-1.2407144	-2.3662120	-0.7348312
C	-3.5543275	-0.9514036	-3.4410952
C	-2.0984619	0.9751883	-3.9931178
H	2.6700411	0.1027658	-0.7328375
C	3.8252866	-1.3141587	-1.8295547
C	2.5986657	-2.6011678	-3.4463181
C	0.2027916	-2.2985018	-3.9979891
C	-0.7738001	3.9703777	-1.8333842
H	-1.4223730	2.2633239	-0.7336667
C	0.9522612	3.5493671	-3.4512673
C	1.8877955	1.3225548	-4.0008345
H	-3.2736766	-3.5965788	-1.3300706
C	-3.8972730	-2.1387734	-2.7993166

H	-4.2005201	-0.5650480	-4.2251975
H	-2.2292341	1.9110752	-3.4427062
H	-1.0749873	0.9703089	-4.3764485
H	-2.7812528	0.9831614	-4.8464593
H	4.7507828	-1.0448517	-1.3300035
C	3.7986810	-2.3080421	-2.8032234
H	2.5861139	-3.3519805	-4.2322539
H	-0.5436406	-2.8796913	-3.4492431
H	-0.3030737	-1.4077402	-4.3791773
H	0.5363689	-2.8922411	-4.8527148
C	0.0989880	4.4429614	-2.8087085
H	-1.4691946	4.6378466	-1.3338163
H	1.6080596	3.9127482	-4.2383204
H	2.7636069	0.9654971	-3.4517426
H	1.3684184	0.4398326	-4.3825499
H	2.2361746	1.9080650	-4.8553237
H	-4.8110503	-2.6586225	-3.0719013
H	4.7049811	-2.8402894	-3.0768068
H	0.1064307	5.4935842	-3.0837406

Bf_3 : Lewis acidic $B(C_6F_5)_3$
34
Energy = -2209.516666700

B	-0.0001185	0.0008677	0.0001180
C	-0.0000251	1.5635825	-0.0000613
C	1.3524825	-0.7826789	-0.0032034
C	-1.3526558	-0.7827436	0.0034840
C	-0.9400024	2.3142300	0.7232154
C	0.9401326	2.3137930	-0.7235636
C	2.4660378	-0.3610241	0.7387457
C	1.5356018	-1.9560748	-0.7502415
C	-1.5351825	-1.9569578	0.7494483
C	-2.4667569	-0.3604755	-0.7373566
C	-0.9483292	3.7025844	0.7446520
C	0.9486362	3.7021468	-0.7455869
C	3.6713166	-1.0501895	0.7558010
C	2.7336840	-2.6576053	-0.7793713
C	-2.7331386	-2.6587074	0.7785483
C	-3.6720062	-1.0497002	-0.7542100
C	0.0001913	4.4002103	-0.0006298
C	3.8065341	-2.2031696	-0.0150905
C	-3.8065721	-2.2035164	0.0155415
F	-1.8719039	1.6896872	1.4731158
F	1.8720534	1.6888280	-1.4730603
F	2.3843906	0.7427128	1.5111550
F	0.5324522	-2.4323071	-1.5172833
F	-0.5315345	-2.4339255	1.5153371
F	-2.3857257	0.7438532	-1.5089154
F	-1.8541446	4.3776839	1.4697240
F	1.8545142	4.3768166	-1.4709787
F	4.7029319	-0.6216728	1.5001915
F	2.8704323	-3.7619429	-1.5302561
F	-2.8692568	-3.7638736	1.5282956
F	-4.7042734	-0.6203397	-1.4971861
F	0.0002614	5.7362863	-0.0009399
F	4.9633891	-2.8718628	-0.0210318
F	-4.9633464	-2.8723049	0.0215666

B : FLP adduct Bf_3.NCClArN.PTo_3
92
Energy = -6777.505770048

Br	-6.0438728	-2.9904294	0.3475813
C	-4.2751098	-2.4719610	-0.1549512
C	-4.0123737	-2.1469915	-1.4834185
C	-3.2780067	-2.4246276	0.8159875
C	-2.7290965	-1.7379934	-1.8369592
H	-4.7943071	-2.1992610	-2.2320450
C	-2.0007863	-2.0124844	0.4467752
H	-3.4900573	-2.6996392	1.8429746
C	-1.7208497	-1.6420242	-0.8725409
H	-2.5248766	-1.4703782	-2.8664165
H	-1.2184235	-1.9620395	1.1901798
C	-0.3565088	-1.1047497	-1.2068602
Cl	0.0819791	-1.5574794	-2.9384521
N	0.2343865	0.0703931	-0.7537450
N	0.6749167	-1.2521289	-0.1787572
B	-0.2230026	1.3042280	0.1415686
P	2.2855349	-1.7462710	-0.5580183
C	0.8107328	1.2780450	1.4242689
C	-0.0561471	2.6742201	-0.7700134
C	-1.8348260	1.3062581	0.5206804
C	2.2064008	-3.5322184	-0.7652111
C	3.2368401	-1.4573738	0.9541693
C	3.0137373	-0.7968224	-1.8966524
C	0.6282227	0.4038161	2.5001684
C	1.9618830	2.0641782	1.5399010
C	0.1736571	2.7591729	-2.1441919
C	-0.2460573	3.9102406	-0.1460415
C	-2.4348494	1.5328426	1.7611017
C	-2.7490746	1.1931559	-0.5316235
C	3.3853076	-4.3106630	-0.6998640
C	0.9518860	-4.1462432	-0.9041102
C	2.9240704	-2.1675189	2.1400278
C	4.3992176	-0.6695688	0.8956125
C	3.0552479	0.5840969	-1.6213607
C	3.4314994	-1.2863909	-3.1583798
F	-0.4202954	-0.4544557	2.5169156
C	1.4516352	0.3472826	3.6176314
F	2.3305614	2.9311103	0.5588894
C	2.8280670	2.0296705	2.6307855
F	0.3151067	1.6552727	-2.9127325
C	0.2539154	3.9693079	-2.8348604
F	-0.4916771	3.9504627	1.1907930
C	-0.1811305	5.1375321	-0.7926648
F	-1.6986185	1.8080993	2.8661000
C	-3.8144969	1.5269900	1.9630569
F	-2.2867811	1.0967676	-1.8071884
C	-4.1299760	1.1865042	-0.3836347
C	3.2508619	-5.6995381	-0.7997766
C	4.7645137	-3.7260994	-0.5250376

H 0.0490114 -3.5499312 -0.9288600
C 0.8497096 -5.5295935 -0.9936997
C 3.8057802 -2.0485808 3.2216804
C 1.7250989 -3.0681671 2.3010186
H 4.6543823 -0.1542739 -0.0224380
C 5.2504326 -0.5639507 1.9890532
C 3.5368315 1.4894486 -2.5578250
H 2.7012339 0.9483275 -0.6660110
C 3.9184045 -0.3480329 -4.0755089
C 3.3537881 -2.7266052 -3.5923763
F 1.1893274 -0.5109127 4.6246680
C 2.5672877 1.1713166 3.6886712
F 3.9253729 2.8126796 2.6603253
F 0.4873306 3.9823908 -4.1657203
C 0.0773398 5.1689510 -2.1591111
F -0.3570312 6.2922195 -0.1150421
F -4.3277609 1.7246145 3.1962395
C -4.6724138 1.3367507 0.8875312
F -4.9442654 1.0513146 -1.4502828
H 4.1484714 -6.3111068 -0.7654420
C 2.0055726 -6.3083724 -0.9436755
H 5.5139356 -4.4094142 -0.9323412
H 4.8774324 -2.7584332 -1.0228213
H 4.9902953 -3.5728646 0.5364371
H -0.1257113 -5.9929946 -1.1014360
H 3.5714090 -2.5842535 4.1374562
C 4.9547896 -1.2652733 3.1570824
H 1.4092403 -3.0769053 3.3471232
H 0.8829318 -2.7477082 1.6881502
H 1.9784718 -4.0970141 2.0199809
H 6.1398633 0.0539747 1.9221598
C 3.9782194 1.0157868 -3.7905062
H 3.5549465 2.5488596 -2.3245419
H 4.2465859 -0.7045976 -5.0481097
H 3.5637695 -2.7955446 -4.6621760
H 4.0830117 -3.3522991 -3.0695859
H 2.3634897 -3.1494044 -3.4052667
F 3.3873093 1.1237621 4.7515778
F 0.1455320 6.3428402 -2.8154768
F -6.0052779 1.3256101 1.0677252
H 1.9391848 -7.3897980 -1.0158989
H 5.6132670 -1.1994546 4.0176332
H 4.3592894 1.7051996 -4.5380316

$ClBf_3^-$: borate anion $ClB(C_6F_5)_3^-$
35
Energy = -2669.945765652
B -0.0030517 0.0031651 0.6288506
C 0.9349583 -1.2710248 0.2052490
C -1.5743481 -0.1738006 0.2014945
C 0.6336648 1.4515986 0.2051427
C 2.0094367 -1.2204608 -0.6845203
C 0.7051501 -2.5432839 0.7427842
C -2.5631433 0.6614183 0.7354674
C -2.0656841 -1.1339350 -0.6849893
C 0.0504785 2.3634338 -0.6764237
C 1.8568119 1.8802970 0.7350940
C 2.8260813 -2.3106978 -0.9824948
F 2.3182173 -0.0775175 -1.3523717
C 1.4922287 -3.6570847 0.4755497
F -0.3651074 -2.7616811 1.5442618
C -3.9208003 0.5336218 0.4676731
F -2.2192533 1.7000429 1.5345161
C -3.4179168 -1.2998230 -0.9826608
F -1.2288989 -1.9742711 -1.3493041
C 0.5922067 3.6131423 -0.9755593
F -1.1018018 2.0693057 -1.3344998
C 2.4336783 3.1156855 0.4664812
F 2.5816941 1.0565259 1.5298517
C 2.5722988 -3.5406221 -0.3925381
F 3.8578540 -2.1829188 -1.8461803
F 1.2088569 -4.8558589 1.0315696
C -4.3578768 -0.4644288 -0.3964337
F -4.8189770 1.3794183 1.0193268
F -3.8210501 -2.2616701 -1.8424474
C 1.7916908 3.9988531 -0.3946282
F -0.0354332 4.4491703 -1.8322377
F 3.6201139 3.4609243 1.0139409
F 3.3486047 -4.6071164 -0.6667744
F -5.6692986 -0.6069969 -0.6704498
F 2.3332534 5.2012331 -0.6706824
Cl -0.0049199 0.0051795 2.5940980

C^+ : cation Bf_3**.**NCArN**.**PTo_3^+
91
Energy = -6317.067193502
Br -6.9975116 0.1341138 0.4244564
C -5.1243941 -0.0774415 0.3323139
C -4.3730898 0.8402285 -0.4138594
C -4.5265028 -1.1456676 1.0135355
C -2.9989453 0.6925552 -0.4751182
H -4.8628121 1.6525338 -0.9371652
C -3.1531398 -1.2950948 0.9465247
H -5.1300998 -1.8369207 1.5894224
C -2.3776195 -0.3783436 0.2068576
H -2.4023889 1.3898598 -1.0498628
H -2.6716759 -2.1043971 1.4795599
C -0.9651376 -0.4909826 0.2055363
N 0.1112458 0.0990035 -0.1603171
N 0.0337689 -1.3132037 0.7174853
B 0.8908841 1.5593111 0.0825436
P 0.4895317 -2.7821608 -0.1363766
C -0.3405940 2.5091121 0.5667407
C 2.0409560 1.2804296 1.1901481
C 1.4919107 1.9142407 -1.3913514
C 0.9181408 -2.3527567 -1.8177569
C -0.9571631 -3.8526253 -0.0857639
C 1.7544552 -3.4770106 0.9133121
C -0.7587550 3.6805361 -0.0745178
C -1.1029037 2.1656144 1.6904265

C	3.0164086	0.3161510	0.9332868
C	2.2335901	1.9970910	2.3734988
C	2.7773424	2.4021843	-1.6352834
C	0.7172042	1.7429405	-2.5396777
C	2.2070104	-2.4436719	-2.4033419
C	-0.1648420	-1.8210820	-2.5543868
C	-1.4898936	-4.2691836	1.1617736
C	-1.4945403	-4.3418339	-1.2902157
C	2.2441201	-2.7251575	1.9925224
C	2.1766562	-4.8086026	0.6947757
F	-0.0942468	4.1704350	-1.1435863
C	-1.8564522	4.4318571	0.3436336
F	-0.7696730	1.0614976	2.4079659
C	-2.2086561	2.8759313	2.1361685
F	2.9415908	-0.4023896	-0.2161143
C	4.0989888	0.0531245	1.7612473
F	1.4049541	3.0057287	2.7257383
C	3.2944504	1.7581609	3.2458148
F	3.6224197	2.6860960	-0.6154079
C	3.2763816	2.6484893	-2.9133876
F	-0.5848288	1.3617547	-2.4211627
C	1.1663481	1.9781042	-3.8316473
C	2.3319886	-2.0000432	-3.7267652
C	3.4376435	-3.0015238	-1.7364833
H	-1.1455479	-1.7405814	-2.0937887
C	-0.0041716	-1.4030615	-3.8657339
C	-2.5847909	-5.1394096	1.1355704
C	-0.9407509	-3.8485365	2.5034815
H	-1.0490778	-4.0621358	-2.2373533
C	-2.5792286	-5.2101044	-1.2790452
C	3.1879668	-3.2764012	2.8498675
H	1.8669984	-1.7250551	2.1677280
C	3.1304082	-5.3305937	1.5727379
C	1.6346177	-5.6737820	-0.4139389
F	-2.2053987	5.5512003	-0.3116357
C	-2.5939369	4.0239408	1.4498143
F	-2.9091743	2.4643995	3.2067340
F	5.0103457	-0.8815069	1.4359740
C	4.2323132	0.7770795	2.9425738
F	3.4257364	2.4780912	4.3731645
F	4.5306342	3.1050640	-3.0805838
C	2.4695611	2.4265325	-4.0229194
F	0.3577902	1.7920803	-4.8922132
H	3.3103676	-2.0599353	-4.1941954
C	1.2576590	-1.4948931	-4.4529299
H	4.3213966	-2.6956219	-2.3001635
H	3.5517879	-2.6650040	-0.7074129
H	3.4151989	-4.0963825	-1.7325732
H	-0.8470729	-0.9977680	-4.4138970
H	-3.0142479	-5.4575900	2.0812783
C	-3.1306659	-5.6030718	-0.0601572
H	-0.1004197	-4.4902265	2.7915681
H	-0.5804946	-2.8161547	2.5167507
H	-1.7127116	-3.9511507	3.2699940
H	-2.9836985	-5.5783408	-2.2157790
C	3.6354297	-4.5799676	2.6338824
H	3.5673432	-2.6942321	3.6831177
H	3.4814696	-6.3466197	1.4162656
H	1.3745899	-5.1010641	-1.3104034
H	2.3705846	-6.4291708	-0.6989378
H	0.7278587	-6.1958849	-0.0870012
F	-3.6577995	4.7290298	1.8538122
F	5.2565032	0.5355481	3.7726557
F	2.9333920	2.6547910	-5.2605596
H	1.4067061	-1.1660693	-5.4766300
H	-3.9812742	-6.2770935	-0.0375285
H	4.3752197	-5.0176163	3.2968142

C$^+$.ClBf$_3^-$: contact ion pair of **C**$^+$ and ClBf$_3^-$
126
Energy = -8987.038388664

Br	0.8922213	4.6259955	-4.6961384
C	0.3347625	3.2524715	-3.5229993
C	0.6623099	3.3423806	-2.1653263
C	-0.3886070	2.1704845	-4.0354355
C	0.2348261	2.3544658	-1.2943253
H	1.2508395	4.1751503	-1.7986868
C	-0.8086138	1.1784011	-3.1657388
H	-0.6243531	2.1154218	-5.0913255
C	-0.5224384	1.2751868	-1.7881006
H	0.5236084	2.4003236	-0.2538578
H	-1.3868926	0.3411532	-3.5397367
C	-1.0930114	0.2907214	-0.9323492
Cl	1.8672701	-0.6438193	-0.1342042
N	-1.5493344	0.0510778	0.2535880
N	-1.8074656	-0.8661427	-1.1086062
B	-2.8016951	0.6338829	1.1607205
P	-1.4177777	-2.5573479	-1.1045711
C	-4.0782629	-0.1575986	0.5342843
C	-2.4640021	0.3823197	2.7355410
C	-2.8654331	2.2655358	0.9741778
C	-0.4478737	-2.8695701	-2.5820620
C	-3.0559935	-3.2739259	-1.3132660
C	-0.7269211	-3.0844647	0.4521138
C	-4.5135947	0.1484886	-0.7612945
C	-4.8260801	-1.1525059	1.1691395
C	-1.2300562	0.0258653	3.2864138
C	-3.4478510	0.7062199	3.6738043
C	-4.0004591	3.0333617	0.6928787
C	-1.7241573	3.0198788	1.2547724
C	-0.5604852	-4.0943931	-3.2885933
C	0.4103521	-1.8556449	-3.0369389
C	-3.8318941	-2.9854928	-2.4655292
C	-3.4957407	-4.2331015	-0.3855643
C	-1.5398960	-2.7836881	1.5646105
C	0.4733545	-3.8215559	0.6105693
F	-3.8465132	1.0771270	-1.4935021
C	-5.6305720	-0.4141749	-1.3612415
F	-4.4923053	-1.6043712	2.4026099
C	-5.9510017	-1.7488576	0.6037296

F	-0.1659104	-0.2524737	2.5082466
C	-1.0040010	-0.0513607	4.6610952
F	-4.6668712	1.1175579	3.2384746
C	-3.2662448	0.6429066	5.0488906
F	-5.2106325	2.4624668	0.4943701
C	-3.9847127	4.4254800	0.6258759
F	-0.5925788	2.3790176	1.6319216
C	-1.6557064	4.4063477	1.1876124
C	0.1625018	-4.2109597	-4.4796451
C	-1.3523537	-5.2882889	-2.8158319
H	0.5748815	-0.9697188	-2.4382924
C	1.1123046	-2.0057920	-4.2267307
C	-5.0365626	-3.6791390	-2.6219232
C	-3.4207157	-2.0137474	-3.5420484
H	-2.8809197	-4.4744981	0.4731784
C	-4.7012720	-4.9009128	-0.5676846
C	-1.1915405	-3.2068825	2.8371663
H	-2.4557322	-2.2228597	1.4239858
C	0.7964226	-4.2219181	1.9125417
C	1.4073877	-4.2072407	-0.5042547
F	-6.0041589	-0.0563822	-2.6038204
C	-6.3660166	-1.3685014	-0.6659223
F	-6.6344352	-2.6923068	1.2749380
F	0.2034637	-0.4168095	5.1331733
C	-2.0268885	0.2539238	5.5498843
F	-4.2663876	0.9550761	5.8964749
F	-5.1104351	5.1064559	0.3406756
C	-2.8032296	5.1204218	0.8603351
F	-0.5029125	5.0542435	1.4369283
H	0.0976283	-5.1444399	-5.0316627
C	0.9701001	-3.1813324	-4.9607090
H	-0.9405000	-6.1945929	-3.2660179
H	-1.3206756	-5.4055757	-1.7291953
H	-2.4055348	-5.2182555	-3.1080066
H	1.7722318	-1.2141705	-4.5628596
H	-5.6437555	-3.4662244	-3.4971147
C	-5.4723217	-4.6243373	-1.6948878
H	-4.2707454	-1.7932062	-4.1904914
H	-3.0606380	-1.0735929	-3.1179371
H	-2.6208234	-2.4290599	-4.1648438
H	-5.0272059	-5.6345481	0.1617899
C	-0.0104205	-3.9279334	3.0085537
H	-1.8322372	-2.9720418	3.6801536
H	1.7165871	-4.7780038	2.0612736
H	2.2551358	-4.7524240	-0.0875248
H	0.9233743	-4.8576956	-1.2376057
H	1.7846958	-3.3237432	-1.0256843
F	-7.4491401	-1.9272472	-1.2228778
F	-1.8225249	0.1826034	6.8760997
F	-2.7751490	6.4594968	0.7865921
H	1.5072880	-3.3115234	-5.8951350
H	-6.4128586	-5.1418802	-1.8553472
H	0.2837423	-4.2653148	3.9976517
B	3.7535788	-0.0383384	0.1068564
C	3.8052471	0.3736950	1.6853275
C	4.6483401	-1.3508333	-0.2736839
C	3.9353419	1.2078956	-0.9322939
C	2.8743469	1.2820731	2.1996644
C	4.6621742	-0.1621279	2.6471334
C	4.4076044	-2.5816476	0.3501265
C	5.6732786	-1.3889287	-1.2208594
C	3.6018312	1.0781160	-2.2847443
C	4.4487199	2.4620924	-0.5952592
C	2.7258195	1.5757261	3.5480761
C	4.5571670	0.1056431	4.0116246
C	5.0730161	-3.7591050	0.0338015
C	6.3653203	-2.5479653	-1.5726182
C	3.7294475	2.0933890	-3.2243921
C	4.5878159	3.5135778	-1.5011865
C	3.5721676	0.9693325	4.4708293
C	6.0591226	-3.7482083	-0.9470475
C	4.2276056	3.3309920	-2.8301106
F	2.0624649	1.9553660	1.3516049
F	5.6851491	-0.9860801	2.2991498
F	3.5039951	-2.6685168	1.3523195
F	6.0840774	-0.2639701	-1.8614660
F	3.1712975	-0.1127521	-2.7670085
F	4.8826177	2.7266342	0.6623559
F	1.7851885	2.4452192	3.9715409
F	5.4092271	-0.4652658	4.8912801
F	4.7844861	-4.9122504	0.6782274
F	7.3351367	-2.5113423	-2.5113271
F	3.3910154	1.8852229	-4.5152628
F	5.0849600	4.7034133	-1.1027009
F	3.4516716	1.2341002	5.7850942
F	6.7149421	-4.8782012	-1.2701736
F	4.3718144	4.3286926	-3.7202996

D$^+$.ClBf$_3^-$: contact ion pair of **D**$^+$ and ClBf$_3^-$
126
Energy = -8987.147847250

Br	-6.7158712	-2.9423554	0.2069723
C	-4.9312729	-2.3465556	0.5275748
C	-4.5103235	-1.1338601	-0.0173765
C	-4.0822157	-3.1303593	1.3041688
C	-3.2025539	-0.7133067	0.2048175
H	-5.1874347	-0.5287434	-0.6094584
C	-2.7741675	-2.6999342	1.5101704
H	-4.4276511	-4.0610568	1.7389733
C	-2.3152915	-1.5033251	0.9511676
H	-2.8658129	0.2221747	-0.2246705
H	-2.1170027	-3.2895726	2.1343897
C	-0.8832803	-1.0825808	1.0952419
N	-0.6385374	0.0706249	1.7611854
N	0.1179521	-1.7621756	0.5702325
B	0.7825119	0.8080497	1.7492972
P	0.0640846	-2.8943779	-0.6180871
C	-1.7249379	0.5939437	2.5259403
C	1.9736042	-0.1500841	2.3217095
C	1.1061428	1.5875395	0.3489953

C	-0.3981364	-2.0344479	-2.1395910
C	-0.9925870	-4.3621306	-0.3789284
C	1.7111620	-3.6288677	-0.6623720
C	-2.3194697	1.8252134	2.2300099
C	-2.2485326	-0.1195457	3.6097728
C	3.1749711	-0.4308506	1.6665683
C	1.9077120	-0.6556090	3.6263724
C	2.2449417	2.4035695	0.2706750
C	0.3141995	1.6234143	-0.7991947
C	0.5448903	-1.3301592	-2.9269355
C	-1.7703703	-1.9339608	-2.4301332
C	-0.8978672	-5.0978205	0.8296929
C	-1.8152952	-4.8355980	-1.4141469
C	2.5643014	-3.3730157	0.4181006
C	2.0912674	-4.5382323	-1.6770435
F	-1.8937102	2.5466567	1.1814185
C	-3.3771541	2.3262779	2.9797454
F	-1.7403131	-1.3185732	3.9418348
C	-3.3215960	0.3565301	4.3559774
F	3.3607042	-0.1021971	0.3646676
C	4.2519677	-1.0811153	2.2643833
F	0.8010337	-0.4644365	4.3766316
C	2.9405693	-1.3519273	4.2456201
F	3.1050802	2.4711218	1.3116972
C	2.5864776	3.1666950	-0.8380469
F	-0.8225897	0.8905892	-0.8994460
C	0.6004414	2.4002806	-1.9225623
C	0.0630631	-0.6208890	-4.0329698
C	2.0203722	-1.2684432	-2.6236137
H	-2.4950379	-2.4149836	-1.7849476
C	-2.2199865	-1.2036681	-3.5241764
C	-1.7019707	-6.2334184	0.9729358
C	0.0348982	-4.7444978	1.9620984
H	-1.8385216	-4.3224238	-2.3667728
C	-2.5978386	-5.9742471	-1.2479040
C	3.8032397	-3.9988529	0.5083898
H	2.2335466	-2.6840232	1.1857362
C	3.3523978	-5.1356613	-1.5718713
C	1.2125680	-4.9083348	-2.8441492
F	-3.9158253	3.5211129	2.6771282
C	-3.8911314	1.5851891	4.0409951
F	-3.8097557	-0.3676173	5.3780579
F	5.3964152	-1.2848978	1.5788250
C	4.1348400	-1.5551731	3.5637714
F	2.8078008	-1.7981388	5.5114105
F	3.7181946	3.9016691	-0.8479959
C	1.7511479	3.1740166	-1.9498343
F	-0.2369270	2.4132122	-2.9796432
H	0.7768754	-0.0810713	-4.6491665
C	-1.2936672	-0.5603529	-4.3420096
H	2.4731728	-0.4465505	-3.1839072
H	2.2089661	-1.1033120	-1.5606183
H	2.5343496	-2.1922461	-2.9098063
H	-3.2837758	-1.1367769	-3.7292306
H	-1.6517386	-6.7866775	1.9071289
C	-2.5550085	-6.6665327	-0.0400121
H	1.0251061	-5.1797306	1.7846640
H	0.1743310	-3.6672433	2.0802465
H	-0.3436665	-5.1537812	2.9024686
H	-3.2329484	-6.3133706	-2.0600734
C	4.2011561	-4.8789108	-0.4964180
H	4.4548337	-3.7999195	1.3522685
H	3.6674857	-5.8216000	-2.3539248
H	0.6555829	-4.0537647	-3.2381730
H	1.8178405	-5.3261092	-3.6523261
H	0.4787896	-5.6668054	-2.5475591
F	-4.9296231	2.0491582	4.7501616
F	5.1556112	-2.2059652	4.1488543
F	2.0520124	3.9193533	-3.0278583
H	-1.6262825	0.0097215	-5.2041931
H	-3.1711932	-7.5479158	0.1107613
H	5.1693628	-5.3679706	-0.4439031
B	1.7440410	4.4324596	4.9003495
C	2.4279420	3.4046581	5.8696663
C	2.5417093	5.0607051	3.7054712
C	0.2763541	4.9128042	5.1934498
C	2.1529042	3.3854864	7.2461263
C	3.3576521	2.4429425	5.4385420
C	1.9298908	5.4417719	2.4982262
C	3.9260090	5.2848475	3.7607076
C	-0.1270928	6.2410245	4.9890740
C	-0.7259071	4.0572861	5.6796527
C	2.7297048	2.4840283	8.1315458
F	1.3132523	4.2946944	7.7879316
C	3.9493287	1.5223168	6.2944489
F	3.7158417	2.3689056	4.1461680
C	2.6326096	5.9646235	1.4212191
F	0.6033374	5.2990737	2.3235812
C	4.6594115	5.8167699	2.7080257
F	4.6164839	5.0139492	4.8895312
C	-1.4200575	6.6940408	5.2177053
F	0.7649313	7.1678362	4.5771266
C	-2.0327196	4.4713348	5.9047105
F	-0.4569992	2.7662712	5.9482582
C	3.6301181	1.5386476	7.6494836
F	2.4356422	2.5194035	9.4428240
F	4.8402405	0.6259155	5.8352548
C	4.0081938	6.1522391	1.5246509
F	1.9991544	6.2928648	0.2813137
F	5.9844581	6.0162638	2.8209660
C	-2.3849105	5.7974773	5.6685147
F	-1.7465141	7.9819805	5.0156231
F	-2.9589239	3.6102548	6.3618813
F	4.1987935	0.6623837	8.4850521
F	4.6979710	6.6609946	0.4983298
F	-3.6381655	6.2101312	5.8894327
Cl	0.6124630	2.2262157	3.0479920

D^+ : cation $Bf_2NfCArNPTo_3^+$

91

Energy = -6317.183001923
Br -2.8562974 6.0070192 -1.3686012
C -2.0245544 4.3608509 -0.9116044
C -1.1016600 3.7963496 -1.7947650
C -2.3579009 3.7458441 0.2949833
C -0.5118641 2.5825116 -1.4648216
H -0.8537967 4.2927187 -2.7254017
C -1.7838974 2.5183873 0.5994560
H -3.0588170 4.2096779 0.9781821
C -0.8698466 1.9214057 -0.2788994
H 0.1922239 2.1270693 -2.1533517
H -2.0335037 2.0317527 1.5364984
C -0.3316443 0.5781594 0.0086902
N 1.0687655 0.4154307 -0.0917021
N -1.0057694 -0.4622327 0.3635772
C 1.8681019 1.5733571 0.1483123
B 1.7291497 -0.8489792 -0.3836101
P -2.6209492 -0.7863857 0.5401249
C 1.7448999 2.3243840 1.3211618
C 2.8153485 1.9848863 -0.7939475
C 1.0190956 -1.9585350 -1.2209182
C 3.2055986 -1.0425810 0.0950196
C -3.7776724 0.3993479 -0.1852488
C -2.8775838 -0.8346119 2.3228356
C -2.8579426 -2.4322701 -0.1459636
F 0.8584473 1.9561840 2.2619441
C 2.5263479 3.4527191 1.5452255
F 2.9465806 1.2983610 -1.9421552
C 3.6186556 3.0996233 -0.5807705
C 0.3286866 -1.6755632 -2.4032590
C 1.1149835 -3.3113724 -0.8807542
C 4.1937882 -1.5538690 -0.7563039
C 3.6126184 -0.7662767 1.4063522
C -3.7174045 0.7743369 -1.5466295
C -4.6769857 1.0416170 0.6866902
C -1.8931871 -0.2628859 3.1442952
C -4.0210253 -1.4485059 2.8854360
C -2.1842095 -3.5523610 0.4006576
C -3.7895845 -2.5959440 -1.1838938
F 2.3882944 4.1577852 2.6743736
C 3.4698552 3.8385127 0.5929567
F 4.5205019 3.4709808 -1.4962969
F 0.2644241 -0.3996398 -2.8480271
C -0.2716124 -2.6554048 -3.1829107
F 1.7761352 -3.6754040 0.2388153
C 0.5261985 -4.3213791 -1.6317926
F 3.8904099 -1.8390531 -2.0388102
C 5.5076780 -1.7553930 -0.3509014
F 2.7109050 -0.3161906 2.3065004
C 4.9120621 -0.9672936 1.8533611
C -2.7866352 0.1376183 -2.5428297
C -4.5648192 1.7990516 -1.9824147
H -4.6998780 0.7723526 1.7371182
C -5.5174167 2.0447161 0.2222854
H -1.0051851 0.1717493 2.6994050
C -2.0313123 -0.2767374 4.5275768
C -4.1324007 -1.4446545 4.2808710
C -5.0981859 -2.1154171 2.0674344
C -2.4562970 -4.8040932 -0.1638815
C -1.2504524 -3.4758917 1.5812710
H -4.3409402 -1.7389189 -1.5535421
C -4.0331132 -3.8514317 -1.7272232
F 4.2332212 4.9136864 0.8066797
F -0.9248494 -2.3373131 -4.3104827
C -0.1777461 -3.9889470 -2.7870097
F 0.6168939 -5.6040534 -1.2509716
F 6.4276319 -2.2265881 -1.2032875
C 5.8661295 -1.4615871 0.9642909
F 5.2538233 -0.7038858 3.1226924
H -2.0953334 -0.5634574 -2.0739573
H -2.2031629 0.9004771 -3.0640544
H -3.3520880 -0.4153660 -3.3002204
H -4.5163184 2.1057825 -3.0237014
C -5.4542322 2.4299732 -1.1176287
H -6.2047480 2.5319614 0.9056029
H -1.2651540 0.1682359 5.1541053
C -3.1583323 -0.8703496 5.0957336
H -5.0068888 -1.9055391 4.7317009
H -5.3371150 -1.5607672 1.1555092
H -6.0121708 -2.2081073 2.6583469
H -4.7885906 -3.1224982 1.7656365
H -1.9467405 -5.6748410 0.2389092
C -3.3567267 -4.9591516 -1.2160786
H -0.6113741 -2.5917826 1.5463520
H -0.6210204 -4.3673375 1.6207690
H -1.8217707 -3.4323498 2.5159213
H -4.7498598 -3.9628567 -2.5338823
F -0.7563185 -4.9455339 -3.5175559
F 7.1207308 -1.6580602 1.3744529
H -6.0943131 3.2248742 -1.4875273
H -3.2792709 -0.8894737 6.1744481
H -3.5378879 -5.9470303 -1.6280489

E : B..N adduct of HBf_2 and **Rc** $ArCClN_2$
39
Energy = -4895.035671652
Br 5.5573430 0.4269997 -0.9560757
C 3.9998087 -0.4277488 -0.2671517
C 3.1913959 -1.1654160 -1.1304338
C 3.6927770 -0.2880973 1.0866414
C 2.0325421 -1.7515306 -0.6283708
H 3.4509121 -1.2734665 -2.1768880
C 2.5391197 -0.8878386 1.5811619
H 4.3381623 0.2830240 1.7438372
C 1.7000166 -1.6056685 0.7221011
H 1.3838177 -2.3089512 -1.2973755
H 2.2894896 -0.7851766 2.6320891
C 0.4052795 -2.1402435 1.2115434
N -0.8160921 -1.5084112 0.7879397
N -0.6230277 -2.6074408 0.2296764

Cl 0.3532191 -2.8854330 2.7950754
B -1.6940868 -0.1993504 0.9189594
C -0.5612333 0.9230374 0.6330023
C -3.0053559 -0.3005286 -0.0041089
C -0.0884749 1.7773894 1.6290516
C 0.0977617 1.0011603 -0.5954764
C -3.4399568 0.6854207 -0.8914806
C -3.8513752 -1.4065124 0.1082215
F -0.6458515 1.7684339 2.8599935
C 0.9658566 2.6632465 1.4224420
F -0.2865898 0.1829974 -1.6047187
C 1.1546943 1.8670956 -0.8440169
F -2.7280308 1.8249717 -1.0605835
C -4.6140064 0.5787095 -1.6330189
F -3.5292559 -2.4019339 0.9741234
C -5.0284139 -1.5579534 -0.6151834
F 1.3921950 3.4724080 2.4086025
C 1.5940122 2.7024990 0.1801538
F 1.7642531 1.8973928 -2.0416991
F -4.9858532 1.5599342 -2.4766618
C -5.4117728 -0.5525974 -1.4978054
F -5.7991353 -2.6514401 -0.4671590
F 2.6211807 3.5378186 -0.0279346
F -6.5447073 -0.6709426 -2.2078366
H -1.9584427 -0.2263390 2.0979599

F : FLP adduct HBf_2.NCArClN.PTo_3
82
Energy = -6049.887814487
Br 5.1140772 -3.4624630 1.3305334
Cl -1.2190623 -0.6132775 2.7509494
P -2.8737709 -0.8489864 -0.1366471
F 0.2696038 2.3272004 2.1067132
F 0.4659465 3.5693565 -2.4858874
F 0.8825775 -0.3293335 -3.1356164
F 2.7889288 1.5056688 0.8278255
F 5.1407923 0.3627609 0.2090537
F 3.2381052 -1.4670292 -3.7256314
F 0.2287098 4.9186558 2.7968755
F 5.3914838 -1.1445705 -2.0650913
F 0.4510740 6.1677549 -1.7712194
N -0.5942078 0.4896097 0.3143672
H -0.4400637 1.2645468 -1.7319649
F 0.3263847 6.8710822 0.8764771
N -1.1662146 -0.8683503 -0.0754776
C -0.3667226 -0.6240546 1.1085797
C 1.7132970 0.6375496 -1.1135392
C 0.9271719 -1.3672609 1.2196232
C 2.8508223 0.7807706 -0.3191409
C 0.3421885 2.8221395 -0.2280433
C 0.2957197 3.2383444 1.1012750
C 0.4028659 3.8582784 -1.1599054
C -3.5787308 0.5070637 0.8056180
C -4.3638414 0.3952789 1.9785371
C 1.9072365 -0.1215211 -2.2676478
C 1.2595065 -2.3236839 0.2561320
H 0.5519002 -2.5570588 -0.5309704
C -3.1991005 -0.5677139 -1.8907202
C 2.4983371 -2.9597292 0.2896733
H 2.7593229 -3.6957814 -0.4621709
C -3.3983946 -2.5124724 0.3013963
C -4.1158749 0.4322661 -2.2562304
H -4.6035155 1.0215368 -1.4884300
C -4.8228158 -0.9069064 2.5807601
H -5.5413387 -1.4252081 1.9389167
H -5.3086945 -0.7140054 3.5398920
H -3.9830520 -1.5865041 2.7497637
C -2.4733309 -3.3550626 0.9390517
H -1.4850489 -2.9888572 1.1883393
C 3.1170703 -0.7231410 -2.6033960
C 4.0816808 0.2027229 -0.6122884
C 1.8340080 -1.0658478 2.2396317
H 1.5786051 -0.3293810 2.9936919
C -4.6883937 -2.9801659 -0.0354884
C -2.5996186 -1.3809363 -2.8834430
C -3.1419767 1.7727739 0.3577813
H -2.5279162 1.8468064 -0.5323519
C -4.7061942 1.5822316 2.6358356
H -5.3143239 1.5158713 3.5338493
C 4.2148862 -0.5627291 -1.7656516
C 0.2815595 4.5753851 1.4903946
C 3.3961577 -2.6260775 1.3005245
C -3.4872369 2.9297419 1.0456498
H -3.1370827 3.8935374 0.6902176
C 0.3969509 5.2084132 -0.8207148
C -4.4199547 0.6709380 -3.5909636
H -5.1274643 1.4510882 -3.8530444
C -5.7215500 -2.1232536 -0.7211597
H -5.6986916 -1.0859868 -0.3709027
H -6.7222031 -2.5225110 -0.5384604
H -5.5566424 -2.1028226 -1.8041046
C -2.9274943 -1.1169114 -4.2190444
H -2.4735596 -1.7318568 -4.9916171
C 3.0778592 -1.6898464 2.2827938
H 3.7884933 -1.4437791 3.0633316
C -4.2781475 2.8316258 2.1879439
H -4.5604324 3.7251247 2.7371861
C 0.3317275 5.5709311 0.5206858
C -2.8161591 -4.6653772 1.2537275
H -2.0959563 -5.3090653 1.7484050
C -5.0057043 -4.3012113 0.2967681
H -5.9975622 -4.6742299 0.0558629
C -3.8137563 -0.1045989 -4.5784045
H -4.0392394 0.0685399 -5.6266134
B 0.2547735 1.2763635 -0.7478432
C -1.6327287 -2.5018420 -2.6002903
H -0.6231907 -2.1029107 -2.4732450
H -1.6228113 -3.2002022 -3.4411503
H -1.8883386 -3.0560105 -1.6946031
C -4.0881346 -5.1380678 0.9303057

H	-4.3684864	-6.1585762	1.1734234

G0 : contact ion pair of G^+ and $ClBf_2H^-$
106
Energy = -7531.803357819

Br	0.9078290	5.0455534	-4.2263525
C	0.3021164	3.5250312	-3.2713743
C	0.2905444	3.5656739	-1.8719689
C	-0.1260101	2.4010821	-3.9827002
C	-0.1837332	2.4695824	-1.1742580
H	0.6510002	4.4419803	-1.3460020
C	-0.5879203	1.2976184	-3.2795380
H	-0.1082436	2.3954346	-5.0658329
C	-0.6296428	1.3346561	-1.8760747
H	-0.1799171	2.4632467	-0.0917016
H	-0.9509966	0.4276794	-3.8152384
C	-1.2109377	0.2541025	-1.1507700
N	-1.8431770	-0.0010805	-0.0631617
N	-1.7765386	-0.9827042	-1.3934438
Cl	1.5461875	-0.4783608	-0.5495218
P	-1.2819499	-2.6149868	-1.0882731
C	-0.4164758	-3.0999427	-2.5814011
C	-2.9031601	-3.3882334	-0.9953763
C	-0.4033304	-2.8101886	0.4585040
C	-0.4768727	-4.4248795	-3.0744337
C	0.3215101	-2.1124669	-3.2536848
C	-3.8134956	-3.3097067	-2.0813026
C	-3.2407932	-4.0979193	0.1681243
C	-1.0683947	-2.2582366	1.5744146
C	0.8947324	-3.3677722	0.6000015
C	0.1954884	-4.6917081	-4.2714936
C	-1.1829767	-5.5549520	-2.3684806
H	0.4278492	-1.1261420	-2.8205114
C	0.9820319	-2.4121637	-4.4385868
C	-5.0553122	-3.9326853	-1.9209498
C	-3.5070109	-2.6536004	-3.4040994
H	-2.5224225	-4.1836096	0.9750033
C	-4.4857534	-4.7045335	0.2943859
C	-0.4571289	-2.2159427	2.8203919
H	-2.0681526	-1.8542702	1.4711049
C	1.4651330	-3.3289361	1.8757773
C	1.7068079	-3.9715641	-0.5138142
H	0.1691350	-5.7042360	-4.6645867
C	0.9061553	-3.7053112	-4.9548220
H	-0.7815657	-6.5108068	-2.7129697
H	-1.0610722	-5.5069553	-1.2819055
H	-2.2587827	-5.5476845	-2.5753471
H	1.5610997	-1.6448863	-4.9419342
H	-5.7682441	-3.8796448	-2.7393080
C	-5.3977567	-4.6160062	-0.7548586
H	-4.4276700	-2.5278271	-3.9780560
H	-3.0352920	-1.6766383	-3.2825811
H	-2.8273054	-3.2754953	-3.9978402
H	-4.7348907	-5.2420555	1.2032021
C	0.8203406	-2.7502323	2.9669846
H	-0.9778505	-1.7692984	3.6606984
H	2.4581984	-3.7499015	2.0038487
H	2.6352764	-4.3755780	-0.1047876
H	1.1796106	-4.7840933	-1.0201574
H	1.9630820	-3.2127354	-1.2584423
H	1.4160928	-3.9541899	-5.8804919
H	-6.3746845	-5.0820526	-0.6716671
H	1.3157962	-2.7250972	3.9329458
B	-3.1814393	0.4472032	0.7236011
C	-4.4379205	0.0270562	-0.1992009
C	-2.8780934	2.0050683	1.0622185
H	-3.1918345	-0.1884396	1.7384475
C	-4.5798959	0.3387203	-1.5518910
C	-5.4515614	-0.7812594	0.3248988
C	-3.5227741	3.1241615	0.5320741
C	-1.8769027	2.2874696	2.0014542
F	-3.6528747	1.1159400	-2.1671495
C	-5.6357703	-0.1005744	-2.3413099
F	-5.4261459	-1.1619560	1.6232639
C	-6.5320986	-1.2368920	-0.4248629
F	-4.5392969	2.9889920	-0.3501563
C	-3.1917980	4.4314912	0.8820242
F	-1.2007630	1.2698842	2.5806550
C	-1.5171584	3.5767911	2.3815273
F	-5.7088639	0.2239238	-3.6474590
C	-6.6225390	-0.8970854	-1.7700863
F	-7.4800544	-2.0164166	0.1297228
F	-3.8469735	5.4762814	0.3402722
C	-2.1787866	4.6594097	1.8071868
F	-0.5513660	3.7943180	3.2900652
F	-7.6449080	-1.3479516	-2.5159688
F	-1.8496434	5.9136684	2.1547061
B	3.3861892	0.1517172	-0.2157112
C	3.4148275	0.2312136	1.3976294
C	3.6311753	1.4928084	-1.0925923
H	4.0774244	-0.7376966	-0.6306807
C	2.5931770	1.0834193	2.1397358
C	4.1341890	-0.6914323	2.1559292
C	3.4510186	1.4521897	-2.4804661
C	4.1079647	2.7149992	-0.6162155
C	2.4794916	1.0347141	3.5238980
F	1.8368721	2.0112743	1.5020073
C	4.0489883	-0.7820931	3.5438615
F	4.9560535	-1.5906951	1.5536663
C	3.6866698	2.5274622	-3.3300006
F	3.0412541	0.3050383	-3.0791184
C	4.3557770	3.8207388	-1.4288498
F	4.3954869	2.8834730	0.6997482
C	3.2142638	0.0877772	4.2332458
F	1.6734889	1.8845906	4.1927748
F	4.7550345	-1.7128905	4.2249370
C	4.1455997	3.7287077	-2.7990483
F	3.4893090	2.4152078	-4.6616040
F	4.8187934	4.9751064	-0.9018244
F	3.1135796	0.0159308	5.5756794

F	4.4021929	4.7787006	-3.6011295

G$^+$: cation HBf_2.NCArClN.PTo_3^+
81
Energy = -5589.442194717

Br	4.4193076	-5.0141299	0.9134642
P	-2.7520367	-0.7768578	-0.8212264
F	0.1258933	1.1397710	2.4028219
F	-0.0662811	4.3943648	-1.0556229
F	1.6977157	-0.1190987	-2.4406700
F	2.7720860	2.4657004	1.4120156
F	5.3234672	1.7038988	1.1939737
F	4.2743695	-0.8841165	-2.6301504
F	-0.7575324	2.9774952	4.1716921
F	6.1108266	0.0182501	-0.8083598
F	-0.9104774	6.2292300	0.7280030
N	-0.2008387	0.2061418	-0.1406490
H	0.1165057	1.8523881	-1.5877104
F	-1.2700738	5.5410781	3.3519186
N	-1.0493788	-0.8214035	-1.1919738
C	-0.0010925	-1.0446972	-0.3008988
C	2.1082038	1.2198053	-0.5197097
C	0.9594108	-2.0336493	0.0373558
C	3.0860545	1.6443661	0.3847031
C	0.0439015	2.6847480	0.5947886
C	-0.1428236	2.3932099	1.9440418
C	-0.2192660	4.0029673	0.2316603
C	-3.0633199	0.8952827	-0.2676344
C	-3.3233603	1.2686378	1.0740737
C	2.5761593	0.3703748	-1.5290831
C	1.0054908	-3.2687877	-0.6403562
H	0.2540112	-3.5155771	-1.3809686
C	-3.5140685	-1.0516743	-2.4258275
C	2.0366333	-4.1546328	-0.3816538
H	2.0973084	-5.0986617	-0.9095105
C	-3.2738642	-2.0789675	0.3061856
C	-4.5770138	-0.2234279	-2.8285401
H	-4.8982308	0.5963937	-2.1974798
C	-3.3162484	0.3295741	2.2526388
H	-4.2220679	-0.2841133	2.2910952
H	-3.2718758	0.9112879	3.1758937
H	-2.4587845	-0.3471829	2.2376196
C	-2.3200019	-2.9332273	0.8796566
H	-1.2668090	-2.7962985	0.6778647
C	3.8956296	-0.0465795	-1.6481275
C	4.4233043	1.2606291	0.2986693
C	1.9352645	-1.7084951	1.0050983
H	1.8746112	-0.7698602	1.5443200
C	-4.6528847	-2.2583536	0.5772003
C	-3.1220565	-2.1522799	-3.2245941
C	-2.9737245	1.8736194	-1.2829632
H	-2.7078290	1.5814787	-2.2930557
C	-3.5842663	2.6235165	1.3145423
H	-3.8027359	2.9301841	2.3332490
C	4.8306847	0.4052860	-0.7205666
C	-0.5772543	3.3219331	2.8815277
C	3.0074733	-3.8087935	0.5681965
C	-3.2236571	3.2076935	-1.0022817
H	-3.1542629	3.9487693	-1.7908821
C	-0.6477451	4.9725323	1.1351509
C	-5.2371372	-0.4562515	-4.0292531
H	-6.0553703	0.1907201	-4.3272492
C	-5.7360267	-1.3813256	0.0083858
H	-5.4465531	-0.3257450	-0.0198267
H	-6.6414670	-1.4662059	0.6130327
H	-5.9852233	-1.6830285	-1.0148517
C	-3.8043169	-2.3594066	-4.4271290
H	-3.5093874	-3.1973315	-5.0523968
C	2.9605493	-2.5988039	1.2717539
H	3.7143447	-2.3649362	2.0134199
C	-3.5553138	3.5778913	0.3018127
H	-3.7703778	4.6160739	0.5349881
C	-0.8327136	4.6271988	2.4698586
C	-2.7057308	-3.9692312	1.7215546
H	-1.9552868	-4.6189939	2.1593411
C	-5.0094502	-3.3081753	1.4302516
H	-6.0619382	-3.4525028	1.6572235
C	-4.8445055	-1.5255974	-4.8332697
H	-5.3532999	-1.7176836	-5.7728434
B	0.5263960	1.5933238	-0.4865912
C	-2.0042367	-3.0865333	-2.8481696
H	-1.0338383	-2.6200150	-3.0512378
H	-2.0649078	-4.0076428	-3.4319353
H	-2.0456792	-3.3625435	-1.7881958
C	-4.0595940	-4.1562050	1.9962760
H	-4.3772309	-4.9587231	2.6544249

HBf_2Cl^- : borate anion $HB(C_6F_5)_2Cl^-$
25
Energy = -1942.325412255

B	-0.0969210	-0.2105672	0.7267231
C	0.8884310	-1.4120499	0.2621499
C	-1.6579628	-0.3128790	0.2741454
C	1.9520517	-1.1852361	-0.6107806
C	0.7965167	-2.7238720	0.7334143
C	-2.5241485	0.7368372	0.6032394
C	-2.2559203	-1.3263704	-0.4765418
C	2.8683234	-2.1660153	-0.9881069
F	2.1481000	0.0451121	-1.1558138
C	1.6862014	-3.7342281	0.3873426
F	-0.2161625	-3.0764488	1.5624999
C	-3.8712173	0.7755868	0.2604080
F	-2.0564179	1.8077525	1.2911920
C	-3.6003171	-1.3292090	-0.8472195
F	-1.5352704	-2.3913054	-0.9174665
C	2.7359084	-3.4523904	-0.4820068
F	3.8828797	-1.8823886	-1.8370548
F	1.5421646	-4.9888238	0.8724326
C	-4.4188713	-0.2727205	-0.4714950
F	-4.6523489	1.8199046	0.6180137

F	-4.1158328	-2.3482193	-1.5721983
F	3.6099217	-4.4186030	-0.8313558
F	-5.7218982	-0.2576310	-0.8182806
Cl	-0.0382732	-0.1650014	2.6847616
H	0.3421279	0.8519757	0.3762358

TS5b : PTo_3 addition to diborane $(HBf_2)_2$
91
Energy = -4118.636929818

B	0.5651351	0.0842904	-0.6765181
C	1.1393141	-1.4065370	-0.4923151
C	1.5474831	1.2283647	-1.2681569
H	0.0392183	0.4485731	0.4557938
C	0.2774296	-2.4552627	-0.1498048
C	2.4817735	-1.7828451	-0.6028335
C	2.1897286	1.0396187	-2.4995757
C	1.7734637	2.5016357	-0.7276904
C	0.6688237	-3.7756940	0.0090961
F	-1.0349356	-2.1899231	0.0665731
C	2.9234718	-3.0958921	-0.4472320
F	3.4466088	-0.8686600	-0.8394334
C	2.9540265	1.9905788	-3.1589708
F	2.0693199	-0.1468360	-3.1485222
C	2.5453650	3.4849458	-1.3458640
F	1.2206844	2.8895804	0.4386783
C	2.0124341	-4.1027090	-0.1483887
F	-0.2247185	-4.7308070	0.3245291
F	4.2305912	-3.3929176	-0.5589106
C	3.1351502	3.2387532	-2.5762122
F	3.5139046	1.7158661	-4.3514696
F	2.6985639	4.6888259	-0.7614740
F	2.4255631	-5.3677370	0.0156407
F	3.8620269	4.1825835	-3.1927475
P	-1.1342011	-0.0250932	-1.9826945
C	-2.6867261	0.5129767	-1.2012974
C	-1.3241957	-1.7124200	-2.6784613
C	-0.7996395	1.2399571	-3.2615708
C	-2.8096559	0.2948182	0.1767337
C	-3.7587976	1.0894400	-1.9230304
C	-0.1261929	-2.2626676	-3.1694323
C	-2.4985136	-2.5098115	-2.6785042
C	-0.7239491	2.5239322	-2.6869235
C	-0.5896402	1.0812592	-4.6475199
C	-3.9608913	0.6529809	0.8674240
H	-1.9935033	-0.1599571	0.7184491
C	-4.8965859	1.4674098	-1.1990308
C	-3.7846395	1.2684408	-3.4209379
H	0.7731190	-1.6673542	-3.1430896
C	-0.0590691	-3.5475136	-3.6917564
C	-2.3992798	-3.8032025	-3.2146552
C	-3.8523239	-2.1159259	-2.1418648
C	-0.4012223	3.6419704	-3.4434711
H	-0.9285668	2.6443955	-1.6276203
C	-0.2507053	2.2275084	-5.3851831
C	-0.7439804	-0.2069167	-5.4161304
H	-4.0315898	0.4668339	1.9339688
C	-5.0058100	1.2608708	0.1747823
H	-5.7264593	1.9125764	-1.7412834
H	-3.2766674	2.1884506	-3.7273369
H	-3.2977820	0.4396823	-3.9424000
H	-4.8212820	1.3238058	-3.7641037
H	0.8854046	-3.9300407	-4.0640731
C	-1.2122896	-4.3264772	-3.7181840
H	-3.2949355	-4.4184823	-3.2204530
H	-3.8055298	-1.7912340	-1.1004351
H	-4.3006700	-1.3026228	-2.7175724
H	-4.5225870	-2.9767170	-2.2027534
C	-0.1434021	3.4869571	-4.8057409
H	-0.3506133	4.6195007	-2.9738402
H	-0.0870941	2.1153064	-6.4541657
H	0.1539044	-0.8293099	-5.3449730
H	-1.5828210	-0.8069669	-5.0578055
H	-0.9064573	0.0218737	-6.4726031
H	-5.9098420	1.5613368	0.6961744
H	-1.1909304	-5.3370358	-4.1159389
H	0.1205551	4.3445343	-5.4170949
B	0.5922241	0.5241616	1.8844385
C	-0.4011818	1.5856869	2.5731245
C	0.5785847	-0.9548326	2.4765660
H	1.6872558	0.9143595	1.6406977
C	-1.2666284	2.4960517	1.9632889
C	-0.3881830	1.6570164	3.9720692
C	-0.5565709	-1.6436157	2.9114148
C	1.7881428	-1.6385025	2.6601878
C	-2.0953431	3.3651094	2.6680559
F	-1.3494749	2.5844977	0.6161355
C	-1.1869431	2.5194733	4.7161865
F	0.4505577	0.8578735	4.6796253
C	-0.5234621	-2.9315536	3.4334413
F	-1.7772616	-1.0645380	2.8221150
C	1.8688587	-2.9239118	3.1841905
F	2.9614567	-1.0602126	2.3202629
C	-2.0594237	3.3778401	4.0569632
F	-2.9230233	4.2012951	2.0121675
F	-1.1206644	2.5339339	6.0616121
C	0.7018221	-3.5791616	3.5667399
F	-1.6537867	-3.5562130	3.8104634
F	3.0561337	-3.5449179	3.3157541
F	-2.8433591	4.2185030	4.7526940
F	0.7577240	-4.8237783	4.0606416

TS5a : dissociation of diborane $(HBf_2)_2$
48
Energy = -2963.787574953

B	-0.4979467	1.2144628	-0.6713752
C	1.0364369	1.4830622	-0.6115863
C	-1.5486314	2.2696765	-0.1480662
C	1.9014143	0.8259128	-1.5028986
C	1.6585582	2.3253920	0.3230614
C	-1.4422134	3.5874445	-0.6134141

C	-2.6339204	2.0305153	0.7018066
C	3.2812449	0.9778614	-1.4725976
C	3.0356760	2.4974198	0.3887058
C	-2.3368311	4.5966488	-0.2816879
C	-3.5382175	3.0203097	1.0763932
C	3.8500475	1.8189480	-0.5172559
C	-3.3949463	4.3101391	0.5761614
F	1.4032118	-0.0065063	-2.4380436
F	0.9221931	2.9857486	1.2362115
F	-0.4345330	3.9183467	-1.4565970
F	-2.8270929	0.8123338	1.2529692
F	4.0724838	0.3188692	-2.3348817
F	3.5912728	3.2966739	1.3118942
F	-2.1927354	5.8374057	-0.7777913
F	-4.5497349	2.7389419	1.9162038
F	5.1763330	1.9651933	-0.4628992
F	-4.2684373	5.2676335	0.9149383
H	-0.8615428	0.3410743	-1.3962611
B	-0.4448044	-1.2432898	0.4322505
C	1.0721866	-1.5440316	0.5405475
C	-1.5275592	-2.2875988	0.0600752
C	1.8642767	-0.8458133	1.4654903
C	1.7601596	-2.4149989	-0.3174354
C	-1.3925903	-3.6348038	0.4495237
C	-2.7256770	-1.9794560	-0.6100257
C	3.2426032	-0.9870924	1.5412687
C	3.1407617	-2.5671329	-0.2855832
C	-2.3574530	-4.6002972	0.2021896
C	-3.6998667	-2.9270131	-0.8977863
C	3.8834122	-1.8515802	0.6535174
C	-3.5171843	-4.2440757	-0.4838667
F	1.2790990	-0.0013385	2.3372881
F	1.0892415	-3.1071602	-1.2565568
F	-0.3032600	-4.0360027	1.1344257
F	-2.9591551	-0.7322149	-1.0522945
F	3.9639961	-0.3005123	2.4410355
F	3.7666913	-3.3858536	-1.1435275
F	-2.1905639	-5.8657398	0.6159313
F	-4.8127787	-2.5875623	-1.5662291
F	5.2106592	-1.9876651	0.6986831
F	-4.4510859	-5.1623894	-0.7403150
H	-0.7831359	-0.1343654	0.7692608

$(HBf_2)_2$: diborane $(HB(C_6F_5)_2)_2$
48
Energy = -2963.821985405

B	0.0073816	0.8943817	0.0021625
C	1.3814807	1.6637188	-0.0559788
C	-1.3746838	1.6499994	0.0647080
C	2.3370647	1.4581916	-1.0530045
C	1.7036404	2.6073630	0.9211149
C	-1.7161103	2.5771659	-0.9212322
C	-2.3167689	1.4491788	1.0750742
C	3.5458903	2.1442745	-1.0881130
C	2.9010730	3.3126935	0.9231457
C	-2.9233120	3.2657278	-0.9237557
C	-3.5332176	2.1215508	1.1123394
C	3.8267260	3.0773249	-0.0919730
C	-3.8360016	3.0345701	0.1040383
F	2.0940298	0.5736275	-2.0474228
F	0.8322108	2.8382176	1.9276007
F	-0.8534284	2.8084696	-1.9353112
F	-2.0528149	0.5820033	2.0794477
F	4.4356065	1.9240660	-2.0683559
F	3.1742737	4.2096043	1.8824931
F	-3.2177746	4.1428080	-1.8951612
F	-4.4091286	1.9079324	2.1064407
F	4.9814876	3.7518118	-0.1141265
F	-5.0000890	3.6929864	0.1258352
H	0.0128982	0.0036992	-0.9789084
B	0.0073865	-0.8929953	-0.0022934
C	1.3815161	-1.6623264	0.0557163
C	-1.3745078	-1.6488913	-0.0648002
C	2.3378224	-1.4558627	1.0519114
C	1.7026085	-2.6078372	-0.9199669
C	-1.7148731	-2.5777237	0.9199875
C	-2.3173028	-1.4473397	-1.0743918
C	3.5457586	-2.1434141	1.0880972
C	2.8988866	-3.3151402	-0.9205235
C	-2.9212176	-3.2677746	0.9216389
C	-3.5335371	-2.1200501	-1.1116866
C	3.8252145	-3.0787465	0.0937083
C	-3.8344830	-3.0359609	-0.1054655
F	2.0966701	-0.5684440	2.0442639
F	0.8318037	-2.8379814	-1.9271365
F	-0.8516702	-2.8098634	1.9334205
F	-2.0539276	-0.5797022	-2.0785721
F	4.4357819	-1.9227289	2.0679538
F	3.1701426	-4.2152393	-1.8774249
F	-3.2144726	-4.1467045	1.8917150
F	-4.4113135	-1.9034900	-2.1035144
F	4.9794801	-3.7540579	0.1164690
F	-4.9970463	-3.6970078	-0.1290166
H	0.0108465	-0.0020843	0.9788535

$HBf_2.PTo_3$: Lewis adduct of HBf_2 and PTo_3
67
Energy = -2636.737871403

B	0.2696123	0.0417502	0.5706341
C	1.4991456	-0.9251891	0.9764818
C	0.2580093	1.5747574	1.0809371
H	-0.7530017	-0.4849343	0.9038536
C	1.4354499	-2.2699534	0.5964506
C	2.6573142	-0.5863835	1.6783171
C	1.1895653	2.5518152	0.7261105
C	-0.8044891	2.0507056	1.8546290
C	2.4459623	-3.1966688	0.8091394
F	0.3279348	-2.7190470	-0.0526691
C	3.6960891	-1.4826376	1.9249751
F	2.8336354	0.6639589	2.1727334

C	1.0894403	3.8917412	1.0749651
F	2.2615006	2.2161681	-0.0377696
C	-0.9478780	3.3836120	2.2330491
F	-1.7895611	1.2123691	2.2635025
C	3.5982565	-2.7944580	1.4773857
F	2.3253416	-4.4703049	0.3837932
F	4.7968687	-1.0895284	2.5995918
C	0.0051002	4.3143531	1.8368245
F	2.0188220	4.7849834	0.6791973
F	-2.0020658	3.7832429	2.9747109
F	4.5952604	-3.6690998	1.7031644
F	-0.1171491	5.6068241	2.1881593
P	0.0929179	0.0304701	-1.4920526
C	-1.5245010	-0.6186455	-2.0389620
C	1.4901023	-0.9167122	-2.1934679
C	-0.0079279	1.7741703	-2.0547140
C	-2.3227998	-1.3077209	-1.1156937
C	-1.9998182	-0.3973646	-3.3540847
C	2.7395853	-0.4417497	-1.7497930
C	1.4297329	-2.1232040	-2.9311306
C	-1.1348343	2.4001036	-1.4845044
C	0.8585000	2.5193803	-2.8796846
C	-3.5847000	-1.7799105	-1.4690563
H	-1.9508471	-1.4751461	-0.1128296
C	-3.2718047	-0.8774987	-3.6825577
C	-1.1991763	0.2999354	-4.4239996
H	2.7745642	0.4550941	-1.1441572
C	3.9249703	-1.0956978	-2.0548110
C	2.6447845	-2.7646297	-3.2227803
C	0.1688255	-2.8044313	-3.4017126
C	-1.3987963	3.7483428	-1.6843183
H	-1.8170630	1.8131935	-0.8753600
C	0.5727184	3.8827503	-3.0593116
C	2.0535199	1.9620477	-3.6098817
H	-4.1846912	-2.3154591	-0.7396523
C	-4.0634146	-1.5583004	-2.7584552
H	-3.6404985	-0.7206159	-4.6932539
H	-1.3235240	1.3864618	-4.3649631
H	-0.1283562	0.0906020	-4.3398082
H	-1.5332871	-0.0258351	-5.4128474
H	4.8704113	-0.6992341	-1.6980200
C	3.8758666	-2.2705157	-2.8035603
H	2.6075220	-3.6919779	-3.7883430
H	-0.5223506	-2.9922482	-2.5768998
H	-0.3666940	-2.2111771	-4.1468151
H	0.4273669	-3.7622290	-3.8597463
C	-0.5244620	4.5010445	-2.4689621
H	-2.2742336	4.2027936	-1.2311316
H	1.2341104	4.4634305	-3.6975035
H	2.9385973	1.9364762	-2.9652144
H	1.8857865	0.9450264	-3.9685242
H	2.2866077	2.5995820	-4.4669219
H	-5.0461538	-1.9181897	-3.0494402
H	4.7872788	-2.8084447	-3.0474886
H	-0.7053905	5.5590088	-2.6347418

HCl : acidic HCl

2

Energy = -460.8427154301

H	0.0557469	0.0000000	0.0000000
Cl	1.3442531	0.0000000	0.0000000

$HPPh_2$: phosphine

24

Energy = -805.5772230056

P	0.0865857	-1.6689018	0.4156635
C	1.4511777	-0.4611744	0.1064127
C	-1.4114247	-0.6451353	0.1686048
H	0.0927189	-2.2784920	-0.8662410
C	2.7697590	-0.9297781	0.2260347
C	1.2375618	0.8912271	-0.1907258
C	-2.0081732	-0.4676588	-1.0891539
C	-1.9912686	-0.0267301	1.2880193
C	3.8499149	-0.0713439	0.0225424
H	2.9520270	-1.9720813	0.4794864
H	0.2242766	1.2723144	-0.2782714
C	2.3201552	1.7535718	-0.3776033
H	-1.5718627	-0.9416654	-1.9643485
C	-3.1556833	0.3149901	-1.2243588
C	-3.1305401	0.7662794	1.1503531
H	-1.5470217	-0.1666106	2.2707804
H	4.8642887	-0.4513787	0.1086157
C	3.6276592	1.2751694	-0.2776368
H	2.1396198	2.8001444	-0.6076125
H	-3.6068666	0.4457874	-2.2041623
C	-3.7158866	0.9369507	-0.1063913
H	-3.5670136	1.2414176	2.0244953
H	4.4680770	1.9469296	-0.4279597
H	-4.6058163	1.5507355	-0.2136889

H^+ : cation $Bf_2NHCArNPTo3^+$

81

Energy = -5589.590590187

Br	-5.6255184	-0.4564491	-3.5312810
P	-1.0194996	-0.5631675	1.8049493
F	4.8647164	1.8927165	-0.9177244
F	0.9546545	2.7138476	-3.5537581
F	2.2466927	-1.3855379	-1.6885263
F	3.1967582	1.9760220	1.5035001
F	4.5008369	0.1489251	3.0397532
F	3.4735447	-3.2341063	-0.1146132
F	6.2961387	3.3093660	-2.6469749
F	4.6382887	-2.4586810	2.2392807
F	2.4355802	4.1148634	-5.2568845
N	0.5514746	1.4231897	-1.1924415
H	0.1582703	1.9560009	-1.9629187
F	5.1149312	4.4399984	-4.8344030
N	-0.1837401	0.4017600	0.7539732
C	-0.4353807	0.7715853	-0.4635309
C	2.6648358	0.3424743	-0.1228691

C	-1.6951496	0.5687886	-1.2121839
C	3.2542379	0.6902281	1.0875541
C	2.8181364	2.2260544	-2.1175374
C	4.2060846	2.4164749	-1.9678894
C	2.2758206	2.8367631	-3.2648786
C	0.2128097	-1.5133451	2.7103646
C	1.1268782	-0.8702474	3.5797750
C	2.7704207	-0.9927687	-0.4993919
C	-1.6357092	0.1361439	-2.5479618
H	-0.6751381	-0.0125594	-3.0312942
C	-1.8590950	0.5333366	2.9641623
C	-2.7991020	-0.1705796	-3.2437502
H	-2.7501932	-0.5406650	-4.2610934
C	-2.2313956	-1.6719868	1.0462518
C	-1.9421144	1.8948163	2.6310322
H	-1.4960870	2.2514209	1.7087203
C	1.1630412	0.6206281	3.7972148
H	0.3881373	0.9232147	4.5109248
H	0.9936430	1.1761119	2.8726382
H	2.1285115	0.9158870	4.2135494
C	-3.5634906	-1.5973011	1.4917241
H	-3.8212615	-0.9294666	2.3066559
C	3.4093127	-1.9516915	0.2806811
C	3.9262309	-0.2280699	1.8843113
C	-2.9442096	0.7494862	-0.6041475
H	-3.0062283	1.1246336	0.4116039
C	-1.8898977	-2.5082480	-0.0399653
C	-2.3802864	0.0582253	4.1905291
C	0.1910721	-2.9135142	2.6092361
H	-0.5450104	-3.3971690	1.9766641
C	2.0090285	-1.6810651	4.3030745
H	2.7192655	-1.2044801	4.9728219
C	3.9988084	-1.5601515	1.4791986
C	4.9811762	3.1536985	-2.8555658
C	-4.0280021	-0.0061925	-2.6036785
C	1.0865530	-3.6919604	3.3332175
H	1.0630640	-4.7727048	3.2406991
C	3.0173615	3.5690303	-4.1795193
C	-2.5607616	2.7976231	3.4884855
H	-2.6180842	3.8473940	3.2200654
C	-0.4907979	-2.6409491	-0.5833820
H	-0.4590754	-2.3348676	-1.6333997
H	-0.1609704	-3.6840468	-0.5406882
H	0.2344815	-2.0416370	-0.0314691
C	-3.0034843	0.9899140	5.0296976
H	-3.4203593	0.6415272	5.9707151
C	-4.1159053	0.4736686	-1.2970353
H	-5.0807583	0.6187344	-0.8260365
C	1.9998656	-3.0693252	4.1835614
H	2.7033158	-3.6636098	4.7584751
C	4.3848729	3.7332804	-3.9709238
C	-4.5595838	-2.3439220	0.8769679
H	-5.5833656	-2.2760134	1.2301065
C	-2.9167532	-3.2395487	-0.6496865
H	-2.6694901	-3.8791253	-1.4927158
C	-3.0975633	2.3385438	4.6913987
H	-3.5858898	3.0308829	5.3704224
B	1.9779149	1.3684756	-1.1074545
C	-2.2697245	-1.3738269	4.6512895
H	-2.3573467	-2.0916186	3.8319470
H	-3.0522138	-1.5927051	5.3818063
H	-1.3009782	-1.5472535	5.1342422
C	-4.2334233	-3.1631552	-0.2052526
H	-5.0054236	-3.7401874	-0.7048327

I : FLP adduct Bf3.NCArClN.HPPh2

73

Energy = -6428.280153840

Br	6.3496928	1.0890216	-1.5806309
Cl	-0.4621128	1.9140176	-2.9059249
P	-1.7005524	2.8638041	-0.1078821
F	2.4911219	-1.9892001	-0.1186795
F	4.7154620	-1.1809606	1.0664738
F	-0.4213016	-1.4400943	3.3340055
F	-0.0005863	1.1024558	2.5488167
F	0.2601545	-3.4225254	1.3757134
F	-0.6395522	-1.2180766	-2.7532424
F	-3.0482502	-0.9957323	-0.6340704
F	-5.2344582	-1.5430436	0.7808441
F	4.6684321	0.8231035	2.9364793
F	0.2607811	-5.7813439	0.0987720
F	-2.6278901	-1.9377958	4.7131617
F	2.2646318	1.9496773	3.6404349
F	-5.0763268	-1.9955472	3.4799668
F	-0.6367006	-3.5664944	-3.9929512
F	-0.1900271	-5.8913662	-2.6069164
N	-0.6887740	0.3906267	-0.6411633
H	-2.2999077	3.0532916	-1.3603863
N	-0.4047999	1.7842832	-0.0886814
C	-0.2012895	-2.1924682	-0.6081962
C	0.1864126	1.2621079	-1.3040327
C	1.0997659	-0.4749202	1.1182456
C	1.6759695	1.2091176	-1.3795947
C	2.3524778	-1.0184610	0.8179941
C	-0.9595626	4.4062456	0.3765229
C	1.1436231	0.5167994	2.1025457
C	3.5436619	-0.6145059	1.4172166
C	-1.6067405	-1.0927191	1.2755444
C	-2.8809989	-1.1770777	0.6991275
C	0.0188141	-3.4118782	0.0405478
C	-3.0235328	2.3161446	0.9433128
C	-1.5848765	-1.3920476	2.6404959
C	2.4321583	2.1157441	-0.6327026
H	1.9316595	2.8367487	0.0038825
C	-0.4207990	-2.3119593	-1.9784991
C	3.5269293	0.3973055	2.3673228
C	0.0299658	-4.6469322	-0.5941994
C	2.3076231	0.9662204	2.7141853
C	-2.7226188	-1.6854213	3.3898036
C	-1.3467582	5.5872635	-0.2741562

H	-2.0786279	5.5601270	-1.0760636
C	-4.0398775	-1.4802888	1.4063916
C	-0.7747306	6.7976898	0.1107835
H	-1.0645261	7.7137316	-0.3936839
C	-4.2491876	2.0123402	0.3283403
H	-4.3500735	2.0641915	-0.7513060
C	-0.0067126	4.4338703	1.4091676
H	0.2887559	3.5153624	1.9049497
C	3.8233480	2.0894205	-0.6908666
H	4.4128083	2.7853623	-0.1047396
C	2.3148884	0.2907831	-2.2177168
H	1.7314358	-0.3979712	-2.8181940
C	3.7047547	0.2506398	-2.2812528
H	4.2041081	-0.4714691	-2.9167737
C	-3.9642913	-1.7257552	2.7711685
C	-0.4194301	-3.5282935	-2.6611829
C	4.4423569	1.1489595	-1.5114204
C	-2.8872075	2.2522509	2.3391185
H	-1.9458611	2.5001244	2.8148235
C	0.1734917	6.8283475	1.1356619
H	0.6190587	7.7736479	1.4296726
C	-0.1946155	-4.7084445	-1.9661027
B	-0.2902892	-0.8258918	0.3092805
C	-5.3371779	1.6430133	1.1150764
H	-6.2857105	1.4074330	0.6446006
C	0.5562169	5.6503507	1.7832161
H	1.2943492	5.6799488	2.5783941
C	-3.9777721	1.8686197	3.1111034
H	-3.8785439	1.8122366	4.1902874
C	-5.2001897	1.5696328	2.5016172
H	-6.0486821	1.2775009	3.1126239

J : cyclic Bf_3.NCArNPPh$_2$
71
Energy = -5967.417344640

Br	5.8108996	3.8561566	-0.7717855
P	-2.1542541	2.1519470	-0.4406403
F	2.1976328	-3.3919816	-0.3919779
F	4.7307351	-3.2323373	0.3765394
F	0.2223726	-2.1410592	3.4011189
F	1.1567539	0.3476803	2.3822923
F	-1.3262489	-4.1130956	0.7444572
F	0.6679629	-1.1922315	-2.4559889
F	-2.8079417	-0.9243075	-0.0707950
F	-4.8169068	-0.9178108	1.7054286
F	5.5525709	-1.2984080	2.1310905
F	-1.9605151	-5.8066294	-1.2221177
F	-1.7844937	-2.0659039	5.1553370
F	3.7227893	0.4872912	3.1280138
F	-4.3274918	-1.4547022	4.3496222
F	-0.0008801	-2.8973078	-4.4048063
F	-1.3185102	-5.2298582	-3.8226557
N	-0.1566907	0.0142128	-0.2676583
N	-0.8074847	1.4198030	0.4655426
C	-0.3728043	-2.5197190	-0.7620072
C	0.4001264	1.1650499	-0.1017373
C	1.5284808	-1.5094535	0.9388233
C	1.6502268	1.8199095	-0.3024873
C	2.5034352	-2.3983367	0.4750969
C	-1.4138958	3.8301895	-0.4969940
C	2.0113391	-0.5634172	1.8513370
C	3.8426764	-2.3444257	0.8626090
C	-1.1784310	-1.4673165	1.5796849
C	-2.5025176	-1.1950997	1.2236026
C	-1.0102880	-3.7374948	-0.5210631
C	-3.3266747	2.2643861	0.9516415
C	-0.9943923	-1.7767983	2.9271570
C	1.8526444	3.1163163	0.2049180
H	1.0471508	3.6296109	0.7189814
C	-0.0407837	-2.2974939	-2.0992820
C	4.2657312	-1.3689452	1.7566616
C	-1.3375771	-4.6493895	-1.5214225
C	3.3339364	-0.4673465	2.2614622
C	-2.0271316	-1.7710377	3.8621290
C	-0.7377384	4.1810412	-1.6775182
H	-0.7316676	3.4937827	-2.5201520
C	-3.5660753	-1.1918963	2.1175837
C	-0.0728926	5.4029697	-1.7698602
H	0.4512272	5.6656496	-2.6838820
C	-4.6652919	2.5247681	0.6212742
H	-4.9541074	2.6376608	-0.4213528
C	-1.4321271	4.7271098	0.5817487
H	-1.9604463	4.4662052	1.4938250
C	3.0912044	3.7233798	0.0681533
H	3.2657106	4.7172630	0.4629507
C	2.6930138	1.1347250	-0.9571878
H	2.5224192	0.1389526	-1.3517577
C	3.9299217	1.7416695	-1.1033025
H	4.7405741	1.2315347	-1.6095253
C	-3.3226603	-1.4710511	3.4574185
C	-0.3494749	-3.1756436	-3.1323383
C	4.1141597	3.0284509	-0.5846140
C	-2.9696680	2.0931072	2.2972945
H	-1.9395167	1.8717447	2.5566673
C	-0.0856604	6.2862393	-0.6881810
H	0.4306204	7.2390428	-0.7598705
C	-1.0117858	-4.3635236	-2.8418145
B	-0.0278491	-1.4458788	0.4231492
C	-5.6293748	2.6318093	1.6224817
H	-6.6629945	2.8338421	1.3572319
C	-0.7697744	5.9504521	0.4835594
H	-0.7845393	6.6405789	1.3220985
C	-3.9402769	2.1864802	3.2942395
H	-3.6584551	2.0447061	4.3337611
C	-5.2683230	2.4589811	2.9602055
H	-6.0217064	2.5277018	3.7395515

K : open Bf_3.NCArNPPh$_2$
71
Energy = -5967.448890510

Br	1.2283519	-7.4515717	-1.0482256
P	-2.5896308	-0.6130972	-0.6887239
F	3.9845391	1.5646703	0.3944720
F	5.5222121	2.1824333	-1.6735992
F	-0.0467517	3.4500758	-1.2040873
F	0.2634657	1.0933309	-2.5546834
F	1.7009503	3.4853676	1.2560278
F	1.6678180	-1.1068369	2.5583111
F	-1.0979293	0.1569661	2.0771338
F	-3.4647975	1.3505113	2.4921540
F	4.4980400	2.2800814	-4.2152286
F	2.6110960	4.1523344	3.6963901
F	-2.3936708	4.6080899	-0.7669752
F	1.8380271	1.7255468	-4.6181538
F	-4.1386736	3.6118395	1.0898034
F	2.5837097	-0.4167195	4.9540482
F	3.0687762	2.2047531	5.5789713
N	0.8565132	-0.6221513	0.0203692
N	-1.1641437	-0.9440151	-1.1231571
C	1.6383594	1.1621283	1.7669585
C	0.0583362	-1.4119436	-0.5498327
C	2.0055704	1.3554504	-0.9522081
C	0.3252118	-2.8614487	-0.7025714
C	3.3705935	1.6163731	-0.8152835
C	-3.4927115	-1.5189285	0.5308436
C	1.5577611	1.3851766	-2.2748305
C	4.2113013	1.9330508	-1.8824450
C	-0.4641939	1.6864230	0.3583956
C	-1.3980987	1.2316448	1.3004373
C	1.8966684	2.4794390	2.1517005
C	-3.4446245	0.5908954	-1.6494691
C	-0.8596862	2.8428624	-0.3097912
C	-0.5680688	-3.7098892	-1.3678288
H	-1.4725764	-3.3055646	-1.8105168
C	1.8869347	0.2159195	2.7613385
C	3.7011881	1.9798363	-3.1725852
C	2.3712254	2.8560029	3.4014230
C	2.3540077	1.6982080	-3.3698508
C	-2.0761323	3.4929417	-0.0833049
C	-2.7440640	-2.2599407	1.4691324
H	-1.6611386	-2.2128663	1.4620385
C	-2.6209892	1.8496084	1.5674766
C	-3.4075395	-3.0407670	2.4055401
H	-2.8379369	-3.6068779	3.1352577
C	-4.5906783	1.2469303	-1.1603447
H	-4.9939846	1.0090429	-0.1829641
C	-4.8999869	-1.5927677	0.5193976
H	-5.4785789	-1.0708709	-0.2332630
C	-0.3066171	-5.0747443	-1.4781968
H	-0.9979943	-5.7297588	-1.9963863
C	1.4958240	-3.3970961	-0.1455462
H	2.1768997	-2.7285771	0.3711892
C	1.7702807	-4.7562255	-0.2432812
H	2.6724834	-5.1715602	0.1919824
C	-2.9638610	2.9989012	0.8638153
C	2.3639141	0.5457829	4.0307028
C	0.8606804	-5.5791309	-0.9107228
C	-2.8891409	0.9497108	-2.8929679
H	-1.9980911	0.4463044	-3.2485179
C	-4.8050396	-3.0985342	2.4075032
H	-5.3174242	-3.7112438	3.1425617
C	2.6090569	1.8732123	4.3567577
B	0.9934504	0.8735276	0.2694293
C	-5.1798155	2.2485561	-1.9234809
H	-6.0556647	2.7645423	-1.5448372
C	-5.5458812	-2.3851589	1.4616956
H	-6.6288577	-2.4516635	1.4517113
C	-3.4907576	1.9535108	-3.6410355
H	-3.0685463	2.2336672	-4.6005226
C	-4.6332627	2.6005804	-3.1600894
H	-5.0952819	3.3871439	-3.7483890

L : cyclic $BF_3NCArNPPh_2$
71
Energy = -5967.505092714

Br	6.6976228	0.3102874	1.7074926
P	-1.4262928	-0.7403852	2.1036965
F	2.0616298	1.3336946	-2.2666643
F	4.1863465	-0.0720635	-2.9329623
F	-1.0297961	-2.0031068	-2.5205066
F	0.2303218	-2.5187291	-0.1206851
F	-0.6881406	1.4142423	-3.1862279
F	0.1848974	2.7295085	1.3183806
F	-2.8488048	1.6001755	0.0310496
F	-5.2909488	0.9170744	-0.7794029
F	4.4368382	-2.7016012	-2.1969341
F	-0.8465522	3.9985243	-3.8893377
F	-3.4776758	-2.6689529	-3.2810469
F	2.4138025	-3.8932586	-0.7699282
F	-5.6532430	-1.2373316	-2.4456825
F	0.0343216	5.2748424	0.5874654
F	-0.4854314	5.9693683	-2.0149252
N	-0.3620982	0.0156941	0.9903254
N	-0.0239749	-0.8023320	3.0360299
C	-0.2615234	1.9224634	-0.8999583
C	0.5938952	-0.2536248	1.9722540
C	0.9857572	-0.5150791	-1.1661481
C	2.0363735	-0.0540122	1.8976650
C	2.0461046	0.0276144	-1.8977137
C	-2.7346110	0.2875594	2.7239006
C	1.1814606	-1.8580945	-0.8254209
C	3.1944110	-0.6798681	-2.2515938
C	-1.7922054	-0.2009395	-1.1357350
C	-2.9438214	0.5085910	-0.7682237
C	-0.5195167	2.3424941	-2.2086329
C	-2.0311299	-2.3678894	1.7073796
C	-2.0384787	-1.2593201	-2.0143142
C	2.8422142	-0.8454625	2.7336324
H	2.3706374	-1.5539242	3.4061596
C	-0.0782483	2.9682485	0.0020802

C	3.3337854	-2.0072378	-1.8724579
C	-0.6012185	3.6707174	-2.6048217
C	2.3081793	-2.6036448	-1.1470606
C	-3.3110946	-1.6192736	-2.4542204
C	-2.4560565	1.6442060	2.9706508
H	-1.4789970	2.0491216	2.7329606
C	-4.2335296	0.1898827	-1.1869694
C	-3.4438323	2.4611518	3.5047440
H	-3.2338021	3.5094297	3.6921228
C	-3.1589730	-2.5610901	0.8935471
H	-3.7008619	-1.7086207	0.5000139
C	-3.9965213	-0.2447087	3.0347089
H	-4.2045516	-1.2967986	2.8762889
C	4.2267615	-0.7429700	2.6842501
H	4.8494394	-1.3669559	3.3148677
C	2.6380150	0.8679362	1.0294548
H	2.0323918	1.5044296	0.3986549
C	4.0216318	0.9899075	0.9799671
H	4.4871890	1.7038779	0.3103256
C	-4.4212820	-0.8944138	-2.0364898
C	-0.1491879	4.3170554	-0.3441872
C	4.7996825	0.1733651	1.8007049
C	-1.3322367	-3.4726235	2.2216520
H	-0.4648790	-3.3132679	2.8524774
C	-4.7073750	1.9353929	3.7949808
H	-5.4781735	2.5801701	4.2062011
C	-0.4148599	4.6749930	-1.6589363
B	-0.3195116	0.3040190	-0.5662121
C	-3.5750944	-3.8516542	0.5861038
H	-4.4430995	-4.0010562	-0.0485082
C	-4.9809292	0.5867305	3.5646745
H	-5.9582914	0.1784203	3.8009141
C	-1.7630922	-4.7604056	1.9134748
H	-1.2267417	-5.6154207	2.3125777
C	-2.8774800	-4.9506350	1.0943246
H	-3.2061115	-5.9568311	0.8526420

$ArCClN_2H^+$: *N*-protonated **Rc**
16
Energy = -3413.482575780

Br	3.3592126	0.2107075	0.0634412
C	1.4790947	0.0253333	-0.0501118
C	0.8868402	-1.1675900	0.3822116
C	0.7186498	1.0771753	-0.5630450
C	-0.4911898	-1.3063551	0.3038103
H	1.4954163	-1.9724062	0.7771230
C	-0.6595367	0.9328233	-0.6459339
H	1.1923693	1.9933317	-0.8946413
C	-1.2711897	-0.2595531	-0.2066046
H	-0.9577633	-2.2243117	0.6417456
H	-1.2440464	1.7524145	-1.0488451
C	-2.7370059	-0.3651213	-0.2568320
N	-3.4391508	0.4172388	-1.2265833
N	-3.5033660	0.9638628	-0.1134529
Cl	-3.5536304	-1.7669331	0.3139158

H	-3.7118904	0.6831926	-2.1899823

PTo_3 : phosphine
43
Energy = -1154.805497735

P	-0.0357434	-0.0575683	-1.0628564
C	1.5464831	-0.7294920	-0.4161180
C	-1.2851260	-1.1640361	-0.2963777
C	-0.2325105	1.4969806	-0.1049320
C	2.7587436	-0.2007061	-0.9189466
C	1.5857886	-1.7855910	0.5055163
C	-2.0592007	-0.7649900	0.8025806
C	-1.5127022	-2.4313981	-0.8829701
C	-1.2980537	2.3655021	-0.4405093
C	0.6744941	1.8745347	0.8956171
C	2.7677838	0.9540440	-1.8874156
C	3.9670950	-0.7516442	-0.4766732
H	0.6542092	-2.1871690	0.8936979
C	2.7990316	-2.3256862	0.9303430
H	-1.8780431	0.2052666	1.2560612
C	-3.0536649	-1.5932589	1.3214240
C	-2.5107571	-3.2518367	-0.3447000
C	-0.6841103	-2.9172436	-2.0443029
C	-1.4156052	3.5808817	0.2438859
C	-2.3130210	1.9909754	-1.4897297
H	1.4909561	1.2059592	1.1526072
C	0.5447301	3.0919369	1.5629342
H	2.0094202	0.8177308	-2.6678110
H	3.7486404	1.0619723	-2.3583531
H	2.5299310	1.8953320	-1.3758672
H	4.9003331	-0.3467012	-0.8620081
C	3.9958889	-1.8059734	0.4368393
H	2.8073728	-3.1453162	1.6436401
H	-3.6450824	-1.2634140	2.1711816
C	-3.2804644	-2.8432095	0.7450620
H	-2.6878995	-4.2262249	-0.7947311
H	-0.5524233	-2.1249298	-2.7911617
H	-1.1501734	-3.7833549	-2.5219510
H	0.3203487	-3.2098461	-1.7131620
H	-2.2345498	4.2497654	-0.0117805
C	-0.5056331	3.9496253	1.2353984
H	-1.8245625	1.5587533	-2.3714047
H	-2.8976106	2.8623036	-1.7973280
H	-3.0076269	1.2316523	-1.1087806
H	1.2588456	3.3658046	2.3347465
H	4.9480207	-2.2188501	0.7588733
H	-4.0532038	-3.4976031	1.1392515
H	-0.6168185	4.9019396	1.7467337

Rc : diazirine $(BrC_6H_4)CClN_2$
15
Energy = -3413.112367427

Br	3.4178084	0.0003254	0.0549467
C	1.5083902	0.0006801	-0.0381068
C	0.8328643	-1.2180061	-0.0811362

C	0.8324264	1.2194501	-0.0635019
C	-0.5592631	-1.2106987	-0.1464527
H	1.3786140	-2.1546951	-0.0648673
C	-0.5598690	1.2124140	-0.1292330
H	1.3777102	2.1560817	-0.0334983
C	-1.2582702	0.0009924	-0.1715127
H	-1.0990521	-2.1523425	-0.1820738
H	-1.1000246	2.1542433	-0.1523443
C	-2.7404848	0.0013740	-0.2372835
N	-3.4671533	-0.5741981	-1.3667913
N	-3.4681030	0.6687728	-1.3140507
Cl	-3.5896757	-0.0671334	1.3227357

TS1 : PTo_3 addition to adduct **Rc.**Bf_3

92

Energy = -6777.475465596

Br	-6.0964530	-3.8142304	0.2521276
C	-4.3661531	-3.1106613	-0.1412582
C	-3.9251718	-3.0843904	-1.4623172
C	-3.5770324	-2.6276382	0.9012052
C	-2.6706895	-2.5486916	-1.7457947
H	-4.5491846	-3.4671459	-2.2616674
C	-2.3315865	-2.0853368	0.6028683
H	-3.9289523	-2.6633593	1.9259406
C	-1.8718651	-2.0355115	-0.7201858
H	-2.3267979	-2.5169261	-2.7731315
H	-1.7179391	-1.6921047	1.4033649
C	-0.5606792	-1.3745940	-0.9846351
Cl	0.1536653	-1.7368656	-2.5654288
N	-0.2269885	-0.1595452	-0.3482160
N	0.3676985	-1.1998875	0.1751564
B	-0.5536652	1.3062282	0.2386047
P	2.8048803	-1.4859173	-0.4086591
C	0.4770676	1.3932815	1.5097308
C	-0.3577489	2.4984831	-0.8587948
C	-2.1663998	1.2699982	0.5744837
C	3.1472444	-3.2608853	-0.6201590
C	3.8270262	-0.9935030	1.0280762
C	3.3169959	-0.4624674	-1.8150057
C	0.3270568	0.5885561	2.6448285
C	1.6017241	2.2216480	1.5573007
C	0.0914393	2.3826174	-2.1713079
C	-0.7008075	3.7968454	-0.4739695
C	-2.7878486	1.5875566	1.7827749
C	-3.0474172	0.9364556	-0.4588429
C	4.4486437	-3.7972360	-0.7397802
C	2.0321861	-4.1121621	-0.5829786
C	3.5185838	-1.5448861	2.2938595
C	4.9207856	-0.1196240	0.9025574
C	3.3777850	0.9228190	-1.5531185
C	3.4894828	-0.9203980	-3.1470958
F	-0.7027229	-0.2904599	2.7278518
C	1.1663598	0.6286809	3.7511127
F	1.9270892	3.0282356	0.5174673
C	2.4731904	2.2947503	2.6430772
F	0.4228016	1.1777481	-2.6821671
C	0.2113906	3.4663692	-3.0397936
F	-1.1246783	4.0239929	0.7963426
C	-0.5992595	4.9065195	-1.3031636
F	-2.0731440	2.0333963	2.8419584
C	-4.1649076	1.4988264	1.9792955
F	-2.5572759	0.6888765	-1.7016701
C	-4.4242653	0.8303869	-0.3087264
C	4.5747126	-5.1883156	-0.8330617
C	5.6849182	-2.9381235	-0.7726690
H	1.0410243	-3.6838030	-0.4781932
C	2.1826521	-5.4924649	-0.6759386
C	4.3147270	-1.1903919	3.3889335
C	2.3828061	-2.5154175	2.4830335
H	5.1859393	0.2785605	-0.0698684
C	5.6922478	0.2245541	2.0072843
C	3.6343724	1.8442363	-2.5609987
H	3.2366200	1.3064488	-0.5492402
C	3.7433517	0.0340607	-4.1403827
C	3.4403299	-2.3674935	-3.5714483
F	0.9455747	-0.1701815	4.8141192
C	2.2489362	1.5005495	3.7583135
F	3.5296940	3.1291255	2.6151263
F	0.6530869	3.2889188	-4.3014772
C	-0.1342255	4.7394223	-2.6041768
F	-0.9326375	6.1363141	-0.8617916
F	-4.7076626	1.8051773	3.1744390
C	-4.9898139	1.1033461	0.9321983
F	-5.2097097	0.4686429	-1.3412057
H	5.5691044	-5.6153067	-0.9383577
C	3.4633178	-6.0304438	-0.8025224
H	6.5076397	-3.4729551	-1.2543905
H	5.5148265	-2.0006788	-1.3103906
H	6.0010023	-2.6733637	0.2428258
H	1.3112476	-6.1395596	-0.6493212
H	4.0729607	-1.6061222	4.3639058
C	5.3851939	-0.3092592	3.2591383
H	1.9114282	-2.3881010	3.4604844
H	1.6389509	-2.3568195	1.7030710
H	2.7373397	-3.5502757	2.4079459
H	6.5306768	0.9038391	1.8870014
C	3.8193387	1.3975440	-3.8671045
H	3.6760327	2.9025599	-2.3225509
H	3.8790334	-0.3141792	-5.1612614
H	3.3577853	-2.4254728	-4.6597850
H	4.3520334	-2.8989709	-3.2773141
H	2.5998935	-2.9060208	-3.1308797
F	3.0641872	1.5660760	4.8213367
F	-0.0245797	5.7964117	-3.4287840
F	-6.3175190	1.0055066	1.1111790
H	3.5993300	-7.1054189	-0.8789663
H	5.9747714	-0.0393730	4.1301937
H	4.0096953	2.1030501	-4.6702721

TS2 : Cl abstraction from **B** with Bf_3

126
Energy = -8987.027378092

Br	0.5424490	6.1788752	-2.1349874
C	0.2940360	4.3488378	-1.6795761
C	0.6052073	3.9191482	-0.3884974
C	-0.1853723	3.4640933	-2.6428231
C	0.4169908	2.5852530	-0.0486936
H	0.9927686	4.6175678	0.3443319
C	-0.3892638	2.1346270	-2.2897010
H	-0.4104893	3.8054152	-3.6463684
C	-0.1125750	1.6921270	-0.9881742
H	0.7030842	2.2492223	0.9368544
H	-0.7964888	1.4445354	-3.0181948
C	-0.5200570	0.3256414	-0.6526669
Cl	1.4629811	-0.7201206	-0.1580103
N	-1.4672931	-0.2415963	0.0679987
N	-1.3181781	-0.5131588	-1.4715367
B	-2.8643570	0.1541112	0.7941832
P	-1.0218584	-2.1355753	-1.9912747
C	-3.9860018	-0.3205086	-0.2937109
C	-2.9116412	-0.6070104	2.2472753
C	-2.9399079	1.7607625	1.1659695
C	0.1256406	-2.0060648	-3.3723108
C	-2.6110277	-2.6321786	-2.6980199
C	-0.6096926	-3.2620471	-0.6640930
C	-4.1673802	0.4235936	-1.4653300
C	-4.8356839	-1.4216018	-0.1797760
C	-1.8420853	-1.2074044	2.9170458
C	-4.0946736	-0.5462410	2.9886776
C	-3.9858839	2.6514029	0.9025773
C	-1.9287281	2.3052578	1.9599138
C	0.1392445	-2.9858476	-4.3953481
C	0.9476634	-0.8725860	-3.4597463
C	-3.1324395	-1.9649208	-3.8350471
C	-3.2202366	-3.8132615	-2.2385877
C	-1.5755584	-3.3149776	0.3598370
C	0.5502966	-4.0701787	-0.6015979
F	-3.3774564	1.4962808	-1.7121967
C	-5.1609468	0.1703447	-2.3998619
F	-4.7308272	-2.2884683	0.8596559
C	-5.8387162	-1.7224077	-1.0987224
F	-0.6116548	-1.2759366	2.3671548
C	-1.9520711	-1.7562268	4.1952764
F	-5.1844014	0.0616800	2.4512374
C	-4.2483240	-1.0754428	4.2633456
F	-5.1082895	2.2608644	0.2533023
C	-3.9800665	3.9857970	1.3063818
F	-0.9138748	1.5062252	2.3676300
C	-1.8710753	3.6293078	2.3768721
C	0.9776965	-2.7585726	-5.4916345
C	-0.6616612	-4.2642004	-4.3639152
H	0.9812221	-0.1612517	-2.6469217
C	1.7648172	-0.6733916	-4.5660722
C	-4.2533487	-2.5246143	-4.4604017
C	-2.5343183	-0.7210856	-4.4417779
H	-2.8016739	-4.3413093	-1.3907048
C	-4.3379205	-4.3385235	-2.8750995
C	-1.4186184	-4.1681869	1.4429240
H	-2.4573035	-2.6877213	0.3040335
C	0.6793997	-4.9122430	0.5083085
C	1.6425567	-4.0766207	-1.6347942
F	-5.3045281	0.9500317	-3.4900573
C	-6.0111559	-0.9142924	-2.2135938
F	-6.6314465	-2.7963537	-0.9216010
F	-0.8864126	-2.3387366	4.7817462
C	-3.1617357	-1.6940667	4.8746388
F	-5.4289101	-0.9974706	4.9108290
F	-5.0155370	4.7944271	1.0039598
C	-2.9096785	4.4876974	2.0371734
F	-0.8266409	4.0819802	3.0985343
H	1.0101897	-3.5062411	-6.2794118
C	1.7715075	-1.6172745	-5.5913938
H	-0.1517046	-5.0286871	-4.9555194
H	-0.7958731	-4.6521872	-3.3505769
H	-1.6595463	-4.1193659	-4.7922800
H	2.3958413	0.2074749	-4.6158123
H	-4.6624075	-2.0192406	-5.3309434
C	-4.8520056	-3.6937501	-3.9989252
H	-3.3127196	-0.1408960	-4.9423337
H	-2.0628674	-0.0882142	-3.6900566
H	-1.7786907	-0.9816445	-5.1916650
H	-4.7941758	-5.2482755	-2.4997142
C	-0.2809576	-4.9692807	1.5162086
H	-2.1745481	-4.1983099	2.2203790
H	1.5666735	-5.5344721	0.5789709
H	2.4508629	-4.7263834	-1.2993778
H	1.2938374	-4.4569286	-2.5979083
H	2.0382295	-3.0704494	-1.7979546
F	-6.9689088	-1.1885479	-3.1128000
F	-3.2813993	-2.2176875	6.1078265
F	-2.8872354	5.7747643	2.4206414
H	2.4042494	-1.4757503	-6.4623307
H	-5.7184024	-4.0965761	-4.5141752
H	-0.1386958	-5.6403236	2.3578657
B	3.7736540	-0.0860881	0.6291759
C	3.5590148	-0.4466381	2.1631362
C	4.5630478	-1.1449650	-0.2601331
C	3.7605849	1.4324095	0.1636614
C	2.4958950	0.0784135	2.9077420
C	4.3898422	-1.3171063	2.8810675
C	4.3371047	-2.5257551	-0.1467538
C	5.6035403	-0.8003365	-1.1342153
C	3.5479984	1.8074295	-1.1709452
C	4.0592086	2.4963420	1.0241948
C	2.2109622	-0.2795396	4.2186020
C	4.1511310	-1.6933852	4.1986994
C	5.0561632	-3.4842367	-0.8487824
C	6.3363965	-1.7301012	-1.8665854
C	3.6219272	3.1154223	-1.6275038
C	4.1397003	3.8225660	0.6066754

C 3.0456331 -1.1820616 4.8705544
C 6.0568085 -3.0841713 -1.7294815
C 3.9261844 4.1356169 -0.7305644
F 1.6829557 0.9934797 2.3512154
F 5.5093595 -1.8317026 2.3193717
F 3.3986023 -2.9916811 0.7017511
F 5.9950653 0.4854854 -1.2813344
F 3.2782596 0.8698678 -2.1032594
F 4.3317085 2.2764956 2.3297548
F 1.1596323 0.2506428 4.8664447
F 4.9854790 -2.5398544 4.8325107
F 4.8113658 -4.7979512 -0.6764943
F 7.3223886 -1.3308328 -2.6906413
F 3.4061833 3.4064615 -2.9234820
F 4.4342934 4.8007904 1.4825861
F 2.8006223 -1.5374447 6.1398092
F 6.7559809 -3.9961640 -2.4188913
F 4.0058839 5.4054203 -1.1493859

TS3^{+} : 1,2-aryl shift within **C**$^{+}$
91
Energy = -6317.064169552
Br -6.9039709 -0.1806534 0.7620580
C -5.0168791 -0.2731251 0.6358549
C -4.3502734 0.6017537 -0.2296290
C -4.3290383 -1.2128120 1.4098583
C -2.9684114 0.5375888 -0.3162532
H -4.9072111 1.3127509 -0.8281187
C -2.9474274 -1.2784746 1.3116367
H -4.8659504 -1.8720834 2.0815919
C -2.2592243 -0.4044019 0.4534518
H -2.4368813 1.1949066 -0.9946789
H -2.3970166 -1.9808933 1.9241416
C -0.8209776 -0.4538580 0.3928623
N 0.0670716 0.3338347 -0.0836014
N 0.0918906 -1.3816664 0.8296856
B 0.9187998 1.6611181 -0.0305505
P 0.4749739 -2.8156145 -0.0034052
C -0.3711698 2.5836005 0.4178115
C 2.0873267 1.4954077 1.0836593
C 1.4900520 1.9391003 -1.5278664
C 0.8231680 -2.4180529 -1.7181253
C -0.9587425 -3.9050036 0.1179442
C 1.8016270 -3.5159725 0.9669968
C -0.9340986 3.6154166 -0.3514647
C -1.0266794 2.3319815 1.6383906
C 3.0294951 0.4810872 0.9136626
C 2.3115113 2.3319842 2.1759466
C 2.7673182 2.4299136 -1.8108900
C 0.7139624 1.6668018 -2.6585285
C 2.0814359 -2.5282870 -2.3605583
C -0.2936787 -1.9093512 -2.4161726
C -1.4221814 -4.3144120 1.3951727
C -1.5678929 -4.3939732 -1.0519365
C 2.3771795 -2.7440775 1.9878214
C 2.1994961 -4.8540813 0.7466803
F -0.3915930 3.9961240 -1.5217352
C -2.0420204 4.3473514 0.0640489
F -0.5738898 1.3551362 2.4534349
C -2.1388282 3.0325911 2.0788421
F 2.9261405 -0.3419830 -0.1602536
C 4.1167806 0.2828244 1.7544929
F 1.5057102 3.3924719 2.4166493
C 3.3769466 2.1602517 3.0575737
F 3.6090020 2.7998264 -0.8174654
C 3.2599985 2.5904873 -3.1051223
F -0.5804397 1.2788881 -2.5109644
C 1.1579605 1.8178708 -3.9643711
C 2.1382093 -2.1461218 -3.7083739
C 3.3524969 -3.0309865 -1.7251604
H -1.2468937 -1.7998352 -1.9060415
C -0.2009055 -1.5501435 -3.7520594
C -2.5171506 -5.1837609 1.4351289
C -0.7943611 -3.8967052 2.7024084
H -1.1784301 -4.1172968 -2.0241069
C -2.6493392 -5.2626070 -0.9758662
C 3.3732828 -3.2856716 2.7907937
H 2.0255076 -1.7333028 2.1585029
C 3.2065430 -5.3672650 1.5689966
C 1.5873819 -5.7337738 -0.3135958
F -2.5165205 5.3494408 -0.6873022
C -2.6571987 4.0490900 1.2792997
F -2.7224196 2.7363208 3.2485248
F 5.0078403 -0.6966616 1.5171516
C 4.2842096 1.1266605 2.8490526
F 3.5416423 2.9904153 4.1013732
F 4.5049311 3.0556067 -3.3093608
C 2.4538588 2.2738507 -4.1917279
F 0.3505004 1.5496949 -5.0071091
H 3.0931892 -2.2226382 -4.2200460
C 1.0267883 -1.6770667 -4.4016314
H 4.2024202 -2.7417718 -2.3469281
H 3.5079858 -2.6348041 -0.7226272
H 3.3530468 -4.1240006 -1.6581207
H -1.0700946 -1.1640699 -4.2725578
H -2.8922192 -5.4985355 2.4048518
C -3.1316037 -5.6500472 0.2742318
H 0.0467437 -4.5543155 2.9497949
H -0.4112247 -2.8722994 2.6882477
H -1.5263666 -3.9778784 3.5098393
H -3.1056930 -5.6352379 -1.8867991
C 3.7895387 -4.5998982 2.5771878
H 3.8196219 -2.6868549 3.5776698
H 3.5392237 -6.3890258 1.4092956
H 1.3020980 -5.1758839 -1.2117911
H 2.2934829 -6.5125915 -0.6109517
H 0.6841378 -6.2265177 0.0641967
F -3.7267756 4.7379733 1.6770240
F 5.3168612 0.9511170 3.6853764
F 2.9087207 2.4215268 -5.4435920

H	1.1216432	-1.3975670	-5.4461365
H	-3.9823509	-6.3203948	0.3476513
H	4.5684621	-5.0313095	3.1982332

TS4 : Cl^{-} transfer to cation **D**$^{+}$
126
Energy = -8987.137648203

Br	-5.1383632	-6.2887394	-0.7886971
C	-3.7062621	-5.0673540	-0.4853979
C	-3.2982285	-4.2257580	-1.5204843
C	-3.0961574	-5.0318130	0.7657728
C	-2.2423622	-3.3475155	-1.2987642
H	-3.7909715	-4.2571786	-2.4854709
C	-2.0335899	-4.1561958	0.9696712
H	-3.4380525	-5.6731212	1.5696485
C	-1.5837933	-3.3231302	-0.0598878
H	-1.9133444	-2.7019578	-2.1035846
H	-1.5755732	-4.1025259	1.9478640
C	-0.3806547	-2.4571682	0.1328501
N	-0.5385232	-1.1108515	0.0108835
N	0.8258445	-2.9259974	0.3706504
B	0.7435318	-0.2188408	-0.0979453
P	1.4101371	-4.4405229	0.0840558
C	-1.8818730	-0.6210641	-0.0078473
C	1.6471476	-0.1252201	1.2092299
C	1.3870627	-0.0735734	-1.5489355
C	1.4574631	-4.6267428	-1.7124695
C	0.5525511	-5.8544574	0.8451856
C	3.0259159	-4.4839652	0.8792283
C	-2.4373084	0.0064932	-1.1285831
C	-2.7196531	-0.8053814	1.0982544
C	3.0411632	-0.2591335	1.1994489
C	1.1018352	0.1442432	2.4727281
C	2.3664978	0.8997387	-1.8128580
C	1.0290522	-0.8352959	-2.6692506
C	2.5231562	-4.1316705	-2.5032606
C	0.2944613	-5.1216208	-2.3301477
C	0.2780555	-5.8328651	2.2362622
C	0.2602103	-7.0074295	0.0977197
C	3.3467775	-3.4482890	1.7652425
C	3.8981106	-5.5854257	0.7149360
F	-1.7054474	0.1928701	-2.2362669
C	-3.7558911	0.4447628	-1.1421021
F	-2.2627358	-1.4257136	2.1991537
C	-4.0479878	-0.3920001	1.0925320
F	3.6968876	-0.6229429	0.0743470
C	3.8415944	-0.0686061	2.3203667
F	-0.2277872	0.2894331	2.6247528
C	1.8657289	0.3114016	3.6225411
F	2.7758131	1.7301201	-0.8381320
C	2.9581630	1.0882108	-3.0548343
F	0.1282294	-1.8383510	-2.5795896
C	1.5710270	-0.6518242	-3.9388232
C	2.3979670	-4.2356673	-3.8934835
C	3.7569194	-3.4705501	-1.9439199
H	-0.5467127	-5.4340771	-1.7238210
C	0.1939524	-5.1959516	-3.7143874
C	-0.3572539	-6.9454676	2.7972059
C	0.6526644	-4.6939045	3.1518800
H	0.5350065	-7.0614870	-0.9477129
C	-0.3629017	-8.1043088	0.6846077
C	4.5326242	-3.4774437	2.4911353
H	2.6469398	-2.6294372	1.8800430
C	5.0952139	-5.5758683	1.4392064
C	3.6014725	-6.7588749	-0.1823284
F	-4.2512965	1.0586064	-2.2289965
C	-4.5728222	0.2311720	-0.0347506
F	-4.8255273	-0.6038837	2.1663498
F	5.1800647	-0.1861421	2.2344914
C	3.2510064	0.2114950	3.5467938
F	1.2789606	0.5934258	4.7998635
F	3.9081409	2.0246806	-3.2284874
C	2.5496690	0.3132584	-4.1356882
F	1.1606313	-1.4062672	-4.9738069
H	3.2112791	-3.8666610	-4.5123038
C	1.2617796	-4.7667602	-4.4993441
H	4.3065559	-2.9813441	-2.7516121
H	3.5061613	-2.7176275	-1.1929953
H	4.4296522	-4.1927413	-1.4704715
H	-0.7126058	-5.5800294	-4.1711621
H	-0.5924543	-6.9252263	3.8580271
C	-0.6896136	-8.0656419	2.0382378
H	1.6901976	-4.8039848	3.4873840
H	0.5730499	-3.7158886	2.6707893
H	0.0140838	-4.6991357	4.0388012
H	-0.5867877	-8.9800293	0.0840057
C	5.4149438	-4.5425096	2.3184670
H	4.7694554	-2.6761167	3.1825595
H	5.7868505	-6.4037575	1.3078628
H	3.1713843	-6.4541262	-1.1411422
H	4.5166892	-7.3215637	-0.3801169
H	2.8852176	-7.4379524	0.2940446
F	-5.8540275	0.6183303	-0.0588036
F	4.0069580	0.3786952	4.6401738
F	3.0917400	0.4955307	-5.3467934
H	1.2033192	-4.8220047	-5.5820093
H	-1.1879978	-8.9086306	2.5073348
H	6.3498268	-4.5731545	2.8699583
B	-0.2423987	4.0053342	0.2035993
C	0.1984628	4.2499591	1.7349913
C	0.8185848	4.3958583	-0.9427320
C	-1.7961362	4.2976411	-0.1315340
C	-0.5384757	5.0035966	2.6546395
C	1.3884967	3.7220111	2.2521753
C	0.6923401	3.9229789	-2.2566845
C	1.9157856	5.2368415	-0.7392742
C	-2.2269929	5.1858436	-1.1218541
C	-2.8364323	3.6890964	0.5860276
C	-0.1624289	5.1855030	3.9843095
F	-1.6745888	5.6451530	2.2882369

C	1.8042694	3.8853978	3.5673776
F	2.2222099	3.0310914	1.4477070
C	1.5941183	4.2129987	-3.2716913
F	-0.3647684	3.1577251	-2.6100409
C	2.8446428	5.5511516	-1.7294829
F	2.1289685	5.8291741	0.4610548
C	-3.5679999	5.4211129	-1.4202663
F	-1.3399211	5.9065232	-1.8492004
C	-4.1836560	3.8982167	0.3226464
F	-2.5625266	2.8734147	1.6294270
C	1.0144988	4.6151968	4.4501940
F	-0.9249743	5.9214908	4.8167231
F	2.9708990	3.3604472	3.9950107
C	2.6891916	5.0290745	-3.0059974
F	1.4205442	3.7120564	-4.5122443
F	3.8890926	6.3594421	-1.4591050
C	-4.5574080	4.7662847	-0.6988025
F	-3.9114834	6.2860183	-2.3945777
F	-5.1320397	3.2913371	1.0640732
F	1.3978434	4.7833507	5.7257755
F	3.5768508	5.3120129	-3.9726810
F	-5.8550167	4.9827374	-0.9661909
Cl	-0.3145573	1.8529504	0.0494298

TS5 : HBf_2 transfer to $ArCClN_2$
82
Energy = -6049.836026277

B	-0.2187995	0.8002974	1.0032848
C	0.6550000	-0.1710320	0.0961936
C	-1.0083136	2.0940816	0.5251237
H	-0.5672160	0.3403808	2.0466269
C	0.7466673	-1.5350684	0.3990740
C	1.4839425	0.2577380	-0.9445136
C	-1.3759538	2.4131351	-0.7846151
C	-1.4655664	2.9970633	1.4933466
C	1.5534218	-2.4223043	-0.3018181
F	0.0352318	-2.0516298	1.4255989
C	2.2979602	-0.5971191	-1.6785530
F	1.5653924	1.5765858	-1.2421698
C	-2.1252564	3.5353783	-1.1213299
F	-1.0489704	1.5956995	-1.8102047
C	-2.2157821	4.1304807	1.2019598
F	-1.1744270	2.7816289	2.8000105
C	2.3296443	-1.9496147	-1.3555130
F	1.6056541	-3.7242520	0.0323204
F	3.0746954	-0.1296645	-2.6743263
C	-2.5471952	4.4033554	-0.1215068
F	-2.4697393	3.7723047	-2.4002179
F	-2.6288261	4.9593855	2.1808543
F	3.1204873	-2.7888752	-2.0413173
F	-3.2859642	5.4823107	-0.4264028
P	-2.6471868	-0.8935011	0.0290786
C	-3.6040626	-1.6246594	1.4137840
C	-2.0133567	-2.1733866	-1.1200063
C	-3.9230539	0.1640071	-0.7752779
C	-2.9943861	-1.5053676	2.6727910
C	-4.8953943	-2.1912530	1.3164164
C	-1.3505444	-1.6132667	-2.2314916
C	-1.9100843	-3.5673074	-0.8989157
C	-4.3001736	1.1972483	0.1083759
C	-4.5081144	0.1212126	-2.0607076
C	-3.6299769	-1.9478374	3.8315430
H	-2.0055668	-1.0609669	2.7309241
C	-5.5207630	-2.6226508	2.4918474
C	-5.6063271	-2.3637345	0.0015266
H	-1.3797157	-0.5377655	-2.3672714
C	-0.6561514	-2.3922409	-3.1470174
C	-1.1952049	-4.3330448	-1.8341366
C	-2.4916837	-4.2877894	0.2927174
C	-5.2246403	2.1714238	-0.2440111
H	-3.8503962	1.2233000	1.0987491
C	-5.4175224	1.1354471	-2.4057641
C	-4.2583946	-0.9574722	-3.0845081
H	-3.1375337	-1.8523437	4.7950761
C	-4.9035918	-2.5067933	3.7382211
H	-6.5103937	-3.0689545	2.4235471
H	-6.0836707	-1.4289510	-0.3137297
H	-4.9099521	-2.6508589	-0.7919485
H	-6.3800570	-3.1324227	0.0814138
H	-0.1648218	-1.9240503	-3.9951548
C	-0.5794258	-3.7714204	-2.9488771
H	-1.1161128	-5.4042065	-1.6640883
H	-2.1371692	-3.8613238	1.2349798
H	-3.5838717	-4.2377945	0.3095351
H	-2.2017826	-5.3415846	0.2606971
C	-5.7774672	2.1507845	-1.5255518
H	-5.4987928	2.9451759	0.4674382
H	-5.8684346	1.1029793	-3.3951645
H	-3.3542023	-0.7572148	-3.6699499
H	-4.1251536	-1.9389046	-2.6260435
H	-5.1012059	-1.0052488	-3.7801006
H	-5.4172914	-2.8548141	4.6302282
H	-0.0304142	-4.4009515	-3.6432371
H	-6.4895559	2.9113572	-1.8327714
Br	6.9544935	-1.1400422	-1.1781934
C	5.6394042	-0.4167853	-0.0014629
C	5.1373912	-1.2113665	1.0280534
C	5.1972084	0.8902844	-0.1977835
C	4.1627971	-0.6868299	1.8722350
H	5.4963837	-2.2240930	1.1700986
C	4.2133220	1.4002177	0.6437882
H	5.6023137	1.4985451	-0.9979302
C	3.6879953	0.6146783	1.6779524
H	3.7680200	-1.2973995	2.6771222
H	3.8516582	2.4110413	0.4824149
C	2.5863603	1.1594667	2.5061742
N	1.3664821	1.5953192	1.8605216
N	2.0811335	2.5347013	2.2494400
Cl	2.3981222	0.5688207	4.1468464

TS6 : P…N addition of PTo_3

82

Energy = -6049.853693707

Br	5.6283027	-3.4520168	1.1915163
C	3.8743614	-2.6985889	1.1399059
C	3.0594201	-2.9571477	0.0386700
C	3.4478586	-1.8938755	2.1947375
C	1.7966903	-2.3738775	-0.0114076
H	3.4047103	-3.5915549	-0.7697294
C	2.1764268	-1.3272820	2.1380209
H	4.0959541	-1.7036831	3.0427918
C	1.3523707	-1.5553271	1.0324030
H	1.1590113	-2.5444892	-0.8737024
H	1.8358156	-0.6930684	2.9496613
C	0.0376417	-0.8662531	0.8928752
Cl	-0.9143613	-0.7861423	2.4013221
N	-0.1298065	0.1636244	-0.0524620
N	-0.6782371	-0.9732687	-0.4029300
B	0.5169179	1.3302226	-0.9273137
H	-0.1598910	1.3306133	-1.9173857
C	2.0164696	0.7834899	-1.2756339
C	0.4542627	2.7735330	-0.2043142
C	3.1318806	0.9474109	-0.4532783
C	2.2335185	0.0024983	-2.4138551
C	0.3646267	3.0188699	1.1634209
C	0.4340949	3.9162659	-1.0052109
F	3.0305921	1.6531656	0.7004192
C	4.3780047	0.3896306	-0.7251666
F	1.2137357	-0.2609839	-3.2675170
C	3.4625941	-0.5700045	-2.7275931
F	0.3997498	1.9897797	2.0438784
C	0.2269842	4.2907536	1.7104618
F	0.5344236	3.7940575	-2.3531919
C	0.3070383	5.2091114	-0.5051475
F	5.4161554	0.5643962	0.1171326
C	4.5439344	-0.3769981	-1.8735821
F	3.6144490	-1.3276554	-3.8339213
F	0.1211743	4.4630291	3.0449649
C	0.1960942	5.3972516	0.8690039
F	0.2883404	6.2763823	-1.3307825
F	5.7336381	-0.9396961	-2.1481769
F	0.0682677	6.6366955	1.3782552
P	-3.1885770	-0.9325383	-0.1246952
C	-3.4708267	0.6590299	0.6699189
C	-3.6910515	-0.7743671	-1.8645330
C	-4.0785405	-2.3519524	0.5806052
C	-4.0044221	0.8724117	1.9659819
C	-2.8934704	1.7402143	-0.0319784
C	-4.5044608	0.2875337	-2.2945608
C	-3.2353986	-1.7350447	-2.7951464
C	-3.2815360	-3.2865731	1.2618350
C	-5.4605779	-2.5891441	0.4017256
C	-4.5916301	-0.2193660	2.8230580
C	-3.9696599	2.1738247	2.4776774
H	-2.4635866	1.5646490	-1.0130309
C	-2.8883457	3.0255234	0.4985867
H	-4.8613920	1.0151187	-1.5734677
C	-4.8729961	0.4088532	-3.6295646
C	-3.6322994	-1.5996162	-4.1310625
C	-2.3260630	-2.8779677	-2.4156427
H	-2.2219995	-3.0852194	1.3853352
C	-3.8342850	-4.4552982	1.7785489
C	-6.3720667	-1.6098755	-0.2888895
C	-5.9900438	-3.7764278	0.9214604
H	-5.3671935	-0.7821461	2.2980493
H	-5.0322621	0.2140928	3.7243034
H	-3.8264491	-0.9410018	3.1294905
H	-4.3878510	2.3489721	3.4657558
C	-3.4291898	3.2436663	1.7628261
H	-2.4558450	3.8421889	-0.0701455
H	-5.5062476	1.2335960	-3.9429015
C	-4.4351697	-0.5418188	-4.5527951
H	-3.2947278	-2.3399257	-4.8523022
H	-1.2788250	-2.5764991	-2.5237077
H	-2.5022960	-3.7383460	-3.0674035
H	-2.4696444	-3.1990779	-1.3806241
H	-3.2080497	-5.1668468	2.3081698
C	-5.1956110	-4.7015195	1.5995759
H	-6.1192249	-0.5759860	-0.0324464
H	-7.4111591	-1.7941507	-0.0046881
H	-6.2955824	-1.6988693	-1.3780579
H	-7.0534907	-3.9692105	0.8006795
H	-3.4265789	4.2387098	2.1979408
H	-4.7197275	-0.4615984	-5.5979328
H	-5.6431589	-5.6105892	1.9917487

TS7a : chloride anion transfer from borate to **H^+**

106

Energy = -7531.946771701

Br	6.7047008	3.9652445	1.6867739
P	2.9436301	-1.2399987	-1.2830005
F	-2.5608648	-1.5446359	3.0667561
F	-1.1419637	2.6799636	1.3524315
F	1.4679222	-1.4837663	2.8593354
F	-2.2866455	-2.3906897	0.0679219
F	-1.7297814	-4.9891486	0.1844362
F	2.0014895	-4.0907870	2.9591518
F	-4.4887738	-0.3220006	4.4288520
F	0.4193475	-5.8925975	1.6269368
F	-3.1190925	3.8694136	2.7038068
N	0.6900667	0.6106361	1.2808065
H	0.7059379	1.4741744	1.8109553
F	-4.8138261	2.3903698	4.2690309
N	1.8335843	-0.5991255	-0.2913396
C	1.8079412	0.3914509	0.5674307
C	-0.3719197	-1.7773041	1.3525609
C	2.9427828	1.3142933	0.8329586
C	-1.1784678	-2.7458515	0.7495343
C	-1.7490824	0.5006843	2.1178861

C	-2.6398299	-0.2006043	2.9382775
C	-1.9547734	1.8864736	2.0948542
C	2.6838053	-3.0267426	-1.2703423
C	1.4678218	-3.5891522	-1.7294312
C	0.6730056	-2.3019317	2.1211435
C	3.3516118	1.5500169	2.1520498
H	2.8256721	1.0775156	2.9761614
C	2.5898338	-0.6212982	-2.9443185
C	4.4667676	2.3416774	2.4154333
H	4.8026315	2.4979699	3.4340880
C	4.6764484	-0.8644925	-0.8887970
C	1.7112921	0.4683628	-3.0421683
H	1.2609123	0.8759071	-2.1441499
C	0.3358576	-2.7743390	-2.2956187
H	0.5523917	-2.4820875	-3.3300980
H	0.1531619	-1.8591889	-1.7286952
H	-0.5844192	-3.3641727	-2.3055638
C	5.4557447	-0.2239762	-1.8682054
H	5.0400777	-0.0482001	-2.8548135
C	0.9617257	-3.6593675	2.2168610
C	-0.9260830	-4.1126499	0.8190098
C	3.6310073	1.9239771	-0.2217465
H	3.3021463	1.7706122	-1.2446561
C	5.1961683	-1.0611374	0.4107583
C	3.1243843	-1.2207555	-4.1083217
C	3.7302163	-3.8562601	-0.8395872
H	4.6754922	-3.4169624	-0.5400953
C	1.3441871	-4.9830231	-1.6847297
H	0.4129755	-5.4306127	-2.0221026
C	0.1604914	-4.5774437	1.5493726
C	-3.6587349	0.4109272	3.6641325
C	5.1490505	2.9147677	1.3442695
C	3.5780807	-5.2382136	-0.8057378
H	4.3931914	-5.8655954	-0.4594533
C	-2.9668040	2.5346785	2.7900803
C	1.3692895	0.9941854	-4.2836661
H	0.6825230	1.8324948	-4.3434270
C	4.4364582	-1.7429104	1.5200344
H	4.4059936	-1.1047910	2.4077259
H	4.9343401	-2.6760747	1.8054339
H	3.4113167	-1.9858595	1.2419227
C	2.7716585	-0.6635131	-5.3431532
H	3.1818812	-1.1044810	-6.2479633
C	4.7332666	2.7342433	0.0261853
H	5.2665034	3.2045296	-0.7914669
C	2.3728973	-5.8018050	-1.2230533
H	2.2350222	-6.8786534	-1.1983077
C	-3.8291078	1.7876625	3.5863180
C	6.7385269	0.2238687	-1.5783910
H	7.3241222	0.7220536	-2.3443723
C	6.4866437	-0.5885420	0.6797588
H	6.8900231	-0.7206988	1.6804590
C	1.9089521	0.4281835	-5.4379677
H	1.6533904	0.8288661	-6.4145371
B	-0.5932605	-0.1824337	1.2429747
C	4.0182224	-2.4359946	-4.0841925
H	4.7354146	-2.4141391	-3.2591818
H	4.5758278	-2.5102663	-5.0211560
H	3.4232514	-3.3496963	-3.9713921
C	7.2522772	0.0474166	-0.2929440
H	8.2456787	0.4101445	-0.0466878
B	-2.8394796	0.8547769	-1.8430755
C	-3.9227623	-0.2680159	-1.5012068
C	-3.0863933	2.3968500	-1.4694905
C	-4.1211105	-1.3493620	-2.3637567
C	-4.6398127	-0.3359132	-0.3054926
C	-2.0365043	3.3244499	-1.4635359
C	-4.3476602	2.9608061	-1.2488984
C	-4.9615759	-2.4200820	-2.0711220
F	-3.4663253	-1.4119739	-3.5484094
C	-5.4854172	-1.3849016	0.0311744
F	-4.5023684	0.6429897	0.6193964
C	-2.1954706	4.6720467	-1.1581428
F	-0.7767215	2.9350831	-1.7683248
C	-4.5535094	4.3045011	-0.9477824
F	-5.4703297	2.2073163	-1.3526669
C	-5.6483324	-2.4392874	-0.8624167
F	-5.1094646	-3.4428074	-2.9391157
F	-6.1368766	-1.3995361	1.2123405
C	-3.4660192	5.1676698	-0.8888780
F	-1.1339963	5.5039943	-1.1328085
F	-5.7974167	4.7799799	-0.7313265
F	-6.4592478	-3.4678676	-0.5579085
F	-3.6428077	6.4685181	-0.5969898
Cl	-1.1263657	0.1655573	-0.8109519
H	-2.3339393	0.7226533	-2.9193406

TS7$^+$: B-to-N 1,2-H-shift within **G$^+$**

81

Energy = -5589.441905948

Br	6.1266733	-2.8328926	0.3970959
C	4.3494366	-2.2299311	0.1470913
C	3.5123028	-2.9260927	-0.7312895
C	3.9091458	-1.1039936	0.8515358
C	2.2101488	-2.4838656	-0.9120456
H	3.8774068	-3.7943800	-1.2666821
C	2.6115693	-0.6583892	0.6564949
H	4.5685680	-0.5950665	1.5445560
C	1.7585544	-1.3406399	-0.2318384
H	1.5558496	-2.9999656	-1.6038750
H	2.2444729	0.1973641	1.2119268
C	0.4384838	-0.8174683	-0.4757860
N	-0.0788889	0.3267353	-0.2525132
N	-0.6513717	-1.3284444	-1.1516215
B	-0.1647268	1.8003984	-0.6378379
P	-2.1546522	-1.8149013	-0.5506907
H	-0.6636172	1.8342582	-1.7394842
C	1.4478582	2.0905020	-0.7763224
C	-1.0039053	2.6637480	0.4189850
C	-3.1055801	-0.3240928	-0.2228791

C	-2.9014065	-2.6909619	-1.9377934
C	-2.0062509	-2.9533459	0.8382209
C	2.2312605	2.7931362	0.1459577
C	2.1349217	1.5530154	-1.8729249
C	-1.0550954	2.3834931	1.7842807
C	-1.7571150	3.7616403	0.0061222
C	-3.5250699	0.1293807	1.0500596
C	-3.3786943	0.4343931	-1.3825467
C	-4.2190922	-2.3726112	-2.3117498
C	-2.2167167	-3.7530540	-2.5734397
C	-0.7424298	-3.1764891	1.4064546
C	-3.1411547	-3.6515287	1.3149760
F	1.6863708	3.3192919	1.2607113
C	3.5969113	2.9973835	-0.0337188
F	1.4608990	0.8096919	-2.7771693
C	3.4958034	1.7271851	-2.0809334
F	-0.3348245	1.3479375	2.2870115
C	-1.8014127	3.1254486	2.6911745
F	-1.7731953	4.1153563	-1.2996606
C	-2.5157182	4.5373620	0.8782057
C	-3.2010137	-0.5312110	2.3659315
C	-4.2945366	1.3017369	1.0818396
H	-3.0014280	0.1013048	-2.3443299
C	-4.1238947	1.6011614	-1.3082302
H	-4.7527982	-1.5800184	-1.8011365
C	-4.8587657	-3.0799875	-3.3223828
C	-2.8866712	-4.4475179	-3.5863752
C	-0.8050891	-4.1483863	-2.2363532
H	0.1261273	-2.6459764	1.0387469
C	-0.5809154	-4.0823783	2.4480369
C	-4.5298151	-3.4606698	0.7642321
C	-2.9468495	-4.5586594	2.3621248
F	4.3077673	3.7017768	0.8600814
C	4.2342105	2.4541350	-1.1474705
F	4.1077543	1.1960912	-3.1503557
F	-1.8261019	2.8040850	3.9982210
C	-2.5419866	4.2116940	2.2307604
F	-3.2407455	5.5772171	0.4275490
H	-3.8364359	-1.4037722	2.5485734
H	-3.3785526	0.1782582	3.1771724
H	-2.1622322	-0.8603977	2.4222988
H	-4.6462995	1.6554824	2.0466269
C	-4.6039040	2.0224675	-0.0669178
H	-4.3238700	2.1745727	-2.2067192
H	-5.8768670	-2.8242067	-3.5967400
C	-4.1866621	-4.1195666	-3.9647434
H	-2.3704851	-5.2643575	-4.0830175
H	-0.1002583	-3.5112350	-2.7820543
H	-0.6175058	-5.1835997	-2.5311934
H	-0.5939959	-4.0606068	-1.1658193
H	0.4012345	-4.2410835	2.8809368
C	-1.6910746	-4.7782199	2.9250227
H	-4.7256527	-2.4238866	0.4728174
H	-5.2712834	-3.7453008	1.5143076
H	-4.6884989	-4.0866598	-0.1207121
H	-3.8093691	-5.0958400	2.7466641
F	5.5472732	2.6302491	-1.3217694
F	-3.2838492	4.9320801	3.0850317
H	-5.2036644	2.9243023	0.0082016
H	-4.6763923	-4.6812137	-4.7543244
H	-1.5815919	-5.4889133	3.7381960

TS8 : HBf_2 transfer to $ArCClN_2$

73

Energy = -6428.239077125

B	-0.8327363	0.0636656	0.5053945
C	0.4155381	-0.3579637	-0.4044869
C	-1.6656007	1.3913068	0.1914733
C	-1.3175449	-0.9079643	1.6942199
C	0.7818211	-1.6910714	-0.6228879
C	1.2454222	0.5742448	-1.0404938
C	-1.8300412	1.9187202	-1.0959172
C	-2.3081974	2.1277362	1.1986710
C	-2.6516015	-1.0480412	2.0885659
C	-0.4209809	-1.6557191	2.4704053
C	1.8677482	-2.0780144	-1.3923197
F	0.0237484	-2.6930856	-0.1298454
C	2.3272158	0.2287682	-1.8402081
F	1.0358449	1.9001004	-0.8834941
C	-2.6111128	3.0324018	-1.3836193
F	-1.2727682	1.3027305	-2.1664323
C	-3.1031687	3.2446203	0.9571208
F	-2.1878779	1.7584267	2.4977712
C	-3.0705863	-1.8310155	3.1577037
F	-3.6382833	-0.4115700	1.4103631
C	-0.7925370	-2.4533233	3.5468831
F	0.9059771	-1.6272976	2.2018265
C	2.6503957	-1.1094429	-2.0088467
F	2.1421484	-3.3771737	-1.5851937
F	3.0697502	1.1775820	-2.4334189
C	-3.2604964	3.7011660	-0.3477037
F	-2.7433637	3.4712145	-2.6489769
F	-3.7093836	3.8927289	1.9736700
C	-2.1323589	-2.5396650	3.9009306
F	-4.3746739	-1.9097388	3.4750480
F	0.1329684	-3.1369790	4.2454047
F	3.6924163	-1.4658648	-2.7690014
F	-4.0192547	4.7787451	-0.6037454
F	-2.5150959	-3.2980248	4.9351489
H	-5.0785646	-0.4102344	-0.2514603
C	-5.0097586	-0.0287336	-1.2631387
C	-3.9027854	-0.3671757	-2.0581441
C	-6.0138420	0.7969284	-1.7652764
P	-2.5317587	-1.3106359	-1.3018970
C	-3.8335509	0.1204453	-3.3713529
C	-5.9256825	1.2993597	-3.0659497
H	-6.8661896	1.0467582	-1.1399752
C	-1.7411871	-2.2203358	-2.6607089
H	-3.2629346	-2.3629874	-0.7058603
C	-4.8397410	0.9487735	-3.8697619

H	-2.9915820	-0.1408440	-4.0047208
H	-6.7057831	1.9475808	-3.4547483
C	-0.7896240	-1.5692625	-3.4640713
C	-1.9925898	-3.5859200	-2.8643390
H	-4.7698065	1.3251762	-4.8871528
H	-0.5927639	-0.5107961	-3.3154347
C	-0.1203759	-2.2701340	-4.4671094
C	-1.3098954	-4.2858320	-3.8578769
H	-2.7240449	-4.0975545	-2.2439353
H	0.6037228	-1.7574502	-5.0944514
C	-0.3749220	-3.6292148	-4.6620665
H	-1.5107547	-5.3436793	-4.0050315
H	0.1553912	-4.1758483	-5.4367148
Cl	1.7565077	0.8508213	4.2395253
C	1.8036588	1.2593092	2.5305923
C	3.0808270	1.0595615	1.8044668
N	0.5336316	1.1302378	1.8242364
N	0.8563206	2.2975260	2.0897014
C	3.7454075	-0.1692717	1.8528596
C	3.5895126	2.0962663	1.0195517
C	4.8922933	-0.3754290	1.0918113
H	3.3637759	-0.9710721	2.4726106
C	4.7434588	1.9052002	0.2627890
H	3.0776497	3.0517169	0.9800910
C	5.3691484	0.6630645	0.2956435
H	5.3989649	-1.3321686	1.1118937
H	5.1314900	2.7049593	-0.3539269
Br	6.9176208	0.3711908	-0.7812249

TS9 : P…N addition of $HPPh_2$ to **A**

73

Energy = -6428.248333846

Br	6.4691356	2.3502578	0.2690095
C	4.6524440	1.9955848	-0.1961175
C	4.2887503	1.9437172	-1.5395316
C	3.7182159	1.7980171	0.8194219
C	2.9591907	1.6910151	-1.8704195
H	5.0257952	2.1000142	-2.3187320
C	2.3957579	1.5408651	0.4763334
H	4.0161383	1.8477607	1.8606187
C	2.0077701	1.4866098	-0.8687064
H	2.6685742	1.6543225	-2.9138844
H	1.6607845	1.3992463	1.2607536
C	0.5734354	1.2329387	-1.1757842
Cl	0.0453773	1.6167691	-2.8214392
N	-0.1685203	0.2316994	-0.4962585
N	-0.4016960	1.4190202	-0.0598595
B	-0.7260877	-1.2803407	-0.2338367
C	-0.5792342	-2.0250942	-1.6792744
C	0.2577334	-1.9115982	0.9127584
C	-2.2649356	-1.0008524	0.2291257
C	-1.5798310	-2.7761443	-2.2970885
C	0.6188775	-1.9639472	-2.3931781
C	0.8585763	-3.1708085	0.8322479
C	0.5814374	-1.1981137	2.0697972
C	-3.1216042	-0.3003930	-0.6263058
C	-2.8456899	-1.3838108	1.4387514
F	-2.7730483	-2.9825804	-1.6882910
C	-1.4282079	-3.3776593	-3.5445407
F	1.6926454	-1.3354199	-1.8455654
C	0.8160962	-2.5404812	-3.6416527
F	0.5944102	-4.0103604	-0.1969119
C	1.7374148	-3.6671696	1.7942576
F	-0.0021127	0.0079749	2.3019729
C	1.4587287	-1.6452533	3.0489691
F	-2.6776094	0.0764338	-1.8523017
C	-4.4381395	0.0200623	-0.3229961
F	-2.1505633	-2.0994105	2.3551404
C	-4.1617023	-1.0827574	1.7859110
F	-2.4366841	-4.0827871	-4.0929527
C	-0.2244329	-3.2530986	-4.2283661
F	1.9994530	-2.4321640	-4.2758044
F	2.2822647	-4.8902956	1.6554535
C	2.0498347	-2.8969269	2.9076913
F	1.7296330	-0.8903321	4.1311736
F	-5.2029178	0.7010029	-1.1981561
C	-4.9653529	-0.3737727	0.9021108
F	-4.6599310	-1.4663732	2.9771528
F	-0.0631092	-3.8250976	-5.4326464
F	2.8964309	-3.3589036	3.8407570
F	-6.2331520	-0.0768672	1.2247538
P	-2.0886761	3.0681603	-0.6154457
H	-2.9241340	3.7293618	-1.5441252
C	-0.7855038	4.2864953	-0.3414037
C	-3.1583380	2.9578134	0.8353318
C	-0.2672873	4.9788890	-1.4499488
C	-0.1474283	4.3990294	0.9034892
C	-4.4695012	3.4591893	0.7738987
C	-2.7300339	2.2861764	1.9934927
H	-0.7473777	4.8907809	-2.4206770
C	0.8669249	5.7739936	-1.3078212
H	-0.5416917	3.8732600	1.7674134
C	0.9882856	5.1962379	1.0371598
H	-4.8157502	3.9667655	-0.1218890
C	-5.3307060	3.3016344	1.8581455
H	-1.7341313	1.8619925	2.0398376
C	-3.5932123	2.1448612	3.0776033
H	1.2585496	6.3096239	-2.1675414
C	1.5007665	5.8791639	-0.0666891
H	1.4736815	5.2809395	2.0049927
H	-6.3433417	3.6891027	1.7996747
C	-4.8939656	2.6487847	3.0125341
H	-3.2533151	1.6271325	3.9697006
H	2.3885799	6.4954598	0.0390341
H	-5.5675855	2.5256322	3.8551569

TS10 : HCl elimination from **I**

73

Energy = -6428.256017716

Br	6.5575358	0.9590781	-1.9152988

Cl	-0.0418402	2.0496957	-3.9452031
P	-1.2802624	2.8142088	-1.1148476
F	1.8701193	-2.5308751	0.2986636
F	3.9711748	-1.8557394	1.7730774
F	-1.8587980	-1.4348322	3.0316303
F	-0.6844263	0.8918015	2.4529948
F	-1.1545553	-3.6357360	1.4381907
F	-0.2260760	-1.6425840	-2.7876059
F	-3.0267076	-0.5367071	-1.5044230
F	-5.6019493	-0.3820590	-0.7920602
F	3.8161013	0.1998294	3.5811149
F	-1.2651153	-6.0486241	0.2601815
F	-4.4225979	-1.2064565	3.7184910
F	1.4507365	1.5520067	3.8831978
F	-6.3345322	-0.6728238	1.8327362
F	-0.3385541	-4.0443341	-3.9308363
F	-0.8552615	-6.2866250	-2.4425527
N	-0.4835207	0.0855799	-0.8724731
H	-1.0059158	2.4929620	-2.6547225
N	-0.3043816	1.6247696	-0.2874407
C	-0.7100970	-2.5148480	-0.6102131
C	0.5693153	0.8367027	-0.9912225
C	0.4524801	-0.8752133	1.2987300
C	1.9499022	0.8706151	-1.2966938
C	1.6856351	-1.5284810	1.1940695
C	-0.3097308	4.2582189	-0.6621216
C	0.4366654	0.1602250	2.2411822
C	2.8113906	-1.1928625	1.9434864
C	-2.2926455	-0.9229961	0.7339162
C	-3.3177477	-0.6809328	-0.1881018
C	-0.9656223	-3.6927275	0.0969185
C	-2.8723597	2.7639562	-0.2963272
C	-2.7301662	-1.1211665	2.0454323
C	2.6970469	1.9707371	-0.8265601
H	2.1927606	2.7807825	-0.3093899
C	-0.5009836	-2.6958785	-1.9761536
C	2.7404362	-0.1493366	2.8576819
C	-1.0209571	-4.9532814	-0.4850842
C	1.5375049	0.5317311	3.0068237
C	-4.0658906	-1.0299616	2.4302177
C	0.1171754	5.1049955	-1.6968452
H	-0.1621844	4.8926058	-2.7242915
C	-4.6641794	-0.6023599	0.1496050
C	0.9186595	6.2060565	-1.3978035
H	1.2485535	6.8634376	-2.1959389
C	-4.0038810	2.8043718	-1.1260587
H	-3.8880245	2.8248927	-2.2057516
C	0.0793956	4.5067423	0.6657426
H	-0.2311245	3.8410231	1.4641981
C	4.0693588	1.9970204	-1.0034822
H	4.6615662	2.8256297	-0.6339047
C	2.5811003	-0.1888737	-1.9784488
H	1.9932670	-1.0160595	-2.3561250
C	3.9509213	-0.1558350	-2.1720045
H	4.4536837	-0.9582260	-2.6981817
C	-5.0417932	-0.7645985	1.4772794
C	-0.5495930	-3.9391851	-2.6042961
C	4.6811657	0.9305085	-1.6733572
C	-3.0166093	2.7162863	1.0999195
H	-2.1465219	2.6572534	1.7418022
C	1.3023495	6.4539188	-0.0790282
H	1.9325679	7.3080576	0.1492555
C	-0.8087609	-5.0795452	-1.8544552
B	-0.7568876	-1.0970115	0.2114446
C	-5.2763565	2.8160991	-0.5570033
H	-6.1522631	2.8463704	-1.1964767
C	0.8862166	5.6039226	0.9510238
H	1.1916117	5.7976432	1.9745735
C	-4.2911515	2.7114781	1.6556005
H	-4.4072021	2.6646173	2.7339058
C	-5.4187115	2.7689694	0.8296697
H	-6.4105882	2.7663420	1.2711797

Computational references

[1] *TURBOMOLE V7.4*, **2019**, a development of University of Karlsruhe and Forschungszentrum Karlsruhe GmbH, 1989-2007, TURBOMOLE GmbH, since 2007; available from http://www.turbomole.com.

[2] a) P. Pracht, F. Bohle, S. Grimme, *Phys. Chem. Chem. Phys.* **2020**, *22*, 7169-7192; b) S. Grimme, *J. Chem. Theory Comput.* **2019**, *15*, 2847-2862.

[3] J. Tao, J. P. Perdew, V. N. Staroverov, G. E. Scuseria, *Phys. Rev. Lett.* **2003**, *91*, 146401.

[4] a) S. Grimme, J. Antony, S. Ehrlich, H. Krieg, *J. Chem. Phys.* **2010**, *132*, 154104-154119; b) S. Grimme, S. Ehrlich, L. Goerigk, *J. Comput. Chem.* **2011**, *32*, 1456-1465.

[5] a) F. Weigend, M. Häser, H. Patzelt, R. Ahlrichs, *Chem. Phys. Lett.* **1998**, *294*, 143-152; b) F. Weigend, R. Ahlrichs, *Phys. Chem. Chem. Phys.* **2005**, *7*, 3297-3305.

[6] A. Klamt, G. Schüürmann, *J. Chem. Soc., Perkin Trans. 2* **1993**, 799-805.

[7] a) K. Eichkorn, F. Weigend, O. Treutler, R. Ahlrichs, *Theor. Chem. Acc.* **1997**, *97*, 119-124; b) F. Weigend, *Phys. Chem. Chem. Phys.* **2006**, *8*, 1057-1065.

[8] P. Deglmann, K. May, F. Furche, R. Ahlrichs, *Chem. Phys. Lett.* **2004**, *384*, 103-107.

[9] S. Grimme, *Chem. Eur. J.* **2012**, *18*, 9955-9964.

[10] F. Eckert, A. Klamt, *AIChE J.* **2002**, *48*, 369-385.

[11] Eckert, F.; Klamt, A. *COSMOtherm, Version C3.0, Release 16.01; COSMOlogic GmbH & Co. KG, Leverkusen, Germany* **2015**.

[12] Y. Zhao, D. G. Truhlar, *J. Phys. Chem. A* **2005**, *109*, 5656-5667.

[13] F. Weigend, F. Furche, R. Ahlrichs, *J. Chem. Phys.* **2003**, *119*, 12753-12762.

[14] L. Goerigk, A. Hansen, C. Bauer, S. Ehrlich, A. Najibi, S. Grimme, *Phys. Chem. Chem. Phys.* **2017**, *19*, 32184-32215.

[15] G. Schreckenbach, T. Ziegler, *J. Phys. Chem.* **1995**, *99*, 606-611.
